# Three decades of atrial fibrillation and flutter epidemiology and risk factors in Iran with a focus on the impact of COVID-19

**DOI:** 10.1038/s41598-025-91737-y

**Published:** 2025-03-20

**Authors:** Hoomaan Ghasemi, Mohammad-Mahdi Bastan, Morvarid Najafi, Seyed Aria Nejadghaderi

**Affiliations:** 1 Center for Orthopedic Trans-Disciplinary Applied Research, Tehran University of Medical Sciences, Tehran, Iran; 2 HIV/STI Surveillance Research Center, and WHO Collaborating Center for HIV Surveillance, Institute for Futures Studies in Health, Kerman University of Medical Sciences, Kerman, Iran

**Keywords:** Iran, Global burden of disease, Epidemiology, Atrial fibrillation, Atrial flutter, Risk factors, Atrial fibrillation, Epidemiology

## Abstract

Atrial fibrillation/flutter (AF/AFL) is one of the most common sustained heart rhythm disorders in clinical practice and a major public health concern. This study aimed to evaluate the disease burden of AF/AFL in Iran and analyze trends using the Global Burden of Disease (GBD) 2021 data, by age, sex, location, risk factor, and socio-demographic index (SDI), considering the impacts of COVID-19. Data on the prevalence, incidence, disability-adjusted life years (DALYs), deaths, and six attributable risk factors related to AF/AFL in Iran and its 31 provinces from 1990 to 2021 was collected from the GBD 2021 study. The International Classification of Disease (ICD) codes used were I48–48.9 for ICD-10 and 427.3 for ICD-9. The data was sourced from surveys, censuses, vital statistics, and other health-related records. In Iran, in 2021, the AF/AFL age-standardized incidence rate (ASIR) was 40.6 (30.0 to 54.4), the age-standardized prevalence rate (ASPR) was 425.4 (327.2 to 559.2), the age-standardized DALY rate was 72.4 (57.4 to 88.3), and the age-standardized death rate (ASDR) was 3.3 (2.5 to 3.8) per 100,000 population. Following COVID-19, there were significant decreases in age-standardized DALY and death rates over 2019–2021. By province, Fars had the highest AF/AFL ASIR and ASPR in 2021. In addition, East and West Azarbayejan had the highest age-standardized DALY rate and ASDR, respectively. The national incidence, prevalence, DALYs, and death rates of AF/AFL showed an overall increasing trend with age. Males experienced higher rates of incidence and prevalence compared to females. In contrast, females had higher rates of DALYs and deaths compared to males. The burden of AF/AFL increased with advancing age. The risk factor with the highest DALYs and deaths attributable to AF/AFL was high systolic blood pressure. Notably, no remarkable association was found between SDI and the burden of AF/AFL in Iran. The incidence and prevalence of AF/AFL in Iran have significantly increased, highlighting the critical need for cost-effective and nationwide interventions.

**Supplementary Information:**

The online version contains supplementary material available at 10.1038/s41598-025-91737-y.

## Introduction

Atrial fibrillation/flutter (AF/AFL) is the most prevalent sustained heart rhythm disorder encountered in clinical settings^1,2^; it raises the risk of severe complications, including myocardial infarction, heart failure, stroke, and potentially death^3^. AF/AFL is strongly associated with multiple risk factors, among which hypertension is the most critical^4–6^. Other significant risk factors include smoking, alcohol use, high sodium intake, and obesity^7^. Socio-demographic aspects like population size, age, race, as well as socio-economic factors such as income, education, and healthcare access also play major roles in the burden of AF/AFL^7^. The prevalence of AF/AFL is anticipated to increase significantly over the next 30–50 years^8^; with significant post-AF/AFL care requirements and substantial treatment costs^2^.

Studies from the Global Burden of Disease (GBD) 2017^9^, 2019^1,2^, and 2021^10^ congruently confirm that AF/AFL remains a significant global health concern. The study using GBD 2017 data showed that while age-standardized rates of prevalence, incidence, and disability-adjusted life years (DALYs) for AF/AFL declined from 1990 to 2017, the absolute numbers of cases and DALYs increased, indicating a persistently high burden^9^. Similarly, GBD 2019 studies reported that from 1990 to 2019, there were no significant changes in age-standardized incidence rates (ASIRs), age-standardized death rates (ASDRs), or age-standardized DALY rates^1,2^. The study on GBD 2021 further revealed that globally, there were 4.48 million incident cases, 8.36 million DALYs, and 0.34 million deaths attributed to AF/AFL in 2021; despite declining age-standardized rates, the absolute number of cases has doubled due to factors like population growth, aging, improved diagnostics, and increased awareness^10^. Regarding Iran, a study on the burden of AF/AFL in the Middle East and North Africa (MENA) region found minimal changes in the ASIR of AF/AFL from 1990 to 2019. However, there was a slight increase in the ASDR and age-standardized DALY rates over the same period. According to this study, Iran had one of the region’s highest AF/AFL ASIRs in 2019^11^.

According to the World Health Organization (WHO), as of January 2025, there have been more than 7.6 million confirmed cases of COVID-19 and nearly 147,000 related deaths in Iran^12^. The COVID-19 pandemic significantly impacted AF/AFL rates, influencing both new diagnoses and treatment initiation. Previous literature reported that new AF/AFL diagnoses dropped by 35% post-pandemic onset, from 1.14 to 0.74 per 1000 individuals, with a consistent decline across outpatient (37%) and inpatient settings (29%)^13^. However, other studies state that a high frequency of AF/AFL cases has been observed in individuals with COVID-19 due to its association with increased inflammatory markers^14^. Regarding treatments, research shows the initiation of oral anticoagulation treatments remained stable, although electrical cardioversion rates fell significantly by 35% in April 2020 compared to the previous year^13,15^. These conflicting findings on the impact of COVID-19 on AF/AFL indicate the need for additional research.

While previous studies using GBD data have highlighted the global, regional, or national burden of AF/AFL^1,7,10,11,16–18^, an analysis explicitly focused on Iran and its subnational regions with specific attention to the impact of COVID-19 is lacking. This targeted study would provide policymakers with the necessary insights to develop comprehensive and effective strategies for controlling and preventing AF/AFL within the country, including data following the COVID-19. Our study aimed to evaluate the disease burden associated with AF/AFL in Iran and to examine the trends in AF/AFL occurrences over 1990–2021, by age, sex, location, and socio-demographic index (SDI). This included analyzing national and subnational prevalence, incidence, death, DALYs, and risk factors. Additionally, we conducted a separate analysis using 2019 as the baseline year before the COVID-19 pandemic and 2021 as the last year for which data were available. The percent changes in age-standardized rates between 2019 and 2021 were considered as indicators of the impact of COVID-19 on the burden of AF/AFL.

## Methods

### Overview

We collected data on the prevalence, incidence, DALY, and deaths related to AF/AFL and the attributable risk factors in Iran and its 31 provinces from 1990 to 2021 from the GBD study 2021. The GBD 2021 study provided data on 88 risk factors, 288 causes of death, and life expectancy decomposition across 204 countries and territories, as well as 811 subnational locations for the same period^19,20^. The specifics of the methodology used for estimating the burden of diseases and risk factors can be found in other sources^19,20^. The GBD study adhered to the Guidelines for Accurate and Transparent Health Estimates Reporting (GATHER)^21^.

### Case definition

The case definition for AF/AFL includes: (i) irregularly irregular RR intervals (excluding cases of complete atrioventricular block), (ii) absence of distinct P waves on the surface electrocardiogram, and (iii) a variable atrial cycle length, typically not exceeding 200 milliseconds. The International Classification of Disease (ICD) codes used for hospital and claims data inclusion were I48-I48.9 for ICD-10 and 427.3 for ICD-9 (https://www.healthdata.org/gbd/methods-appendices-2021/atrial-fibrillation-and-flutter)^19^.

### Data sources

GBD 2021 incorporates a wide array of data sources, including surveys, censuses, vital statistics, and other health-related data, to estimate morbidity, illness, injury, and attributable risk across 204 countries and territories from 1990 to 2021. It also provides cause-specific death estimates from 1990 to 2021. These data sources can be accessed through an interactive citation tool on the Global Health Data Exchange (GHDx) website. The GBD 2021 database uses standardized geospatial estimation models and small-area estimation techniques to address changes in administrative boundaries over time. For provinces that were established as independent administrative divisions after 1990, data for earlier years was retrospectively estimated based on population distribution and other regional characteristics derived from the larger original province. For specific citations related to GBD components, causes, risks, and locations, the GBD Sources Tool can be used which is available at http://ghdx.healthdata.org/gbd-2021/sources.

### Modeling strategy

The modeling strategy described in this section was performed by the GBD research team. Specifically, the calculation of excess deaths and their application to all countries and time periods is part of the standardized approach used in the GBD database. Excess deaths were initially estimated using prevalence and cause-of-death data from countries with the highest ratio of cause-specific deaths to prevalence, indicating likely under-reporting. DisMod-MR 2.1 was then used to estimate cause-specific death rates using these excess death estimates, which were applied consistently across countries and time periods as part of the uniform methodology of the GBD studies. Detailed methods are available in the GBD capstone paper^19^.

### Years of life lost and years lived with disability

The concept of years of life lost (YLLs) is based on the estimated number of deaths multiplied by predicted life expectancy, accounting for age, sex, location, and year. Years lived with disability (YLDs) were calculated by adjusting YLLs to account for health status, weighted by the severity of the disability. The total DALYs were obtained by adding YLLs and YLDs together^19,20^. The framework known as cause of death ensemble modelling (CODEm) was used to model the majority of cause-specific death rates in the GBD^19^.

### Risk factors

The GBD 2021 study identified six risk factors for AF/AFL, including high body-mass index (BMI), high systolic blood pressure (SBP), smoking, high alcohol use, diet high in sodium, and lead exposure. Detailed definitions of these risk factors and their contributions to AF/AFL-related deaths have been reported in previous studies^20^.

### Statistical analysis

To analyze the time trend of the burden attributable to AF/AFL, we calculated age-standardized rates and their estimated percentage changes from 1990 to 2021 and 2019 to 2021 for prevalence, incidence, deaths, and DALYs. The GBD standard population and life expectancy were used. Age groups were categorized as follows: <5, 5–9, 10–14, 15–19, 20–24, 25–29, 30–34, 35–39, 40–44, 45–49, 50–54, 55–59, 60–64, 65–69, 70–74, 75–79, 80–84, 85–89, 90–94, and 95+. The age-standardized rates are expressed per 100,000 population. For each estimate, we provided 95% uncertainty intervals (95% UIs), defined as the 25th and 975th percentiles of the 500 ordered draws. The SDI is a metric that measures development levels by combining factors such as per capita income, average education levels for those aged 15 and older, and fertility rates for individuals under 25. It ranges from zero, representing the lowest level of development, to one, representing the highest level^22^. The relationship between the burden of AF/AFL, as measured in age-standardized DALYs rates, and the SDI was analyzed using smoothing splines models^23^. This approach provides flexibility in modeling non-linear relationships over time while minimizing the risk of overfitting. The optimal smoothing parameter is determined through cross-validation, ensuring a balance between model complexity and fit. By doing so, the model generates robust estimates of incidence, prevalence, mortality, and DALYs, enabling an accurate representation of disease trends and their associated uncertainties. In this study, although specific counts and age-standardized rates for the year 2020 were not separately tabulated, data from 2020 were incorporated into the analysis of trends and percent changes. The year 2020 was considered in the calculation of percent changes over the 2019–2021 period. Trends in incidence, prevalence, and attributable burden for 2020 were also visualized in a figure to provide insight into the impacts of the pandemic. Statistical analysis was conducted using R programming software, version 4.2.1, Python (version 3.10.4), and Stata (version 13.1).

## Results

### National epidemiology of AF/AFL

**Fig. 1:**
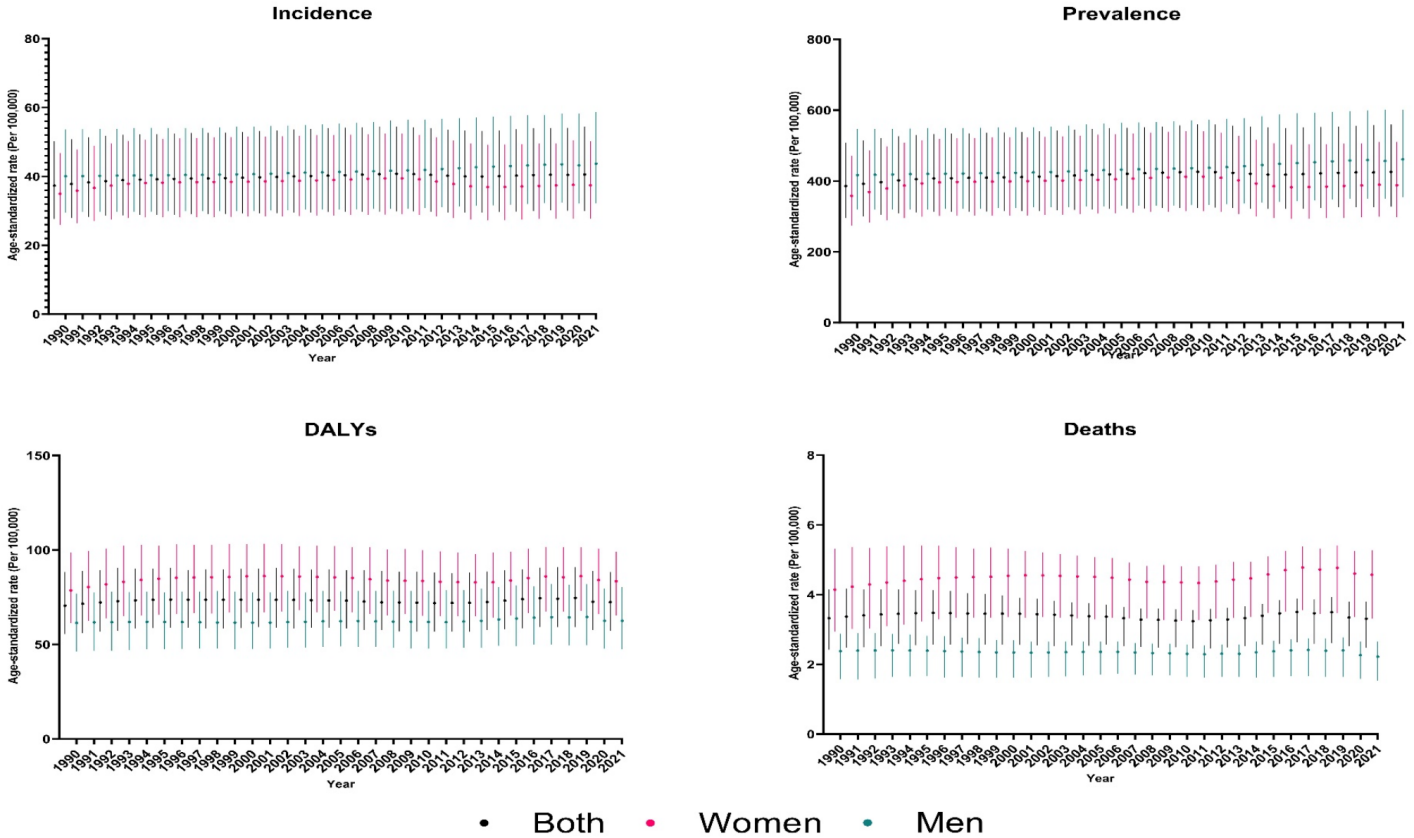
Time trends of age-standardized rates of incidence, prevalence, disability-adjusted life years (DALYs), and deaths of atrial fibrillation and flutter in Iran from 1990 to 2021, by sex.

In Iran, the number of incident cases of AF/AFL in both sexes combined has increased from 8136.4 (95% UI: 6131.5 to 10786.8) in 1990 to 29418.3 (22665.1 to 38723.1) in 2021, with a percentage change of 261.6% (236.1 to 289.9). The AF/AFL ASIR has increased by 8.6% (6.9 to 10.2), from 37.3 (27.6 to 50.2) per 100,000 population in 1990 to 40.6 (30.0 to 54.4) per 100,000 population in 2021. Moreover, the number of prevalent cases of AF/AFL has grown from 75832.5 (58813.8 to 99095.2) in 1990 to 294248.7 (230024.2 to 382165.9) in 2021, with 288.0% (268.9 to 313.2) increase. The AF/AFL age-standardized prevalence rate (ASPR) has increased by 10.2% (8.6 to 11.7), from 386.0 (296.2 to 507.6) per 100,000 population in 1990 to 425.4 (327.2 to 559.2) per 100,000 population in 2021. Furthermore, the number of DALYs attributed to AF/AFL has increased from 12115.7 (9430.1 to 15214.4) in 1990 to 47524.7 (37527.7 to 58270.9) in 2021, with a percentage change of 292.3% (239.7 to 352.3). The age-standardized DALYs rate was 72.4 (57.4 to 88.3) per 100,000 population in 2021, with no significant changes over 1990–2021. Additionally, the number of deaths due to AF/AFL has grown from 410.6 (305.9 to 509.9) in 1990 to 1948.9 (1462.6 to 2233.1) in 2021, with 374.6% (268.7 to 511.3) increase. However, the AF/AFL ASDR showed no significant changes (-0.4% [UI − 22.3 to 28.3]) between 1990 and 2021 (Table 1; Fig. 1, and Table S1).

**Table 1:** All‑ages number and age‑standardized rate of incidence, prevalence, disability-adjusted life years (DALYs), and deaths of atrial fibrillation and flutter by sex in 1990 and 2021 and overall percent change over 1990–2021 in Iran.

Measure	Age, Metric	Year						% Change (1990 to 2021)		
		1990			2021					
		Both	Women	Men	Both	Women	Men	Both	Women	Men
Incidence	Age-standardized	37.3 (27.6 to 50.2)	35 (25.9 to 46.8)	40 (29.6 to 53.7)	40.6 (30 to 54.4)	37.4 (27.7 to 50.2)	43.7 (32.3 to 58.7)	8.6 (6.9 to 10.2)	7 (5.3 to 8.8)	9.2 (6.4 to 11.6)
	All ages	8136.4 (6131.5 to 10786.8)	3690.9 (2775 to 4890.5)	4445.5 (3327.9 to 5896.3)	29418.3 (22665.1 to 38723.1)	13622.3 (10363.4 to 18155.8)	15,796 (12121.5 to 20711)	261.6 (236.1 to 289.9)	269.1 (249.1 to 290.5)	255.3 (223.9 to 291.2)
Prevalence	Age-standardized	386 (296.2 to 507.6)	358.1 (273.8 to 471.7)	416.7 (318.6 to 546.4)	425.4 (327.2 to 559.2)	387.8 (298 to 510.7)	461.1 (354.7 to 601.6)	10.2 (8.6 to 11.7)	8.3 (6.4 to 10.2)	10.7 (8.1 to 12.8)
	All ages	75832.5 (58813.8 to 99095.2)	34230.7 (26107.8 to 44725)	41601.8 (32447.4 to 54379.3)	294248.7 (230024.2 to 382165.9)	134056.9 (103750.2 to 176465.5)	160191.8 (124346.3 to 206830.5)	288 (268.9 to 313.2)	291.6 (274.1 to 311)	285.1 (260.5 to 316.7)
DALYs	Age-standardized	70.5 (55.4 to 88.3)	78.7 (61.2 to 98.7)	61.4 (46.2 to 76.9)	72.4 (57.4 to 88.3)	83.5 (65.3 to 99.2)	62.5 (47.4 to 80.3)	2.6 (− 13.1 to 18.2)	6.1 (− 14.5 to 24.6)	1.8 (− 9.9 to 23)
	All ages	12115.7 (9430.1 to 15214.4)	6569.4 (5120.9 to 8236.8)	5546.3 (4130.2 to 7086.4)	47524.7 (37527.7 to 58270.9)	26310.9 (20508.1 to 31274.3)	21213.7 (16083.8 to 27193.4)	292.3 (239.7 to 352.3)	300.5 (227.6 to 368.7)	282.5 (236.4 to 359.6)
Deaths	Age-standardized	3.3 (2.4 to 4.1)	4.1 (2.9 to 5.3)	2.4 (1.6 to 2.9)	3.3 (2.5 to 3.8)	4.6 (3.3 to 5.3)	2.2 (1.5 to 2.7)	− 0.4 (− 22.3 to 28.3)	10.2 (− 18.8 to 40.3)	− 6.6 (− 24 to 41.4)
	All ages	410.6 (305.9 to 509.9)	266.4 (193.5 to 341.2)	144.2 (97.9 to 176.6)	1948.9 (1462.6 to 2233.1)	1258.4 (920 to 1445.6)	690.6 (478.6 to 823.5)	374.6 (268.7 to 511.3)	372.3 (246.1 to 502.3)	378.7 (281.6 to 618.5)

Rates are per 100,000 population.

Between 2019 and 2021, while the ASIR and ASPR were almost steady, there were noticeable decreases in age-standardized DALYs and death rates in Iran. The age-standardized DALYs rate decreased by 3.0% (− 5.9 to − 0.1), from 74.7 (59.1 to 91.0) per 100,000 population in 2019 to 72.4 (57.4 to 88.3) per 100,000 population in 2021. Moreover, the ASDR decreased by 5.2% (− 9.8 to − 0.5), from 3.5 (2.6 to 3.9) per 100,000 population in 2019 to 3.3 (2.5 to 3.8) per 100,000 population in 2021 (Table S2).

### Provincial epidemiology of AF/AFL

**Fig. 2:**
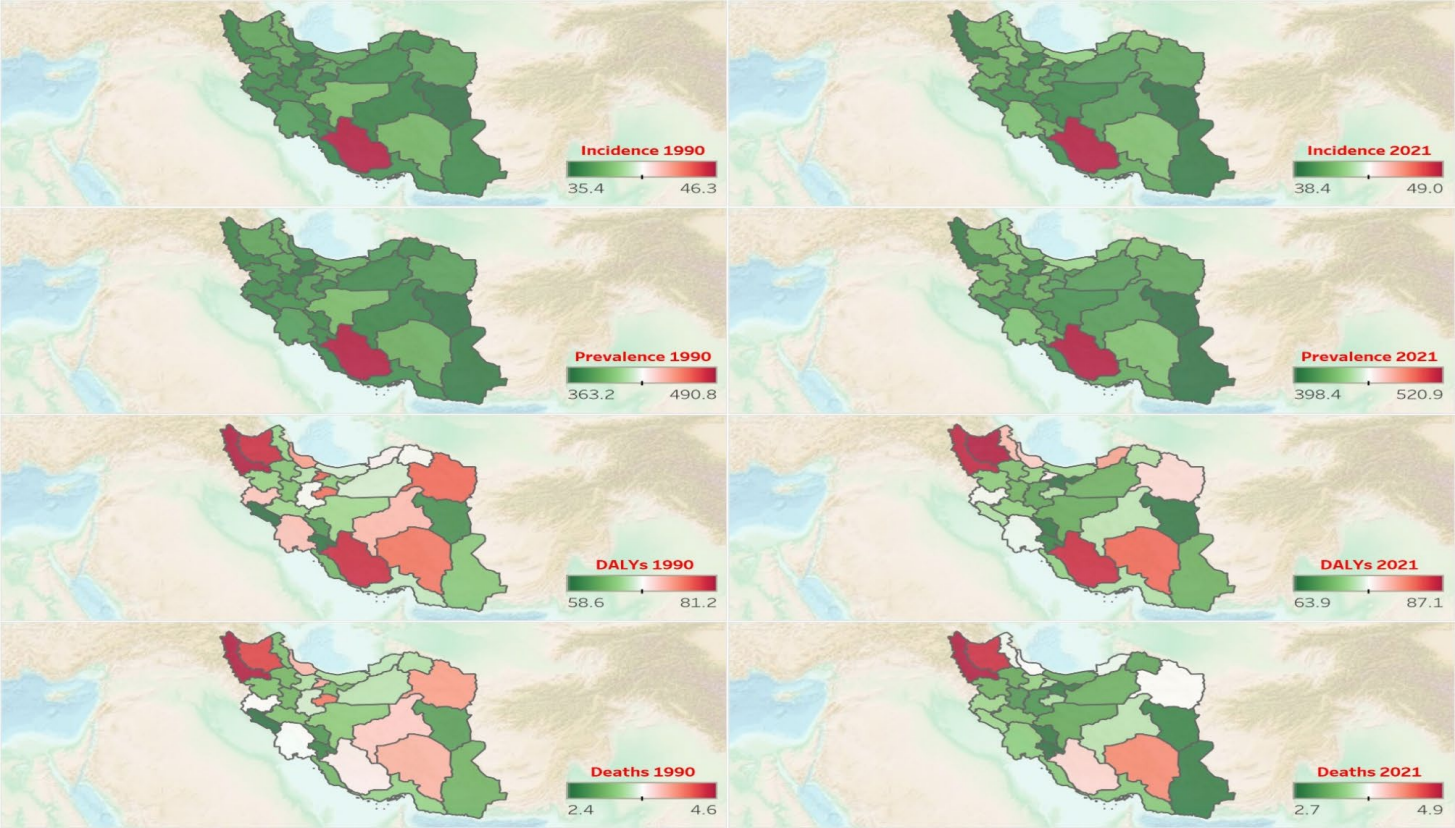
Geographical distribution of age-standardized rates of incidence, prevalence, disability-adjusted life years (DALYs), and deaths of atrial fibrillation and flutter among both sexes in 1990 and 2021 in Iran. The maps were generated using R software (version 4.2.1, available at https://www.r-project.org/). Base map data are reproduced from the GEBCO_2022 Grid, GEBCO Compilation Group (2022), GEBCO 2022 Grid (doi:10.5285/e0f0bb80-ab44-2739-e053-6c86abc0289c). The GEBCO Grid is in the public domain and is used here with acknowledgment in accordance with the GEBCO license terms.

**Fig. 3:**
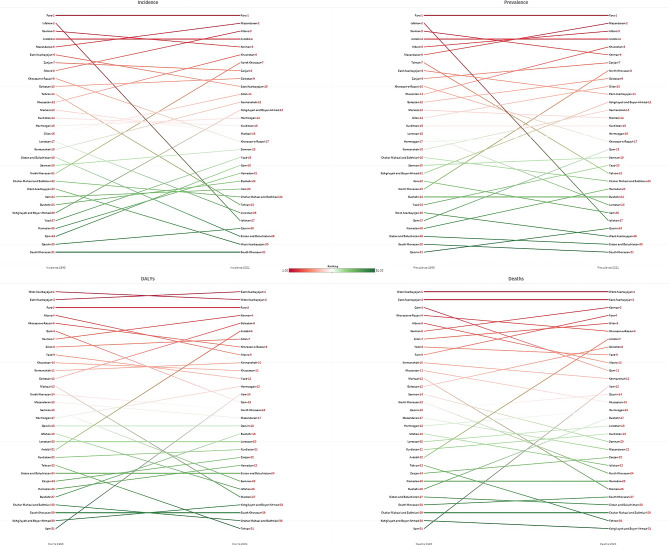
Ranking of the age-standardized rates of incidence, prevalence, disability-adjusted life years (DALYs), and deaths of atrial fibrillation and flutter among both sexes in 1990 and 2021 in Iran.

In 1990, Fars (46.3 [34.1 to 63.0]), Isfahan (37.9 [28.3 to 50.7]), and Kerman (37.8 [27.9 to 50.3]) had the highest ASIRs among both sexes in Iran, while South Khorasan (35.4 [26.1 to 47.5]), Qazvin (35.5 [26.0 to 47.7]), and Qom (35.7 [26.3 to 48.0]) reported the lowest ASIRs. In 2021, Fars (49.0 [36.1 to 66.8]) remained the province with the highest ASIR, followed by Mazandaran (41.8 [31.2 to 56.1]) and Alborz (41.5 [30.7 to 56.2]). On the other hand, South Khorasan (38.4 [28.2 to 51.0]) remained the province with the lowest ASIR, followed by West Azarbayejan (38.5 [28.6 to 51.4]) and Sistan and Baluchistan (38.7 [28.2 to 51.9]). From 1990 to 2021, North Khorasan experienced the highest percentage change in ASIR and ASPR, with increases of 13.5% (8.5 to 19.2) and 15.6% (9.8 to 20.8), respectively (Table S1, Fig. 2, and Fig. 3).

Similar to the ASIR ranking, in 1990, Fars (490.8 [368.3 to 648.4]), Isfahan (393.4 [302.7 to 516.3]), and Kerman (388.0 [296.7 to 510.9]) exhibited the highest ASPR, while Qazvin (363.2 [277.9 to 473.3]), South Khorasan (363.7 [276.0 to 478.8]), and Sistan and Baluchistan (366.8 [282.0 to 484.7]) had the lowest ASPR values among provinces. In 2021, similar to the ASIR pattern, Fars (520.9 [394.7 to 694.4]), Mazandaran (439.5 [335.2 to 578.2]), and Alborz (439.0 [336.3 to 579.4]) ranked top with the highest ASPR values, while South Khorasan (398.4 [303.8 to 520.4]), Sistan and Baluchistan (398.7 [306.5 to 522.2]), and West Azarbayejan (401.8 [309.0 to 526.0]) ranked bottom in ASPR (Table S1, Fig. 2, and Fig. 3).

In 1990, West Azarbayejan (81.2 [59.3 to 99.8]), East Azarbayejan (80.0 [61.3 to 100.4]), and Fars (80.0 [61.5 to 100.9]) experienced the highest age-standardized DALYs rates, while Ilam (58.6 [44.4 to 75.2]), Kohgiluyeh and Boyer-Ahmad (58.8 [44.9 to 77.5]), and South Khorasan (60.7 [46.3 to 77.3]) had the lowest ones. In 2021, the trend slightly changed, with East Azarbayejan (87.1 [69.1 to 106.4]), West Azarbayejan (86.4 [64.7 to 103.9]), and Fars (85.8 [66.9 to 108.1]) bearing the highest age-standardized DALYs rates. In contrast, Tehran (63.9 [49.3 to 79.3]), Chahar Mahaal and Bakhtiari (64.3 [49.2 to 79.9]), and South Khorasan (64.4 [49.9 to 80.4]) had the lowest values. Between 1990 and 2021, Ilam experienced the highest DALY percentage change among provinces, with an increase of 24.1% (2.1 to 55.9) (Table S1, Fig. 2, and Fig. 3).

West Azarbayejan (4.6 [2.8 to 5.8]), East Azarbayejan (4.3 [3.0 to 5.5]), and Qom (4.1 [2.7 to 5.2]) reported the highest ASDRs in 1990. On the other hand, Ilam (2.4 [1.6 to 3.1]), Kohgiluye and Boyer-Ahmad (2.5 [1.6 to 3.4]), and Chahar Mahaal and Bakhtiari (2.6 [1.8 to 3.4]) exhibited the lowest ASDRs in Iran. In 2021, while West Azarbayejan (4.9 [3.3 to 5.9]) and East Azarbayejan (4.7 [3.4 to 5.8]) remained in first and second place with the highest ASDR values, Kerman (4.3 [3.0 to 5.2]) took third place. In contrast, Kohgiluye and Boyer-Ahmad (2.7 [2.0 to 3.3]), Tehran (2.8 [2.0 to 3.4]), and Chahar Mahaal and Bakhtiari (2.8 [2.1 to 3.4]) had the lowest ASDRs in Iran. Ilam had the highest ASDR percent change among Iran’s provinces, with an increase of 45.7% (1.0 to 122.1) over the 1990 to 2021 period (Table S1, Fig. 2, and Fig. 3).

In females, Fars had the highest ASIR, ASPR, and age-standardized DALYs rate among Iran’s provinces in 2021, while West Azarbayejan had the highest ASDR (Figs. S1, S2). In males, Mazandaran had the highest ASIR and ASPR among provinces of Iran in 2021. On the other hand, East and West Azarbayejan had the highest age-standardized DALY and death rates, respectively (Figs. S3, S4).

### Age and sex patterns of AF/AFL in Iran

**Fig. 4:**
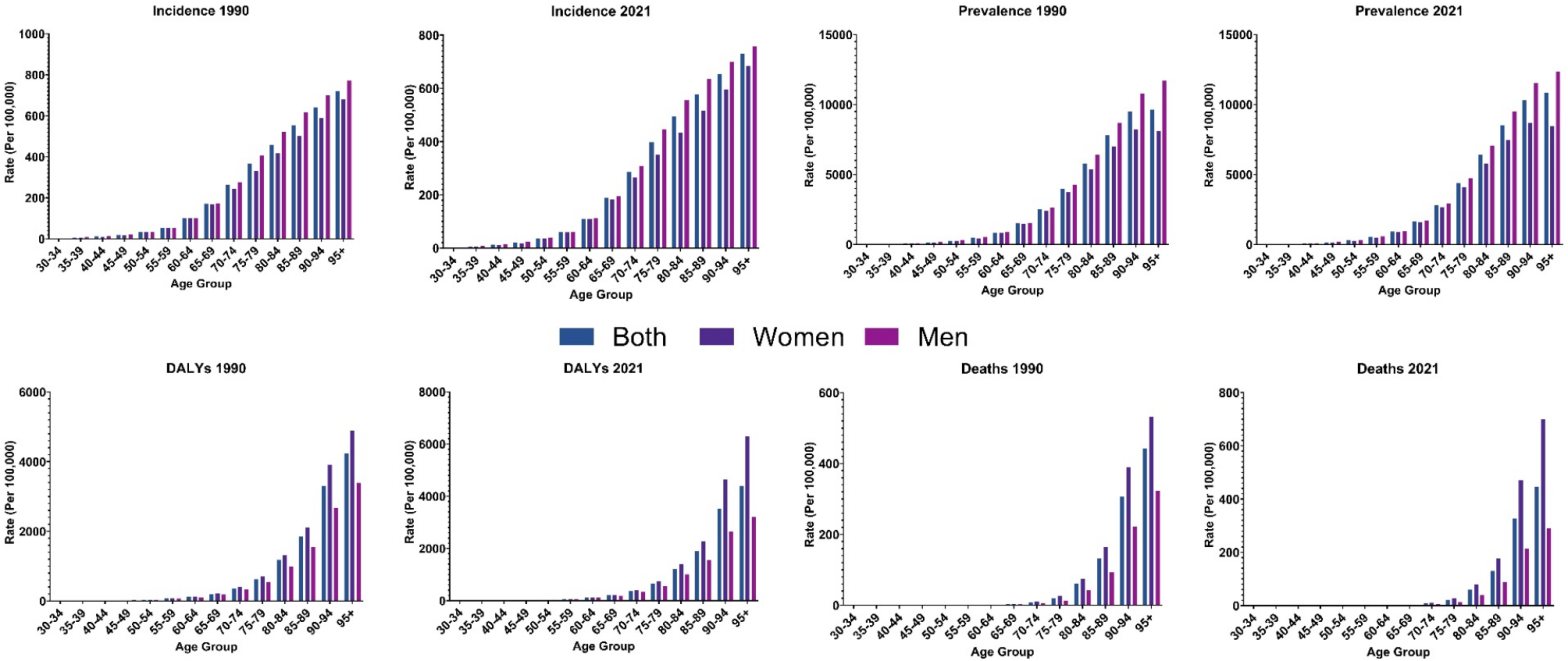
Rates of incidence, prevalence, disability-adjusted life years (DALYs), and deaths of atrial fibrillation and flutter in Iran in 1990 and 2021, by sex and age.

The national incidence, prevalence, DALYs, and death rates of AF/AFL indicated an overall increasing trend with age, peaking at the 95+ age group (Fig. 4). In terms of incidence and prevalence, males experienced higher rates compared to females, while regarding DALYs and deaths, females had higher values than males (Figs. 1 and 4). In the 1990 to 2021 period, the difference between females and males regarding DALYs and death rates increased, leading to an even wider gap in 2021 compared to 1990 (Table 1; Fig. 4).

### The attributable burden of AF/AFL to risk factors

**Fig. 5:**
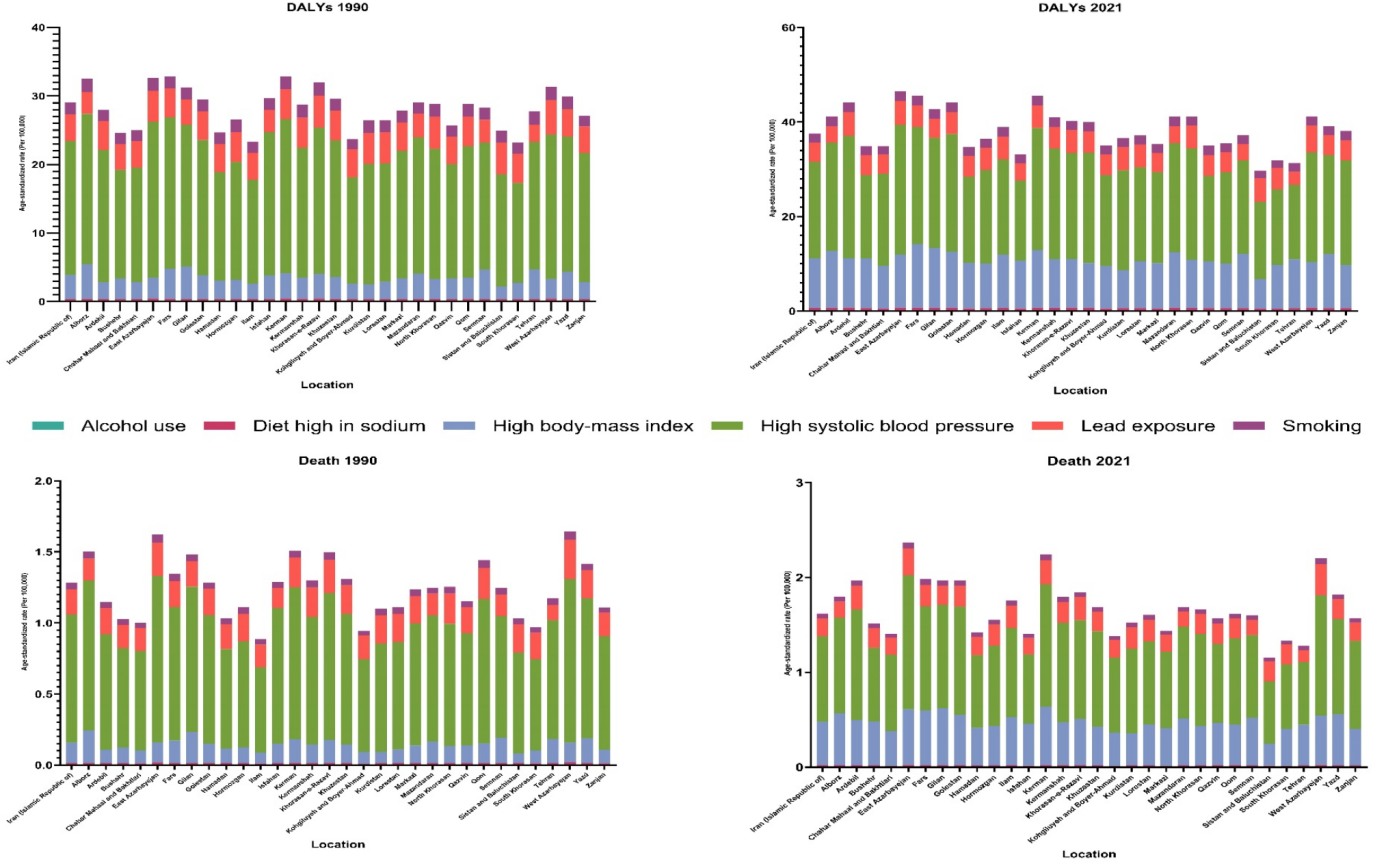
Age-standardized rates of disability-adjusted life years (DALYs) and deaths of atrial fibrillation and flutter attributable to risk factors among both sexes in 1990 and 2021 in Iran.

At the national level, in 2021, the AF/AFL DALYs were attributable to six risk factors, including high SBP (20.5 [6.8 to 34.5]), high BMI (10.5 [4.4 to 18.1]), lead exposure (4.0 [− 0.6 to 10.1]), smoking (1.9 [1.1 to 3.0]), diet high in sodium (0.4 [0.0 to 2.4]), and high alcohol use (0.2 [0.1 to 0.3]). The risk factors that contributed the most to AF/AFL deaths were high SBP (0.9 [0.3 to 1.5]), high BMI (0.5 [0.2 to 0.8]), and lead exposure (0.2 [0.0 to 0.5]) (Table 2; Fig. 5). From 1990 to 2021, as high SBP remained the AF/AFL risk factor with the highest attributable DALYs and deaths, the proportion of DALYs and deaths attributable to high BMI increased significantly over this period (Table 2; Fig. 5). Similarly, high SBP had the highest attributable burden in all provinces and among males and females in Iran (Figs. S5–S12). Table S3 shows the distribution of DALYs and death numbers and rates of AF/AFL attributable to risk factors across Iran and its provinces from 1990 to 2021.

**Table 2:** All‑ages number and age‑standardized rate of disability-adjusted life years (DALYs) and deaths of atrial fibrillation and flutter attributable to risk factors by sex in 1990 and 2021 and overall percent change over 1990–2021 in Iran.

Risk factor	Measure	Age, Metric	Year						% Change (1990 to 2021)		
			1990			2021					
			Both	Women	Men	Both	Women	Men	Both	Women	Men
All risk factors	DALYs	Age-standardized	22.6 (10.1 to 35.6)	25.9 (11.5 to 41.7)	18.9 (8.1 to 29.8)	27.4 (14.7 to 40.8)	32.2 (16.8 to 49.5)	23 (12.1 to 35.3)	21 (− 2.1 to 61.4)	24.1 (− 6.6 to 66.3)	21.9 (2.9 to 69.3)
		All ages	4046.2 (1834.1 to 6377.4)	2210.8 (993.9 to 3499.2)	1835.4 (827.8 to 2919.4)	18252.5 (9774.3 to 27579.4)	10305.9 (5381.3 to 15540.2)	7946.6 (4210.4 to 12110.5)	351.1 (274.3 to 481.3)	366.2 (259.2 to 510.8)	333 (268.3 to 470.6)
	Deaths	Age-standardized	1 (0.4 to 1.7)	1.3 (0.6 to 2.2)	0.7 (0.2 to 1.1)	1.2 (0.6 to 1.8)	1.7 (0.8 to 2.6)	0.7 (0.4 to 1.1)	17 (− 13.8 to 71.4)	27.6 (− 12.7 to 85.1)	13.1 (− 12.7 to 97.3)
		All ages	129 (56.3 to 209.6)	86.1 (38.5 to 144.7)	42.9 (16.5 to 70.5)	704.2 (368 to 1079)	468.9 (228.4 to 722)	235.3 (118.8 to 358.2)	445.9 (303.9 to 686)	444.8 (277.8 to 675.6)	448.1 (316.8 to 818.7)
Diet high in sodium	DALYs	Age-standardized	0.4 (0 to 2.3)	0.3 (0 to 2)	0.5 (0 to 2.6)	0.4 (0 to 2.4)	0.3 (0 to 2.1)	0.5 (0 to 2.7)	0.7 (− 86.9 to 356)	0.2 (− 99.5 to 2615.1)	1 (− 86.2 to 374.9)
		All ages	78 (0 to 443.5)	25.8 (0 to 190)	52.2 (0 to 263.7)	264.2 (0 to 1594.9)	91.1 (0 to 690.3)	173.1 (0 to 940.5)	238.6 (− 55 to 1036.4)	252.9 (− 99.8 to 4560.2)	231.5 (− 57.4 to 1037.7)
	Deaths	Age-standardized	0 (0 to 0.1)	0 (0 to 0.1)	0 (0 to 0.1)	0 (0 to 0.1)	0 (0 to 0.1)	0 (0 to 0.1)	− 1.6 (− 88.4 to 381.9)	3.6 (− 100 to 3113)	− 6.9 (− 88.7 to 425.1)
		All ages	2 (0 to 12.3)	0.9 (0 to 6.9)	1.1 (0 to 5.8)	8.1 (0 to 56.5)	3.6 (0 to 29.6)	4.5 (0 to 27.8)	308.9 (− 60.8 to 1189.9)	311.7 (− 99.3 to 7053.5)	306.8 (− 61.7 to 1249.8)
High alcohol use	DALYs	Age-standardized	0 (0 to 0)	0 (0 to 0)	0 (0 to 0)	0.2 (0.1 to 0.3)	0 (0 to 0.1)	0.3 (0.2 to 0.5)	NA	NA	NA
		All ages	0 (0 to 0)	0 (0 to 0)	0 (0 to 0)	141 (87.4 to 211.6)	11.5 (5.7 to 20)	129.5 (80.8 to 195.2)	NA	NA	NA
	Deaths	Age-standardized	0 (0 to 0)	0 (0 to 0)	0 (0 to 0)	0 (0 to 0)	0 (0 to 0)	0 (0 to 0)	NA	NA	NA
		All ages	0 (0 to 0)	0 (0 to 0)	0 (0 to 0)	3.2 (1.8 to 4.9)	0.4 (0.2 to 0.8)	2.8 (1.5 to 4.2)	NA	NA	NA
High body-mass index	DALYs	Age-standardized	3.5 (1.4 to 6)	5.4 (2.1 to 9.3)	1.5 (0.6 to 2.9)	10.5 (4.4 to 18.1)	13.9 (5.9 to 23.9)	7.4 (2.9 to 12.8)	200.7 (80.4 to 354.7)	157.8 (37.1 to 299.2)	375.2 (198.8 to 904.3)
		All ages	706.5 (276.7 to 1173.3)	523.9 (210.9 to 881.9)	182.7 (69.5 to 329.9)	7061.4 (2941.6 to 12080.8)	4520.5 (1937.9 to 7735.9)	2540.9 (1001.6 to 4465.5)	899.4 (537 to 1280.2)	762.9 (410.3 to 1114.4)	1291 (800.1 to 2445.4)
	Deaths	Age-standardized	0.1 (0.1 to 0.3)	0.2 (0.1 to 0.4)	0 (0 to 0.1)	0.5 (0.2 to 0.8)	0.7 (0.3 to 1.2)	0.3 (0.1 to 0.4)	219.6 (66.3 to 494.4)	198 (39.3 to 468.7)	476.7 (198.5 to 1714)
		All ages	20.4 (8.1 to 35.2)	17 (6.7 to 31.1)	3.4 (1.1 to 6.5)	273.6 (115.9 to 484.6)	195.3 (81.9 to 340.9)	78.3 (30.9 to 134.5)	1241.6 (654.1 to 2101.8)	1048.8 (479.9 to 1853.4)	2208.2 (1226.6 to 5379.2)
High systolic blood pressure	DALYs	Age-standardized	19.6 (6.5 to 32.9)	22.8 (7.7 to 38.5)	15.9 (5 to 27.4)	20.5 (6.8 to 34.5)	24.5 (8.2 to 41.7)	16.8 (5.6 to 29.4)	4.7 (− 12.1 to 23.1)	7.7 (− 14.5 to 29.8)	5.2 (− 8.9 to 28.8)
		All ages	3370.5 (1113.1 to 5682)	1896.4 (641.9 to 3199.7)	1474.1 (458.5 to 2552.3)	13514.7 (4484.7 to 22734.4)	7813.4 (2582.7 to 13294.2)	5701.2 (1888.2 to 9843.1)	301 (241.1 to 367.9)	312 (229.8 to 391)	286.8 (237.6 to 372.6)
	Deaths	Age-standardized	0.9 (0.3 to 1.6)	1.2 (0.4 to 2.1)	0.6 (0.2 to 1.1)	0.9 (0.3 to 1.5)	1.3 (0.5 to 2.2)	0.6 (0.2 to 1)	− 0.1 (− 24.1 to 31.3)	9.6 (− 21.5 to 44.1)	− 4.7 (− 24.6 to 45.7)
		All ages	113.1 (38.1 to 193.5)	76.3 (26.6 to 132.5)	36.8 (10.9 to 64.8)	537.5 (189.4 to 916.7)	362.9 (126.4 to 617.3)	174.6 (59.6 to 306.2)	375.3 (260.9 to 520.2)	375.5 (240.1 to 524.6)	375.1 (268.3 to 616.1)
Lead exposure	DALYs	Age-standardized	3.8 (− 0.5 to 9.8)	4 (− 0.6 to 10.4)	3.6 (− 0.5 to 8.9)	4 (− 0.6 to 10.1)	4.3 (− 0.6 to 10.8)	3.8 (− 0.5 to 9.4)	5.2 (− 11.5 to 26.2)	7.1 (− 16.6 to 32.9)	6.1 (− 8.6 to 32.9)
		All ages	674.1 (− 90.6 to 1695.5)	340.4 (− 47.8 to 873.6)	333.7 (− 44.5 to 834.4)	2604.4 (− 350.4 to 6540)	1330.8 (− 180.3 to 3297.7)	1273.6 (− 170.1 to 3156.2)	286.4 (226.7 to 362.4)	291 (209.4 to 380)	281.7 (229 to 374)
	Deaths	Age-standardized	0.2 (0 to 0.4)	0.2 (0 to 0.5)	0.1 (0 to 0.3)	0.2 (0 to 0.5)	0.3 (0 to 0.6)	0.1 (0 to 0.3)	8.8 (− 16.3 to 43.7)	18.8 (− 15.7 to 58.9)	3.8 (− 17 to 63.7)
		All ages	22.1 (− 3.4 to 55.4)	13.6 (− 2.2 to 34.9)	8.4 (− 1.3 to 21.5)	111.9 (− 16.7 to 273.9)	68.2 (− 10 to 166.1)	43.7 (− 7 to 107.2)	406.6 (290.4 to 572.4)	400 (254 to 561.7)	417.4 (305.5 to 708.9)
Smoking	DALYs	Age-standardized	1.8 (1 to 2.8)	0.6 (0.3 to 1)	3 (1.7 to 4.6)	1.9 (1.1 to 3)	0.6 (0.3 to 1)	3.2 (1.8 to 5.1)	8.3 (− 10.9 to 30.9)	3.9 (− 30.7 to 52.8)	9.5 (− 10.1 to 36.3)
		All ages	444 (253.9 to 692.2)	72.6 (39.8 to 113.1)	371.4 (210.7 to 580.6)	1487.3 (822.5 to 2315.4)	241.9 (126.5 to 399.2)	1245.3 (688.9 to 1930.4)	234.9 (178 to 298.5)	233.3 (131.3 to 373.7)	235.3 (176 to 312.8)
	Deaths	Age-standardized	0 (0 to 0.1)	0 (0 to 0)	0.1 (0 to 0.1)	0 (0 to 0.1)	0 (0 to 0)	0.1 (0 to 0.1)	7.1 (− 23.7 to 63.5)	6 (− 44 to 97.8)	1.5 (− 29.4 to 64.6)
		All ages	7.9 (4.3 to 11.7)	1.8 (0.9 to 2.8)	6.1 (3.2 to 9.5)	31.7 (17 to 49.7)	6.8 (3.4 to 12.1)	24.9 (13 to 39.6)	301 (186.6 to 497.3)	285 (121.4 to 564.9)	305.5 (174.2 to 542.3)

Rates are per 100,000 population.

### The association between SDI and AF/AFL burden

**Fig. 6:**
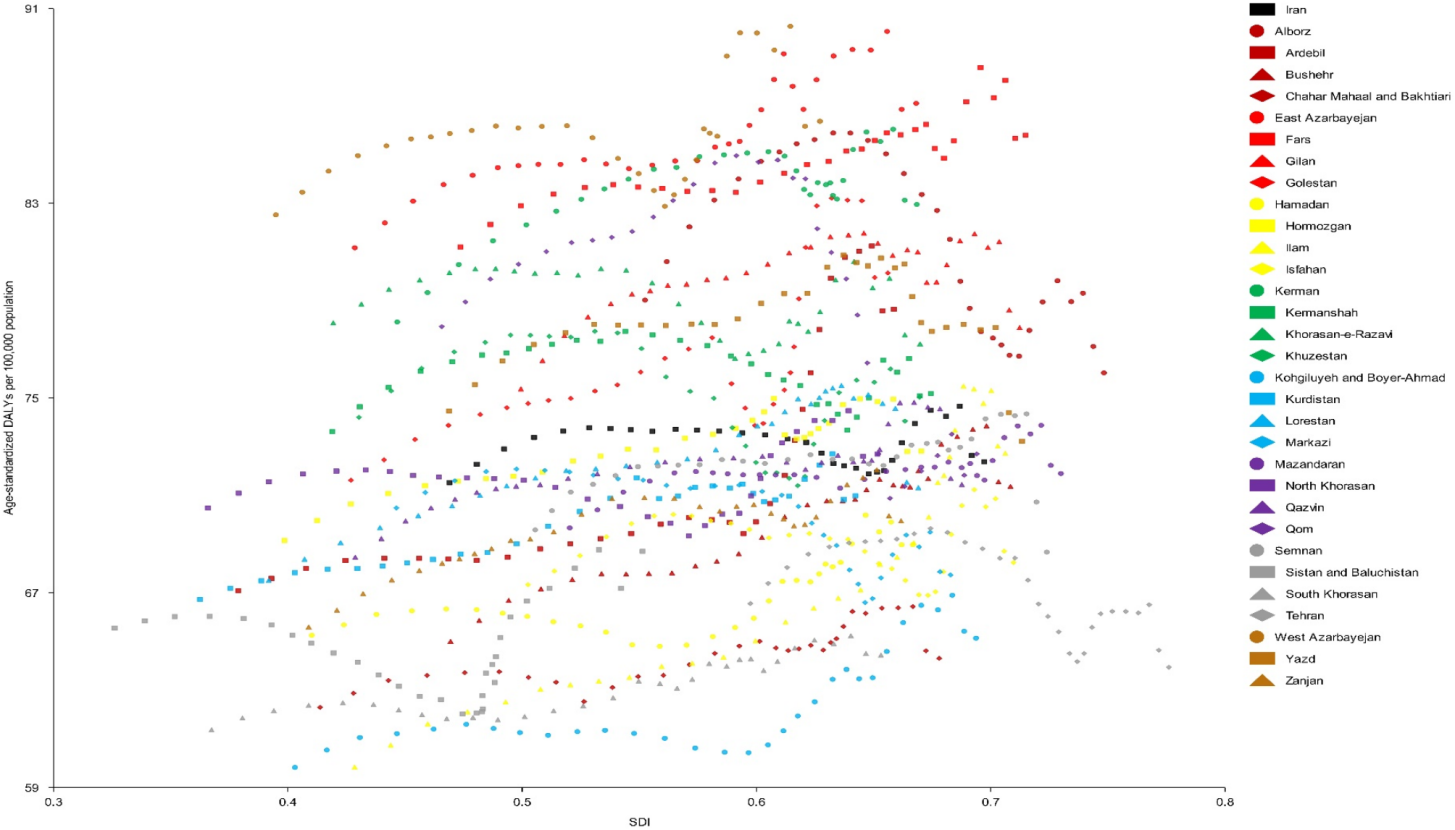
Age-standardized DALY rates of atrial fibrillation and flutter for the 31 Iran provinces from 1990 to 2021, by SDI; Expected values based on the SDI and disease rates in all locations are shown as the black rectangle. Each point shows the observed age-standardized DALYs rate for each province from 1990 to 2021. DALY: disability-adjusted life year. *SDI* socio-demographic index.

While there were fluctuations between SDI and DALYs rates over 1990 to 2021, SDI appeared to have a neutral association with age-standardized DALYs rates of AF/AFL in Iran. Provinces such as East and West Azarbayejan and Fars had higher than expected AF/AFL burden. In contrast, Kohgiluyeh and Boyer-Ahmad, South Khorasan, and Chahar Mahaal and Bakhtiari had lower than expected AF/AFL burden in Iran (Fig. 6).

## Discussion

The current analysis of the GBD 2021 data thoroughly investigated the epidemiology and burden metrics of AF/AFL and its attributable risk factors in Iran at the national and subnational levels over 32 years, considering the effects of COVID-19 by reporting the epidemiology between 2019 and 2021. The findings revealed a significant increase in the ASIR and ASPR of AF/AFL from 1990 to 2021 in Iran, while the age-standardized rates of death and DALY decreased over the 2019–2021 period. Additionally, males experienced higher ASIR and ASPR of AF/AFL than females, while females had higher age-standardized DALYs rate and ASDR. The burden of AF/AFL increased with age, and high SBP was identified as the most significant risk factor contributing to DALYs and deaths from AF/AFL.

One of the primary findings of this study showed a significant increase in the AF/AFL ASIR and ASPR from 1990 to 2021 in Iran. In contrast, a previous GBD analysis by Soleimani et al. reported different results^18^. They investigated the epidemiology and burden of AF/AFL in Iran from 1990 to 2019 using the GBD 2019 data and found no significant changes in ASIR and ASPR of AF/AFL from 1990 to 2019^18^. The elevated ASIR and ASPR of AF/AFL in Iran from 1990 to 2021 could be attributed to several factors. Iran has experienced a significant demographic shift, with an increasing proportion of older adults^24^. As demonstrated in the Framingham Heart Study, a cohort study with 9511 participants and a 50-year study period, age is a critical risk factor for AF and the risk of developing AF escalates with age^25^. Thus, the increase in the elderly population directly correlates with higher incidence and prevalence rates of AF/AFL. Moreover, there has been a notable rise in comorbid conditions such as hypertension, diabetes, and obesity in Iran^26,27^. These conditions are well-established risk factors for AF/AFL^2,10^. For instance, a recent prospective survey from the Iranian Registry of Atrial Fibrillation on 1300 individuals found that 52% of patients with AF had hypertension^28^. As these comorbidities become more prevalent, they contribute to the increasing incidence and prevalence of AF/AFL. Furthermore, shifts in lifestyle factors, including increased sedentary behavior, poor dietary habits (e.g., high intakes of calories, fat, sodium, and sugar), and smoking, may also play a role by leading to conditions that predispose individuals to AF/AFL^29,30^. Additionally, advancements in healthcare infrastructure and increased awareness of AF/AFL may have led to better detection and reporting of AF/AFL.

The study found no significant changes in the age-standardized DALY and death rates of AF/AFL in Iran from 1990 to 2021. These findings were in line with results from the previous GBD investigation in Iran, which reported no significant changes in the age-standardized DALYs rate and ASDR for AF/AFL, with values of 0% (95% UI: − 10.4 to 19.1) and 1.9% (95% UI: − 18.7 to 51.5), respectively^18^. Although the burden of AF/AFL, as reflected in age-standardized DALYs and death rates, has remained relatively constant from 1990 to 2021, this should not lead to complacency. The observed trends likely reflect advancements in medical management, early diagnosis, and improvements in education over the past three decades. However, the rising incidence and prevalence of AF/AFL indicate a growing public health challenge. It is crucial to prioritize and promote preventive measures, such as the management of hypertension and other modifiable risk factors, to mitigate the future burden of AF/AFL. Proactive intervention is essential to address the growing number of individuals living with AF/AFL and to reduce associated complications.

It is worth mentioning that the age-standardized DALYs rate and ASDR for AF/AFL decreased significantly between 2019 and 2021. Given that the mentioned period coincides with the COVID-19 pandemic, several explanations could be hypothesized regarding this finding. During the pandemic, there might be cause of death misclassification, where some deaths from other causes were inaccurately classified as COVID-related, leading to an underestimation of deaths specially attributed to AF/AFL during this time^31,32^. Additionally, the focus on COVID-related data collection may have resulted in less rigorous tracking of other diseases, including AF/AFL, affecting the reported statistics for age-standardized DALYs rate and ASDR. However, further investigation is needed to identify the primary contributing factors.

Our study found higher AF/AFL ASIR and ASPR in males compared to females in 2021 in Iran, consistent with the GBD 2021 of AF/AFL on the global scale^10^. However, the prior GBD 2019 studies in Iran and the MENA region found incompatible results, indicating higher ASIR and ASPR of AF/AFL in females^2,18^. The differences in AF/AFL ASIR and ASPR between males and females could be attributed to biological, lifestyle, and sociocultural factors. Research indicated that estrogen may have a protective role in AF by extending the period of effective atrial inactivity, which may account for the increase in the incidence of AF/AFL in females following menopause^33,34^. Moreover, males generally experience a higher prevalence of AF/AFL risk factors, including hypertension, smoking, and alcohol consumption, which may lead to higher ASIR and ASPR in males^35–37^.

Another finding of this study was higher age-standardized DALYs and ASDR of AF/AFL in females compared to males, which was in line with results reported by the GBD 2019 investigations in Iran and the MENA region^2,18^. Conversely, the global GBD 2021 on AF/AFL found inconsistent results, reporting higher AF/AFL age-standardized DALYs and ASDR in males^10^. The Euro Heart Survey of AF comprising 5333 patients with AF found that females presented with more symptoms, such as difficulty breathing and chest pain, and had lower quality of life scores^38^. Additionally, it has been suggested that female patients with arrhythmia are less likely to utilize rhythm control strategies, including electrical cardioversion, catheter ablation, or surgical ablation, which may lead to higher morbidity and mortality^34,39^. These findings suggest that healthcare systems may need to prioritize more resources for the prevention, early detection, and management of AF/AFL in females, which could include targeted public health campaigns and improving access to specialized care. For instance, it could be beneficial to raise awareness regarding the higher risk of AF/AFL in postmenopausal females or promote lifestyle modifications such as maintaining a healthy weight and engaging in physical activity to mitigate risk factors like obesity, which is more prevalent among females^40^.

Consistent with previous GBD investigations on AF/AFL^2,10^, our study demonstrated that the incidence, prevalence, DALYs, and death rates of AF/AFL presented an overall increasing trend with aging. Age-related changes such as oxidative stress, mitochondrial dysfunction, cardiomyocyte hypertrophy, and ion channel inactivation are associated with atrial structural and electrical remodeling, leading to an elevated risk of AF^41,42^. This indicates that the current approach to managing AF/AFL in older individuals needs to be revised, and more efficient strategies and tailored screening programs are required.

As shown in this study, the main risk factor contributing to DALYs and deaths from AF/AFL was high SBP, in line with prior GBD analyses of AF/AFL^2,10^. It has been suggested that an increase of 10 mmHg in blood pressure raises the risk of AF by 19%^43^, while adequate blood pressure management significantly reduces the risk of AF/AFL^44,45^. Our findings highlight the crucial importance of effective hypertension control in patients with AF/AFL, along with adopting a healthy lifestyle, such as a low-salt diet, to reduce morbidity and mortality in this susceptible population.

It is noteworthy that, similar to an earlier study^18^, we did not find a significant association between SDI and the burden of AF/AFL in Iran. The differences regarding the association between SDI and burden of the underlying risk factors might partly explain this. Accordingly, there was a negative association between SDI and age-standardized DALY rates of high SBP over 1990–2019^26^. However, SDI was positively associated with age-standardized DALY rate of high excess body weight^27^. Further studies are required to explore the possible relationship and explain the underlying mechanisms completely.

### Strengths and limitations

The main strength of the study is its comprehensive analysis, which utilized the latest data from the GBD dataset. It offered a robust estimation of the evolving epidemiology and burden of AF/AFL and its associated risk factors in Iran and its provinces from 1990 to 2021, stratified by sex, age, and SDI quintiles. In addition, the effects of the COVID-19 pandemic on the epidemiology of these conditions were investigated. On the other hand, several limitations should be noted. First, although ICD codes are widely recognized for identifying AF/AFL cases, some inaccuracies may persist despite the considerable efforts made in the GBD 2021 study to enhance data quality. Such inaccuracies could affect the precision of the reported findings. Additionally, despite diagnostic advancements regarding the AF/AFL diagnosis, a significant proportion of asymptomatic or paroxysmal cases likely remain undiagnosed, which could result in an underestimation of the actual burden of AF/AFL in the population. Another limitation is that the GBD database does not account for the different subtypes of AF/AFL, such as paroxysmal, persistent, and permanent forms. This lack of distinction constrains our ability to explore variations in the disease burden across these subtypes, which may limit the granularity of our findings. Furthermore, while our analysis included primary risk factors associated with AF/AFL, other potentially influential risk factors, such as diabetes, physical inactivity, and hyperthyroidism, were not incorporated into the GBD study. Finally, data sparsity, especially in low- and middle-income countries is one of the main limitations of GBD studies.

## Conclusions

Our analysis showed the changing trends of AF/AFL burden in Iran from 1990 to 2021 across sex, age, and SDI quintiles. The notable rise in the incidence and prevalence of AF/AFL in Iran underscores its importance as a serious public health concern. Since the AF/AFL is preventable and treatable, it is essential to promptly implement cost-effective strategies that target modifiable risk factors. Future research should focus on the AF/AFL burden of risk factors such as diabetes, hyperthyroidism, and physical inactivity, as well as different subtypes of AF/AFL.

## Electronic supplementary material

Below is the link to the electronic supplementary material.

Supplementary Material 1

## Data Availability

The data used for these analyses are all publicly available at https://vizhub.healthdata.org/gbd-results/.
