# Supplementary material for: Three decades of atrial fibrillation and flutter epidemiology and risk factors in Iran with a focus on the impact of COVID-19
Source: Sci Rep. 2025 Mar 20;15:9697. doi: 10.1038/s41598-025-91737-y (PMC11926214; doi:10.1038/s41598-025-91737-y)
Supplement: Supplementary file 1 — Supplementary Material 1 [file 41598_2025_91737_MOESM1_ESM.docx]

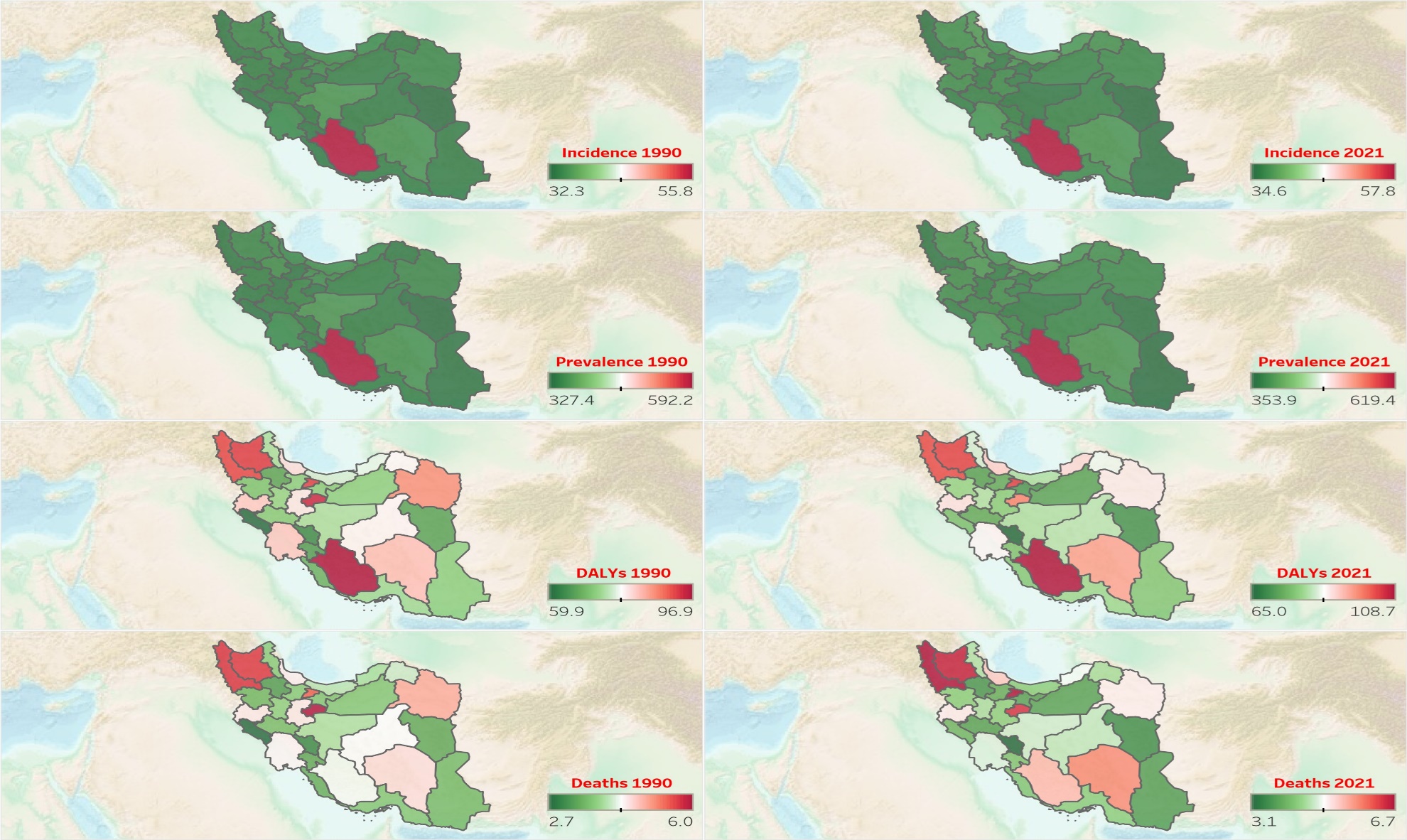


**Figure S1.** Geographical distribution of age-standardized rate of incidence, prevalence, disability-adjusted life years (DALYs), and deaths of atrial fibrillation and flutter among women in 1990 and 2021 in Iran. The maps were generated using R software (version 4.2.1, available at https://www.r-project.org/). Base map data are reproduced from the GEBCO_2022 Grid, GEBCO Compilation Group (2022), GEBCO 2022 Grid (doi:10.5285/e0f0bb80-ab44-2739-e053-6c86abc0289c). The GEBCO Grid is in the public domain and is used here with acknowledgment in accordance with the GEBCO license terms.


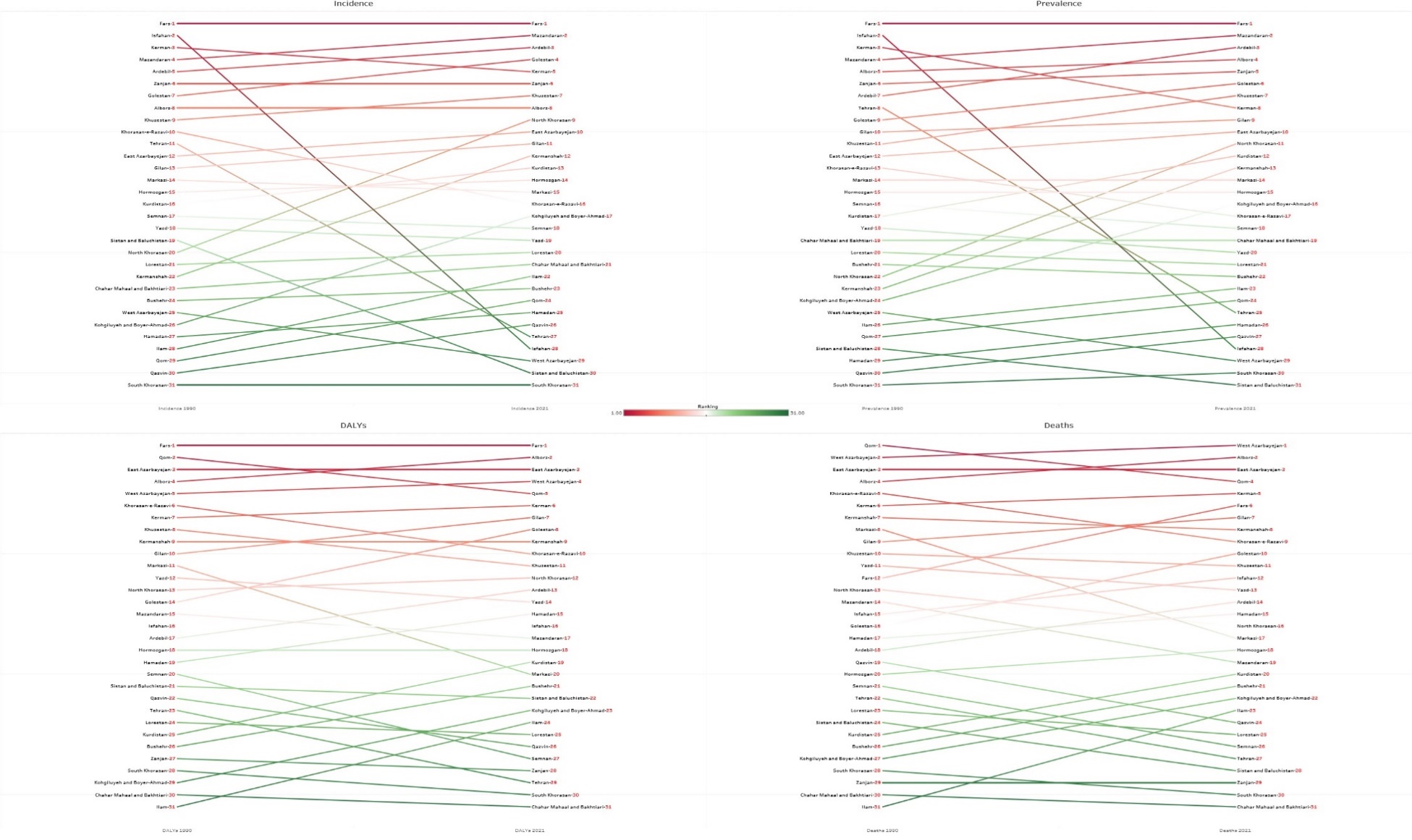
 **Figure S2.** Ranking of the age-standardized rate of incidence, prevalence, disability-adjusted life years (DALYs), and deaths of atrial fibrillation and flutter among women in 1990 and 2021 in Iran


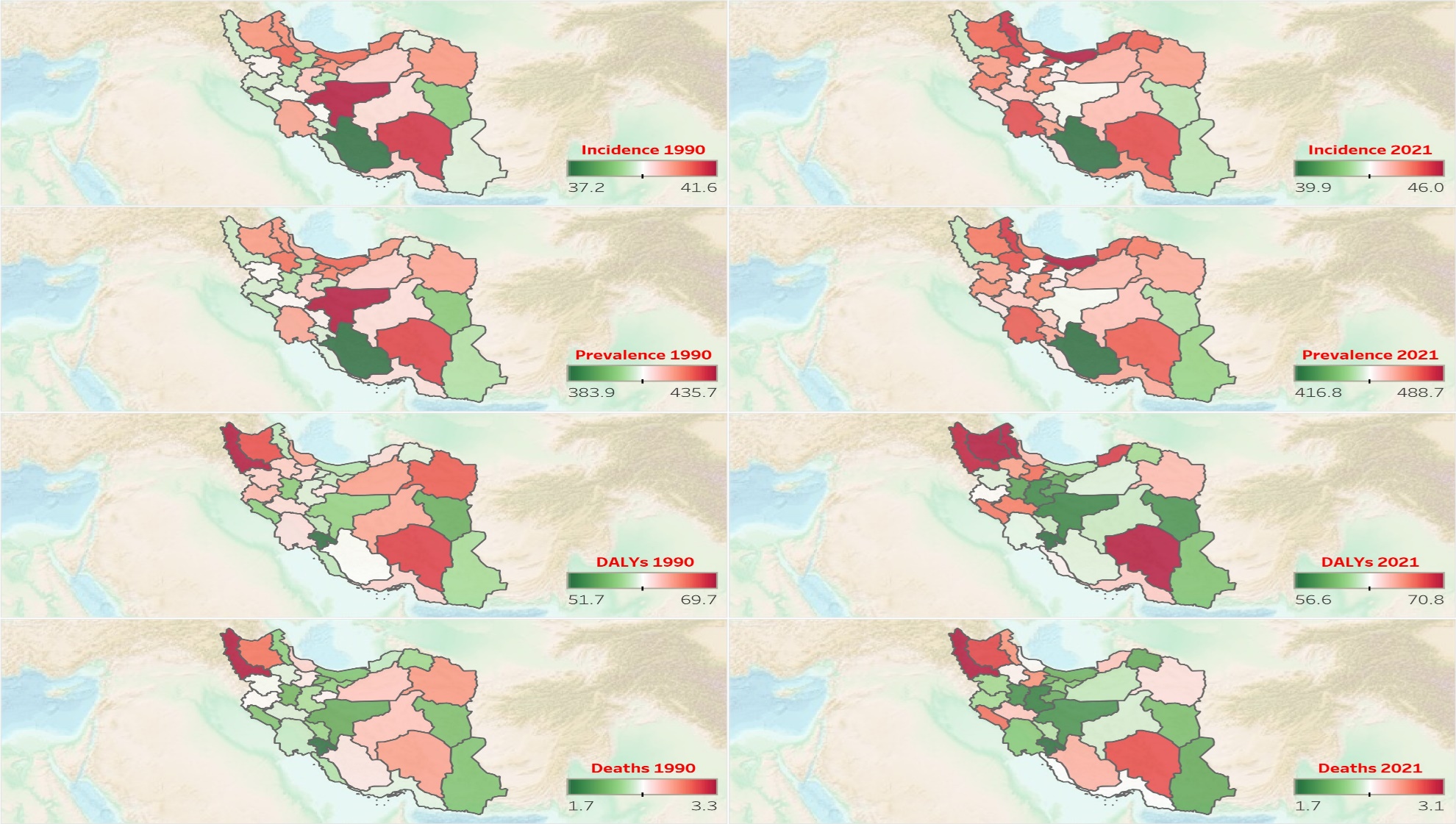
 **Figure S3.** Geographical distribution of age-standardized rate of incidence, prevalence, disability-adjusted life years (DALYs), and deaths of atrial fibrillation and flutter among men in 1990 and 2021 in Iran. The maps were generated using R software (version 4.2.1, available at https://www.r-project.org/). Base map data are reproduced from the GEBCO_2022 Grid, GEBCO Compilation Group (2022), GEBCO 2022 Grid (doi:10.5285/e0f0bb80-ab44-2739-e053-6c86abc0289c). The GEBCO Grid is in the public domain and is used here with acknowledgment in accordance with the GEBCO license terms.


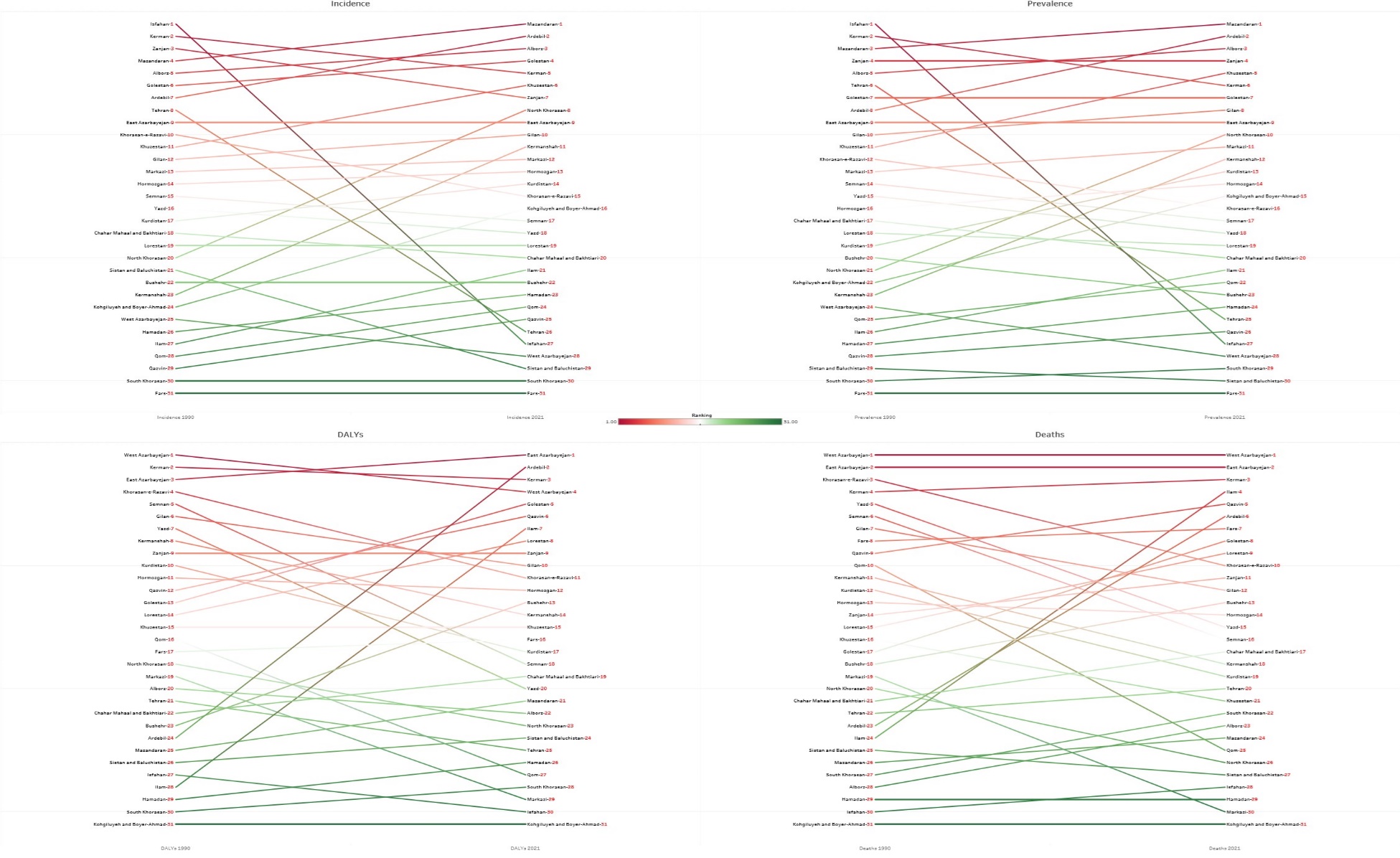
 **Figure S4.** Ranking of the age-standardized rate of incidence, prevalence, disability-adjusted life years (DALYs), and deaths of atrial fibrillation and flutter among men in 1990 and 2021 in Iran


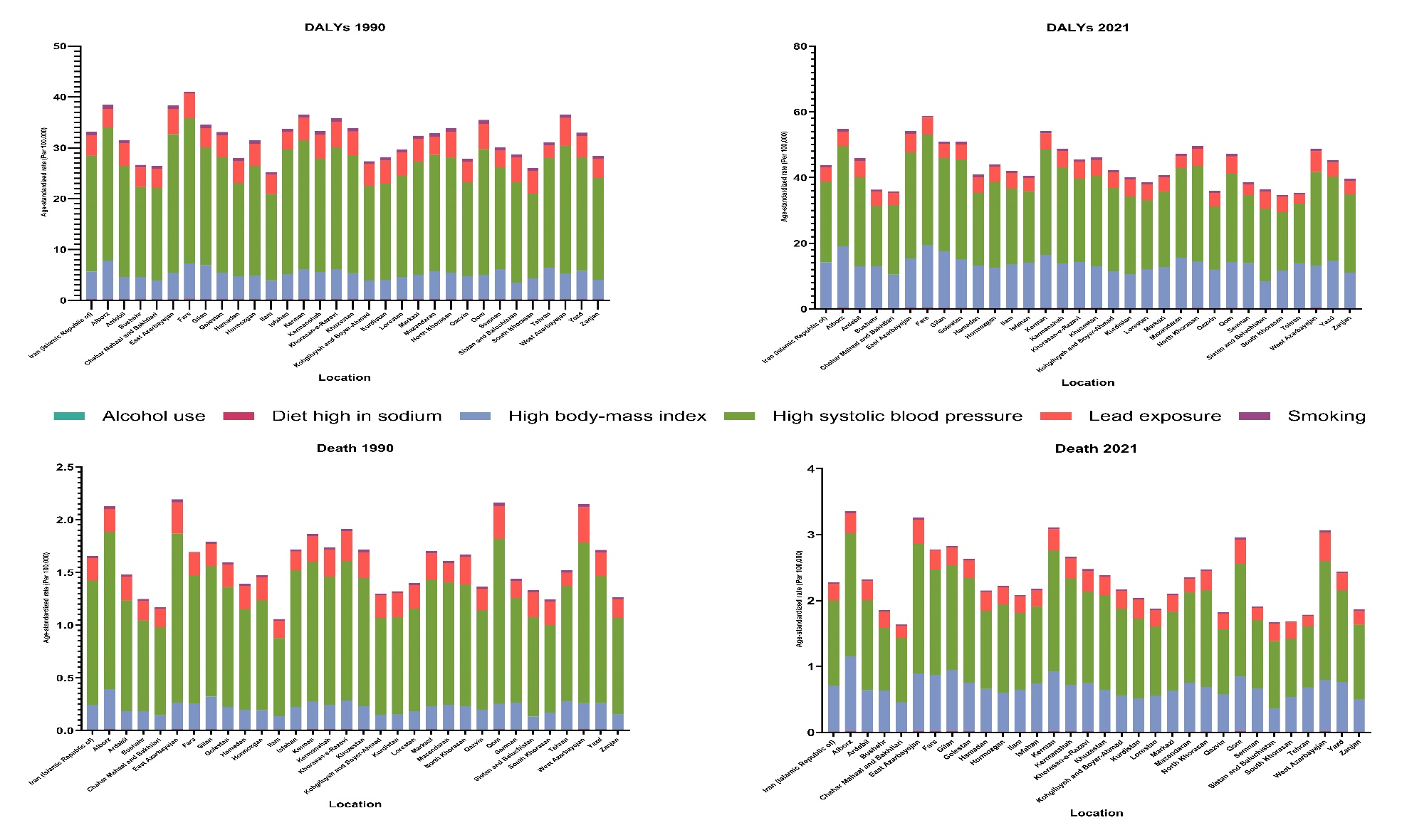
 **Figure S5.** Age-standardized rate of disability-adjusted life years (DALYs) and deaths of atrial fibrillation and flutter attributable to risk factors among women in 1990 and 2021 in Iran


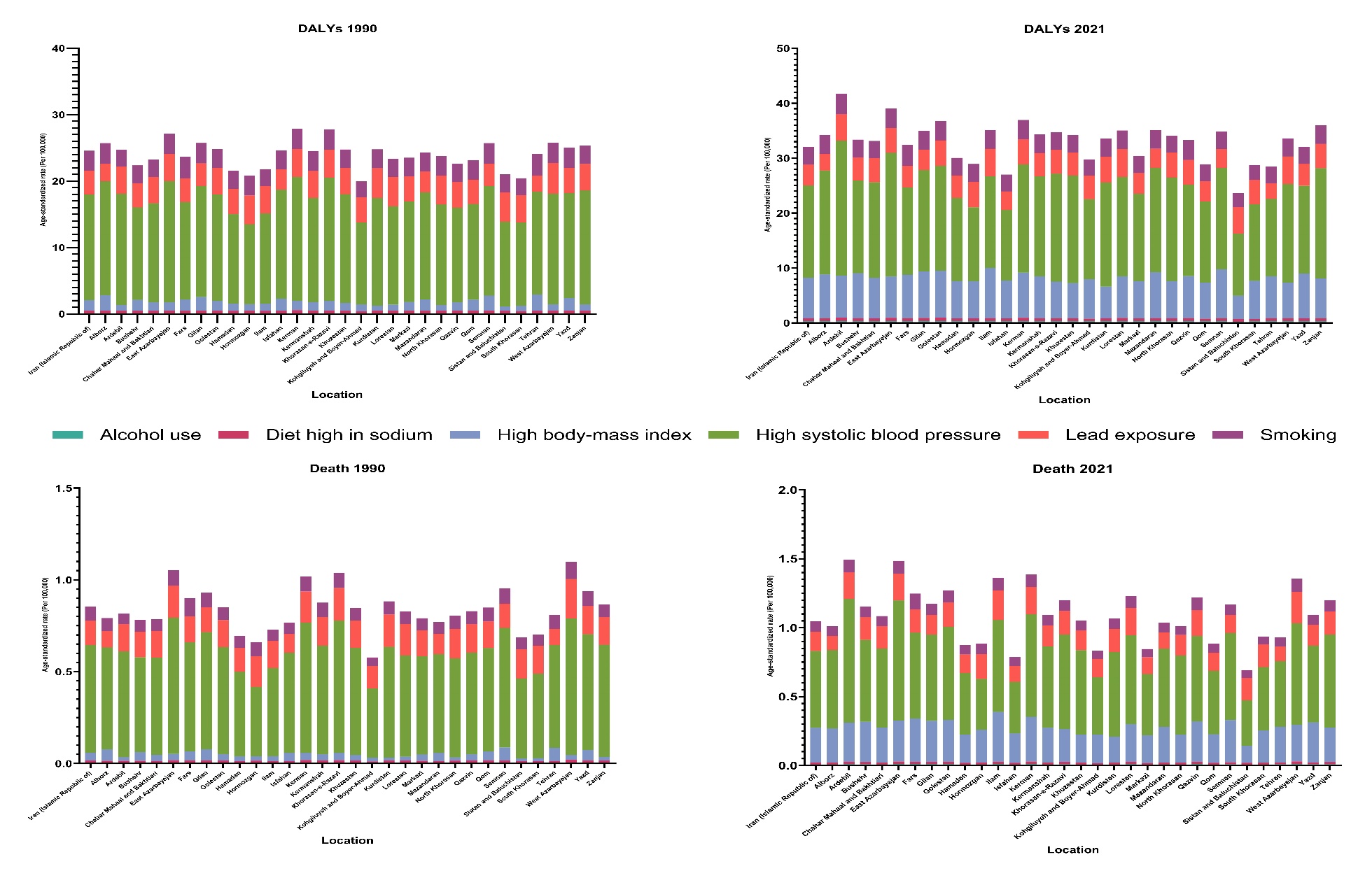
 **Figure S6.** Age-standardized rate of disability-adjusted life years (DALYs) and deaths of atrial fibrillation and flutter attributable to risk factors among men in 1990 and 2021 in Iran


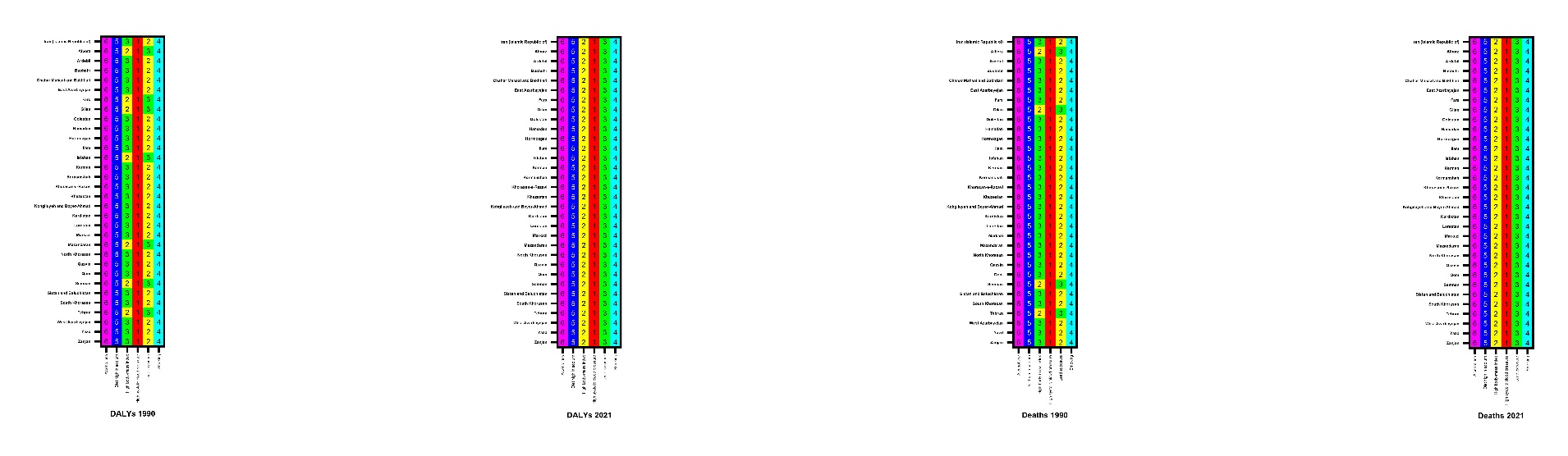
**Figure S7.** National and province-specific ranking of age-standardized rate of disability-adjusted life years (DALYs) and deaths of atrial fibrillation and flutter attributable to risk factors among both sexes in 1990 and 2021 in Iran


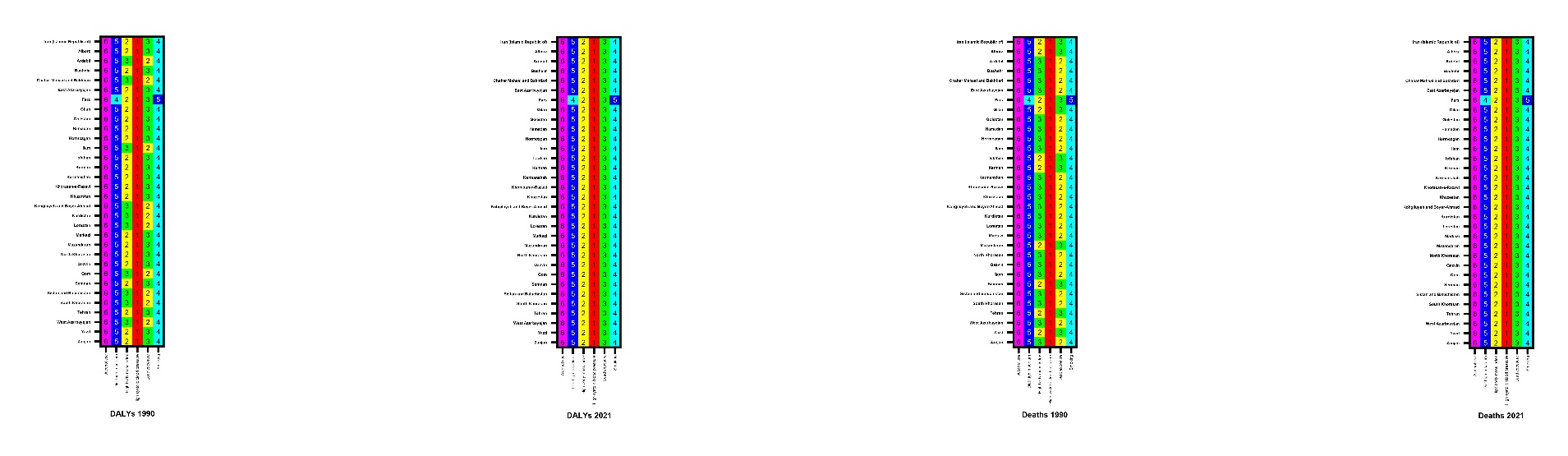
 **Figure S8.** National and province-specific ranking of age-standardized rate of disability-adjusted life years (DALYs) and deaths of atrial fibrillation and flutter attributable to risk factors among women in 1990 and 2021 in Iran


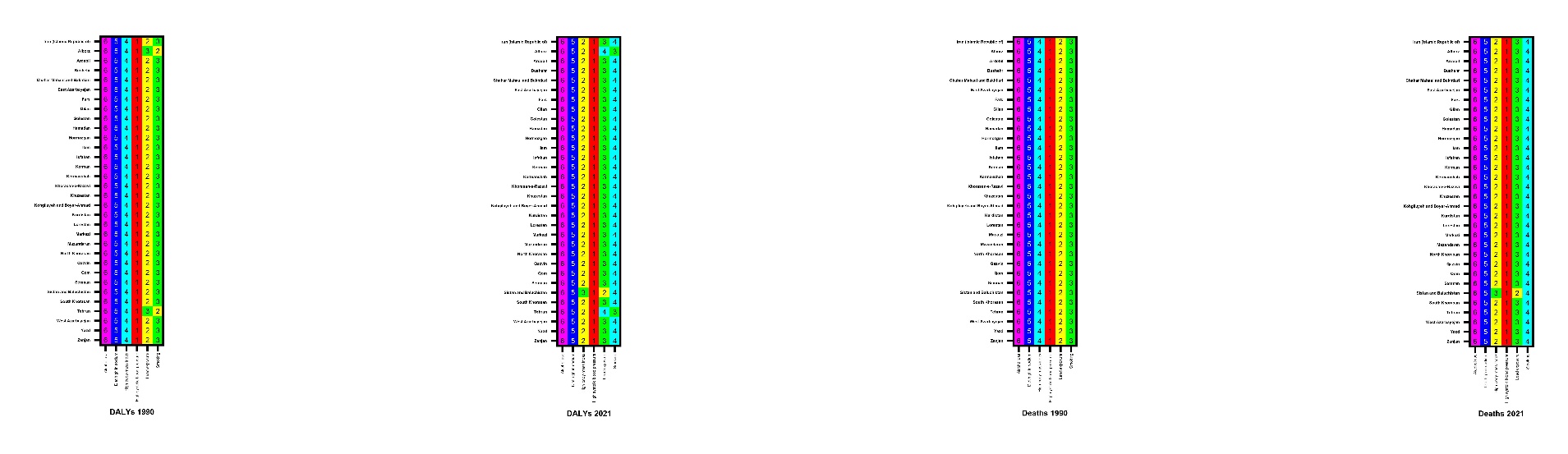
 **Figure S9.** National and province-specific ranking of age-standardized rate of disability-adjusted life years (DALYs) and deaths of atrial fibrillation and flutter attributable to risk factors among men in 1990 and 2021 in Iran


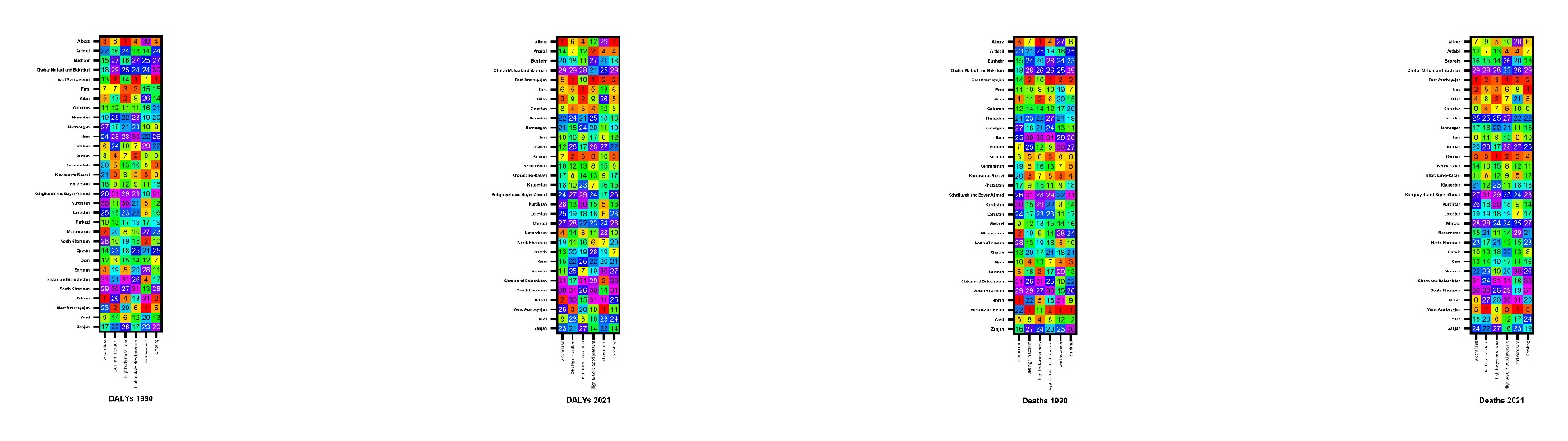
 **Figure S10.** Sub-national ranking of the age-standardized rate of disability-adjusted life years (DALYs) and deaths of atrial fibrillation and flutter attributable to risk factors among both sexes in 1990 and 2021 in Iran


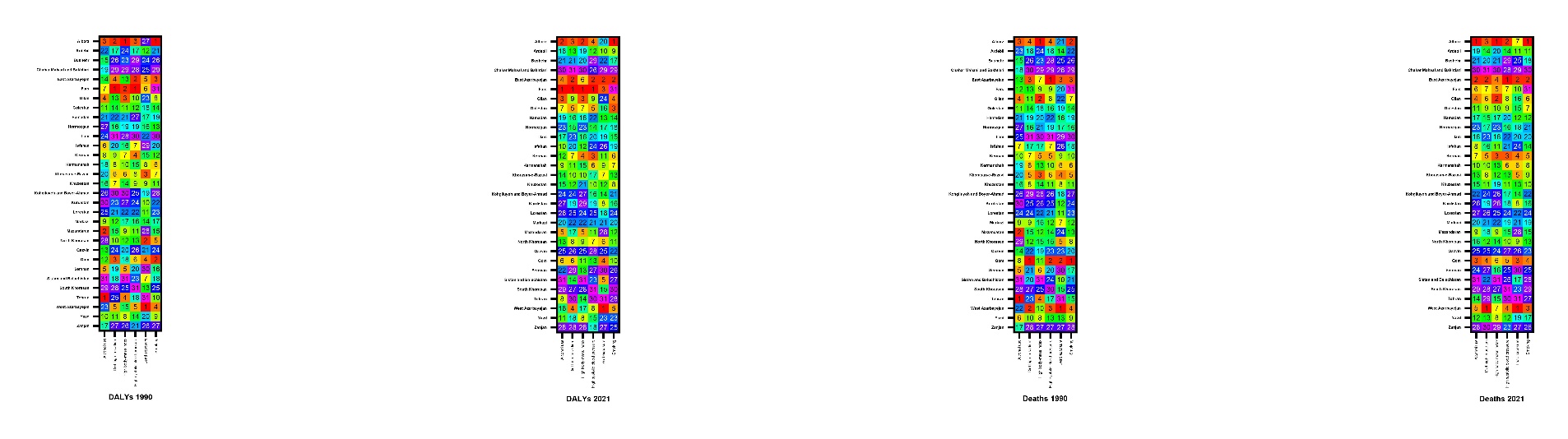
 **Figure S11.** Sub-national ranking of the age-standardized rate of disability-adjusted life years (DALYs) and deaths of atrial fibrillation and flutter attributable to risk factors among women in 1990 and 2021 in Iran


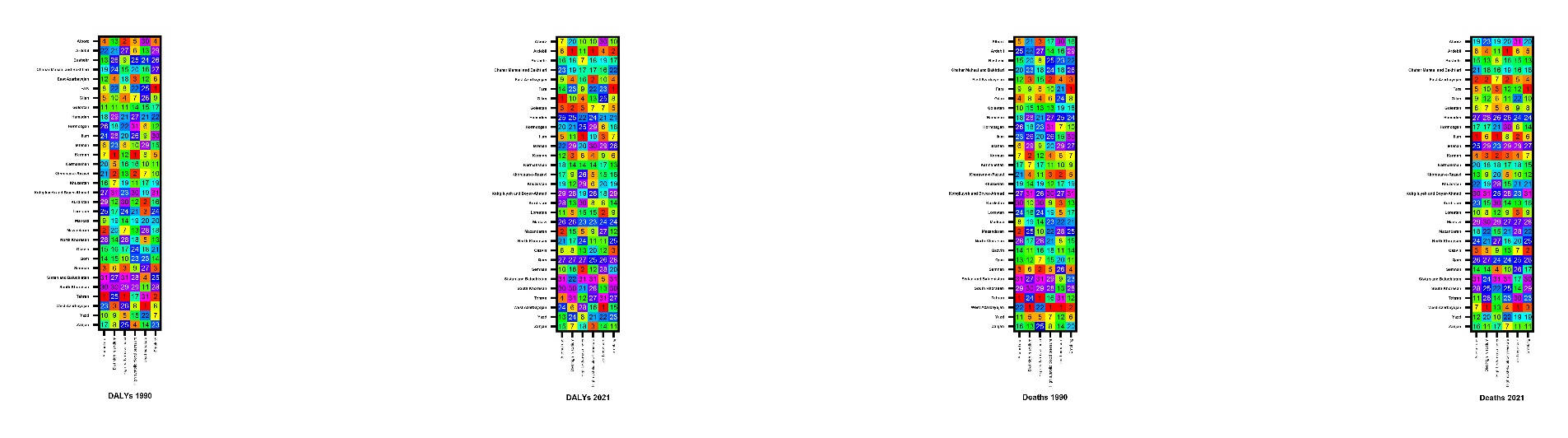
 **Figure S12.** Sub-national ranking of the age-standardized rate of disability-adjusted life years (DALYs) and deaths of atrial fibrillation and flutter attributable to risk factors among men in 1990 and 2021 in Iran

**Table S1.** All‑ages number and age‑standardized rate of incidence, prevalence, disability-adjusted life years (DALYs), and deaths of atrial fibrillation and flutter by sex in 1990 and 2021 and overall percent change over 1990–2021 in Iran provinces

| Location | Measure | Age, Metric | Year | | | | | | % Change (1990 to 2021) | | |
| --- | --- | --- | --- | --- | --- | --- | --- | --- | --- | --- | --- |
|  |  |  | 1990 | | | 2021 | | |  |  |  |
|  |  |  | Both | Women | Men | Both | Women | Men | Both | Women | Men |
| Iran (Islamic Republic of) | Incidence | Age-standardized | 37.3 (27.6 to 50.2) | 35 (25.9 to 46.8) | 40 (29.6 to 53.7) | 40.6 (30 to 54.4) | 37.4 (27.7 to 50.2) | 43.7 (32.3 to 58.7) | 8.6 (6.9 to 10.2) | 7 (5.3 to 8.8) | 9.2 (6.4 to 11.6) |
|  |  | All ages | 8136.4 (6131.5 to 10786.8) | 3690.9 (2775 to 4890.5) | 4445.5 (3327.9 to 5896.3) | 29418.3 (22665.1 to 38723.1) | 13622.3 (10363.4 to 18155.8) | 15796 (12121.5 to 20711) | 261.6 (236.1 to 289.9) | 269.1 (249.1 to 290.5) | 255.3 (223.9 to 291.2) |
|  | Prevalence | Age-standardized | 386 (296.2 to 507.6) | 358.1 (273.8 to 471.7) | 416.7 (318.6 to 546.4) | 425.4 (327.2 to 559.2) | 387.8 (298 to 510.7) | 461.1 (354.7 to 601.6) | 10.2 (8.6 to 11.7) | 8.3 (6.4 to 10.2) | 10.7 (8.1 to 12.8) |
|  |  | All ages | 75832.5 (58813.8 to 99095.2) | 34230.7 (26107.8 to 44725) | 41601.8 (32447.4 to 54379.3) | 294248.7 (230024.2 to 382165.9) | 134056.9 (103750.2 to 176465.5) | 160191.8 (124346.3 to 206830.5) | 288 (268.9 to 313.2) | 291.6 (274.1 to 311) | 285.1 (260.5 to 316.7) |
|  | DALYs (Disability-Adjusted Life Years) | Age-standardized | 70.5 (55.4 to 88.3) | 78.7 (61.2 to 98.7) | 61.4 (46.2 to 76.9) | 72.4 (57.4 to 88.3) | 83.5 (65.3 to 99.2) | 62.5 (47.4 to 80.3) | 2.6 (-13.1 to 18.2) | 6.1 (-14.5 to 24.6) | 1.8 (-9.9 to 23) |
|  |  | All ages | 12115.7 (9430.1 to 15214.4) | 6569.4 (5120.9 to 8236.8) | 5546.3 (4130.2 to 7086.4) | 47524.7 (37527.7 to 58270.9) | 26310.9 (20508.1 to 31274.3) | 21213.7 (16083.8 to 27193.4) | 292.3 (239.7 to 352.3) | 300.5 (227.6 to 368.7) | 282.5 (236.4 to 359.6) |
|  | Deaths | Age-standardized | 3.3 (2.4 to 4.1) | 4.1 (2.9 to 5.3) | 2.4 (1.6 to 2.9) | 3.3 (2.5 to 3.8) | 4.6 (3.3 to 5.3) | 2.2 (1.5 to 2.7) | -0.4 (-22.3 to 28.3) | 10.2 (-18.8 to 40.3) | -6.6 (-24 to 41.4) |
|  |  | All ages | 410.6 (305.9 to 509.9) | 266.4 (193.5 to 341.2) | 144.2 (97.9 to 176.6) | 1948.9 (1462.6 to 2233.1) | 1258.4 (920 to 1445.6) | 690.6 (478.6 to 823.5) | 374.6 (268.7 to 511.3) | 372.3 (246.1 to 502.3) | 378.7 (281.6 to 618.5) |
| Alborz | Incidence | Age-standardized | 37.1 (27.8 to 50) | 34 (25.2 to 45.6) | 40.7 (30.2 to 55.3) | 41.5 (30.7 to 56.2) | 37 (27.2 to 49.4) | 45.4 (33.4 to 62.1) | 11.8 (6.8 to 17.3) | 8.9 (1.6 to 17.2) | 11.5 (3.7 to 17.7) |
|  |  | All ages | 184.1 (138.6 to 245.2) | 80.7 (60 to 107.4) | 103.4 (77 to 136.9) | 973.7 (745.4 to 1286.5) | 420.5 (317.3 to 556.6) | 553.2 (426.7 to 737.3) | 428.9 (400.1 to 456.5) | 420.9 (386.8 to 464.6) | 435.1 (391.9 to 479.5) |
|  | Prevalence | Age-standardized | 385.3 (293.5 to 507.9) | 348.8 (264.9 to 457) | 425.2 (322.9 to 559.6) | 439 (336.3 to 579.4) | 385 (292.3 to 505.6) | 481.2 (370.7 to 639.9) | 13.9 (9.1 to 18.8) | 10.4 (2.9 to 19) | 13.2 (5.9 to 18.9) |
|  |  | All ages | 1699.4 (1314.6 to 2226.1) | 748.5 (571.7 to 994) | 951 (730.6 to 1242.2) | 9335.2 (7256.4 to 12223.4) | 3912.7 (3009 to 5182.6) | 5422.5 (4151.6 to 7061.2) | 449.3 (420.1 to 477.1) | 422.8 (383.7 to 461.1) | 470.2 (430.7 to 515.2) |
|  | DALYs (Disability-Adjusted Life Years) | Age-standardized | 77.2 (60.2 to 98) | 92.6 (69.7 to 124.3) | 59.9 (42.4 to 80.8) | 76 (58.5 to 93.7) | 103.6 (77.6 to 126.2) | 62 (45.1 to 81) | -1.5 (-22.2 to 19.3) | 11.9 (-22.7 to 48.1) | 3.6 (-14.8 to 27.1) |
|  |  | All ages | 283.3 (215.8 to 360.6) | 160.8 (121.5 to 211.3) | 122.5 (85.1 to 164) | 1420.6 (1091.7 to 1777.8) | 757.2 (584 to 936.3) | 663.4 (484.7 to 869.4) | 401.4 (311 to 497.1) | 370.8 (243.1 to 508.8) | 441.6 (350 to 561.1) |
|  | Deaths | Age-standardized | 3.8 (2.8 to 5.1) | 5.4 (3.8 to 7.7) | 2 (1.2 to 3) | 3.6 (2.5 to 4.3) | 6.7 (4.5 to 8.5) | 2 (1.3 to 2.7) | -7.3 (-36.3 to 26) | 24.1 (-26.2 to 82.4) | -2 (-35.6 to 59) |
|  |  | All ages | 9.3 (7.1 to 12.3) | 6.7 (4.7 to 9.6) | 2.6 (1.7 to 3.8) | 50.2 (36.5 to 60.9) | 33.2 (23.6 to 41.9) | 17.1 (11.1 to 22.4) | 440.6 (277.6 to 648.8) | 398.4 (211.3 to 653.1) | 547.1 (323.9 to 938.6) |
| Ardebil | Incidence | Age-standardized | 37.4 (27.5 to 50.1) | 34 (25.1 to 45.5) | 40.5 (29.7 to 54.8) | 41.3 (30.5 to 55.6) | 37.5 (27.7 to 50.4) | 45.7 (33.4 to 61.7) | 10.4 (5.1 to 15.7) | 10.2 (3.5 to 18.7) | 12.8 (3.8 to 21.3) |
|  |  | All ages | 152.5 (114.9 to 202.5) | 63.6 (47.9 to 83.4) | 88.8 (66.6 to 120.2) | 469 (355.3 to 623.3) | 227.2 (171.3 to 302.8) | 241.7 (181.8 to 324) | 207.6 (179.7 to 238.1) | 257.1 (221.6 to 299.1) | 172.2 (141 to 203.9) |
|  | Prevalence | Age-standardized | 385.4 (293.7 to 504.7) | 347.9 (264.2 to 455.3) | 421.3 (325.2 to 554.9) | 431.4 (330.9 to 567) | 388.3 (296.3 to 515.6) | 483.2 (366.2 to 644.8) | 12 (7.3 to 17.1) | 11.6 (4.5 to 19.6) | 14.7 (6.8 to 22.6) |
|  |  | All ages | 1424.1 (1115 to 1851.7) | 583.9 (450.7 to 768.9) | 840.2 (651.9 to 1108.4) | 4710.5 (3625 to 6192.5) | 2283.3 (1745.3 to 3016.2) | 2427.3 (1865.9 to 3175.1) | 230.8 (206.1 to 257.6) | 291 (254 to 328.6) | 188.9 (161.9 to 215.3) |
|  | DALYs (Disability-Adjusted Life Years) | Age-standardized | 66.2 (50.3 to 84.8) | 73.8 (54.7 to 96.9) | 59 (42.9 to 75.7) | 78.6 (60.8 to 95) | 84.4 (64.5 to 102.4) | 70.7 (52.3 to 89.4) | 18.8 (-2.3 to 46.3) | 14.4 (-13.9 to 45.8) | 19.8 (0.6 to 58.3) |
|  |  | All ages | 220.6 (167.1 to 281.3) | 112.7 (84 to 146.2) | 107.9 (78.1 to 142.4) | 815.2 (634.4 to 986.6) | 475.2 (366.5 to 574.3) | 340 (253.9 to 426.2) | 269.5 (208.7 to 348.3) | 321.5 (217.9 to 438.5) | 215.1 (165 to 315.8) |
|  | Deaths | Age-standardized | 2.9 (2 to 3.9) | 3.7 (2.5 to 5.3) | 2.2 (1.3 to 2.8) | 3.8 (2.7 to 4.5) | 4.5 (3.1 to 5.5) | 2.7 (1.8 to 3.4) | 28.2 (-6.6 to 81.4) | 22.9 (-19.6 to 73.9) | 24.6 (-11.8 to 130.7) |
|  |  | All ages | 7.3 (4.9 to 9.6) | 4.5 (3.1 to 6.4) | 2.8 (1.6 to 3.7) | 35.1 (25.4 to 41.8) | 23.9 (16.8 to 29.2) | 11.2 (7.6 to 14.1) | 383.1 (251.7 to 590.2) | 430.3 (247.4 to 662.8) | 306.4 (187 to 653.1) |
| Bushehr | Incidence | Age-standardized | 35.9 (26.9 to 47.5) | 32.9 (24.2 to 43.7) | 39.2 (29.3 to 51.5) | 39.3 (29.5 to 52.2) | 35.5 (26.6 to 47.8) | 43.3 (32.1 to 58) | 9.4 (4.5 to 14.5) | 8.2 (1.5 to 14.9) | 10.3 (2.7 to 19.2) |
|  |  | All ages | 79 (59.3 to 104.4) | 35.4 (26.7 to 46.9) | 43.6 (33 to 57.4) | 322 (248.3 to 423.7) | 146.8 (111.9 to 194.5) | 175.2 (134.1 to 230.2) | 307.7 (277 to 344.2) | 315 (278.2 to 356) | 301.7 (265.5 to 343.6) |
|  | Prevalence | Age-standardized | 370.4 (284.1 to 482.8) | 335.2 (253.5 to 442.9) | 407.9 (313.9 to 525.5) | 410.8 (315.6 to 537.6) | 367.4 (282.3 to 485.5) | 456 (348.4 to 601.6) | 10.9 (6.1 to 15.7) | 9.6 (3 to 16.1) | 11.8 (5.1 to 20.4) |
|  |  | All ages | 727.6 (568.8 to 946.6) | 322.8 (248.9 to 430.3) | 404.9 (319 to 526.3) | 3110.6 (2445 to 4083.4) | 1413.3 (1100.8 to 1852.8) | 1697.3 (1322.2 to 2212) | 327.5 (299.7 to 357.7) | 337.9 (307.6 to 373.8) | 319.2 (285.8 to 356.7) |
|  | DALYs (Disability-Adjusted Life Years) | Age-standardized | 63.8 (49 to 81.3) | 68.2 (52.8 to 87.8) | 59 (43 to 75.4) | 71.4 (55.4 to 88.1) | 78 (59.9 to 94.6) | 64.1 (47.7 to 81.9) | 11.9 (-6.4 to 37) | 14.4 (-11.1 to 45.6) | 8.5 (-9.4 to 41.1) |
|  |  | All ages | 111.1 (84.6 to 142.7) | 58.5 (45 to 75.9) | 52.6 (38.2 to 68) | 484.8 (375.1 to 602.9) | 264.4 (201.5 to 322.3) | 220.4 (164.4 to 283.2) | 336.5 (269.7 to 427.5) | 352.1 (254.9 to 466.5) | 319.2 (249.5 to 426.8) |
|  | Deaths | Age-standardized | 2.9 (2 to 3.6) | 3.4 (2.3 to 4.5) | 2.3 (1.4 to 3.1) | 3.3 (2.4 to 3.9) | 4.2 (3 to 5) | 2.4 (1.5 to 3) | 16.2 (-13 to 64.5) | 24.2 (-15.7 to 77.4) | 4.3 (-25.4 to 88.8) |
|  |  | All ages | 3.6 (2.6 to 4.6) | 2.2 (1.5 to 3) | 1.4 (0.9 to 1.8) | 17.6 (13.2 to 20.8) | 11.4 (8.2 to 13.7) | 6.3 (4.1 to 7.9) | 393.7 (267.7 to 595.6) | 414.4 (247.1 to 634.6) | 360.2 (222.7 to 722.5) |
| Chahar Mahaal and Bakhtiari | Incidence | Age-standardized | 36 (26.7 to 48.6) | 32.9 (24.3 to 43.9) | 39.4 (29.2 to 53.1) | 39.2 (29.2 to 52.1) | 35.7 (26.8 to 47.8) | 43.5 (32.3 to 57.8) | 8.9 (4.8 to 14.3) | 8.5 (1.7 to 16.6) | 10.3 (1.5 to 18.4) |
|  |  | All ages | 88.6 (66.5 to 117.7) | 37.9 (28.5 to 49.5) | 50.7 (37.1 to 68) | 322 (247.5 to 419.9) | 155.3 (118.1 to 203.9) | 166.7 (127.9 to 216.4) | 263.3 (236.2 to 296.7) | 309.7 (277.8 to 350.4) | 228.7 (192.6 to 267.5) |
|  | Prevalence | Age-standardized | 371.3 (283.3 to 490.5) | 336.5 (257.2 to 439.6) | 411 (312.4 to 543.3) | 411.3 (317.9 to 535.3) | 370.9 (280.6 to 484.6) | 460.4 (355.9 to 601.1) | 10.8 (6.3 to 15.7) | 10.2 (3.2 to 18.3) | 12 (4.3 to 20.5) |
|  |  | All ages | 824.2 (639.2 to 1064.5) | 352.9 (271.8 to 460.5) | 471.3 (362.8 to 614) | 3219.4 (2538.2 to 4172.2) | 1560.7 (1191.5 to 2029.2) | 1658.7 (1293.1 to 2130.9) | 290.6 (266.3 to 321.1) | 342.3 (309.4 to 384.8) | 251.9 (221.8 to 283.9) |
|  | DALYs (Disability-Adjusted Life Years) | Age-standardized | 61.5 (47.3 to 78.5) | 63.6 (46.6 to 81.2) | 59.1 (42.5 to 77.2) | 64.3 (49.2 to 79.9) | 65 (48.2 to 81.9) | 62.7 (46.7 to 80.1) | 4.5 (-14 to 28.2) | 2.2 (-22.6 to 26.8) | 6 (-11.9 to 38.2) |
|  |  | All ages | 121.5 (92.9 to 154.7) | 60.8 (45.5 to 77) | 60.7 (42.6 to 80.5) | 478.2 (364.6 to 602.2) | 263.1 (196.2 to 332.9) | 215.1 (161 to 277.6) | 293.5 (226.4 to 372.5) | 332.3 (230.2 to 435) | 254.6 (192.7 to 352.4) |
|  | Deaths | Age-standardized | 2.6 (1.8 to 3.4) | 3 (2 to 3.9) | 2.2 (1.3 to 2.9) | 2.8 (2.1 to 3.4) | 3.1 (2.2 to 4) | 2.2 (1.5 to 2.8) | 5 (-24.4 to 51.6) | 5.6 (-31.4 to 48.6) | 0.6 (-27.7 to 80.3) |
|  |  | All ages | 3.7 (2.6 to 4.7) | 2.3 (1.6 to 3) | 1.4 (0.9 to 1.9) | 18.4 (13.8 to 22.5) | 11.9 (8.4 to 15.1) | 6.5 (4.5 to 8.1) | 397.4 (259.9 to 617.8) | 425.8 (240.4 to 638.6) | 352.7 (210.7 to 708.2) |
| East Azarbayejan | Incidence | Age-standardized | 37.1 (27.7 to 49.6) | 33.7 (25.4 to 45.2) | 40.4 (29.8 to 54.4) | 40.8 (30 to 54.4) | 36.7 (26.9 to 49.2) | 44.9 (33.3 to 60.2) | 9.7 (5.5 to 14.7) | 8.8 (1.9 to 16.1) | 11 (5.4 to 20.1) |
|  |  | All ages | 492.8 (367.6 to 645) | 210.3 (157.8 to 277.2) | 282.5 (208.6 to 370.1) | 1523.2 (1163 to 2025.7) | 693.1 (522.4 to 927.9) | 830.1 (635.5 to 1093.3) | 209.1 (182 to 243.1) | 229.6 (198.6 to 265.2) | 193.8 (162.1 to 229.1) |
|  | Prevalence | Age-standardized | 382.9 (296.4 to 503.1) | 344.2 (264.6 to 451.3) | 420.7 (321.8 to 558.8) | 425.9 (325.8 to 554.9) | 378.9 (290.2 to 503.5) | 473.3 (364.3 to 613.3) | 11.2 (6.6 to 16.6) | 10.1 (3.2 to 17) | 12.5 (6.5 to 21) |
|  |  | All ages | 4551.2 (3506.3 to 5903.4) | 1905.4 (1479.9 to 2499.2) | 2645.8 (2027.6 to 3405.4) | 14983.7 (11699.5 to 19453.4) | 6747.9 (5235.2 to 8899.7) | 8235.8 (6429.3 to 10585.8) | 229.2 (204.2 to 256.5) | 254.1 (223.5 to 286.1) | 211.3 (183 to 243.3) |
|  | DALYs (Disability-Adjusted Life Years) | Age-standardized | 80 (61.3 to 100.4) | 93 (68.2 to 118.3) | 67.2 (48.4 to 86.2) | 87.1 (69.1 to 106.4) | 103.4 (80 to 126.2) | 70.8 (52.9 to 89.2) | 8.9 (-11.8 to 34) | 11.3 (-18 to 45.5) | 5.3 (-11.6 to 36.3) |
|  |  | All ages | 772.9 (591.2 to 977.7) | 409.6 (308.6 to 514.6) | 363.3 (266.2 to 484) | 2615.8 (2077.1 to 3238.2) | 1503.4 (1172.8 to 1844.2) | 1112.4 (818.5 to 1425.7) | 238.4 (182.3 to 311.8) | 267 (172.6 to 374) | 206.2 (156.9 to 279.4) |
|  | Deaths | Age-standardized | 4.3 (3 to 5.5) | 5.7 (3.9 to 7.6) | 3 (1.8 to 3.9) | 4.7 (3.4 to 5.8) | 6.6 (4.4 to 8.2) | 2.9 (1.8 to 3.8) | 10.2 (-20.3 to 54.8) | 16.1 (-23.7 to 66.6) | -0.9 (-30.5 to 72.4) |
|  |  | All ages | 28.4 (20.2 to 35.7) | 18.2 (12.7 to 24.2) | 10.2 (6.6 to 13.3) | 110 (80.7 to 133.5) | 75.4 (52.7 to 94.8) | 34.5 (22.2 to 45.3) | 287.5 (180.5 to 444.8) | 314.3 (174 to 500.1) | 239.5 (140.7 to 481.5) |
| Fars | Incidence | Age-standardized | 46.3 (34.1 to 63) | 55.8 (41.5 to 75.2) | 37.2 (27.1 to 51.7) | 49 (36.1 to 66.8) | 57.8 (42.9 to 77.4) | 39.8 (28.7 to 55.5) | 5.9 (1.1 to 11.2) | 3.5 (-2.1 to 10) | 7 (-1.4 to 14.6) |
|  |  | All ages | 607.6 (446.1 to 823.9) | 366.1 (267.2 to 491.8) | 241.5 (173.6 to 331.9) | 2190.2 (1640.6 to 2929.7) | 1329.1 (995.3 to 1774.2) | 861.2 (637.7 to 1191.2) | 260.5 (231.2 to 293.1) | 263.1 (232.5 to 295.5) | 256.6 (220.1 to 296.2) |
|  | Prevalence | Age-standardized | 490.8 (368.3 to 648.4) | 592.2 (447 to 778.2) | 383.9 (282.5 to 516.5) | 520.9 (394.7 to 694.4) | 619.4 (468.6 to 822.5) | 416.8 (308.4 to 572.6) | 6.1 (1 to 11.9) | 4.6 (-2 to 11.9) | 8.6 (1.3 to 15.9) |
|  |  | All ages | 5616.8 (4200 to 7393.5) | 3391.1 (2569 to 4492) | 2225.8 (1667.4 to 2993) | 21782.3 (16721.8 to 28803.5) | 13267.1 (10170.6 to 17638.3) | 8515.2 (6379.4 to 11592.4) | 287.8 (260.4 to 319.5) | 291.2 (261.5 to 324.4) | 282.6 (243.4 to 319.2) |
|  | DALYs (Disability-Adjusted Life Years) | Age-standardized | 80 (61.5 to 100.9) | 96.9 (73.7 to 125.9) | 60.6 (43.7 to 78.3) | 85.8 (66.9 to 108.1) | 108.7 (84.4 to 135.2) | 63.1 (45.4 to 82.2) | 7.3 (-11.4 to 25.1) | 12.2 (-12.8 to 36.9) | 4.1 (-15.7 to 31.3) |
|  |  | All ages | 796.5 (599.7 to 1024.8) | 486.6 (368.8 to 632.6) | 309.9 (220 to 414.5) | 3270.6 (2556.1 to 4141.8) | 2057.2 (1604.5 to 2597.4) | 1213.4 (868 to 1574.7) | 310.6 (239.6 to 377.9) | 322.8 (232.2 to 417.9) | 291.6 (216.4 to 394.7) |
|  | Deaths | Age-standardized | 3.5 (2.5 to 4.5) | 4.3 (3.1 to 5.7) | 2.6 (1.7 to 3.4) | 4 (2.9 to 4.7) | 5.4 (3.9 to 6.6) | 2.6 (1.7 to 3.3) | 12.6 (-18.3 to 50.8) | 26.8 (-14.4 to 75.3) | 2.2 (-27.3 to 61.8) |
|  |  | All ages | 23.5 (17.7 to 29.4) | 15.3 (11.3 to 20.4) | 8.2 (5.4 to 11.1) | 125 (93.2 to 147.5) | 82.6 (59.8 to 101) | 42.4 (27.5 to 52.6) | 432.5 (279.8 to 615.1) | 440.8 (260.9 to 660.8) | 417 (256.2 to 716.5) |
| Gilan | Incidence | Age-standardized | 36.4 (27 to 49) | 33.7 (25.1 to 45.9) | 40.3 (29.4 to 54.4) | 40.6 (30 to 55) | 36.6 (27.1 to 49.9) | 44.7 (32.8 to 59.6) | 11.5 (6.2 to 17) | 8.6 (0.6 to 15.4) | 11 (2.3 to 20) |
|  |  | All ages | 381 (282.4 to 505.4) | 183.8 (136.5 to 245.9) | 197.2 (146.8 to 261.6) | 1220.9 (918.9 to 1636.4) | 561.4 (423.4 to 763.2) | 659.5 (494.4 to 873.6) | 220.5 (193.8 to 254) | 205.5 (184.3 to 233.9) | 234.4 (195 to 280.2) |
|  | Prevalence | Age-standardized | 375.5 (285.7 to 494.9) | 345.1 (260.7 to 454.7) | 419.7 (321.3 to 557.5) | 426.6 (327.5 to 567.7) | 380 (291.3 to 512.9) | 473.4 (359.3 to 621.1) | 13.6 (8.6 to 18.9) | 10.1 (2.5 to 16) | 12.8 (4.7 to 21.2) |
|  |  | All ages | 3550.4 (2742.2 to 4670.3) | 1725.5 (1324.9 to 2297.4) | 1824.9 (1408.8 to 2411.7) | 12284.7 (9487.7 to 16119.6) | 5563.4 (4284.1 to 7474.6) | 6721.3 (5152.3 to 8683.4) | 246 (219.6 to 278.3) | 222.4 (199.8 to 249.7) | 268.3 (233.9 to 314.6) |
|  | DALYs (Disability-Adjusted Life Years) | Age-standardized | 74.6 (57.5 to 94.2) | 80.7 (60.8 to 104.6) | 64.2 (47.4 to 82.3) | 77.9 (60.9 to 93.6) | 90.9 (69.4 to 109) | 65.8 (48.7 to 83.4) | 4.5 (-16.4 to 27.1) | 12.6 (-16.8 to 45.7) | 2.5 (-14.6 to 28.9) |
|  |  | All ages | 606.2 (469.7 to 770.1) | 360.8 (268 to 464.3) | 245.4 (181 to 323.6) | 2086.2 (1632.2 to 2524.4) | 1186.8 (909 to 1424.2) | 899.4 (660.1 to 1152.7) | 244.1 (183.2 to 312.8) | 229 (148.3 to 321) | 266.4 (206.5 to 362) |
|  | Deaths | Age-standardized | 3.8 (2.7 to 5.1) | 4.4 (3.1 to 6.2) | 2.6 (1.6 to 3.5) | 3.8 (2.8 to 4.5) | 5.3 (3.7 to 6.6) | 2.4 (1.6 to 3.1) | 0.4 (-30 to 39.6) | 19.7 (-24.1 to 71.7) | -8.1 (-36 to 53.9) |
|  |  | All ages | 22.9 (16.8 to 30) | 16.6 (11.7 to 23.1) | 6.3 (4.1 to 8.3) | 88.4 (64.5 to 104.5) | 59.5 (42.3 to 73.7) | 28.9 (19.1 to 36.8) | 285.9 (171.4 to 435.8) | 258.5 (126 to 418.9) | 358.1 (228.3 to 641.1) |
| Golestan | Incidence | Age-standardized | 37.1 (27.4 to 50) | 34 (25.1 to 46.3) | 40.7 (30.2 to 54.7) | 41 (30.1 to 54.8) | 37.3 (28 to 50.1) | 45.3 (32.9 to 60) | 10.6 (5.4 to 16.5) | 9.8 (2.2 to 17.3) | 11.3 (3.1 to 20.1) |
|  |  | All ages | 164.2 (122.7 to 217.8) | 73.1 (55 to 97.2) | 91.1 (68.3 to 122.9) | 595.2 (457 to 776.6) | 288.1 (221 to 384.2) | 307.1 (232.1 to 401.5) | 262.5 (235.9 to 294.7) | 294 (259.6 to 329) | 237.3 (207.6 to 278.2) |
|  | Prevalence | Age-standardized | 379.5 (289.6 to 497.9) | 345.6 (261.8 to 455.9) | 421.3 (318.1 to 554.2) | 427.4 (325.3 to 559.3) | 384 (294.8 to 506.3) | 476.5 (359.2 to 617.6) | 12.6 (7.4 to 18.6) | 11.1 (3.2 to 18.3) | 13.1 (5.4 to 21.6) |
|  |  | All ages | 1497.8 (1155.3 to 1972.5) | 666.2 (504.1 to 882) | 831.7 (643.2 to 1095.2) | 5790.5 (4533.3 to 7529.2) | 2777.6 (2147.5 to 3650.7) | 3012.9 (2338 to 3925.6) | 286.6 (262.5 to 318.5) | 317 (283.2 to 350.8) | 262.3 (233.5 to 300.1) |
|  | DALYs (Disability-Adjusted Life Years) | Age-standardized | 70.6 (55.1 to 88.6) | 77.3 (56.6 to 99.2) | 62 (45.1 to 82.2) | 80.1 (61.7 to 97.7) | 90.2 (68.6 to 109.2) | 69.5 (51.8 to 88) | 13.6 (-6.5 to 34.8) | 16.7 (-11.3 to 43.1) | 12 (-5 to 39.5) |
|  |  | All ages | 243.2 (185.9 to 311.3) | 132.8 (98.5 to 170.7) | 110.4 (77.4 to 150.1) | 1011.4 (784.8 to 1237.7) | 590.4 (451.3 to 718.7) | 421 (312.1 to 536.5) | 315.9 (248.1 to 394.9) | 344.7 (239.8 to 446.9) | 281.3 (218.6 to 372.2) |
|  | Deaths | Age-standardized | 3.2 (2.3 to 4.1) | 3.9 (2.7 to 5.1) | 2.3 (1.5 to 3.2) | 3.7 (2.7 to 4.5) | 4.9 (3.5 to 6) | 2.6 (1.7 to 3.2) | 15.2 (-17 to 50.3) | 25.1 (-16 to 70.1) | 9.4 (-20.3 to 70.8) |
|  |  | All ages | 7.5 (5.4 to 9.4) | 5 (3.6 to 6.6) | 2.5 (1.6 to 3.4) | 39.7 (28.6 to 47) | 26.7 (19.3 to 32.7) | 13 (8.6 to 16.1) | 428.2 (285.7 to 594.7) | 431.2 (258.2 to 614.2) | 422.1 (273.6 to 712.4) |
| Hamadan | Incidence | Age-standardized | 35.9 (26.4 to 48) | 32.6 (23.8 to 44.3) | 39.1 (28.7 to 52.3) | 39.3 (29.2 to 53.1) | 35.4 (26.2 to 47.5) | 43.3 (32.1 to 58.8) | 9.6 (4.3 to 15.5) | 8.4 (0.4 to 15.5) | 10.8 (2.9 to 19) |
|  |  | All ages | 245.1 (178.9 to 327.3) | 105.8 (78.2 to 140.5) | 139.3 (101.6 to 185.5) | 706.4 (534.3 to 928.7) | 313.9 (233.4 to 418.2) | 392.4 (297.9 to 516.1) | 188.2 (160.6 to 218.6) | 196.6 (166.1 to 225) | 181.7 (153.2 to 217.8) |
|  | Prevalence | Age-standardized | 368.5 (279.3 to 483.6) | 331.1 (249.9 to 436) | 404.4 (308.1 to 530.4) | 411.3 (314.2 to 546.3) | 364.1 (276 to 485.5) | 454.8 (345.9 to 601.3) | 11.6 (6.2 to 17) | 10 (2.5 to 17.1) | 12.5 (5.2 to 20.6) |
|  |  | All ages | 2294.9 (1775.2 to 2992.6) | 975.7 (742.4 to 1284.2) | 1319.2 (1019.7 to 1718.1) | 7297.7 (5648.9 to 9604.4) | 3150.6 (2409.4 to 4163.3) | 4147.1 (3209.7 to 5390.4) | 218 (192.5 to 247) | 222.9 (195.2 to 251.9) | 214.4 (184.7 to 250.6) |
|  | DALYs (Disability-Adjusted Life Years) | Age-standardized | 64.6 (48.8 to 82.2) | 72.4 (53.8 to 94.4) | 57.1 (41.2 to 75.3) | 69 (52.5 to 84.6) | 81.9 (61.4 to 100.2) | 58.9 (43.5 to 76.1) | 6.8 (-12.8 to 30.6) | 13.1 (-15.3 to 44.5) | 3.1 (-14.4 to 32.3) |
|  |  | All ages | 363.1 (277 to 463.4) | 187.3 (139 to 243.1) | 175.7 (128.6 to 230.9) | 1226 (931.9 to 1508.3) | 674.9 (508.4 to 825.9) | 551.1 (409.1 to 699.9) | 237.7 (177.6 to 308.4) | 260.2 (172.9 to 358.6) | 213.6 (153.7 to 304.7) |
|  | Deaths | Age-standardized | 2.9 (1.8 to 3.7) | 3.7 (2.5 to 5.2) | 2 (1.2 to 2.8) | 3 (2.1 to 3.6) | 4.5 (2.9 to 5.6) | 1.8 (1.3 to 2.4) | 3.9 (-25.3 to 45.1) | 20.2 (-21.1 to 72.2) | -9.5 (-36.9 to 60.1) |
|  |  | All ages | 12 (7.8 to 15.6) | 7.4 (5 to 10.4) | 4.6 (2.6 to 6.3) | 53.6 (36.8 to 64.1) | 34.7 (22.5 to 43.2) | 18.9 (12.5 to 24.6) | 346.7 (220.5 to 528.2) | 368.7 (205.5 to 575) | 311.3 (178.5 to 623.2) |
| Hormozgan | Incidence | Age-standardized | 36.5 (26.8 to 49.2) | 33.3 (24.8 to 44.3) | 39.9 (29.1 to 53.4) | 40.3 (29.8 to 53.8) | 36.4 (26.9 to 48.9) | 44.3 (32.5 to 59.5) | 10.5 (4.9 to 15) | 9.4 (3 to 16) | 11.1 (2.8 to 18.2) |
|  |  | All ages | 120.6 (89.9 to 160.5) | 51.7 (38.1 to 68.3) | 68.9 (50.3 to 92.3) | 454.1 (350.1 to 591) | 206.7 (158.3 to 269.9) | 247.3 (188.7 to 321.2) | 276.5 (248.6 to 306.4) | 300 (271.4 to 329) | 258.9 (220.2 to 304.2) |
|  | Prevalence | Age-standardized | 373.9 (284.5 to 492.5) | 338.1 (258.3 to 442) | 413.2 (313.6 to 545.1) | 420.6 (323.4 to 550) | 375.4 (287.2 to 491.4) | 466.2 (357.6 to 609.9) | 12.5 (6.9 to 17) | 11 (4.4 to 18.4) | 12.8 (5.3 to 19.4) |
|  |  | All ages | 1130.7 (878 to 1489.4) | 486.3 (369.4 to 637.8) | 644.4 (502.7 to 853.4) | 4413.5 (3450.2 to 5720.2) | 1980.9 (1531.4 to 2590.7) | 2432.5 (1913.8 to 3107) | 290.3 (269.6 to 312.9) | 307.3 (281.6 to 332.9) | 277.5 (247.6 to 309) |
|  | DALYs (Disability-Adjusted Life Years) | Age-standardized | 68 (51.6 to 85.9) | 73.1 (54 to 94.9) | 62.3 (44.4 to 80.4) | 72.8 (56.5 to 89.3) | 80.4 (59.8 to 97.6) | 65.2 (48.3 to 83.2) | 7.1 (-13 to 31.9) | 10 (-15.7 to 41.7) | 4.6 (-12.3 to 40.2) |
|  |  | All ages | 186.4 (139.3 to 236.1) | 97.5 (71.9 to 126) | 88.9 (62.1 to 116.6) | 714.3 (553.1 to 878.7) | 387.7 (291.7 to 474) | 326.6 (241.5 to 415.4) | 283.2 (215.3 to 366) | 297.8 (206.7 to 407.1) | 267.2 (205.5 to 376.7) |
|  | Deaths | Age-standardized | 3.1 (2 to 3.9) | 3.6 (2.4 to 4.9) | 2.4 (1.4 to 3.2) | 3.3 (2.4 to 4) | 4.3 (2.9 to 5.1) | 2.4 (1.5 to 3.1) | 8 (-23 to 57.8) | 17.5 (-21.8 to 68.4) | -1.1 (-31.6 to 87.6) |
|  |  | All ages | 6.4 (4.2 to 8.1) | 4 (2.6 to 5.5) | 2.4 (1.4 to 3.1) | 28.3 (20.3 to 33.6) | 17.9 (12.2 to 21.5) | 10.4 (6.6 to 13.5) | 342.6 (217.8 to 548.8) | 343.2 (194.7 to 541.7) | 341.5 (213.4 to 726.8) |
| Ilam | Incidence | Age-standardized | 36 (26.5 to 48.2) | 32.6 (24 to 43.9) | 39 (28.7 to 52.3) | 39.3 (29.3 to 52.2) | 35.6 (26.1 to 47.9) | 43.3 (32.3 to 57.3) | 9.2 (4.1 to 14.3) | 9.2 (2 to 16) | 11.1 (4.6 to 18.6) |
|  |  | All ages | 50.7 (38.7 to 66.6) | 20 (15.2 to 26.3) | 30.7 (23.1 to 40.3) | 185.1 (140.3 to 242.6) | 87.5 (65 to 115.8) | 97.6 (74.2 to 126.3) | 265.4 (243.9 to 286.2) | 338 (310 to 365.4) | 218.1 (193.4 to 244.8) |
|  | Prevalence | Age-standardized | 370.9 (282.9 to 483.1) | 332.5 (253.8 to 437.3) | 404.8 (304.9 to 522.7) | 409.5 (310.3 to 536.3) | 367.4 (275.1 to 482.9) | 456.5 (347.7 to 594.7) | 10.4 (6 to 16.5) | 10.5 (3.7 to 17.8) | 12.8 (7 to 20.9) |
|  |  | All ages | 482.9 (370.9 to 635.1) | 188.4 (146.3 to 245.8) | 294.4 (226.1 to 384.8) | 1804.5 (1391 to 2341.8) | 846.9 (642.2 to 1105.5) | 957.7 (738.8 to 1232.2) | 273.7 (256 to 294.7) | 349.4 (318.8 to 378.1) | 225.3 (203.9 to 246.9) |
|  | DALYs (Disability-Adjusted Life Years) | Age-standardized | 58.6 (44.4 to 75.2) | 59.9 (45.2 to 78.4) | 57.4 (41.3 to 74.3) | 72.7 (58.1 to 88) | 76.7 (61.9 to 92.9) | 67.8 (51 to 84.7) | 24.1 (2.1 to 55.9) | 27.9 (-3.9 to 61.8) | 18 (-2.7 to 63.2) |
|  |  | All ages | 71.1 (53.3 to 91.2) | 32 (24.2 to 42) | 39.2 (28.3 to 51.2) | 288.9 (229 to 353.8) | 158.6 (126.4 to 194.7) | 130.3 (96.4 to 164.7) | 306.3 (237.9 to 403.4) | 396.2 (277.1 to 521.5) | 232.9 (178 to 340.7) |
|  | Deaths | Age-standardized | 2.4 (1.6 to 3.1) | 2.6 (1.9 to 3.7) | 2.1 (1.2 to 2.9) | 3.5 (2.7 to 4) | 4 (3.1 to 4.8) | 2.8 (1.8 to 3.6) | 45.7 (1 to 122.1) | 50.3 (-3.9 to 111.5) | 32.8 (-15.4 to 171.4) |
|  |  | All ages | 2.3 (1.6 to 3) | 1.2 (0.8 to 1.7) | 1.1 (0.6 to 1.5) | 11 (8.8 to 12.7) | 6.8 (5.3 to 8.1) | 4.2 (2.7 to 5.3) | 374.5 (233 to 621.1) | 466.4 (257.2 to 694.6) | 276.8 (147 to 662.2) |
| Isfahan | Incidence | Age-standardized | 37.9 (28.3 to 50.7) | 34.8 (25.4 to 46.1) | 41.6 (31.1 to 56.3) | 39.1 (29.2 to 52) | 35.1 (26.3 to 46.9) | 42.9 (32.1 to 56.9) | 3 (-1.4 to 8.6) | 0.9 (-6.1 to 8.1) | 2.9 (-4 to 10.4) |
|  |  | All ages | 583.7 (435.6 to 776.1) | 266.8 (196.8 to 358.6) | 316.9 (238.7 to 418.6) | 2008.7 (1544.2 to 2653.1) | 884.2 (674.9 to 1164.7) | 1124.5 (859.8 to 1469.1) | 244.2 (217.2 to 277.8) | 231.4 (205.4 to 263.6) | 254.9 (216.2 to 302.6) |
|  | Prevalence | Age-standardized | 393.4 (302.7 to 516.3) | 356.8 (270.7 to 473) | 435.7 (335.4 to 574.7) | 409.4 (318.7 to 536.3) | 362.7 (279.1 to 474.6) | 451.9 (348 to 586.8) | 4.1 (-0.5 to 9) | 1.6 (-5.8 to 9.2) | 3.7 (-2.8 to 10.8) |
|  |  | All ages | 5461.1 (4227.3 to 7175.4) | 2497.2 (1895.4 to 3303.2) | 2964 (2278.2 to 3884.7) | 20192.4 (15904.1 to 26297.1) | 8702.3 (6753.9 to 11381.9) | 11490.1 (9025.8 to 14917.7) | 269.7 (245 to 300.3) | 248.5 (222.5 to 276.8) | 287.7 (250.3 to 334.2) |
|  | DALYs (Disability-Adjusted Life Years) | Age-standardized | 66.5 (52.5 to 85.2) | 73.9 (56.8 to 93.9) | 57.6 (42.2 to 75.9) | 68.2 (52.4 to 84.2) | 81.7 (61.6 to 98.9) | 57.5 (41.8 to 74.6) | 2.5 (-14.4 to 20.8) | 10.6 (-14.9 to 37.2) | -0.1 (-16 to 24.5) |
|  |  | All ages | 794 (615 to 1017.3) | 436.7 (333.2 to 559.8) | 357.3 (256.5 to 480.9) | 3196.4 (2473.1 to 3976.3) | 1758.8 (1350.5 to 2136) | 1437.6 (1039.5 to 1870.5) | 302.6 (240 to 374.5) | 302.8 (216.5 to 398.6) | 302.4 (234.3 to 394.9) |
|  | Deaths | Age-standardized | 3 (2.2 to 3.9) | 3.9 (2.8 to 5.3) | 2 (1.2 to 2.7) | 3.1 (2.2 to 3.7) | 4.7 (3.3 to 5.8) | 1.8 (1.2 to 2.4) | 2 (-25.6 to 36.8) | 19.7 (-19.9 to 65.7) | -6.4 (-35.4 to 59.8) |
|  |  | All ages | 25.2 (18.4 to 32.4) | 17.3 (12.5 to 23.2) | 8 (5.3 to 10.7) | 130.8 (94 to 156.7) | 88 (62.5 to 109.4) | 42.8 (28.3 to 56.1) | 418.1 (279.4 to 594.3) | 409.7 (240.8 to 613.6) | 436.4 (267.6 to 813.4) |
| Kerman | Incidence | Age-standardized | 37.8 (27.9 to 50.3) | 34.6 (25.4 to 46.3) | 41.3 (30.6 to 55) | 41.2 (30.5 to 54.8) | 37.1 (27.5 to 49.6) | 45.3 (33.5 to 60.4) | 9 (3.8 to 14.6) | 7.3 (1.3 to 13.5) | 9.5 (1.7 to 18) |
|  |  | All ages | 268.9 (197.8 to 364.7) | 119 (87.9 to 159.4) | 149.9 (108.7 to 203.9) | 982.2 (747.3 to 1277.6) | 445.4 (336.9 to 586.3) | 536.7 (410.2 to 695.6) | 265.3 (234 to 300.2) | 274.5 (239.4 to 309.4) | 258 (218.5 to 303.6) |
|  | Prevalence | Age-standardized | 388 (296.7 to 510.9) | 352.7 (267.4 to 462.7) | 430 (328.7 to 564.3) | 429.9 (328.1 to 558) | 383.1 (290.2 to 498.7) | 476.8 (364.2 to 619.7) | 10.8 (5.9 to 16.5) | 8.6 (2.7 to 15.8) | 10.9 (3.5 to 19.5) |
|  |  | All ages | 2485.6 (1904.1 to 3266.4) | 1099.4 (836.6 to 1444.9) | 1386.2 (1076.3 to 1809.1) | 9606.4 (7418.8 to 12372.7) | 4307.5 (3322.8 to 5623.2) | 5299 (4102 to 6832.5) | 286.5 (260.7 to 317.9) | 291.8 (263.8 to 320.5) | 282.3 (246.2 to 320.2) |
|  | DALYs (Disability-Adjusted Life Years) | Age-standardized | 76.6 (58.6 to 96) | 83.2 (61.9 to 105.2) | 67.8 (49.3 to 87.4) | 83 (62.9 to 100.8) | 95.2 (71.3 to 114.5) | 70.7 (51.1 to 91.1) | 8.3 (-11.6 to 31.4) | 14.5 (-13.1 to 48.8) | 4.2 (-15 to 33.5) |
|  |  | All ages | 411.2 (316.9 to 518.2) | 219.6 (163.5 to 282) | 191.6 (135.8 to 253.7) | 1632.6 (1241.6 to 2009.2) | 906.8 (681 to 1107.3) | 725.8 (527 to 938.5) | 297.1 (232.1 to 373.5) | 312.9 (213.2 to 426.6) | 278.9 (208.2 to 373) |
|  | Deaths | Age-standardized | 3.8 (2.6 to 4.8) | 4.5 (3.2 to 6) | 2.8 (1.6 to 3.7) | 4.3 (3 to 5.2) | 5.8 (4 to 7.1) | 2.9 (1.8 to 3.6) | 13.8 (-15.9 to 53.5) | 27.5 (-11.6 to 79) | 3.5 (-24.2 to 68.9) |
|  |  | All ages | 13.5 (9.6 to 16.8) | 8.8 (6.2 to 11.5) | 4.7 (2.8 to 6.3) | 68.6 (48.6 to 82.5) | 44.9 (31 to 55.3) | 23.7 (14.7 to 29.7) | 409.6 (276 to 595.8) | 412.6 (248.4 to 637.8) | 404 (252 to 713.5) |
| Kermanshah | Incidence | Age-standardized | 36.2 (26.8 to 48.5) | 32.9 (24.1 to 44.5) | 39.2 (29.2 to 52.3) | 40.5 (29.9 to 54.6) | 36.5 (27 to 49.4) | 44.6 (33 to 60.5) | 11.9 (6.8 to 17.6) | 10.9 (4.4 to 17.5) | 13.8 (6.9 to 23.1) |
|  |  | All ages | 220.7 (164.3 to 293.4) | 89.6 (67 to 119.7) | 131.2 (97.3 to 174.4) | 767.2 (577.5 to 1026) | 352.8 (264.5 to 469.4) | 414.4 (312 to 552.5) | 247.6 (217.9 to 280.6) | 293.9 (258.9 to 331.8) | 215.9 (180.3 to 252.1) |
|  | Prevalence | Age-standardized | 372.1 (284.9 to 490.1) | 334.5 (254 to 441.1) | 406.9 (310.1 to 531.5) | 423.9 (324.5 to 562.4) | 376 (285.9 to 492.8) | 469.6 (355.7 to 628.3) | 13.9 (9.2 to 19.1) | 12.4 (4.5 to 19.6) | 15.4 (8.8 to 23.3) |
|  |  | All ages | 2044.6 (1588.5 to 2690) | 817.1 (627.9 to 1079.6) | 1227.4 (951.2 to 1614.9) | 7678.2 (5939.5 to 9972.4) | 3446 (2644.7 to 4521.8) | 4232.2 (3262 to 5563.8) | 275.5 (251.2 to 308.9) | 321.7 (287 to 355.5) | 244.8 (213.9 to 282.4) |
|  | DALYs (Disability-Adjusted Life Years) | Age-standardized | 72.5 (56.3 to 89.2) | 82.2 (60.2 to 103.1) | 63.3 (46.7 to 80.8) | 75.2 (57.6 to 92.9) | 89.1 (66.9 to 110.5) | 63.7 (47.7 to 82.6) | 3.7 (-15.1 to 22.6) | 8.4 (-20.1 to 38.4) | 0.7 (-20.2 to 24.3) |
|  |  | All ages | 351.4 (270.1 to 436.4) | 175.7 (129.6 to 221.7) | 175.7 (128.5 to 229.1) | 1298.5 (995.3 to 1616.8) | 730.4 (549.1 to 916) | 568.1 (424.3 to 730.3) | 269.5 (205.8 to 337.9) | 315.6 (206.5 to 427.5) | 223.4 (156 to 302.3) |
|  | Deaths | Age-standardized | 3.5 (2.4 to 4.3) | 4.5 (3 to 5.9) | 2.5 (1.6 to 3.3) | 3.5 (2.4 to 4.2) | 5.1 (3.5 to 6.2) | 2.2 (1.3 to 2.8) | -0.2 (-26.1 to 31.9) | 13 (-24.6 to 58.8) | -11.4 (-39.6 to 36.4) |
|  |  | All ages | 12.1 (8.4 to 15) | 7.3 (4.9 to 9.6) | 4.7 (3 to 6.5) | 54.2 (38.2 to 65.1) | 35.5 (24.7 to 44) | 18.7 (11.4 to 23.7) | 349.7 (232.4 to 492.1) | 384.1 (222.5 to 581) | 296.3 (161.7 to 511) |
| Khorasan-e-Razavi | Incidence | Age-standardized | 37.1 (27.1 to 49.9) | 33.8 (24.9 to 45.3) | 40.4 (29.5 to 54.3) | 40.1 (30.1 to 53.2) | 36.2 (27.1 to 48.6) | 44.2 (32.7 to 59) | 8.2 (3.8 to 12.6) | 7 (0.1 to 14.9) | 9.3 (3.4 to 16.9) |
|  |  | All ages | 660.7 (484.2 to 885.6) | 286.8 (212.6 to 381.6) | 373.9 (270.6 to 504.8) | 2102.5 (1612.8 to 2774.6) | 964.3 (732.7 to 1273.1) | 1138.1 (861.7 to 1501.1) | 218.2 (185.7 to 255.4) | 236.3 (202.3 to 271) | 204.4 (167.6 to 246.5) |
|  | Prevalence | Age-standardized | 381.3 (290 to 501.3) | 344.1 (261.8 to 450) | 419.6 (319.3 to 555.4) | 418.2 (320.4 to 549.3) | 372.6 (283.2 to 487.9) | 465.2 (357.4 to 607.5) | 9.7 (5.3 to 14.2) | 8.3 (1.2 to 16.4) | 10.9 (4.4 to 18.6) |
|  |  | All ages | 6079.1 (4701.3 to 7951.4) | 2608 (1986.4 to 3415.9) | 3471.1 (2673.3 to 4582.6) | 20908.4 (16145.8 to 26951.6) | 9474.2 (7288.2 to 12368.4) | 11434.2 (8731.8 to 14795.8) | 243.9 (217.9 to 279.7) | 263.3 (231.6 to 299.7) | 229.4 (196.7 to 263.8) |
|  | DALYs (Disability-Adjusted Life Years) | Age-standardized | 77.1 (58.3 to 96.4) | 86.9 (63.5 to 113.2) | 66.8 (48.4 to 85.7) | 77.2 (59.8 to 92.9) | 88.5 (66.8 to 106.6) | 65.6 (48 to 82.7) | 0.1 (-18.3 to 22.1) | 1.8 (-24.6 to 33.3) | -1.8 (-19.1 to 27.4) |
|  |  | All ages | 1025.7 (775.5 to 1300.6) | 543.2 (405.9 to 706) | 482.5 (353.5 to 630.5) | 3590.4 (2785.7 to 4338.4) | 2047.6 (1557.2 to 2485.2) | 1542.9 (1112.9 to 1948.4) | 250.1 (192.2 to 323.9) | 277 (185 to 392.8) | 219.8 (159.4 to 302.9) |
|  | Deaths | Age-standardized | 3.9 (2.5 to 5.1) | 4.9 (3.3 to 6.8) | 2.8 (1.7 to 3.7) | 3.8 (2.6 to 4.6) | 5.1 (3.4 to 6.2) | 2.5 (1.5 to 3.2) | -3.5 (-29 to 34) | 2.9 (-31.4 to 47.5) | -12.6 (-35.6 to 52.3) |
|  |  | All ages | 33.4 (22.7 to 43.3) | 21.2 (14.5 to 29.4) | 12.2 (7.7 to 16.3) | 152.5 (107 to 185.1) | 102.6 (69.8 to 127.2) | 49.9 (30.8 to 64.1) | 356.4 (232.5 to 529.3) | 384.3 (219.9 to 600.5) | 308.1 (189.3 to 597.7) |
| Khuzestan | Incidence | Age-standardized | 36.9 (27 to 49.5) | 33.9 (24.7 to 45.3) | 40.3 (29.8 to 54.5) | 41.1 (30.6 to 54.9) | 37.1 (27.7 to 48.8) | 45.2 (33.3 to 62.1) | 11.4 (6.8 to 16.8) | 9.5 (2.3 to 18.5) | 12.1 (4.9 to 20) |
|  |  | All ages | 408.3 (311.3 to 536.7) | 186 (139.1 to 247.2) | 222.3 (169.2 to 293.4) | 1415.9 (1086.2 to 1846.5) | 644.2 (485.7 to 847.3) | 771.7 (590.9 to 1007.2) | 246.8 (230.7 to 263.6) | 246.4 (224.6 to 273.3) | 247.1 (218.1 to 271.5) |
|  | Prevalence | Age-standardized | 380.3 (288.5 to 498) | 345.1 (259.4 to 453.9) | 419.7 (320.4 to 551.3) | 430.6 (328 to 564.4) | 383.2 (288.3 to 501.1) | 477.5 (365 to 628) | 13.2 (8.9 to 18.1) | 11.1 (4.1 to 19.4) | 13.8 (7.1 to 21.6) |
|  |  | All ages | 3926.6 (3019.4 to 5112.8) | 1790.5 (1341.3 to 2334.5) | 2136.1 (1665.7 to 2754) | 13990.7 (10669.2 to 18158.8) | 6270.2 (4780.2 to 8209.6) | 7720.5 (5940.2 to 9895.1) | 256.3 (239 to 271.9) | 250.2 (227.4 to 276.1) | 261.4 (233.5 to 286.2) |
|  | DALYs (Disability-Adjusted Life Years) | Age-standardized | 72.8 (56.9 to 91) | 82.6 (62 to 107.5) | 61.8 (45.5 to 80.7) | 75 (57.4 to 92.5) | 87.6 (65.2 to 107.9) | 63.3 (46 to 82.1) | 3 (-16.4 to 24.6) | 6.1 (-20.9 to 35.2) | 2.4 (-14.8 to 30.6) |
|  |  | All ages | 694.7 (545.6 to 873.9) | 398 (303 to 517.4) | 296.7 (218.2 to 388.4) | 2302.6 (1761.2 to 2838.2) | 1304.8 (977.7 to 1607.4) | 997.7 (728.2 to 1284.7) | 231.5 (170.9 to 297.5) | 227.8 (144 to 317.4) | 236.3 (179.7 to 325.9) |
|  | Deaths | Age-standardized | 3.4 (2.4 to 4.4) | 4.4 (2.9 to 5.9) | 2.4 (1.5 to 3.1) | 3.4 (2.4 to 4.1) | 4.8 (3.2 to 6) | 2.1 (1.4 to 2.7) | -2.2 (-28.4 to 33.6) | 8.6 (-27.9 to 49.5) | -10.6 (-35.7 to 51.9) |
|  |  | All ages | 27.3 (18.8 to 34.8) | 18.3 (12.4 to 24.7) | 8.9 (5.8 to 11.8) | 90.4 (63.7 to 109.8) | 61 (42.1 to 76.1) | 29.4 (19.1 to 37.9) | 231.7 (141.3 to 352.4) | 232.8 (120.7 to 364) | 229.3 (130.8 to 464.2) |
| Kohgiluyeh and Boyer-Ahmad | Incidence | Age-standardized | 35.9 (26.4 to 48.6) | 32.7 (24.2 to 44) | 39.2 (28.9 to 53.6) | 40.4 (29.9 to 53.8) | 36.1 (26.8 to 48.5) | 44.1 (32.7 to 58.7) | 12.3 (7.6 to 16.9) | 10.5 (4.4 to 17.8) | 12.7 (6.3 to 20.5) |
|  |  | All ages | 49.4 (37.5 to 65.3) | 21.1 (15.9 to 27.8) | 28.3 (21.5 to 37.8) | 214.2 (162.7 to 281.4) | 91.3 (69.4 to 121.4) | 122.9 (92.9 to 161.6) | 333.3 (288.1 to 387.9) | 332 (298.2 to 380.1) | 334.2 (278.5 to 404.4) |
|  | Prevalence | Age-standardized | 371.1 (283.9 to 492.5) | 333.9 (255.3 to 441.4) | 407.4 (312.1 to 543.3) | 424.3 (324.4 to 554) | 374.4 (284.4 to 493.3) | 466.2 (356.6 to 605.9) | 14.3 (9.2 to 18.4) | 12.1 (5.8 to 19) | 14.4 (7.6 to 21.6) |
|  |  | All ages | 457.5 (356.4 to 604.8) | 194 (148.4 to 258.5) | 263.5 (204.3 to 345.8) | 2175 (1676.8 to 2828.6) | 899.2 (692.8 to 1178.7) | 1275.8 (980.9 to 1651.1) | 375.4 (338.1 to 420.5) | 363.4 (327.7 to 400.5) | 384.2 (333.5 to 445.2) |
|  | DALYs (Disability-Adjusted Life Years) | Age-standardized | 58.8 (44.9 to 77.5) | 65.7 (47.9 to 89) | 51.7 (36.8 to 69.4) | 65.1 (49.7 to 80.4) | 77.2 (59.5 to 93.7) | 56.6 (41.4 to 73.4) | 10.8 (-11.1 to 35.6) | 17.5 (-15.2 to 52.2) | 9.5 (-9.4 to 42.2) |
|  |  | All ages | 63.7 (47.6 to 85.3) | 33 (24 to 44.9) | 30.7 (21.7 to 41.7) | 325.3 (249.3 to 403.3) | 170.6 (133.6 to 206.6) | 154.7 (113.6 to 198.9) | 410.7 (317.7 to 525.5) | 417.6 (280.5 to 564.3) | 403.3 (310.5 to 551.3) |
|  | Deaths | Age-standardized | 2.5 (1.6 to 3.4) | 3.3 (2.2 to 4.8) | 1.7 (0.9 to 2.4) | 2.7 (2 to 3.3) | 4.2 (2.9 to 5.2) | 1.7 (1.1 to 2.2) | 8.1 (-27.1 to 61.7) | 25.6 (-23.1 to 87.3) | 2.5 (-31.2 to 99) |
|  |  | All ages | 1.8 (1.2 to 2.5) | 1.2 (0.8 to 1.7) | 0.6 (0.3 to 0.9) | 12.6 (9.2 to 15.4) | 8.1 (5.6 to 10) | 4.6 (3 to 6) | 591.7 (362.1 to 946.5) | 568.4 (307.2 to 896.7) | 637 (381.6 to 1349.2) |
| Kurdistan | Incidence | Age-standardized | 36.5 (27.1 to 49.3) | 33.2 (24.7 to 44.6) | 39.5 (29.1 to 53.6) | 40.3 (29.9 to 54.6) | 36.4 (27.1 to 49.7) | 44.2 (32.6 to 59.3) | 10.3 (5.4 to 15) | 9.8 (2.9 to 16.7) | 11.9 (4.6 to 20.1) |
|  |  | All ages | 169.3 (126 to 224.5) | 69.3 (52.6 to 91.2) | 100 (73.2 to 134) | 600.1 (453.7 to 793.9) | 274.3 (206.9 to 369.5) | 325.8 (245.6 to 425.8) | 254.4 (226.3 to 285) | 295.8 (259.9 to 333.5) | 225.7 (193 to 264.5) |
|  | Prevalence | Age-standardized | 375.2 (288.5 to 492.8) | 337.9 (256.3 to 446.2) | 410 (311.7 to 542.2) | 421.6 (322.9 to 553.5) | 376 (283.2 to 499.7) | 466.6 (358.1 to 606.8) | 12.4 (8.1 to 17.2) | 11.3 (5.1 to 18.4) | 13.8 (7 to 21.7) |
|  |  | All ages | 1585.4 (1218.4 to 2077.4) | 639.9 (490 to 855.8) | 945.5 (728.7 to 1230.2) | 6039.4 (4649.4 to 7887.3) | 2711.3 (2056.1 to 3578.7) | 3328.1 (2562.7 to 4287.6) | 280.9 (254 to 307.7) | 323.7 (289.9 to 359.9) | 252 (222.6 to 284.7) |
|  | DALYs (Disability-Adjusted Life Years) | Age-standardized | 66.1 (51.1 to 84.1) | 70.4 (52.1 to 92.1) | 62.3 (45.2 to 80.3) | 71 (54.1 to 88.6) | 79.7 (59.5 to 98.5) | 63.1 (46.5 to 81.2) | 7.3 (-13 to 27.7) | 13.2 (-13.6 to 43.9) | 1.3 (-20 to 28.7) |
|  |  | All ages | 252.8 (192.5 to 322.4) | 121.9 (91.1 to 159.4) | 130.9 (93.6 to 171.4) | 981.4 (754.3 to 1228.8) | 536.4 (400.8 to 666.8) | 445 (329.5 to 580.2) | 288.2 (216 to 363.7) | 340.1 (234 to 458) | 239.9 (172.1 to 323.8) |
|  | Deaths | Age-standardized | 3 (2 to 3.8) | 3.5 (2.3 to 4.9) | 2.5 (1.5 to 3.4) | 3.2 (2.1 to 3.8) | 4.3 (3 to 5.3) | 2.2 (1.4 to 2.9) | 7.2 (-24.4 to 46.5) | 23.1 (-18 to 79.1) | -11 (-41.3 to 49.5) |
|  |  | All ages | 8.5 (5.8 to 11) | 4.9 (3.2 to 6.8) | 3.6 (2.2 to 5.1) | 40.8 (27.7 to 49.6) | 26 (18.3 to 32.1) | 14.8 (9.2 to 19.7) | 381.2 (241.1 to 560.6) | 433.9 (254.1 to 679.8) | 310.2 (158.4 to 587.1) |
| Lorestan | Incidence | Age-standardized | 36.3 (26.9 to 48.2) | 32.9 (24.1 to 44.8) | 39.4 (29.5 to 52.2) | 39.2 (28.9 to 52.6) | 35.7 (26.5 to 47.5) | 43.6 (31.8 to 58.7) | 8.1 (3.5 to 15) | 8.4 (1.5 to 16.9) | 10.7 (3.8 to 20.2) |
|  |  | All ages | 192.5 (145.6 to 257) | 79.2 (58.9 to 105.4) | 113.3 (84.8 to 152.3) | 561 (424.9 to 736.6) | 278.4 (210 to 364.7) | 282.6 (213.8 to 372.9) | 191.5 (171.2 to 212) | 251.5 (224.5 to 281.3) | 149.5 (125.3 to 174.6) |
|  | Prevalence | Age-standardized | 374.5 (287.6 to 493.4) | 336.4 (255 to 444.5) | 410.3 (315.5 to 543.7) | 410.1 (313.3 to 535.9) | 369.9 (280.8 to 484.7) | 460.5 (352.5 to 607) | 9.5 (5.1 to 15.9) | 9.9 (3.3 to 18.8) | 12.3 (5.5 to 19.9) |
|  |  | All ages | 1807.5 (1389.3 to 2363.8) | 734.8 (559.7 to 970.3) | 1072.7 (833.3 to 1410.4) | 5534.4 (4280.8 to 7205.7) | 2748.2 (2089.4 to 3594.6) | 2786.2 (2146.2 to 3596.5) | 206.2 (187.3 to 227.4) | 274 (242.2 to 304.4) | 159.7 (139 to 182.8) |
|  | DALYs (Disability-Adjusted Life Years) | Age-standardized | 66.4 (51 to 83.7) | 71.3 (53 to 92.9) | 61.8 (42.9 to 80.5) | 71.3 (55 to 88) | 73.8 (55 to 91.9) | 67.6 (49.4 to 84.5) | 7.4 (-13.2 to 35.5) | 3.5 (-23.7 to 31.5) | 9.3 (-9.2 to 49.9) |
|  |  | All ages | 288.8 (218.9 to 368.2) | 141.3 (106.4 to 186.4) | 147.5 (105.7 to 191.7) | 890.6 (678.8 to 1090.9) | 504.7 (377.3 to 630.9) | 385.9 (285.4 to 482.5) | 208.3 (151.9 to 283.5) | 257.2 (159.1 to 350.5) | 161.5 (118.2 to 248.5) |
|  | Deaths | Age-standardized | 3 (2 to 3.8) | 3.5 (2.4 to 4.8) | 2.4 (1.3 to 3.2) | 3.3 (2.4 to 3.9) | 3.8 (2.7 to 4.6) | 2.5 (1.5 to 3.2) | 10 (-20.5 to 63.8) | 6.5 (-31.5 to 52.4) | 6 (-26.8 to 105.6) |
|  |  | All ages | 9.6 (6.5 to 12.6) | 5.6 (3.9 to 7.6) | 4 (2.3 to 5.4) | 34.1 (25.1 to 40.6) | 22.2 (15.5 to 27.2) | 11.9 (7.2 to 15.1) | 255.4 (155.6 to 424.9) | 300.2 (155.3 to 470.2) | 193.7 (105.2 to 465.8) |
| Markazi | Incidence | Age-standardized | 36.7 (26.9 to 49.1) | 33.5 (24.6 to 44.6) | 39.9 (29.2 to 53.4) | 40.3 (29.9 to 54) | 36.3 (26.7 to 48.5) | 44.5 (33 to 60) | 9.7 (4.7 to 14.8) | 8.5 (1.2 to 16.1) | 11.4 (3.8 to 18.9) |
|  |  | All ages | 185.9 (138.1 to 244.4) | 82 (60.5 to 109.4) | 103.8 (77.7 to 136.6) | 621 (470.2 to 823.7) | 284.3 (212.3 to 376.7) | 336.7 (255 to 446.8) | 234.1 (194.4 to 277.1) | 246.6 (206.9 to 295.8) | 224.3 (182 to 269.5) |
|  | Prevalence | Age-standardized | 378.8 (288 to 492.6) | 341.7 (257.8 to 445.9) | 415.2 (317 to 541.3) | 422.4 (324.5 to 558.4) | 375.5 (285.4 to 495.9) | 469.7 (357.1 to 626.8) | 11.5 (6.7 to 15.3) | 9.9 (2.8 to 17.3) | 13.1 (6.2 to 20.3) |
|  |  | All ages | 1743 (1344.6 to 2266.1) | 753.3 (577.3 to 992.9) | 989.7 (765.1 to 1277.3) | 6522.8 (5041.5 to 8556.8) | 2926 (2235.4 to 3870.1) | 3596.7 (2743.2 to 4757.8) | 274.2 (243.5 to 314.2) | 288.4 (255.1 to 332) | 263.4 (228.5 to 309.6) |
|  | DALYs (Disability-Adjusted Life Years) | Age-standardized | 69.7 (54.1 to 86.1) | 80 (59.9 to 100.5) | 59.9 (44.7 to 75.9) | 67.7 (52.3 to 84.3) | 78.7 (60.9 to 97.9) | 57.7 (43.5 to 75.7) | -2.8 (-20.6 to 13.9) | -1.6 (-25.2 to 22.8) | -3.6 (-20.6 to 17.7) |
|  |  | All ages | 285.7 (221 to 360.6) | 151.4 (113.4 to 189.7) | 134.2 (98.6 to 172.3) | 1063.2 (825.5 to 1316.5) | 610.2 (474 to 755.3) | 453 (344 to 590.6) | 272.2 (206.3 to 336.7) | 302.9 (209.1 to 397.6) | 237.5 (175.9 to 314.7) |
|  | Deaths | Age-standardized | 3.3 (2.3 to 4.2) | 4.5 (3.1 to 5.9) | 2.3 (1.4 to 3) | 3 (2.2 to 3.5) | 4.3 (3 to 5.3) | 1.8 (1.1 to 2.2) | -11.4 (-35.2 to 18.5) | -3.8 (-34.5 to 31.8) | -22.3 (-44.5 to 25) |
|  |  | All ages | 10.3 (7.3 to 13) | 6.5 (4.6 to 8.7) | 3.8 (2.3 to 5) | 48.3 (35.3 to 56.5) | 33.1 (23.3 to 40.5) | 15.1 (9.6 to 19.2) | 368.3 (246.3 to 521.4) | 409.4 (247.4 to 609.5) | 297.8 (174.6 to 543.1) |
| Mazandaran | Incidence | Age-standardized | 37.2 (27.5 to 49.6) | 34.1 (25.1 to 45.2) | 40.8 (29.9 to 55.6) | 41.8 (31.2 to 56.1) | 37.6 (28 to 50.5) | 46 (34 to 62.1) | 12.4 (7 to 18.2) | 10.3 (2.9 to 19.5) | 12.7 (3.8 to 20.6) |
|  |  | All ages | 371.4 (276.1 to 492.8) | 170.6 (127.7 to 226.4) | 200.8 (147.4 to 267.3) | 1527.4 (1166.4 to 2027.6) | 699.5 (524.9 to 923.8) | 827.9 (630.8 to 1101.9) | 311.2 (277.8 to 352.2) | 309.9 (275.4 to 344.1) | 312.3 (269.9 to 362.7) |
|  | Prevalence | Age-standardized | 385 (293.9 to 507.5) | 350.5 (261.3 to 460.2) | 426.7 (324.8 to 567.4) | 439.5 (335.2 to 578.2) | 390.7 (294.2 to 512.3) | 488.7 (372.1 to 647.2) | 14.2 (8.2 to 19.9) | 11.5 (3.8 to 19.7) | 14.5 (6 to 22.3) |
|  |  | All ages | 3464.3 (2668.4 to 4566.5) | 1589.5 (1196.9 to 2097.1) | 1874.8 (1452.2 to 2474.3) | 15410.1 (11957.9 to 20069.4) | 6939 (5292.6 to 9042.9) | 8471.1 (6532.8 to 11006) | 344.8 (311.4 to 386.5) | 336.5 (300.5 to 375.2) | 351.8 (306.8 to 404.9) |
|  | DALYs (Disability-Adjusted Life Years) | Age-standardized | 68.6 (53.6 to 87) | 76.4 (57.8 to 98.5) | 58.7 (43.7 to 76.7) | 71.9 (55.5 to 89.6) | 81.7 (62.7 to 102.3) | 62.2 (46 to 80.5) | 4.8 (-14.9 to 26.2) | 6.9 (-20.5 to 36.5) | 6 (-10.5 to 30.9) |
|  |  | All ages | 542.3 (417.9 to 685.8) | 307.6 (233.2 to 394.5) | 234.7 (170.9 to 312.5) | 2427.8 (1875.9 to 3019.2) | 1370 (1058.9 to 1707.3) | 1057.8 (778.5 to 1366.1) | 347.7 (272.1 to 434.3) | 345.4 (237.2 to 470.6) | 350.7 (280.9 to 452.6) |
|  | Deaths | Age-standardized | 3.2 (2.4 to 4.2) | 4 (3 to 5.5) | 2.1 (1.4 to 2.8) | 3.1 (2.3 to 3.9) | 4.3 (3 to 5.5) | 2 (1.4 to 2.5) | -1.9 (-30.1 to 36.1) | 6.8 (-31.2 to 53.9) | -5.3 (-33.4 to 50.6) |
|  |  | All ages | 18.5 (13.9 to 24.1) | 12.9 (9.5 to 17.7) | 5.6 (3.6 to 7.4) | 96.9 (72.5 to 120.5) | 65.8 (47.2 to 85.3) | 31.1 (22.2 to 39) | 424.5 (274.4 to 631.7) | 409.8 (225.8 to 642.9) | 458.7 (285.8 to 791.8) |
| North Khorasan | Incidence | Age-standardized | 36.1 (26.8 to 48) | 33 (24.2 to 44.5) | 39.3 (29.4 to 52) | 41 (30.3 to 54.8) | 36.8 (27.3 to 49.4) | 45 (33 to 60.2) | 13.5 (8.5 to 19.2) | 11.6 (4.4 to 20.5) | 14.4 (8.2 to 23.2) |
|  |  | All ages | 79.4 (59.1 to 105.9) | 34.1 (25.5 to 45.3) | 45.3 (33.9 to 60.5) | 284.1 (217.6 to 374.4) | 126.4 (96.2 to 166) | 157.8 (118.6 to 206.9) | 257.8 (217.7 to 302.3) | 270.9 (235.5 to 315.6) | 248 (200.8 to 303.8) |
|  | Prevalence | Age-standardized | 370.6 (282.6 to 489.1) | 334.6 (255.4 to 438.8) | 407.5 (309.6 to 536.6) | 428.3 (326.9 to 565.1) | 378.6 (290.3 to 497.2) | 472.8 (358 to 620.9) | 15.6 (9.8 to 20.8) | 13.1 (5.5 to 21.2) | 16 (9.6 to 23.7) |
|  |  | All ages | 721 (558.8 to 946.9) | 304.1 (234 to 405.3) | 416.9 (321.3 to 546.9) | 2860.3 (2202.5 to 3698.6) | 1230.1 (958.7 to 1605) | 1630.2 (1252.4 to 2124.4) | 296.7 (260.4 to 337) | 304.5 (271.2 to 348.3) | 291 (249.3 to 344.4) |
|  | DALYs (Disability-Adjusted Life Years) | Age-standardized | 69.6 (53.4 to 87.2) | 78.9 (58.2 to 102.2) | 60 (43.7 to 77.7) | 72.7 (56.5 to 89.6) | 85.9 (65.3 to 105.2) | 61.7 (45.4 to 81) | 4.4 (-14.6 to 30.2) | 8.9 (-19.5 to 43.2) | 2.9 (-15.9 to 34.4) |
|  |  | All ages | 117.8 (90.2 to 151.1) | 61.8 (46 to 81) | 55.9 (40.2 to 73.2) | 473.1 (366.3 to 581.4) | 260.6 (200.6 to 322.7) | 212.4 (157.3 to 277.6) | 301.7 (231.2 to 400.7) | 321.4 (212.8 to 461.4) | 279.8 (204.5 to 387.6) |
|  | Deaths | Age-standardized | 3.2 (2.3 to 4.2) | 4.1 (3 to 5.7) | 2.2 (1.4 to 3) | 3.1 (2.2 to 3.8) | 4.5 (3.1 to 5.7) | 2 (1.3 to 2.5) | -4.6 (-32 to 38.1) | 8.9 (-30.1 to 59.5) | -12.6 (-38.9 to 58.6) |
|  |  | All ages | 3.5 (2.6 to 4.6) | 2.2 (1.6 to 3.1) | 1.3 (0.8 to 1.7) | 18.7 (13.6 to 23) | 12.1 (8.5 to 15.4) | 6.7 (4.6 to 8.5) | 433.4 (282.9 to 677.1) | 438.1 (247.3 to 700.6) | 425.1 (255.1 to 846.2) |
| Qazvin | Incidence | Age-standardized | 35.5 (26 to 47.7) | 32.5 (23.8 to 43.4) | 38.9 (28.5 to 51.7) | 38.9 (28.7 to 52.6) | 35.2 (26 to 47.2) | 43 (31.5 to 58.7) | 9.7 (5 to 14.1) | 8.3 (1.1 to 15) | 10.4 (2.9 to 18.1) |
|  |  | All ages | 127 (94.3 to 167.9) | 57.1 (42.5 to 75.6) | 69.9 (51.3 to 91.9) | 428.9 (329.7 to 568.9) | 201.1 (151.5 to 266.1) | 227.8 (174.6 to 302.2) | 237.6 (210.3 to 264.4) | 251.9 (219.7 to 279.6) | 226 (188.5 to 262.3) |
|  | Prevalence | Age-standardized | 363.2 (277.9 to 473.3) | 330.7 (252.4 to 434.9) | 403.9 (308.5 to 520.7) | 404.9 (308.2 to 534.3) | 363.2 (275.2 to 483.3) | 452.6 (344.4 to 596.7) | 11.5 (7 to 16.1) | 9.8 (3.6 to 16.5) | 12.1 (4.8 to 19.9) |
|  |  | All ages | 1184.7 (918 to 1542) | 534.9 (411.3 to 699.1) | 649.8 (503.9 to 843.6) | 4232.8 (3280.4 to 5539.4) | 1983.6 (1500.4 to 2622.8) | 2249.2 (1729.4 to 2942) | 257.3 (237.2 to 285) | 270.8 (246.1 to 298.5) | 246.1 (218.2 to 276.6) |
|  | DALYs (Disability-Adjusted Life Years) | Age-standardized | 67.5 (51.7 to 85.6) | 71.6 (53.9 to 92.6) | 62 (43.9 to 80) | 71.4 (54.9 to 89.4) | 73.5 (56 to 91) | 68 (50.4 to 85.6) | 5.9 (-13.3 to 31.7) | 2.7 (-21.1 to 29.7) | 9.6 (-10.9 to 47.2) |
|  |  | All ages | 193.8 (149.2 to 245) | 106 (80.4 to 138.3) | 87.8 (63.1 to 114.9) | 695.4 (536.8 to 865) | 378.8 (288.3 to 468.8) | 316.6 (233.8 to 399) | 258.8 (197.5 to 342) | 257.3 (176 to 348.8) | 260.6 (196.8 to 374.2) |
|  | Deaths | Age-standardized | 3.2 (2.1 to 4.2) | 3.7 (2.5 to 5) | 2.5 (1.5 to 3.5) | 3.4 (2.4 to 4.1) | 3.9 (2.7 to 4.8) | 2.7 (1.7 to 3.5) | 6.3 (-23.9 to 54.7) | 6.4 (-30.3 to 51.9) | 7.3 (-25.8 to 98.6) |
|  |  | All ages | 6.7 (4.5 to 8.7) | 4.5 (3.1 to 6.1) | 2.2 (1.3 to 3.1) | 28.7 (20.6 to 34.9) | 18.3 (13 to 22.6) | 10.3 (6.4 to 13.1) | 325.2 (208.1 to 525.6) | 306 (170.8 to 481.4) | 364.1 (223.3 to 753.7) |
| Qom | Incidence | Age-standardized | 35.7 (26.3 to 48) | 32.6 (23.7 to 43.9) | 38.9 (29 to 52.9) | 39.4 (29.4 to 52.7) | 35.4 (26.1 to 47.4) | 43.2 (32.3 to 57.9) | 10.4 (5.4 to 16.8) | 8.7 (2 to 16.2) | 11 (3.8 to 19.9) |
|  |  | All ages | 89.8 (66.7 to 119.2) | 39.3 (29.2 to 52.9) | 50.5 (37.8 to 66.4) | 407.2 (312.8 to 533.9) | 176.7 (134.1 to 233.5) | 230.5 (177.2 to 301.7) | 353.2 (316.8 to 394.8) | 349.6 (312.5 to 389.6) | 356 (308.9 to 405.4) |
|  | Prevalence | Age-standardized | 369.4 (281.5 to 486.1) | 332 (252.1 to 441.8) | 405 (309.4 to 531) | 415.1 (320.2 to 541.4) | 366.2 (279.7 to 482.1) | 456.2 (349.6 to 595.9) | 12.4 (7.6 to 17.9) | 10.3 (2.9 to 17.2) | 12.6 (5.5 to 20.6) |
|  |  | All ages | 835.8 (654.5 to 1089.5) | 359.9 (275.5 to 479.2) | 475.9 (372.2 to 614) | 4020.3 (3129.6 to 5231.8) | 1702.6 (1302.2 to 2238.7) | 2317.7 (1803 to 2959) | 381 (350.9 to 418.9) | 373.1 (338.3 to 410.7) | 387.1 (349.9 to 433.6) |
|  | DALYs (Disability-Adjusted Life Years) | Age-standardized | 76.8 (59 to 95.1) | 94.8 (68.3 to 120.5) | 61.6 (45.2 to 79.7) | 72.7 (54.6 to 88.7) | 98.1 (70.8 to 117.2) | 58.6 (43.2 to 76.3) | -5.4 (-24.3 to 12.1) | 3.4 (-21.5 to 30.7) | -4.8 (-22 to 17.4) |
|  |  | All ages | 148.4 (114 to 185.5) | 81.7 (59.9 to 104.5) | 66.7 (49 to 88.4) | 651.9 (495.9 to 797.7) | 362.1 (268.4 to 436.7) | 289.9 (212 to 374.7) | 339.2 (258.8 to 412.9) | 343.3 (236.6 to 456.1) | 334.3 (256.4 to 430.7) |
|  | Deaths | Age-standardized | 4.1 (2.7 to 5.2) | 6 (3.9 to 8) | 2.5 (1.6 to 3.3) | 3.5 (2.4 to 4.2) | 6.4 (4 to 7.7) | 2 (1.2 to 2.5) | -14.1 (-37 to 13.1) | 7 (-25 to 47.2) | -21.8 (-44.7 to 21.1) |
|  |  | All ages | 5.8 (4.2 to 7.3) | 3.8 (2.5 to 5) | 2 (1.3 to 2.7) | 27.2 (18.9 to 32.4) | 18.5 (12.1 to 22.6) | 8.8 (5.6 to 11) | 368 (240.8 to 514.8) | 384.1 (233.1 to 573.5) | 337.4 (205.8 to 584.8) |
| Semnan | Incidence | Age-standardized | 36.2 (26.4 to 48.5) | 33.2 (24.3 to 44.2) | 39.8 (29.1 to 53.1) | 39.8 (29.4 to 53.1) | 35.9 (26.1 to 48.2) | 43.9 (32.4 to 58.9) | 10 (3.8 to 15.6) | 8.4 (0.4 to 16.2) | 10.2 (2.2 to 17.1) |
|  |  | All ages | 84.8 (60.8 to 113.8) | 39.4 (28.8 to 52.6) | 45.4 (32.4 to 60.7) | 267.2 (203.4 to 355) | 124.6 (92.2 to 165.4) | 142.5 (107.9 to 188.2) | 215.1 (185.3 to 247.7) | 216.1 (188 to 246.9) | 214.2 (174.5 to 251) |
|  | Prevalence | Age-standardized | 371.2 (281.5 to 483.5) | 338.1 (254.6 to 445.7) | 414.5 (314.7 to 540.6) | 414.7 (318.4 to 545.3) | 372.2 (283.5 to 491) | 463.5 (352.8 to 605.7) | 11.7 (6.3 to 17.5) | 10.1 (3.3 to 17.6) | 11.8 (3.8 to 19.1) |
|  |  | All ages | 798.3 (613.1 to 1054.3) | 373.4 (286.3 to 495.6) | 424.9 (323.7 to 556) | 2667.5 (2068.7 to 3458.5) | 1248.3 (954.6 to 1638) | 1419.2 (1098.2 to 1844.1) | 234.2 (213.1 to 264.2) | 234.3 (212.2 to 261.9) | 234 (203.6 to 267.8) |
|  | DALYs (Disability-Adjusted Life Years) | Age-standardized | 68.5 (54.6 to 84.2) | 71.6 (55.7 to 88.8) | 64.4 (46.5 to 80.4) | 68.7 (53.2 to 85.7) | 72.5 (56.2 to 90.1) | 63.1 (46.5 to 80.2) | 0.2 (-16.4 to 18.3) | 1.2 (-20.7 to 24.7) | -2 (-17.7 to 26.7) |
|  |  | All ages | 130.1 (100.6 to 162.3) | 72.1 (55.9 to 90) | 58 (41.4 to 74.9) | 415.9 (322.3 to 520.4) | 233.4 (181.5 to 290.3) | 182.5 (134 to 235) | 219.6 (169.1 to 279.2) | 223.6 (154.4 to 298.1) | 214.7 (164.3 to 292.2) |
|  | Deaths | Age-standardized | 3.2 (2.4 to 4) | 3.6 (2.7 to 4.6) | 2.7 (1.7 to 3.5) | 3.1 (2.3 to 3.8) | 3.8 (2.7 to 4.7) | 2.3 (1.5 to 2.9) | -3.2 (-28.7 to 28.4) | 3.5 (-28.7 to 42.4) | -14.7 (-39.8 to 47.5) |
|  |  | All ages | 4.6 (3.5 to 5.6) | 3 (2.2 to 3.9) | 1.5 (1 to 2) | 16.8 (12.5 to 20.2) | 11.4 (8.1 to 14.3) | 5.4 (3.6 to 6.8) | 266.4 (169 to 385.7) | 275.2 (156.3 to 416.9) | 249.1 (147.4 to 489) |
| Sistan and Baluchistan | Incidence | Age-standardized | 36.2 (26.7 to 49.1) | 33 (24.6 to 44) | 39.2 (29 to 53.9) | 38.7 (28.2 to 51.9) | 34.9 (25.8 to 46.5) | 42.3 (31 to 56.7) | 7 (1.5 to 11.7) | 5.6 (-0.7 to 12.8) | 7.9 (-0.1 to 15.3) |
|  |  | All ages | 166.2 (124.2 to 222.3) | 67.4 (50.1 to 88.9) | 98.8 (73 to 133.3) | 541.1 (412.1 to 715.5) | 239.8 (183.6 to 310.7) | 301.4 (227.4 to 393.4) | 225.6 (195.8 to 268.6) | 255.8 (226.3 to 289.1) | 205.1 (171 to 256.5) |
|  | Prevalence | Age-standardized | 366.8 (282 to 484.7) | 331.4 (252.6 to 435.9) | 403.7 (305.7 to 537.6) | 398.7 (306.5 to 522.2) | 353.9 (270 to 462.6) | 440.7 (337.3 to 577.7) | 8.7 (3.6 to 13.9) | 6.8 (-0.2 to 13.6) | 9.2 (2.2 to 16.6) |
|  |  | All ages | 1521.6 (1181 to 2000.3) | 615.2 (472.7 to 806.7) | 906.5 (704.7 to 1200.7) | 5330.7 (4143.3 to 6913.7) | 2299.2 (1771.1 to 2970.6) | 3031.6 (2360 to 3949.4) | 250.3 (221.8 to 285.5) | 273.7 (245.9 to 300.7) | 234.4 (202.1 to 279.8) |
|  | DALYs (Disability-Adjusted Life Years) | Age-standardized | 65.1 (49.7 to 82) | 71.6 (51.5 to 91.8) | 58.3 (42.3 to 75.7) | 68.7 (50.9 to 85.5) | 77.7 (53.5 to 97.6) | 60.5 (43 to 76.9) | 5.6 (-12.1 to 27.5) | 8.5 (-18.4 to 40.4) | 3.8 (-15 to 33.1) |
|  |  | All ages | 240.6 (183.3 to 307.3) | 120.8 (87.8 to 155.4) | 119.8 (86.3 to 159.9) | 912.4 (678.6 to 1130.6) | 496.3 (347.2 to 623.9) | 416.1 (299.4 to 525.9) | 279.2 (216.2 to 363.5) | 310.9 (210.9 to 435.5) | 247.2 (180.5 to 346.3) |
|  | Deaths | Age-standardized | 2.8 (1.8 to 3.6) | 3.5 (2.3 to 4.7) | 2.1 (1.2 to 2.8) | 2.8 (1.7 to 3.4) | 3.7 (2.3 to 4.8) | 2 (1.1 to 2.6) | -2 (-28.4 to 35.8) | 6.7 (-29.6 to 53.7) | -7.2 (-35.1 to 58.4) |
|  |  | All ages | 7.1 (4.8 to 9) | 4.5 (3 to 6.1) | 2.6 (1.6 to 3.7) | 35.9 (22.6 to 44.4) | 22.6 (14.3 to 29.5) | 13.2 (7.4 to 17.5) | 404.2 (267.7 to 603.7) | 405.2 (234.2 to 644.1) | 402.3 (238 to 751.4) |
| South Khorasan | Incidence | Age-standardized | 35.4 (26.1 to 47.5) | 32.2 (23.8 to 43.4) | 38.6 (28.4 to 52.7) | 38.4 (28.2 to 51) | 34.6 (25.7 to 46.3) | 42.3 (30.8 to 56) | 8.4 (4.4 to 13.6) | 7.4 (0.4 to 14) | 9.8 (3.3 to 16.5) |
|  |  | All ages | 105.6 (78.3 to 140) | 45.5 (34 to 61.2) | 60.2 (44.2 to 79.6) | 285.1 (212.6 to 374.4) | 132.6 (100 to 175.6) | 152.5 (112.4 to 200.1) | 170 (136.9 to 201.6) | 191.7 (162.8 to 227.9) | 153.6 (115.1 to 190.3) |
|  | Prevalence | Age-standardized | 363.7 (276 to 478.8) | 327.4 (248.4 to 432.9) | 399.2 (304.2 to 527.3) | 398.4 (303.8 to 520.4) | 356.4 (272.3 to 464.2) | 444.1 (338.2 to 584.9) | 9.5 (5.2 to 14.5) | 8.9 (2.7 to 15) | 11.2 (4.9 to 17.9) |
|  |  | All ages | 990.9 (761.5 to 1300.9) | 421.3 (321.2 to 557.9) | 569.6 (437.3 to 750) | 2938.5 (2241.8 to 3824.5) | 1354 (1044.8 to 1759.6) | 1584.6 (1206.2 to 2078.2) | 196.6 (170 to 225.1) | 221.4 (196 to 253.7) | 178.2 (149 to 208.4) |
|  | DALYs (Disability-Adjusted Life Years) | Age-standardized | 60.7 (46.3 to 77.3) | 66.3 (48 to 86.3) | 55.3 (40 to 72.3) | 64.4 (49.9 to 80.4) | 70.2 (54.2 to 86.3) | 58.2 (43.7 to 75.3) | 6.1 (-13.6 to 30) | 5.9 (-20.5 to 38.9) | 5.2 (-13.7 to 37.5) |
|  |  | All ages | 147.4 (111.7 to 189.3) | 75.7 (54.9 to 98) | 71.7 (50.7 to 94.2) | 477.9 (369.7 to 596) | 272.5 (210.6 to 335.5) | 205.4 (154.1 to 267.9) | 224.3 (165.6 to 296.2) | 260.2 (175.6 to 376.8) | 186.5 (133.2 to 271.7) |
|  | Deaths | Age-standardized | 2.7 (1.7 to 3.6) | 3.3 (2.2 to 4.7) | 2.1 (1.2 to 2.9) | 2.8 (2 to 3.4) | 3.5 (2.4 to 4.4) | 2.1 (1.4 to 2.6) | 5.2 (-25.6 to 57.5) | 6.9 (-31 to 61) | -1.6 (-31.4 to 90.6) |
|  |  | All ages | 4.7 (3.1 to 6.2) | 2.8 (1.9 to 4) | 1.8 (1.1 to 2.5) | 21.3 (14.8 to 26) | 14.3 (9.8 to 17.9) | 7 (4.7 to 9) | 354.1 (220.1 to 582.4) | 402.1 (224.1 to 663.9) | 279.9 (162.3 to 626.2) |
| Tehran | Incidence | Age-standardized | 37 (27.4 to 49.7) | 33.8 (25.1 to 45.5) | 40.5 (29.7 to 54.4) | 39.2 (29 to 52.7) | 35.2 (26 to 47.9) | 42.9 (31.4 to 57.4) | 6.1 (-0.1 to 11.4) | 4.2 (-2.1 to 11.2) | 6 (-3 to 13.7) |
|  |  | All ages | 1289.1 (979.4 to 1711.1) | 579.7 (438.1 to 775.8) | 709.4 (541.7 to 940.9) | 5680.5 (4266.8 to 7525.2) | 2454.7 (1858.9 to 3267.8) | 3225.9 (2401 to 4279.2) | 340.7 (305.4 to 379.4) | 323.5 (294.2 to 359.4) | 354.7 (304.7 to 413.4) |
|  | Prevalence | Age-standardized | 384.7 (294.7 to 509.3) | 347.6 (263.6 to 459) | 423.7 (322.5 to 564.5) | 413.3 (317.9 to 543.5) | 365.6 (278 to 480.2) | 453.9 (349.3 to 588) | 7.4 (1.5 to 12.7) | 5.2 (-1.8 to 13.2) | 7.1 (-0.3 to 13.8) |
|  |  | All ages | 12155.5 (9419.6 to 15986.2) | 5445.8 (4162.9 to 7164.6) | 6709.7 (5195.7 to 8735.5) | 58038.5 (45232.7 to 76182.8) | 24288.1 (18550.5 to 31855.8) | 33750.3 (26028.7 to 43851.8) | 377.5 (345.1 to 415.1) | 346 (314.8 to 383) | 403 (354.7 to 453.2) |
|  | DALYs (Disability-Adjusted Life Years) | Age-standardized | 65.5 (50.8 to 84.8) | 71.3 (53.8 to 93.9) | 59.2 (41 to 78.6) | 63.9 (49.3 to 79.3) | 70.9 (53 to 86.3) | 59.3 (44 to 78.2) | -2.4 (-20.1 to 13.8) | -0.6 (-25.9 to 25) | 0.2 (-18.8 to 23) |
|  |  | All ages | 1852.8 (1408.5 to 2402.4) | 991.3 (747.2 to 1312.6) | 861.4 (609.3 to 1138.8) | 8800 (6771.1 to 10928.6) | 4338 (3230.1 to 5300.8) | 4461.9 (3324.6 to 5867.8) | 375 (295.5 to 453.8) | 337.6 (227.6 to 447) | 418 (318.4 to 540.2) |
|  | Deaths | Age-standardized | 2.9 (2.1 to 3.9) | 3.6 (2.6 to 5.2) | 2.2 (1.4 to 3.2) | 2.8 (2 to 3.4) | 3.8 (2.7 to 4.7) | 2.1 (1.4 to 2.8) | -4.9 (-31.8 to 25.9) | 4 (-32.8 to 46.5) | -3.5 (-36.3 to 50.2) |
|  |  | All ages | 62.8 (46 to 82.5) | 39.4 (28.6 to 56.5) | 23.3 (14.9 to 34.6) | 366.8 (270.6 to 443.2) | 203.3 (145.4 to 256.3) | 163.6 (109.4 to 215.2) | 484.5 (317.9 to 678.6) | 415.7 (230.2 to 634.7) | 600.5 (357.3 to 999.3) |
| West Azarbayejan | Incidence | Age-standardized | 36 (26.4 to 48) | 32.8 (23.8 to 44.1) | 39.2 (28.9 to 52.2) | 38.5 (28.6 to 51.4) | 34.9 (25.8 to 46.7) | 42.5 (31.4 to 56.7) | 7.1 (1.8 to 12.6) | 6.4 (-0.9 to 14.2) | 8.6 (2.2 to 16.9) |
|  |  | All ages | 283.9 (212.6 to 376.5) | 122.4 (90.8 to 161.8) | 161.5 (120.4 to 215.3) | 992.9 (760.6 to 1303.7) | 464.6 (352.9 to 622.2) | 528.2 (407.4 to 691.2) | 249.7 (224.5 to 285.8) | 279.7 (247.4 to 322.2) | 227 (199.3 to 264.4) |
|  | Prevalence | Age-standardized | 369.4 (281 to 485.7) | 333.4 (251 to 439.6) | 406.1 (310 to 535.3) | 401.8 (309 to 526) | 359 (275.1 to 475.1) | 447.3 (344.7 to 584.6) | 8.8 (4.3 to 14.3) | 7.7 (-0.1 to 15.8) | 10.2 (4.3 to 17.7) |
|  |  | All ages | 2591.6 (2008.4 to 3409.7) | 1098.2 (841.7 to 1449.3) | 1493.4 (1161.5 to 1974.5) | 9613.2 (7515 to 12648.7) | 4469.8 (3476.9 to 5961.1) | 5143.4 (3966.3 to 6701.9) | 270.9 (245.7 to 300) | 307 (268.3 to 345) | 244.4 (218.7 to 274.3) |
|  | DALYs (Disability-Adjusted Life Years) | Age-standardized | 81.2 (59.3 to 99.8) | 92.1 (66.5 to 116.1) | 69.7 (50.6 to 88.8) | 86.4 (64.7 to 103.9) | 102.5 (73.3 to 123.5) | 70.3 (51.5 to 87.7) | 6.4 (-13.4 to 32.3) | 11.4 (-16.8 to 43.9) | 0.9 (-15.7 to 36.6) |
|  |  | All ages | 451 (350.1 to 558.8) | 238.5 (176.5 to 300.7) | 212.5 (156.3 to 273.2) | 1725.1 (1331.3 to 2093) | 1010.2 (753.7 to 1234.4) | 715 (522.2 to 899.4) | 282.5 (219.5 to 362.3) | 323.5 (222.3 to 439.3) | 236.5 (180.1 to 333.3) |
|  | Deaths | Age-standardized | 4.6 (2.8 to 5.8) | 5.7 (3.6 to 7.5) | 3.3 (1.9 to 4.4) | 4.9 (3.3 to 5.9) | 6.7 (4.4 to 8.3) | 3.1 (1.8 to 4) | 7.3 (-22 to 49.6) | 18 (-19.3 to 68.6) | -6.4 (-35.4 to 70.7) |
|  |  | All ages | 16.5 (10.6 to 21.2) | 10.5 (6.9 to 14) | 6 (3.6 to 8) | 73.7 (51 to 89.5) | 51.1 (34 to 63.8) | 22.7 (14.1 to 29.4) | 345.7 (229.4 to 520) | 384.5 (225.3 to 601.1) | 277.5 (169.1 to 567.6) |
| Yazd | Incidence | Age-standardized | 35.9 (26.5 to 48.5) | 33.1 (24.7 to 44.8) | 39.7 (29.1 to 54.2) | 39.7 (29.3 to 53.7) | 35.8 (26.5 to 47.9) | 43.7 (32.3 to 59.6) | 10.6 (5.9 to 15.4) | 8.2 (1.6 to 15.8) | 10.1 (3.6 to 17.9) |
|  |  | All ages | 107.3 (80.5 to 142.7) | 51.6 (38.4 to 70.3) | 55.7 (41.1 to 73.2) | 364.5 (276.8 to 486.8) | 162.1 (122.9 to 214.7) | 202.4 (154 to 269.8) | 239.8 (212.2 to 270.8) | 214.1 (184.9 to 243) | 263.6 (225.7 to 305.3) |
|  | Prevalence | Age-standardized | 369.4 (282 to 488.5) | 337.7 (254.5 to 446.9) | 413.2 (312.7 to 545.7) | 414.6 (317 to 547.8) | 370.1 (281.1 to 488.7) | 461.5 (351 to 608.2) | 12.2 (7.8 to 17.4) | 9.6 (2.7 to 16.9) | 11.7 (5.1 to 19) |
|  |  | All ages | 1008.5 (776.8 to 1329.6) | 489.7 (372.1 to 653.5) | 518.8 (405.7 to 684.6) | 3652.8 (2819.8 to 4760.5) | 1622.2 (1241 to 2141.8) | 2030.6 (1566.5 to 2622.3) | 262.2 (237.5 to 287.3) | 231.3 (205 to 259.8) | 291.4 (258.3 to 328.9) |
|  | DALYs (Disability-Adjusted Life Years) | Age-standardized | 73.1 (56.8 to 91.7) | 79.2 (59.6 to 98.8) | 64 (46.1 to 82.1) | 73.2 (56.7 to 88.5) | 82.3 (63.8 to 102.1) | 62.6 (46.9 to 79.9) | 0.1 (-18.9 to 20.3) | 4 (-21 to 33.2) | -2.1 (-19.2 to 27.3) |
|  |  | All ages | 172 (135.5 to 215.7) | 101.9 (77.4 to 128.8) | 70.1 (50.2 to 92.5) | 604.8 (470.4 to 736.7) | 345.1 (266.5 to 429.4) | 259.7 (194 to 330.7) | 251.6 (187.1 to 318.5) | 238.6 (160.8 to 330.5) | 270.5 (212 to 367.4) |
|  | Deaths | Age-standardized | 3.7 (2.6 to 4.8) | 4.3 (3 to 5.7) | 2.7 (1.6 to 3.9) | 3.6 (2.6 to 4.3) | 4.6 (3.3 to 5.8) | 2.3 (1.5 to 3) | -2.9 (-31 to 34) | 7.4 (-28.3 to 54.1) | -13.4 (-41.4 to 55.5) |
|  |  | All ages | 6.4 (4.6 to 8.1) | 4.5 (3.1 to 6) | 1.9 (1.2 to 2.5) | 26.5 (19.3 to 32.3) | 18.5 (13.3 to 23.3) | 8 (5.1 to 10.3) | 314.6 (195.1 to 470.2) | 308.3 (174.7 to 488.6) | 330.1 (198.1 to 662.9) |
| Zanjan | Incidence | Age-standardized | 37.1 (27.7 to 49.8) | 34 (25.5 to 45.5) | 40.9 (30 to 54.3) | 41 (30.3 to 54.4) | 37.1 (27.6 to 50) | 45.2 (33.4 to 60.4) | 10.3 (6.2 to 14.6) | 9 (2.9 to 16.3) | 10.7 (4.9 to 17.5) |
|  |  | All ages | 126.3 (93.6 to 167.9) | 55.7 (41.9 to 74.1) | 70.7 (51.1 to 95.3) | 404.6 (307.1 to 532.4) | 191.3 (144.5 to 253.3) | 213.3 (161 to 280.7) | 220.3 (182.9 to 261.6) | 243.6 (209 to 281.5) | 201.9 (156.7 to 252) |
|  | Prevalence | Age-standardized | 381.5 (290.9 to 499.4) | 348.3 (266.1 to 456.2) | 425.2 (324.3 to 559.3) | 428.7 (329.8 to 564.2) | 384.6 (291.5 to 505.4) | 478.4 (368.4 to 629.4) | 12.4 (7.7 to 17.5) | 10.4 (4.1 to 16.9) | 12.5 (6.5 to 19.5) |
|  |  | All ages | 1170 (901.8 to 1538) | 517.9 (401.5 to 682.2) | 652 (498.8 to 848.6) | 4103.5 (3184.1 to 5355.7) | 1931 (1482.2 to 2516.2) | 2172.5 (1688.6 to 2840.8) | 250.7 (220.6 to 283) | 272.8 (237.1 to 305.1) | 233.2 (199.8 to 272.8) |
|  | DALYs (Disability-Adjusted Life Years) | Age-standardized | 64.8 (49.6 to 81.6) | 66.6 (50.8 to 83.9) | 62.4 (45 to 79.5) | 70 (53.8 to 86.7) | 72.3 (55.1 to 89.5) | 66.5 (50.2 to 85.5) | 8 (-9.7 to 29) | 8.6 (-14.7 to 36.2) | 6.6 (-11 to 36.3) |
|  |  | All ages | 175.7 (133.8 to 223.5) | 91.7 (69.1 to 117.3) | 84 (60.3 to 110.3) | 647.5 (498.8 to 808.3) | 355 (270.4 to 439.4) | 292.5 (218.6 to 375.9) | 268.5 (212.5 to 340.1) | 287 (204.2 to 390.8) | 248.4 (189.3 to 341.7) |
|  | Deaths | Age-standardized | 2.9 (1.9 to 3.7) | 3.2 (2.2 to 4.2) | 2.4 (1.4 to 3.3) | 3.1 (2.2 to 3.8) | 3.6 (2.5 to 4.6) | 2.4 (1.6 to 3) | 8 (-20 to 49.9) | 14.1 (-22.5 to 64.2) | 0.5 (-30.1 to 75.1) |
|  |  | All ages | 5.6 (3.8 to 7.1) | 3.7 (2.5 to 4.9) | 1.9 (1.1 to 2.6) | 26.8 (18.9 to 32.5) | 17.2 (11.9 to 21.8) | 9.6 (6.3 to 12) | 381.5 (256.7 to 574.4) | 366 (217.2 to 574.6) | 412.3 (256.6 to 778.7) |

**Table S2.** All‑ages number and age‑standardized rate of incidence, prevalence, disability-adjusted life years (DALYs), and deaths of atrial fibrillation and flutter by sex in 2019 and 2021 and overall percent change over 2019–2021 in Iran provinces

| Location | Measure | Age, Metric | Year | | | | | | % Change (2019 to 2021) | | |
| --- | --- | --- | --- | --- | --- | --- | --- | --- | --- | --- | --- |
|  |  |  | 2019 | | | 2021 | | |  |  |  |
|  |  |  | Both | Women | Men | Both | Women | Men | Both | Women | Men |
| Iran (Islamic Republic of) | Incidence | Age-standardized | 40.5 (30.1 to 54.1) | 37.4 (27.8 to 49.6) | 43.5 (32.4 to 58.2) | 40.6 (30 to 54.4) | 37.4 (27.7 to 50.2) | 43.7 (32.3 to 58.7) | 0.2 (-1.1 to 1.6) | 0.1 (-1.6 to 1.8) | 0.5 (-1.3 to 2.2) |
|  |  | All ages | 27685.4 (21235 to 36313.8) | 12746.5 (9751.9 to 16787.2) | 14938.9 (11441.1 to 19762.5) | 29418.3 (22665.1 to 38723.1) | 13622.3 (10363.4 to 18155.8) | 15796 (12121.5 to 20711) | 6.3 (4.5 to 8) | 6.9 (5 to 8.7) | 5.7 (3.4 to 8) |
|  | Prevalence | Age-standardized | 424.5 (325.8 to 555.4) | 387.4 (297.1 to 506.4) | 459.1 (350.5 to 599.9) | 425.4 (327.2 to 559.2) | 387.8 (298 to 510.7) | 461.1 (354.7 to 601.6) | 0.2 (-1.2 to 1.6) | 0.1 (-1.5 to 1.7) | 0.5 (-1.3 to 2.2) |
|  |  | All ages | 278770.9 (216848.8 to 360907.9) | 125967.5 (97128.5 to 164514.5) | 152803.4 (117891.9 to 197505.6) | 294248.7 (230024.2 to 382165.9) | 134056.9 (103750.2 to 176465.5) | 160191.8 (124346.3 to 206830.5) | 5.6 (4 to 7) | 6.4 (4.8 to 8.1) | 4.8 (2.7 to 6.9) |
|  | DALYs (Disability-Adjusted Life Years) | Age-standardized | 74.7 (59.1 to 91) | 86.2 (67.7 to 101.4) | 64.5 (49.7 to 81.9) | 72.4 (57.4 to 88.3) | 83.5 (65.3 to 99.2) | 62.5 (47.4 to 80.3) | -3 (-5.9 to -0.1) | -3.1 (-7 to 0.7) | -3.1 (-6.6 to 0) |
|  |  | All ages | 46952.6 (37082.5 to 57309.6) | 25858.2 (20240.6 to 30539.4) | 21094.5 (16143.2 to 26593.5) | 47524.7 (37527.7 to 58270.9) | 26310.9 (20508.1 to 31274.3) | 21213.7 (16083.8 to 27193.4) | 1.2 (-1.8 to 4.2) | 1.8 (-2.1 to 6) | 0.6 (-3 to 3.8) |
|  | Deaths | Age-standardized | 3.5 (2.6 to 3.9) | 4.8 (3.5 to 5.4) | 2.4 (1.6 to 2.8) | 3.3 (2.5 to 3.8) | 4.6 (3.3 to 5.3) | 2.2 (1.5 to 2.7) | -5.2 (-9.8 to -0.5) | -4.1 (-9.6 to 1.1) | -7.4 (-14.1 to -1.2) |
|  |  | All ages | 2004.4 (1498.6 to 2250.6) | 1270.1 (940.8 to 1436.8) | 734.2 (505.4 to 847.2) | 1948.9 (1462.6 to 2233.1) | 1258.4 (920 to 1445.6) | 690.6 (478.6 to 823.5) | -2.8 (-7.6 to 2) | -0.9 (-6.7 to 4.7) | -5.9 (-12.6 to 0.4) |
| Alborz | Incidence | Age-standardized | 41.3 (30.8 to 55.3) | 36.9 (27.7 to 49.1) | 45 (33.2 to 60.6) | 41.5 (30.7 to 56.2) | 37 (27.2 to 49.4) | 45.4 (33.4 to 62.1) | 0.5 (-4 to 5) | 0.2 (-5.4 to 6.9) | 0.9 (-6.4 to 8.6) |
|  |  | All ages | 883.9 (680.3 to 1165) | 377.1 (286.7 to 491.7) | 506.8 (388 to 667.5) | 973.7 (745.4 to 1286.5) | 420.5 (317.3 to 556.6) | 553.2 (426.7 to 737.3) | 10.2 (5.3 to 14.7) | 11.5 (4.8 to 19.3) | 9.2 (2.1 to 16.8) |
|  | Prevalence | Age-standardized | 436.7 (333.6 to 575.4) | 384 (293.2 to 500) | 476.7 (361.7 to 628.2) | 439 (336.3 to 579.4) | 385 (292.3 to 505.6) | 481.2 (370.7 to 639.9) | 0.5 (-3.6 to 4.9) | 0.3 (-5.6 to 6.8) | 1 (-5.6 to 7.5) |
|  |  | All ages | 8500 (6659 to 11060.2) | 3511.5 (2706.4 to 4576.8) | 4988.4 (3880.7 to 6502.7) | 9335.2 (7256.4 to 12223.4) | 3912.7 (3009 to 5182.6) | 5422.5 (4151.6 to 7061.2) | 9.8 (5.6 to 14.2) | 11.4 (5 to 19.1) | 8.7 (2.5 to 15.5) |
|  | DALYs (Disability-Adjusted Life Years) | Age-standardized | 79.3 (61.9 to 97.9) | 112 (82.3 to 136.3) | 63.6 (47.4 to 82.3) | 76 (58.5 to 93.7) | 103.6 (77.6 to 126.2) | 62 (45.1 to 81) | -4.1 (-13.1 to 6.4) | -7.5 (-20.9 to 7) | -2.5 (-14.3 to 10.9) |
|  |  | All ages | 1358.9 (1052.6 to 1683.7) | 725.2 (547.8 to 894.6) | 633.7 (466.2 to 816.4) | 1420.6 (1091.7 to 1777.8) | 757.2 (584 to 936.3) | 663.4 (484.7 to 869.4) | 4.5 (-5.4 to 16.2) | 4.4 (-11 to 21.4) | 4.7 (-8.5 to 19.3) |
|  | Deaths | Age-standardized | 3.9 (2.7 to 4.6) | 7.6 (5.1 to 9.4) | 2.2 (1.4 to 2.8) | 3.6 (2.5 to 4.3) | 6.7 (4.5 to 8.5) | 2 (1.3 to 2.7) | -8 (-21.1 to 7.4) | -11.1 (-26.6 to 6.1) | -7.4 (-29 to 17.5) |
|  |  | All ages | 51.1 (35.8 to 61.5) | 33.6 (23 to 42) | 17.5 (11.7 to 22.6) | 50.2 (36.5 to 60.9) | 33.2 (23.6 to 41.9) | 17.1 (11.1 to 22.4) | -1.7 (-16.9 to 16.1) | -1.2 (-20.6 to 22.2) | -2.7 (-26.2 to 25.1) |
| Ardebil | Incidence | Age-standardized | 41.2 (30.3 to 54.9) | 37.4 (27.4 to 48.8) | 45.6 (33.5 to 61.7) | 41.3 (30.5 to 55.6) | 37.5 (27.7 to 50.4) | 45.7 (33.4 to 61.7) | 0.2 (-3.9 to 4.5) | 0.4 (-6.3 to 7.4) | 0.3 (-7.4 to 6.4) |
|  |  | All ages | 451.4 (340.7 to 599.1) | 216 (161.1 to 280.2) | 235.5 (177.8 to 315.7) | 469 (355.3 to 623.3) | 227.2 (171.3 to 302.8) | 241.7 (181.8 to 324) | 3.9 (-0.2 to 8.1) | 5.2 (-1.5 to 11.8) | 2.7 (-5.4 to 9.2) |
|  | Prevalence | Age-standardized | 431.1 (329.8 to 564.8) | 387.2 (289.5 to 506.4) | 481.9 (367.3 to 634.8) | 431.4 (330.9 to 567) | 388.3 (296.3 to 515.6) | 483.2 (366.2 to 644.8) | 0.1 (-4 to 4.5) | 0.3 (-6.3 to 7.6) | 0.3 (-6.9 to 6.8) |
|  |  | All ages | 4576.6 (3510.7 to 5931.3) | 2185.4 (1643.1 to 2846.6) | 2391.2 (1819 to 3115.7) | 4710.5 (3625 to 6192.5) | 2283.3 (1745.3 to 3016.2) | 2427.3 (1865.9 to 3175.1) | 2.9 (-1 to 7.3) | 4.5 (-1.9 to 11.8) | 1.5 (-5.6 to 8.3) |
|  | DALYs (Disability-Adjusted Life Years) | Age-standardized | 81.2 (63.3 to 98.1) | 87.3 (68.4 to 102.8) | 73.5 (54.7 to 91.8) | 78.6 (60.8 to 95) | 84.4 (64.5 to 102.4) | 70.7 (52.3 to 89.4) | -3.2 (-9.4 to 3.6) | -3.3 (-12.2 to 6.7) | -3.8 (-10.8 to 3.3) |
|  |  | All ages | 829 (645.6 to 1001.6) | 477.2 (375.3 to 565.2) | 351.8 (260.1 to 441.7) | 815.2 (634.4 to 986.6) | 475.2 (366.5 to 574.3) | 340 (253.9 to 426.2) | -1.7 (-8.3 to 5.2) | -0.4 (-9.7 to 9.7) | -3.4 (-10.8 to 4.4) |
|  | Deaths | Age-standardized | 3.9 (2.9 to 4.6) | 4.7 (3.4 to 5.7) | 2.9 (2 to 3.6) | 3.8 (2.7 to 4.5) | 4.5 (3.1 to 5.5) | 2.7 (1.8 to 3.4) | -4.8 (-13.6 to 4.7) | -4.4 (-16 to 7.9) | -7.7 (-17.8 to 3.5) |
|  |  | All ages | 37.2 (27.3 to 43.5) | 24.6 (17.8 to 29.5) | 12.5 (8.4 to 15.2) | 35.1 (25.4 to 41.8) | 23.9 (16.8 to 29.2) | 11.2 (7.6 to 14.1) | -5.5 (-14.5 to 4.3) | -3.1 (-15.2 to 9.9) | -10.3 (-20.3 to 1.3) |
| Bushehr | Incidence | Age-standardized | 39.4 (29.1 to 52.6) | 35.5 (26.3 to 47.3) | 43.2 (32.1 to 58.1) | 39.3 (29.5 to 52.2) | 35.5 (26.6 to 47.8) | 43.3 (32.1 to 58) | -0.1 (-4 to 4.4) | 0.1 (-5.7 to 7.6) | 0.1 (-6.8 to 7) |
|  |  | All ages | 298.8 (230.8 to 394.2) | 134.4 (101.5 to 177.4) | 164.4 (127.5 to 214.3) | 322 (248.3 to 423.7) | 146.8 (111.9 to 194.5) | 175.2 (134.1 to 230.2) | 7.8 (3.6 to 12.5) | 9.2 (3.1 to 17.2) | 6.6 (0 to 13.3) |
|  | Prevalence | Age-standardized | 411.3 (314.9 to 539.3) | 366.9 (277.9 to 481.9) | 455.9 (349.5 to 598) | 410.8 (315.6 to 537.6) | 367.4 (282.3 to 485.5) | 456 (348.4 to 601.6) | -0.1 (-4.1 to 4.4) | 0.1 (-5.9 to 7.4) | 0 (-6 to 6.4) |
|  |  | All ages | 2890.5 (2267.8 to 3765.6) | 1292.4 (987.7 to 1677.4) | 1598.2 (1259.8 to 2076.3) | 3110.6 (2445 to 4083.4) | 1413.3 (1100.8 to 1852.8) | 1697.3 (1322.2 to 2212) | 7.6 (3.2 to 12.7) | 9.4 (3.1 to 17.2) | 6.2 (0.2 to 12.7) |
|  | DALYs (Disability-Adjusted Life Years) | Age-standardized | 73.9 (56.8 to 89.7) | 80.9 (62.3 to 97.3) | 66.3 (50.5 to 84.4) | 71.4 (55.4 to 88.1) | 78 (59.9 to 94.6) | 64.1 (47.7 to 81.9) | -3.4 (-10.3 to 4) | -3.6 (-12 to 6.1) | -3.4 (-12.6 to 6.3) |
|  |  | All ages | 467.7 (364.7 to 570.1) | 252.6 (198 to 304.6) | 215.1 (160.8 to 273.3) | 484.8 (375.1 to 602.9) | 264.4 (201.5 to 322.3) | 220.4 (164.4 to 283.2) | 3.7 (-3.9 to 12.4) | 4.7 (-4.9 to 15.9) | 2.5 (-8.2 to 13.7) |
|  | Deaths | Age-standardized | 3.5 (2.6 to 4.1) | 4.4 (3.3 to 5.2) | 2.6 (1.7 to 3.2) | 3.3 (2.4 to 3.9) | 4.2 (3 to 5) | 2.4 (1.5 to 3) | -5.4 (-14.9 to 5.4) | -4.7 (-16.1 to 9.7) | -7.4 (-21 to 7.9) |
|  |  | All ages | 17.6 (13 to 20.6) | 11.1 (8.2 to 13.3) | 6.5 (4.3 to 8) | 17.6 (13.2 to 20.8) | 11.4 (8.2 to 13.7) | 6.3 (4.1 to 7.9) | 0 (-10.7 to 11.9) | 2.1 (-11.1 to 18.9) | -3.7 (-18.8 to 12.9) |
| Chahar Mahaal and Bakhtiari | Incidence | Age-standardized | 39.2 (29.4 to 52.5) | 35.6 (26.7 to 47.6) | 43.3 (32 to 58.8) | 39.2 (29.2 to 52.1) | 35.7 (26.8 to 47.8) | 43.5 (32.3 to 57.8) | 0.1 (-4.1 to 4.4) | 0.1 (-5 to 6) | 0.3 (-6.2 to 8.4) |
|  |  | All ages | 298.7 (230 to 394.7) | 143.1 (109 to 187.9) | 155.5 (118.8 to 205.4) | 322 (247.5 to 419.9) | 155.3 (118.1 to 203.9) | 166.7 (127.9 to 216.4) | 7.8 (3 to 12.5) | 8.5 (3 to 15.2) | 7.2 (0.9 to 15.2) |
|  | Prevalence | Age-standardized | 410.9 (316.9 to 537.4) | 370.1 (282.4 to 482) | 459.1 (353.6 to 605) | 411.3 (317.9 to 535.3) | 370.9 (280.6 to 484.6) | 460.4 (355.9 to 601.1) | 0.1 (-3.8 to 4.4) | 0.2 (-5.5 to 6.5) | 0.3 (-5.4 to 7.4) |
|  |  | All ages | 3005.1 (2316.6 to 3919.8) | 1440.4 (1104.2 to 1861.4) | 1564.6 (1202 to 2052.4) | 3219.4 (2538.2 to 4172.2) | 1560.7 (1191.5 to 2029.2) | 1658.7 (1293.1 to 2130.9) | 7.1 (3 to 11.7) | 8.3 (2 to 15.4) | 6 (-0.3 to 13) |
|  | DALYs (Disability-Adjusted Life Years) | Age-standardized | 66.4 (50.6 to 82.3) | 67.2 (50.6 to 83.4) | 64.9 (48.7 to 82.3) | 64.3 (49.2 to 79.9) | 65 (48.2 to 81.9) | 62.7 (46.7 to 80.1) | -3.2 (-10.6 to 5.1) | -3.3 (-14.5 to 9) | -3.4 (-13 to 6.7) |
|  |  | All ages | 465.1 (353.5 to 580.4) | 253 (192.1 to 315) | 212.1 (160.6 to 270.7) | 478.2 (364.6 to 602.2) | 263.1 (196.2 to 332.9) | 215.1 (161 to 277.6) | 2.8 (-5.2 to 11.7) | 4 (-8.1 to 17.1) | 1.4 (-8.3 to 12.3) |
|  | Deaths | Age-standardized | 2.9 (2.1 to 3.5) | 3.3 (2.4 to 4.1) | 2.4 (1.7 to 3) | 2.8 (2.1 to 3.4) | 3.1 (2.2 to 4) | 2.2 (1.5 to 2.8) | -5.4 (-16.5 to 6.6) | -4.7 (-20.4 to 13.3) | -7.7 (-22.9 to 8.9) |
|  |  | All ages | 18.6 (13.6 to 21.7) | 11.7 (8.7 to 14.5) | 6.9 (4.8 to 8.5) | 18.4 (13.8 to 22.5) | 11.9 (8.4 to 15.1) | 6.5 (4.5 to 8.1) | -1.1 (-13.2 to 11.8) | 1.7 (-15.5 to 21.2) | -5.7 (-21.5 to 11.7) |
| East Azarbayejan | Incidence | Age-standardized | 40.6 (30.4 to 53.9) | 36.7 (27.5 to 49.4) | 44.6 (32.8 to 59.4) | 40.8 (30 to 54.4) | 36.7 (26.9 to 49.2) | 44.9 (33.3 to 60.2) | 0.3 (-4.4 to 4.5) | 0 (-6 to 6.3) | 0.8 (-5.6 to 7.9) |
|  |  | All ages | 1443.4 (1104.3 to 1914.1) | 655.5 (494.7 to 886) | 787.9 (592.3 to 1045.9) | 1523.2 (1163 to 2025.7) | 693.1 (522.4 to 927.9) | 830.1 (635.5 to 1093.3) | 5.5 (0.9 to 10.8) | 5.7 (-0.7 to 13.3) | 5.4 (-1.5 to 13) |
|  | Prevalence | Age-standardized | 424.5 (322.8 to 556.9) | 378.5 (288.1 to 498.2) | 470.4 (357.9 to 610.1) | 425.9 (325.8 to 554.9) | 378.9 (290.2 to 503.5) | 473.3 (364.3 to 613.3) | 0.3 (-3.4 to 5) | 0.1 (-5.8 to 7.2) | 0.6 (-5.5 to 7.6) |
|  |  | All ages | 14288.9 (10937.2 to 18580.9) | 6410 (4923.9 to 8457.1) | 7878.9 (6040.9 to 10243.4) | 14983.7 (11699.5 to 19453.4) | 6747.9 (5235.2 to 8899.7) | 8235.8 (6429.3 to 10585.8) | 4.9 (0.9 to 9.8) | 5.3 (-1 to 12.8) | 4.5 (-2 to 11.4) |
|  | DALYs (Disability-Adjusted Life Years) | Age-standardized | 90.1 (71.4 to 108.6) | 106.9 (81.3 to 129.9) | 73.4 (54.5 to 90.7) | 87.1 (69.1 to 106.4) | 103.4 (80 to 126.2) | 70.8 (52.9 to 89.2) | -3.3 (-12.5 to 5.9) | -3.2 (-16.2 to 10.1) | -3.5 (-12.2 to 6.2) |
|  |  | All ages | 2595.3 (2067.4 to 3136.9) | 1484.3 (1142.4 to 1820.1) | 1111 (833.3 to 1382.1) | 2615.8 (2077.1 to 3238.2) | 1503.4 (1172.8 to 1844.2) | 1112.4 (818.5 to 1425.7) | 0.8 (-8.6 to 10.6) | 1.3 (-12.5 to 15.8) | 0.1 (-8.9 to 10.5) |
|  | Deaths | Age-standardized | 5 (3.7 to 5.9) | 6.8 (4.7 to 8.5) | 3.2 (2.1 to 3.9) | 4.7 (3.4 to 5.8) | 6.6 (4.4 to 8.2) | 2.9 (1.8 to 3.8) | -4.8 (-17.1 to 8.1) | -3.8 (-20 to 13.6) | -7.2 (-21.3 to 8.8) |
|  |  | All ages | 111.6 (80.8 to 132.3) | 75 (52.3 to 92.1) | 36.5 (24.6 to 45) | 110 (80.7 to 133.5) | 75.4 (52.7 to 94.8) | 34.5 (22.2 to 45.3) | -1.4 (-14.8 to 13.3) | 0.5 (-17.5 to 20.3) | -5.4 (-21.6 to 13.9) |
| Fars | Incidence | Age-standardized | 49.1 (35.9 to 65.9) | 58 (42.6 to 76.9) | 39.8 (28.4 to 54.2) | 49 (36.1 to 66.8) | 57.8 (42.9 to 77.4) | 39.8 (28.7 to 55.5) | -0.1 (-5 to 4.8) | -0.3 (-7.1 to 6) | 0.2 (-8.8 to 7.8) |
|  |  | All ages | 2042.8 (1544 to 2673.4) | 1235.1 (920.1 to 1626.2) | 807.7 (597.9 to 1092.8) | 2190.2 (1640.6 to 2929.7) | 1329.1 (995.3 to 1774.2) | 861.2 (637.7 to 1191.2) | 7.2 (2 to 12.3) | 7.6 (0.9 to 14.2) | 6.6 (-2.8 to 14.4) |
|  | Prevalence | Age-standardized | 520.5 (391.3 to 686.1) | 621 (463 to 812.7) | 415.8 (309.4 to 567.9) | 520.9 (394.7 to 694.4) | 619.4 (468.6 to 822.5) | 416.8 (308.4 to 572.6) | 0.1 (-4.3 to 4.8) | -0.3 (-5.8 to 6) | 0.2 (-8.5 to 7.7) |
|  |  | All ages | 20448.2 (15507.9 to 26930.1) | 12384.9 (9414.3 to 16201.5) | 8063.3 (6089.2 to 10973.9) | 21782.3 (16721.8 to 28803.5) | 13267.1 (10170.6 to 17638.3) | 8515.2 (6379.4 to 11592.4) | 6.5 (1.9 to 11.6) | 7.1 (1.4 to 13.8) | 5.6 (-3.4 to 13.6) |
|  | DALYs (Disability-Adjusted Life Years) | Age-standardized | 88 (69 to 107.7) | 111.5 (83.9 to 136.1) | 65.3 (48.4 to 83.5) | 85.8 (66.9 to 108.1) | 108.7 (84.4 to 135.2) | 63.1 (45.4 to 82.2) | -2.6 (-11.3 to 7) | -2.5 (-13.5 to 11.3) | -3.3 (-14.5 to 8.7) |
|  |  | All ages | 3177.6 (2464.3 to 3937.8) | 1976.4 (1492.5 to 2456.5) | 1201.2 (885.3 to 1539.6) | 3270.6 (2556.1 to 4141.8) | 2057.2 (1604.5 to 2597.4) | 1213.4 (868 to 1574.7) | 2.9 (-6 to 13.1) | 4.1 (-7.6 to 19.3) | 1 (-10.7 to 13.7) |
|  | Deaths | Age-standardized | 4.2 (3 to 5) | 5.6 (4.1 to 6.8) | 2.8 (1.8 to 3.5) | 4 (2.9 to 4.7) | 5.4 (3.9 to 6.6) | 2.6 (1.7 to 3.3) | -4.9 (-18.3 to 10.7) | -4.1 (-20 to 16.5) | -7.3 (-24.1 to 12) |
|  |  | All ages | 126.6 (93.9 to 150.9) | 81.8 (60.6 to 99.2) | 44.8 (29.4 to 56.4) | 125 (93.2 to 147.5) | 82.6 (59.8 to 101) | 42.4 (27.5 to 52.6) | -1.3 (-15.7 to 15.3) | 1 (-16.5 to 23.8) | -5.5 (-22.9 to 15.5) |
| Gilan | Incidence | Age-standardized | 40.6 (30.3 to 54.4) | 36.7 (27.2 to 48.8) | 44.6 (33.4 to 60) | 40.6 (30 to 55) | 36.6 (27.1 to 49.9) | 44.7 (32.8 to 59.6) | 0 (-4.7 to 4.5) | -0.2 (-6.7 to 6.7) | 0.1 (-5.6 to 7.5) |
|  |  | All ages | 1166 (889.1 to 1549.7) | 534.6 (406 to 709.1) | 631.4 (478.6 to 838.5) | 1220.9 (918.9 to 1636.4) | 561.4 (423.4 to 763.2) | 659.5 (494.4 to 873.6) | 4.7 (-0.3 to 9.5) | 5 (-2.1 to 12.4) | 4.5 (-1.6 to 11.7) |
|  | Prevalence | Age-standardized | 426.4 (324.3 to 561.6) | 380.1 (284.6 to 498.6) | 472.5 (358.8 to 619.1) | 426.6 (327.5 to 567.7) | 380 (291.3 to 512.9) | 473.4 (359.3 to 621.1) | 0.1 (-4.3 to 4.3) | 0 (-6.1 to 7.2) | 0.2 (-5.5 to 7.3) |
|  |  | All ages | 11797.3 (9057.3 to 15356.1) | 5325.7 (3999.6 to 6948.9) | 6471.5 (4933.4 to 8486.9) | 12284.7 (9487.7 to 16119.6) | 5563.4 (4284.1 to 7474.6) | 6721.3 (5152.3 to 8683.4) | 4.1 (-0.3 to 8.6) | 4.5 (-1.9 to 12.1) | 3.9 (-2.1 to 11) |
|  | DALYs (Disability-Adjusted Life Years) | Age-standardized | 81.4 (64 to 96.2) | 95.9 (71.9 to 113.7) | 67.8 (49 to 86.3) | 77.9 (60.9 to 93.6) | 90.9 (69.4 to 109) | 65.8 (48.7 to 83.4) | -4.3 (-12.5 to 5.2) | -5.2 (-17.6 to 9.2) | -2.9 (-13.4 to 9.1) |
|  |  | All ages | 2102.1 (1647.9 to 2519.5) | 1207.1 (906.8 to 1432.7) | 895 (646.1 to 1146.8) | 2086.2 (1632.2 to 2524.4) | 1186.8 (909 to 1424.2) | 899.4 (660.1 to 1152.7) | -0.8 (-9.1 to 8.8) | -1.7 (-14.1 to 12.8) | 0.5 (-10.5 to 13) |
|  | Deaths | Age-standardized | 4.1 (2.9 to 4.8) | 5.7 (3.9 to 6.9) | 2.6 (1.8 to 3.2) | 3.8 (2.8 to 4.5) | 5.3 (3.7 to 6.6) | 2.4 (1.6 to 3.1) | -7.4 (-19.6 to 5.8) | -7.3 (-23.4 to 11.6) | -6.7 (-22.3 to 12.5) |
|  |  | All ages | 93 (65.8 to 108.3) | 62.8 (44.1 to 75.9) | 30.2 (20.7 to 37.1) | 88.4 (64.5 to 104.5) | 59.5 (42.3 to 73.7) | 28.9 (19.1 to 36.8) | -4.9 (-17.6 to 9) | -5.2 (-21.8 to 14.6) | -4.2 (-20.8 to 16.4) |
| Golestan | Incidence | Age-standardized | 40.9 (30.4 to 54.5) | 37.2 (28 to 50.1) | 45 (33 to 59.3) | 41 (30.1 to 54.8) | 37.3 (28 to 50.1) | 45.3 (32.9 to 60) | 0.2 (-4.3 to 5.2) | 0.3 (-6.4 to 6.3) | 0.6 (-5.9 to 6.7) |
|  |  | All ages | 563.3 (434 to 738.4) | 269.1 (205.7 to 359.9) | 294.2 (225.3 to 386.2) | 595.2 (457 to 776.6) | 288.1 (221 to 384.2) | 307.1 (232.1 to 401.5) | 5.7 (0.9 to 10.6) | 7.1 (0.2 to 13.9) | 4.4 (-2.5 to 11.1) |
|  | Prevalence | Age-standardized | 426.7 (325.1 to 561.8) | 382.7 (288.1 to 504.9) | 474.1 (360.3 to 620.2) | 427.4 (325.3 to 559.3) | 384 (294.8 to 506.3) | 476.5 (359.2 to 617.6) | 0.2 (-4.1 to 4.7) | 0.3 (-6 to 6.6) | 0.5 (-6 to 7) |
|  |  | All ages | 5529.6 (4328.7 to 7248.7) | 2603.9 (1995.1 to 3471.7) | 2925.8 (2283.3 to 3795.9) | 5790.5 (4533.3 to 7529.2) | 2777.6 (2147.5 to 3650.7) | 3012.9 (2338 to 3925.6) | 4.7 (0.5 to 9.2) | 6.7 (-0.1 to 13.9) | 3 (-4 to 9.5) |
|  | DALYs (Disability-Adjusted Life Years) | Age-standardized | 83.1 (64.4 to 99.6) | 94.9 (72.4 to 113.3) | 71.2 (52.6 to 90) | 80.1 (61.7 to 97.7) | 90.2 (68.6 to 109.2) | 69.5 (51.8 to 88) | -3.6 (-8.8 to 2.9) | -4.9 (-12.7 to 4.1) | -2.5 (-10.9 to 6.7) |
|  |  | All ages | 1016.1 (799 to 1221.5) | 590 (453 to 703.5) | 426.1 (318.8 to 539.2) | 1011.4 (784.8 to 1237.7) | 590.4 (451.3 to 718.7) | 421 (312.1 to 536.5) | -0.5 (-6.2 to 6.7) | 0.1 (-8.6 to 10.3) | -1.2 (-10.6 to 8.4) |
|  | Deaths | Age-standardized | 3.9 (2.9 to 4.5) | 5.2 (3.7 to 6.2) | 2.7 (1.8 to 3.4) | 3.7 (2.7 to 4.5) | 4.9 (3.5 to 6) | 2.6 (1.7 to 3.2) | -5.3 (-12.2 to 3) | -6.6 (-16 to 4.1) | -5.1 (-17.9 to 9.8) |
|  |  | All ages | 41.8 (30.6 to 48.1) | 27.7 (19.6 to 32.8) | 14.1 (9 to 17.6) | 39.7 (28.6 to 47) | 26.7 (19.3 to 32.7) | 13 (8.6 to 16.1) | -5 (-12.3 to 3.7) | -3.5 (-13.8 to 8.1) | -7.9 (-20.3 to 6.6) |
| Hamadan | Incidence | Age-standardized | 39.2 (29.3 to 51.9) | 35.3 (26.4 to 47) | 43 (32.2 to 57) | 39.3 (29.2 to 53.1) | 35.4 (26.2 to 47.5) | 43.3 (32.1 to 58.8) | 0.3 (-4.3 to 5) | 0.1 (-7.2 to 7) | 0.6 (-6.9 to 7.1) |
|  |  | All ages | 681.1 (517 to 888.7) | 301.3 (226.3 to 398.7) | 379.8 (290.5 to 493.7) | 706.4 (534.3 to 928.7) | 313.9 (233.4 to 418.2) | 392.4 (297.9 to 516.1) | 3.7 (-1.2 to 8.9) | 4.2 (-3.1 to 11) | 3.3 (-4.2 to 10.4) |
|  | Prevalence | Age-standardized | 410.3 (313.6 to 531.6) | 363.9 (274.9 to 473.3) | 452.1 (344.5 to 587.2) | 411.3 (314.2 to 546.3) | 364.1 (276 to 485.5) | 454.8 (345.9 to 601.3) | 0.2 (-4.4 to 5) | 0.1 (-7 to 6.4) | 0.6 (-6.4 to 7) |
|  |  | All ages | 7092.2 (5467.4 to 9163.5) | 3036.5 (2294.7 to 3960.7) | 4055.7 (3149.3 to 5266.2) | 7297.7 (5648.9 to 9604.4) | 3150.6 (2409.4 to 4163.3) | 4147.1 (3209.7 to 5390.4) | 2.9 (-1.8 to 7.9) | 3.8 (-3.2 to 10) | 2.3 (-4.3 to 8.7) |
|  | DALYs (Disability-Adjusted Life Years) | Age-standardized | 69.9 (53.8 to 86) | 83.4 (59.6 to 101.9) | 59.7 (44.4 to 77.5) | 69 (52.5 to 84.6) | 81.9 (61.4 to 100.2) | 58.9 (43.5 to 76.1) | -1.3 (-8.5 to 7.3) | -1.8 (-13 to 11) | -1.3 (-10.7 to 10.2) |
|  |  | All ages | 1222.2 (945.3 to 1498.4) | 670.1 (481.5 to 821.7) | 552.1 (409.6 to 713) | 1226 (931.9 to 1508.3) | 674.9 (508.4 to 825.9) | 551.1 (409.1 to 699.9) | 0.3 (-6.9 to 9.1) | 0.7 (-11.1 to 13.5) | -0.2 (-9.6 to 11) |
|  | Deaths | Age-standardized | 3 (2.1 to 3.6) | 4.6 (3 to 5.6) | 1.9 (1.3 to 2.4) | 3 (2.1 to 3.6) | 4.5 (2.9 to 5.6) | 1.8 (1.3 to 2.4) | -1.9 (-13.3 to 11.1) | -2.1 (-17.1 to 15.7) | -3.4 (-20.7 to 17.8) |
|  |  | All ages | 54.8 (37.3 to 65.5) | 34.9 (22.8 to 42.7) | 19.9 (12.8 to 25.7) | 53.6 (36.8 to 64.1) | 34.7 (22.5 to 43.2) | 18.9 (12.5 to 24.6) | -2.2 (-13.5 to 10.4) | -0.8 (-16.1 to 17.6) | -4.6 (-20.7 to 14.5) |
| Hormozgan | Incidence | Age-standardized | 40.4 (29.7 to 54.4) | 36.5 (26.5 to 48.9) | 44.3 (32.6 to 59.6) | 40.3 (29.8 to 53.8) | 36.4 (26.9 to 48.9) | 44.3 (32.5 to 59.5) | -0.2 (-4.8 to 4.4) | -0.1 (-4.9 to 5.8) | 0 (-6.8 to 6.7) |
|  |  | All ages | 421.4 (321.3 to 552.6) | 190.3 (145.3 to 249.5) | 231.1 (176.3 to 301.8) | 454.1 (350.1 to 591) | 206.7 (158.3 to 269.9) | 247.3 (188.7 to 321.2) | 7.7 (3.3 to 12.4) | 8.6 (3.1 to 15.4) | 7 (-0.1 to 13.6) |
|  | Prevalence | Age-standardized | 421.2 (322.7 to 552.3) | 375.6 (287.1 to 496.4) | 466.3 (355.2 to 607.6) | 420.6 (323.4 to 550) | 375.4 (287.2 to 491.4) | 466.2 (357.6 to 609.9) | -0.2 (-4.6 to 3.7) | -0.1 (-5.4 to 5.8) | 0 (-7.4 to 6.1) |
|  |  | All ages | 4142.9 (3221.7 to 5371.5) | 1834.8 (1413.9 to 2401.2) | 2308.1 (1791.2 to 2983) | 4413.5 (3450.2 to 5720.2) | 1980.9 (1531.4 to 2590.7) | 2432.5 (1913.8 to 3107) | 6.5 (1.8 to 10.7) | 8 (1.8 to 14.6) | 5.4 (-2.4 to 11.2) |
|  | DALYs (Disability-Adjusted Life Years) | Age-standardized | 75 (56.9 to 91.6) | 83 (63.4 to 99.9) | 67.1 (49.7 to 84.6) | 72.8 (56.5 to 89.3) | 80.4 (59.8 to 97.6) | 65.2 (48.3 to 83.2) | -2.8 (-10.9 to 5.8) | -3.2 (-13.8 to 10.5) | -2.9 (-12.9 to 7.6) |
|  |  | All ages | 698.6 (536.2 to 859.5) | 377.3 (286.4 to 460.4) | 321.3 (240.1 to 408.4) | 714.3 (553.1 to 878.7) | 387.7 (291.7 to 474) | 326.6 (241.5 to 415.4) | 2.3 (-6.3 to 11.3) | 2.8 (-8.5 to 17) | 1.6 (-9.1 to 14.1) |
|  | Deaths | Age-standardized | 3.5 (2.5 to 4.1) | 4.5 (3.2 to 5.3) | 2.6 (1.7 to 3.2) | 3.3 (2.4 to 4) | 4.3 (2.9 to 5.1) | 2.4 (1.5 to 3.1) | -4.6 (-16.3 to 8.1) | -4.1 (-18.6 to 14.6) | -6.6 (-23.7 to 14.3) |
|  |  | All ages | 29 (20.9 to 33.7) | 18.1 (12.9 to 21.5) | 10.9 (7.4 to 13.6) | 28.3 (20.3 to 33.6) | 17.9 (12.2 to 21.5) | 10.4 (6.6 to 13.5) | -2.4 (-14.8 to 11.6) | -0.9 (-16.6 to 19.5) | -4.8 (-23.2 to 17.2) |
| Ilam | Incidence | Age-standardized | 39.2 (28.9 to 52.6) | 35.7 (25.8 to 47.7) | 43 (31.9 to 57.4) | 39.3 (29.3 to 52.2) | 35.6 (26.1 to 47.9) | 43.3 (32.3 to 57.3) | 0.1 (-4.5 to 4.2) | -0.2 (-7.7 to 6.4) | 0.6 (-5.5 to 7.5) |
|  |  | All ages | 173.3 (131.3 to 226.7) | 80.9 (60 to 106.2) | 92.4 (69.7 to 121.7) | 185.1 (140.3 to 242.6) | 87.5 (65 to 115.8) | 97.6 (74.2 to 126.3) | 6.8 (2 to 11.6) | 8.2 (0.8 to 15.2) | 5.6 (-0.9 to 11.6) |
|  | Prevalence | Age-standardized | 410.2 (312.4 to 536.1) | 368.6 (278.5 to 482.2) | 454.5 (345.5 to 599.3) | 409.5 (310.3 to 536.3) | 367.4 (275.1 to 482.9) | 456.5 (347.7 to 594.7) | -0.2 (-4.4 to 4.1) | -0.3 (-6.6 to 6.2) | 0.4 (-5.4 to 6.4) |
|  |  | All ages | 1703.8 (1304.5 to 2209.8) | 784.6 (597.9 to 1019.7) | 919.1 (705 to 1184.4) | 1804.5 (1391 to 2341.8) | 846.9 (642.2 to 1105.5) | 957.7 (738.8 to 1232.2) | 5.9 (1.7 to 10.1) | 7.9 (1.2 to 14.9) | 4.2 (-1.6 to 10) |
|  | DALYs (Disability-Adjusted Life Years) | Age-standardized | 75.3 (60.2 to 91.1) | 79.5 (64.4 to 96.3) | 70.3 (52.9 to 87.6) | 72.7 (58.1 to 88) | 76.7 (61.9 to 92.9) | 67.8 (51 to 84.7) | -3.4 (-10.1 to 3.8) | -3.5 (-13.3 to 6.9) | -3.7 (-13.1 to 6) |
|  |  | All ages | 283.9 (223.8 to 343.7) | 152.9 (123.8 to 185.8) | 131.1 (98.3 to 163.5) | 288.9 (229 to 353.8) | 158.6 (126.4 to 194.7) | 130.3 (96.4 to 164.7) | 1.7 (-5.5 to 9.1) | 3.7 (-7.9 to 15.1) | -0.6 (-10.2 to 9.4) |
|  | Deaths | Age-standardized | 3.7 (2.9 to 4.3) | 4.2 (3.2 to 4.9) | 3.1 (2.1 to 3.8) | 3.5 (2.7 to 4) | 4 (3.1 to 4.8) | 2.8 (1.8 to 3.6) | -5.5 (-14.7 to 4.9) | -4.6 (-16.6 to 9.1) | -8.3 (-23.6 to 7.3) |
|  |  | All ages | 11.3 (9.1 to 13.1) | 6.7 (5.3 to 7.9) | 4.6 (3.2 to 5.7) | 11 (8.8 to 12.7) | 6.8 (5.3 to 8.1) | 4.2 (2.7 to 5.3) | -2.6 (-12.4 to 8) | 1.2 (-12.2 to 16.2) | -8.2 (-23.8 to 8.2) |
| Isfahan | Incidence | Age-standardized | 39 (28.7 to 51.8) | 35.1 (26.1 to 46.3) | 42.6 (31 to 57) | 39.1 (29.2 to 52) | 35.1 (26.3 to 46.9) | 42.9 (32.1 to 56.9) | 0.2 (-4.5 to 4.6) | -0.1 (-7 to 6.1) | 0.6 (-6.1 to 6.6) |
|  |  | All ages | 1903.4 (1437.9 to 2500.6) | 835.1 (632.3 to 1091.9) | 1068.3 (800.6 to 1408.9) | 2008.7 (1544.2 to 2653.1) | 884.2 (674.9 to 1164.7) | 1124.5 (859.8 to 1469.1) | 5.5 (0.6 to 10.2) | 5.9 (-1.6 to 12.6) | 5.3 (-1.3 to 11.3) |
|  | Prevalence | Age-standardized | 408.4 (310.5 to 534.8) | 363.1 (274.9 to 468.2) | 449.2 (340.5 to 595.1) | 409.4 (318.7 to 536.3) | 362.7 (279.1 to 474.6) | 451.9 (348 to 586.8) | 0.2 (-4.1 to 4.8) | -0.1 (-7.6 to 5.7) | 0.6 (-6.3 to 6.7) |
|  |  | All ages | 19285.8 (14815.6 to 25108.4) | 8274.9 (6337.2 to 10780.8) | 11010.9 (8462.3 to 14404.9) | 20192.4 (15904.1 to 26297.1) | 8702.3 (6753.9 to 11381.9) | 11490.1 (9025.8 to 14917.7) | 4.7 (0.3 to 9.5) | 5.2 (-2.6 to 10.9) | 4.4 (-2.4 to 10.8) |
|  | DALYs (Disability-Adjusted Life Years) | Age-standardized | 70.9 (55 to 86.3) | 85.3 (66.4 to 101.1) | 59.5 (43.9 to 76.1) | 68.2 (52.4 to 84.2) | 81.7 (61.6 to 98.9) | 57.5 (41.8 to 74.6) | -3.7 (-12.2 to 5.1) | -4.1 (-16.4 to 9.7) | -3.3 (-14.5 to 7.7) |
|  |  | All ages | 3205 (2484.8 to 3894.1) | 1764.9 (1375.7 to 2108.2) | 1440.1 (1057.7 to 1847.2) | 3196.4 (2473.1 to 3976.3) | 1758.8 (1350.5 to 2136) | 1437.6 (1039.5 to 1870.5) | -0.3 (-9 to 8.7) | -0.3 (-13.3 to 13.7) | -0.2 (-11.7 to 11.3) |
|  | Deaths | Age-standardized | 3.3 (2.4 to 3.9) | 5 (3.5 to 6) | 2 (1.3 to 2.5) | 3.1 (2.2 to 3.7) | 4.7 (3.3 to 5.8) | 1.8 (1.2 to 2.4) | -6.9 (-19.1 to 5.7) | -6 (-22.3 to 13) | -8.8 (-25.6 to 11.3) |
|  |  | All ages | 138.3 (101 to 162.6) | 91.9 (65.7 to 111.5) | 46.4 (30.5 to 58) | 130.8 (94 to 156.7) | 88 (62.5 to 109.4) | 42.8 (28.3 to 56.1) | -5.4 (-18.2 to 7.8) | -4.2 (-21.2 to 15.5) | -7.9 (-24.9 to 12.6) |
| Kerman | Incidence | Age-standardized | 41 (30.4 to 54.6) | 37 (27.6 to 49.2) | 45.1 (33.3 to 59.4) | 41.2 (30.5 to 54.8) | 37.1 (27.5 to 49.6) | 45.3 (33.5 to 60.4) | 0.3 (-3.6 to 4.3) | 0.3 (-5.5 to 5.7) | 0.4 (-5.7 to 6.3) |
|  |  | All ages | 909.3 (699.7 to 1186) | 410.9 (316 to 532.2) | 498.4 (383 to 646.1) | 982.2 (747.3 to 1277.6) | 445.4 (336.9 to 586.3) | 536.7 (410.2 to 695.6) | 8 (3.9 to 12.5) | 8.4 (2.3 to 15.1) | 7.7 (1.2 to 13.4) |
|  | Prevalence | Age-standardized | 428.5 (328.4 to 558.1) | 381.8 (292.6 to 498.9) | 474.7 (359.1 to 619.7) | 429.9 (328.1 to 558) | 383.1 (290.2 to 498.7) | 476.8 (364.2 to 619.7) | 0.3 (-3.3 to 4.8) | 0.3 (-5.1 to 6.8) | 0.4 (-5.5 to 6.1) |
|  |  | All ages | 8947 (6975.8 to 11629.5) | 3985.2 (3087.1 to 5251.5) | 4961.8 (3827.6 to 6453.1) | 9606.4 (7418.8 to 12372.7) | 4307.5 (3322.8 to 5623.2) | 5299 (4102 to 6832.5) | 7.4 (3.4 to 12) | 8.1 (2.7 to 15) | 6.8 (0.7 to 12.4) |
|  | DALYs (Disability-Adjusted Life Years) | Age-standardized | 86 (66.6 to 104.6) | 99 (73.8 to 119.7) | 73.1 (54.3 to 91.4) | 83 (62.9 to 100.8) | 95.2 (71.3 to 114.5) | 70.7 (51.1 to 91.1) | -3.6 (-11.8 to 4.7) | -3.8 (-15.4 to 10.6) | -3.3 (-13.4 to 8.6) |
|  |  | All ages | 1578.6 (1233.3 to 1922.5) | 873.5 (653.2 to 1066.9) | 705.2 (516.2 to 891.2) | 1632.6 (1241.6 to 2009.2) | 906.8 (681 to 1107.3) | 725.8 (527 to 938.5) | 3.4 (-5.7 to 12.6) | 3.8 (-9 to 19.8) | 2.9 (-8.1 to 15.8) |
|  | Deaths | Age-standardized | 4.6 (3.2 to 5.4) | 6.2 (4.3 to 7.4) | 3.1 (2 to 3.8) | 4.3 (3 to 5.2) | 5.8 (4 to 7.1) | 2.9 (1.8 to 3.6) | -6.6 (-17.7 to 5.6) | -6 (-20.3 to 11.8) | -7.5 (-21.8 to 9.3) |
|  |  | All ages | 68.8 (48.7 to 80.4) | 44.4 (30.8 to 53.7) | 24.4 (15.9 to 29.7) | 68.6 (48.6 to 82.5) | 44.9 (31 to 55.3) | 23.7 (14.7 to 29.7) | -0.3 (-13.6 to 14.2) | 1 (-16 to 23.2) | -2.6 (-19.5 to 17.1) |
| Kermanshah | Incidence | Age-standardized | 40.4 (29.6 to 54.1) | 36.5 (26.7 to 48.5) | 44.3 (32.6 to 59.7) | 40.5 (29.9 to 54.6) | 36.5 (27 to 49.4) | 44.6 (33 to 60.5) | 0.2 (-4 to 4.9) | 0 (-6.5 to 6.7) | 0.7 (-5.3 to 6.3) |
|  |  | All ages | 725 (543.1 to 953) | 329.9 (246.6 to 437.4) | 395.1 (297.3 to 521.2) | 767.2 (577.5 to 1026) | 352.8 (264.5 to 469.4) | 414.4 (312 to 552.5) | 5.8 (1.4 to 10.6) | 6.9 (0 to 13.6) | 4.9 (-0.9 to 11) |
|  | Prevalence | Age-standardized | 423.5 (325.5 to 558.4) | 376.2 (286.7 to 493.7) | 467.1 (358.3 to 613.2) | 423.9 (324.5 to 562.4) | 376 (285.9 to 492.8) | 469.6 (355.7 to 628.3) | 0.1 (-3.9 to 4.6) | 0 (-6.1 to 5.9) | 0.5 (-4.8 to 6.6) |
|  |  | All ages | 7312.8 (5685.4 to 9472.1) | 3229.9 (2471.7 to 4213) | 4083 (3160 to 5287.7) | 7678.2 (5939.5 to 9972.4) | 3446 (2644.7 to 4521.8) | 4232.2 (3262 to 5563.8) | 5 (0.8 to 9.5) | 6.7 (0.2 to 12.8) | 3.7 (-2 to 9.8) |
|  | DALYs (Disability-Adjusted Life Years) | Age-standardized | 76.6 (58.5 to 95.1) | 91.4 (67.1 to 110.7) | 64.9 (49.3 to 84.9) | 75.2 (57.6 to 92.9) | 89.1 (66.9 to 110.5) | 63.7 (47.7 to 82.6) | -1.9 (-10.7 to 7.5) | -2.5 (-15.9 to 12.3) | -1.8 (-11.5 to 10) |
|  |  | All ages | 1274.4 (980.7 to 1577.4) | 708.3 (524.1 to 862.4) | 566.1 (430.9 to 740.9) | 1298.5 (995.3 to 1616.8) | 730.4 (549.1 to 916) | 568.1 (424.3 to 730.3) | 1.9 (-7.2 to 11.9) | 3.1 (-10.9 to 18.7) | 0.4 (-9.9 to 12.6) |
|  | Deaths | Age-standardized | 3.6 (2.5 to 4.2) | 5.2 (3.7 to 6.4) | 2.3 (1.5 to 2.9) | 3.5 (2.4 to 4.2) | 5.1 (3.5 to 6.2) | 2.2 (1.3 to 2.8) | -3.3 (-16 to 11.8) | -3.4 (-19.4 to 17.3) | -5 (-21.7 to 16.8) |
|  |  | All ages | 54.9 (38.1 to 64.9) | 35.2 (24.5 to 43.1) | 19.8 (12.7 to 24.9) | 54.2 (38.2 to 65.1) | 35.5 (24.7 to 44) | 18.7 (11.4 to 23.7) | -1.3 (-14.5 to 14.3) | 0.9 (-16.4 to 23.2) | -5.1 (-21.6 to 16.3) |
| Khorasan-e-Razavi | Incidence | Age-standardized | 40.1 (29.7 to 53.2) | 36.3 (26.8 to 48.6) | 43.9 (32.5 to 57.9) | 40.1 (30.1 to 53.2) | 36.2 (27.1 to 48.6) | 44.2 (32.7 to 59) | 0.1 (-4.2 to 4.4) | -0.4 (-6.3 to 6.3) | 0.6 (-6.5 to 9.2) |
|  |  | All ages | 2005.4 (1531.7 to 2609) | 919.2 (700 to 1229.7) | 1086.1 (819.5 to 1415.5) | 2102.5 (1612.8 to 2774.6) | 964.3 (732.7 to 1273.1) | 1138.1 (861.7 to 1501.1) | 4.8 (0 to 9.3) | 4.9 (-1.3 to 11.3) | 4.8 (-1.8 to 12.7) |
|  | Prevalence | Age-standardized | 417.6 (319.4 to 544.4) | 373.7 (283.8 to 493.1) | 462.3 (354.1 to 594.8) | 418.2 (320.4 to 549.3) | 372.6 (283.2 to 487.9) | 465.2 (357.4 to 607.5) | 0.2 (-4.3 to 4.4) | -0.3 (-6.6 to 6.8) | 0.6 (-5.6 to 8.4) |
|  |  | All ages | 20088.2 (15438.1 to 25883.1) | 9077.4 (6901.4 to 12012.8) | 11010.7 (8476 to 13972) | 20908.4 (16145.8 to 26951.6) | 9474.2 (7288.2 to 12368.4) | 11434.2 (8731.8 to 14795.8) | 4.1 (-0.5 to 8.6) | 4.4 (-1.9 to 11.1) | 3.8 (-2.6 to 11.4) |
|  | DALYs (Disability-Adjusted Life Years) | Age-standardized | 79.9 (62.2 to 96.1) | 91.5 (70.6 to 111) | 68 (50.9 to 84.8) | 77.2 (59.8 to 92.9) | 88.5 (66.8 to 106.6) | 65.6 (48 to 82.7) | -3.4 (-12.1 to 5.9) | -3.4 (-15.7 to 10.6) | -3.5 (-13.6 to 8) |
|  |  | All ages | 3586.3 (2802.6 to 4316.9) | 2035.6 (1565.2 to 2468.1) | 1550.7 (1170.5 to 1928.3) | 3590.4 (2785.7 to 4338.4) | 2047.6 (1557.2 to 2485.2) | 1542.9 (1112.9 to 1948.4) | 0.1 (-8.9 to 10) | 0.6 (-11.9 to 15.2) | -0.5 (-12.2 to 11.5) |
|  | Deaths | Age-standardized | 4 (2.9 to 4.7) | 5.3 (3.9 to 6.4) | 2.7 (1.7 to 3.4) | 3.8 (2.6 to 4.6) | 5.1 (3.4 to 6.2) | 2.5 (1.5 to 3.2) | -5.4 (-16.8 to 7.6) | -4.4 (-19.6 to 14.2) | -7.7 (-22.5 to 10.2) |
|  |  | All ages | 157 (112.5 to 185.7) | 103.7 (77 to 126.3) | 53.4 (33.5 to 66.7) | 152.5 (107 to 185.1) | 102.6 (69.8 to 127.2) | 49.9 (30.8 to 64.1) | -2.9 (-15.3 to 10.7) | -1 (-17.4 to 18.9) | -6.5 (-22.2 to 12.9) |
| Khuzestan | Incidence | Age-standardized | 41 (30 to 54.6) | 37.1 (27.1 to 49.8) | 45.1 (33.1 to 59.6) | 41.1 (30.6 to 54.9) | 37.1 (27.7 to 48.8) | 45.2 (33.3 to 62.1) | 0 (-4.1 to 5.1) | 0 (-6.6 to 7.5) | 0.4 (-5.5 to 7.6) |
|  |  | All ages | 1340 (1016.1 to 1753.6) | 605.5 (453.1 to 801) | 734.5 (559.9 to 955.5) | 1415.9 (1086.2 to 1846.5) | 644.2 (485.7 to 847.3) | 771.7 (590.9 to 1007.2) | 5.7 (1.1 to 11.1) | 6.4 (-0.6 to 14.4) | 5.1 (-0.7 to 12.2) |
|  | Prevalence | Age-standardized | 430.5 (330.3 to 564) | 383.3 (290 to 505.8) | 475.9 (367.2 to 620.2) | 430.6 (328 to 564.4) | 383.2 (288.3 to 501.1) | 477.5 (365 to 628) | 0 (-4.6 to 4.8) | 0 (-6.9 to 7.4) | 0.3 (-5.2 to 7.1) |
|  |  | All ages | 13360.9 (10437.4 to 17304.2) | 5924.2 (4541.4 to 7779.1) | 7436.7 (5784.9 to 9634.2) | 13990.7 (10669.2 to 18158.8) | 6270.2 (4780.2 to 8209.6) | 7720.5 (5940.2 to 9895.1) | 4.7 (0.2 to 9.9) | 5.8 (-1.3 to 13.7) | 3.8 (-1.5 to 10.6) |
|  | DALYs (Disability-Adjusted Life Years) | Age-standardized | 76.2 (58.6 to 93.6) | 88.9 (65.7 to 106.9) | 64.6 (47.6 to 82.9) | 75 (57.4 to 92.5) | 87.6 (65.2 to 107.9) | 63.3 (46 to 82.1) | -1.5 (-9.1 to 7) | -1.4 (-12.2 to 11.7) | -2 (-10.6 to 6.8) |
|  |  | All ages | 2258.9 (1757.2 to 2766.5) | 1268.1 (952 to 1534.1) | 990.9 (723.6 to 1278.5) | 2302.6 (1761.2 to 2838.2) | 1304.8 (977.7 to 1607.4) | 997.7 (728.2 to 1284.7) | 1.9 (-6 to 11.2) | 2.9 (-8.4 to 16.3) | 0.7 (-8.4 to 10.6) |
|  | Deaths | Age-standardized | 3.4 (2.4 to 4) | 4.8 (3.3 to 5.8) | 2.2 (1.4 to 2.7) | 3.4 (2.4 to 4.1) | 4.8 (3.2 to 6) | 2.1 (1.4 to 2.7) | -2 (-13 to 10.6) | -1.1 (-15.6 to 17.3) | -4.5 (-19.4 to 11.1) |
|  |  | All ages | 91 (63.6 to 106.3) | 60.2 (42.1 to 72) | 30.8 (19.8 to 37.9) | 90.4 (63.7 to 109.8) | 61 (42.1 to 76.1) | 29.4 (19.1 to 37.9) | -0.6 (-12.1 to 12.5) | 1.4 (-13.8 to 21) | -4.5 (-19.9 to 11.8) |
| Kohgiluyeh and Boyer-Ahmad | Incidence | Age-standardized | 40.2 (30 to 53.9) | 36.1 (26.7 to 48.7) | 43.8 (32.5 to 58.7) | 40.4 (29.9 to 53.8) | 36.1 (26.8 to 48.5) | 44.1 (32.7 to 58.7) | 0.3 (-4.1 to 4.5) | 0 (-6.9 to 7.4) | 0.8 (-5.5 to 6.8) |
|  |  | All ages | 204.9 (154.9 to 270.8) | 86.7 (65.6 to 115.9) | 118.2 (88.7 to 156.1) | 214.2 (162.7 to 281.4) | 91.3 (69.4 to 121.4) | 122.9 (92.9 to 161.6) | 4.5 (0.4 to 8.7) | 5.3 (-1.4 to 12.6) | 4 (-1.9 to 9.7) |
|  | Prevalence | Age-standardized | 422.8 (323.2 to 554.7) | 374.2 (285.5 to 493.6) | 463 (354.4 to 608.3) | 424.3 (324.4 to 554) | 374.4 (284.4 to 493.3) | 466.2 (356.6 to 605.9) | 0.3 (-3.8 to 4.3) | 0.1 (-6.6 to 7.1) | 0.7 (-5.8 to 6.2) |
|  |  | All ages | 2094 (1611.6 to 2721.8) | 857.7 (657.6 to 1125.3) | 1236.3 (945.6 to 1617.8) | 2175 (1676.8 to 2828.6) | 899.2 (692.8 to 1178.7) | 1275.8 (980.9 to 1651.1) | 3.9 (-0.1 to 8) | 4.8 (-1.7 to 11.6) | 3.2 (-3.2 to 8.7) |
|  | DALYs (Disability-Adjusted Life Years) | Age-standardized | 66.9 (51.9 to 83.6) | 79.3 (59.5 to 97.2) | 58.2 (43.3 to 76.4) | 65.1 (49.7 to 80.4) | 77.2 (59.5 to 93.7) | 56.6 (41.4 to 73.4) | -2.6 (-11 to 6.2) | -2.6 (-14.4 to 11.2) | -2.8 (-12.3 to 7.7) |
|  |  | All ages | 322.8 (250.4 to 404.2) | 167.4 (127.5 to 206.6) | 155.4 (115.7 to 202.6) | 325.3 (249.3 to 403.3) | 170.6 (133.6 to 206.6) | 154.7 (113.6 to 198.9) | 0.8 (-7.6 to 10.1) | 1.9 (-10.9 to 16.1) | -0.4 (-11 to 10.1) |
|  | Deaths | Age-standardized | 2.9 (2 to 3.4) | 4.3 (2.9 to 5.3) | 1.8 (1.2 to 2.3) | 2.7 (2 to 3.3) | 4.2 (2.9 to 5.2) | 1.7 (1.1 to 2.2) | -5.2 (-17.5 to 8.6) | -3.5 (-20.9 to 15.1) | -8.1 (-24.9 to 13.2) |
|  |  | All ages | 12.8 (8.9 to 15.2) | 8 (5.5 to 9.8) | 4.8 (3.1 to 6) | 12.6 (9.2 to 15.4) | 8.1 (5.6 to 10) | 4.6 (3 to 6) | -1.6 (-14.8 to 13.1) | 1 (-17.8 to 21.4) | -5.8 (-23.7 to 16.4) |
| Kurdistan | Incidence | Age-standardized | 40.1 (29.7 to 54.1) | 36.2 (26.5 to 48.5) | 43.9 (32.1 to 59.2) | 40.3 (29.9 to 54.6) | 36.4 (27.1 to 49.7) | 44.2 (32.6 to 59.3) | 0.6 (-3.6 to 5.2) | 0.7 (-5.2 to 7.7) | 0.6 (-5.1 to 6.9) |
|  |  | All ages | 559.4 (421.7 to 742) | 253.6 (189.1 to 336.7) | 305.7 (228.3 to 403.3) | 600.1 (453.7 to 793.9) | 274.3 (206.9 to 369.5) | 325.8 (245.6 to 425.8) | 7.3 (2.7 to 12.1) | 8.1 (1.9 to 16) | 6.6 (0.5 to 12.7) |
|  | Prevalence | Age-standardized | 419.5 (317.4 to 558.8) | 373.9 (283.8 to 494.4) | 463.6 (350.8 to 617) | 421.6 (322.9 to 553.5) | 376 (283.2 to 499.7) | 466.6 (358.1 to 606.8) | 0.5 (-3.8 to 4.8) | 0.6 (-5.9 to 8.1) | 0.6 (-5.3 to 7.2) |
|  |  | All ages | 5671.6 (4320.6 to 7442.3) | 2520.7 (1928.7 to 3311) | 3150.9 (2399.8 to 4167.6) | 6039.4 (4649.4 to 7887.3) | 2711.3 (2056.1 to 3578.7) | 3328.1 (2562.7 to 4287.6) | 6.5 (1.9 to 11) | 7.6 (0.7 to 15.2) | 5.6 (-0.6 to 12.6) |
|  | DALYs (Disability-Adjusted Life Years) | Age-standardized | 72.7 (55.4 to 90.7) | 81.5 (61.3 to 99.9) | 64.8 (48 to 83.5) | 71 (54.1 to 88.6) | 79.7 (59.5 to 98.5) | 63.1 (46.5 to 81.2) | -2.4 (-9.8 to 4.6) | -2.2 (-12.7 to 9.6) | -2.7 (-12.2 to 7.8) |
|  |  | All ages | 956.8 (735.7 to 1200.9) | 519 (393.4 to 640.1) | 437.7 (324.2 to 561.4) | 981.4 (754.3 to 1228.8) | 536.4 (400.8 to 666.8) | 445 (329.5 to 580.2) | 2.6 (-5.4 to 10) | 3.3 (-8 to 16.2) | 1.7 (-8.4 to 13) |
|  | Deaths | Age-standardized | 3.3 (2.3 to 3.9) | 4.4 (3 to 5.3) | 2.3 (1.5 to 3) | 3.2 (2.1 to 3.8) | 4.3 (3 to 5.3) | 2.2 (1.4 to 2.9) | -4.2 (-14.9 to 7.7) | -2.8 (-17.2 to 13.6) | -6.6 (-21.8 to 10) |
|  |  | All ages | 41.1 (28.4 to 48.7) | 25.7 (17.8 to 31.2) | 15.4 (9.6 to 20) | 40.8 (27.7 to 49.6) | 26 (18.3 to 32.1) | 14.8 (9.2 to 19.7) | -0.9 (-12 to 11.5) | 0.9 (-14.5 to 18.3) | -3.8 (-19.5 to 13.7) |
| Lorestan | Incidence | Age-standardized | 39.2 (29.3 to 52.3) | 35.7 (26.6 to 48) | 43.4 (32.2 to 58) | 39.2 (28.9 to 52.6) | 35.7 (26.5 to 47.5) | 43.6 (31.8 to 58.7) | 0 (-4.2 to 4.1) | 0 (-5.8 to 6.5) | 0.5 (-6.3 to 8) |
|  |  | All ages | 529.2 (406.6 to 698.9) | 259 (194.9 to 340.8) | 270.2 (206.9 to 357.1) | 561 (424.9 to 736.6) | 278.4 (210 to 364.7) | 282.6 (213.8 to 372.9) | 6 (1.1 to 10.6) | 7.5 (0.8 to 14.6) | 4.6 (-1.9 to 11.5) |
|  | Prevalence | Age-standardized | 410.3 (313.3 to 541.7) | 369.4 (276.8 to 486.2) | 458.9 (353.3 to 604.7) | 410.1 (313.3 to 535.9) | 369.9 (280.8 to 484.7) | 460.5 (352.5 to 607) | -0.1 (-4.5 to 4.4) | 0.1 (-6.1 to 6.7) | 0.4 (-6.1 to 6.6) |
|  |  | All ages | 5246.7 (4054.3 to 6893.1) | 2552.8 (1931.6 to 3375.2) | 2694 (2087.8 to 3504.9) | 5534.4 (4280.8 to 7205.7) | 2748.2 (2089.4 to 3594.6) | 2786.2 (2146.2 to 3596.5) | 5.5 (0.5 to 10.3) | 7.7 (0.7 to 14.9) | 3.4 (-3.1 to 10.2) |
|  | DALYs (Disability-Adjusted Life Years) | Age-standardized | 74.6 (58.5 to 90.9) | 77.1 (60.1 to 94.7) | 71 (53 to 89.6) | 71.3 (55 to 88) | 73.8 (55 to 91.9) | 67.6 (49.4 to 84.5) | -4.4 (-13.3 to 5) | -4.3 (-18.2 to 9.3) | -4.9 (-12.8 to 3.7) |
|  |  | All ages | 888.2 (689.6 to 1102.5) | 492.7 (385.7 to 611.9) | 395.5 (292.3 to 503.6) | 890.6 (678.8 to 1090.9) | 504.7 (377.3 to 630.9) | 385.9 (285.4 to 482.5) | 0.3 (-9 to 10.1) | 2.4 (-12.6 to 17.1) | -2.4 (-10.9 to 7.2) |
|  | Deaths | Age-standardized | 3.5 (2.6 to 4.1) | 4 (3 to 4.8) | 2.8 (1.7 to 3.5) | 3.3 (2.4 to 3.9) | 3.8 (2.7 to 4.6) | 2.5 (1.5 to 3.2) | -7.2 (-20.8 to 7.1) | -6.5 (-25 to 13.3) | -10.1 (-19.7 to 2.9) |
|  |  | All ages | 35.7 (26.5 to 41.8) | 22.4 (16.7 to 27) | 13.3 (7.8 to 16.3) | 34.1 (25.1 to 40.6) | 22.2 (15.5 to 27.2) | 11.9 (7.2 to 15.1) | -4.5 (-18.8 to 10.2) | -0.8 (-21.2 to 20.7) | -10.8 (-21 to 2.8) |
| Markazi | Incidence | Age-standardized | 40.2 (29.9 to 54.1) | 36.3 (26.6 to 48.6) | 44.3 (32.8 to 59) | 40.3 (29.9 to 54) | 36.3 (26.7 to 48.5) | 44.5 (33 to 60) | 0.1 (-4.3 to 4.9) | 0 (-6.2 to 5.6) | 0.5 (-5.7 to 7.7) |
|  |  | All ages | 600.2 (446.7 to 796) | 273 (201.6 to 363) | 327.1 (245.3 to 431) | 621 (470.2 to 823.7) | 284.3 (212.3 to 376.7) | 336.7 (255 to 446.8) | 3.5 (-1.2 to 8.1) | 4.1 (-2.3 to 9.8) | 2.9 (-3.3 to 10) |
|  | Prevalence | Age-standardized | 421.3 (324.2 to 554) | 374.8 (282.5 to 487.8) | 467 (357.5 to 609.5) | 422.4 (324.5 to 558.4) | 375.5 (285.4 to 495.9) | 469.7 (357.1 to 626.8) | 0.3 (-4 to 5.9) | 0.2 (-5.8 to 6.1) | 0.6 (-5.6 to 7.4) |
|  |  | All ages | 6331.8 (4877.9 to 8304.6) | 2816.6 (2124.9 to 3666.9) | 3515.2 (2682.5 to 4586) | 6522.8 (5041.5 to 8556.8) | 2926 (2235.4 to 3870.1) | 3596.7 (2743.2 to 4757.8) | 3 (-1.4 to 8.2) | 3.9 (-2.3 to 9.8) | 2.3 (-3.8 to 9.3) |
|  | DALYs (Disability-Adjusted Life Years) | Age-standardized | 69.5 (53.9 to 85.9) | 81.1 (63.4 to 98.3) | 59.3 (44.2 to 78) | 67.7 (52.3 to 84.3) | 78.7 (60.9 to 97.9) | 57.7 (43.5 to 75.7) | -2.5 (-11 to 5.9) | -2.9 (-14.1 to 8.3) | -2.7 (-14.1 to 8.4) |
|  |  | All ages | 1062.9 (830.1 to 1309.3) | 605 (472.6 to 729.9) | 457.9 (343.5 to 598.6) | 1063.2 (825.5 to 1316.5) | 610.2 (474 to 755.3) | 453 (344 to 590.6) | 0 (-8.9 to 8.5) | 0.9 (-10.7 to 12.8) | -1.1 (-12.8 to 10.3) |
|  | Deaths | Age-standardized | 3.1 (2.2 to 3.7) | 4.5 (3.3 to 5.4) | 1.9 (1.2 to 2.4) | 3 (2.2 to 3.5) | 4.3 (3 to 5.3) | 1.8 (1.1 to 2.2) | -4.5 (-16.9 to 8.1) | -4.1 (-18.8 to 12.5) | -7.4 (-24.2 to 14.3) |
|  |  | All ages | 49.1 (35.5 to 58.6) | 33.1 (24.2 to 40) | 16.1 (10.3 to 20.5) | 48.3 (35.3 to 56.5) | 33.1 (23.3 to 40.5) | 15.1 (9.6 to 19.2) | -1.8 (-14.5 to 10.6) | 0.2 (-15.2 to 17.1) | -5.9 (-22.8 to 15.2) |
| Mazandaran | Incidence | Age-standardized | 41.7 (31.1 to 55.9) | 37.6 (28.1 to 50.3) | 45.8 (34.3 to 62) | 41.8 (31.2 to 56.1) | 37.6 (28 to 50.5) | 46 (34 to 62.1) | 0.2 (-4.5 to 5.3) | -0.1 (-6.1 to 6.8) | 0.6 (-5.8 to 6.6) |
|  |  | All ages | 1431.8 (1097.3 to 1886.4) | 654.4 (493.7 to 853.5) | 777.4 (598.3 to 1033.7) | 1527.4 (1166.4 to 2027.6) | 699.5 (524.9 to 923.8) | 827.9 (630.8 to 1101.9) | 6.7 (2.2 to 12.3) | 6.9 (0.6 to 14.8) | 6.5 (0.6 to 13.5) |
|  | Prevalence | Age-standardized | 438.1 (335.3 to 576.2) | 391 (296 to 510.8) | 485.1 (372.3 to 635.2) | 439.5 (335.2 to 578.2) | 390.7 (294.2 to 512.3) | 488.7 (372.1 to 647.2) | 0.3 (-4 to 5.4) | -0.1 (-5.9 to 7.6) | 0.7 (-5.4 to 6.3) |
|  |  | All ages | 14554.9 (11308.8 to 18879.8) | 6535.9 (5001 to 8553.9) | 8019 (6198.1 to 10371.6) | 15410.1 (11957.9 to 20069.4) | 6939 (5292.6 to 9042.9) | 8471.1 (6532.8 to 11006) | 5.9 (1.7 to 11) | 6.2 (0.2 to 14.4) | 5.6 (-0.4 to 11.4) |
|  | DALYs (Disability-Adjusted Life Years) | Age-standardized | 73.9 (57.8 to 91.2) | 84.1 (65.7 to 102.5) | 63.8 (47.3 to 82.4) | 71.9 (55.5 to 89.6) | 81.7 (62.7 to 102.3) | 62.2 (46 to 80.5) | -2.7 (-11.9 to 6.9) | -2.9 (-15.7 to 10.8) | -2.6 (-12.9 to 8.8) |
|  |  | All ages | 2386.5 (1875.5 to 2938.4) | 1344.9 (1057.7 to 1631.8) | 1041.6 (773.9 to 1335.3) | 2427.8 (1875.9 to 3019.2) | 1370 (1058.9 to 1707.3) | 1057.8 (778.5 to 1366.1) | 1.7 (-8 to 11.3) | 1.9 (-11.8 to 16.3) | 1.6 (-9.3 to 13.8) |
|  | Deaths | Age-standardized | 3.3 (2.4 to 3.9) | 4.4 (3.3 to 5.5) | 2.1 (1.5 to 2.7) | 3.1 (2.3 to 3.9) | 4.3 (3 to 5.5) | 2 (1.4 to 2.5) | -4.7 (-18.3 to 9.8) | -3.9 (-22.7 to 15.8) | -6.9 (-23.3 to 15) |
|  |  | All ages | 99.3 (74 to 118.2) | 66.4 (50 to 82.4) | 32.8 (22.4 to 41) | 96.9 (72.5 to 120.5) | 65.8 (47.2 to 85.3) | 31.1 (22.2 to 39) | -2.4 (-16.5 to 12.5) | -1 (-20.8 to 19.5) | -5.2 (-21.9 to 17.3) |
| North Khorasan | Incidence | Age-standardized | 40.8 (30.5 to 54.3) | 36.7 (27 to 49.5) | 44.6 (33.3 to 58.8) | 41 (30.3 to 54.8) | 36.8 (27.3 to 49.4) | 45 (33 to 60.2) | 0.5 (-3.5 to 5.4) | 0.3 (-5.5 to 7.6) | 0.9 (-5.6 to 8.7) |
|  |  | All ages | 274.6 (210.2 to 362.1) | 121.2 (91.3 to 160.4) | 153.5 (116.8 to 200.7) | 284.1 (217.6 to 374.4) | 126.4 (96.2 to 166) | 157.8 (118.6 to 206.9) | 3.5 (-1.4 to 8.3) | 4.3 (-2 to 12.4) | 2.8 (-3.2 to 9.9) |
|  | Prevalence | Age-standardized | 426.2 (327.6 to 555) | 377.2 (287.9 to 496.1) | 469.1 (357.8 to 613.7) | 428.3 (326.9 to 565.1) | 378.6 (290.3 to 497.2) | 472.8 (358 to 620.9) | 0.5 (-3.9 to 5.1) | 0.4 (-5.4 to 8.1) | 0.8 (-5.4 to 7.2) |
|  |  | All ages | 2786.5 (2150.1 to 3608.2) | 1185 (915.3 to 1550.6) | 1601.5 (1236.2 to 2072.8) | 2860.3 (2202.5 to 3698.6) | 1230.1 (958.7 to 1605) | 1630.2 (1252.4 to 2124.4) | 2.6 (-2.1 to 7.2) | 3.8 (-2 to 11.4) | 1.8 (-4.3 to 7.8) |
|  | DALYs (Disability-Adjusted Life Years) | Age-standardized | 74.5 (58.9 to 90.7) | 88.3 (68.2 to 108) | 63.3 (47.5 to 81.1) | 72.7 (56.5 to 89.6) | 85.9 (65.3 to 105.2) | 61.7 (45.4 to 81) | -2.4 (-11 to 6.3) | -2.7 (-13.8 to 9.2) | -2.4 (-12.1 to 7.8) |
|  |  | All ages | 477.8 (379.4 to 580.5) | 261.4 (202.9 to 319.8) | 216.4 (162.1 to 276.4) | 473.1 (366.3 to 581.4) | 260.6 (200.6 to 322.7) | 212.4 (157.3 to 277.6) | -1 (-9.8 to 7.6) | -0.3 (-12.1 to 12.3) | -1.8 (-11.6 to 9) |
|  | Deaths | Age-standardized | 3.2 (2.4 to 3.8) | 4.6 (3.4 to 5.7) | 2.1 (1.5 to 2.6) | 3.1 (2.2 to 3.8) | 4.5 (3.1 to 5.7) | 2 (1.3 to 2.5) | -4.5 (-16.4 to 8.6) | -3.7 (-18.4 to 13.1) | -6.9 (-22.9 to 11.5) |
|  |  | All ages | 19.7 (14.6 to 23.3) | 12.4 (9.2 to 15.3) | 7.2 (5.1 to 8.9) | 18.7 (13.6 to 23) | 12.1 (8.5 to 15.4) | 6.7 (4.6 to 8.5) | -4.7 (-16.7 to 8.4) | -2.7 (-17.6 to 14.3) | -8.1 (-23.7 to 9.4) |
| Qazvin | Incidence | Age-standardized | 38.8 (28.6 to 52.1) | 35.2 (26 to 46.5) | 42.8 (31.4 to 57.9) | 38.9 (28.7 to 52.6) | 35.2 (26 to 47.2) | 43 (31.5 to 58.7) | 0.3 (-4.1 to 4.8) | 0.2 (-5.9 to 7.4) | 0.5 (-6.3 to 7.7) |
|  |  | All ages | 405.2 (307.2 to 535.7) | 189 (143.5 to 246) | 216.3 (163.9 to 288.8) | 428.9 (329.7 to 568.9) | 201.1 (151.5 to 266.1) | 227.8 (174.6 to 302.2) | 5.8 (1.1 to 11) | 6.4 (0.1 to 13.5) | 5.3 (-1 to 12.2) |
|  | Prevalence | Age-standardized | 403.8 (308.2 to 527.8) | 362.4 (276.4 to 474.4) | 450.3 (343 to 590.2) | 404.9 (308.2 to 534.3) | 363.2 (275.2 to 483.3) | 452.6 (344.4 to 596.7) | 0.3 (-4.3 to 5) | 0.2 (-5.3 to 7.9) | 0.5 (-6 to 6.4) |
|  |  | All ages | 4028.2 (3111.5 to 5211.7) | 1874.4 (1438 to 2433.6) | 2153.8 (1660.1 to 2789.3) | 4232.8 (3280.4 to 5539.4) | 1983.6 (1500.4 to 2622.8) | 2249.2 (1729.4 to 2942) | 5.1 (0 to 10.1) | 5.8 (0.2 to 13.7) | 4.4 (-2.3 to 10.7) |
|  | DALYs (Disability-Adjusted Life Years) | Age-standardized | 74.5 (57.3 to 90.7) | 76.8 (58.1 to 91.4) | 71.2 (52 to 89.3) | 71.4 (54.9 to 89.4) | 73.5 (56 to 91) | 68 (50.4 to 85.6) | -4.2 (-11.8 to 4.5) | -4.3 (-15 to 8.4) | -4.4 (-12.8 to 3.4) |
|  |  | All ages | 699.7 (536.6 to 855.3) | 379.1 (288.7 to 456) | 320.6 (235.1 to 402.8) | 695.4 (536.8 to 865) | 378.8 (288.3 to 468.8) | 316.6 (233.8 to 399) | -0.6 (-8.8 to 8.6) | -0.1 (-11.4 to 13) | -1.3 (-10.6 to 7.4) |
|  | Deaths | Age-standardized | 3.6 (2.6 to 4.3) | 4.1 (2.9 to 5) | 3 (1.9 to 3.7) | 3.4 (2.4 to 4.1) | 3.9 (2.7 to 4.8) | 2.7 (1.7 to 3.5) | -6.7 (-17.7 to 7.3) | -5.9 (-20 to 12.1) | -9.2 (-21.9 to 4.1) |
|  |  | All ages | 30.4 (21.6 to 35.8) | 19 (13.4 to 22.8) | 11.3 (7 to 14) | 28.7 (20.6 to 34.9) | 18.3 (13 to 22.6) | 10.3 (6.4 to 13.1) | -5.6 (-17.2 to 9) | -3.6 (-18.4 to 15.4) | -9 (-21.9 to 4.9) |
| Qom | Incidence | Age-standardized | 39.3 (29.3 to 52.5) | 35.3 (26.1 to 47.3) | 42.9 (31.5 to 57.4) | 39.4 (29.4 to 52.7) | 35.4 (26.1 to 47.4) | 43.2 (32.3 to 57.9) | 0.4 (-3.8 to 4.7) | 0.1 (-6 to 6.9) | 0.7 (-5.3 to 8) |
|  |  | All ages | 375.8 (287 to 491.3) | 162.5 (123.4 to 216.9) | 213.3 (161.9 to 278.9) | 407.2 (312.8 to 533.9) | 176.7 (134.1 to 233.5) | 230.5 (177.2 to 301.7) | 8.4 (4 to 13.2) | 8.8 (1.9 to 15.6) | 8 (2.6 to 14.9) |
|  | Prevalence | Age-standardized | 413.6 (317.8 to 543.7) | 365.3 (274.5 to 479.4) | 453.2 (347.1 to 592.8) | 415.1 (320.2 to 541.4) | 366.2 (279.7 to 482.1) | 456.2 (349.6 to 595.9) | 0.4 (-4.3 to 4.4) | 0.3 (-6.3 to 6.3) | 0.6 (-5.3 to 7.2) |
|  |  | All ages | 3737.3 (2879.3 to 4876.5) | 1571.5 (1188.7 to 2065.3) | 2165.9 (1664.8 to 2814.4) | 4020.3 (3129.6 to 5231.8) | 1702.6 (1302.2 to 2238.7) | 2317.7 (1803 to 2959) | 7.6 (3.2 to 12.4) | 8.3 (1.3 to 14.8) | 7 (1.2 to 14.1) |
|  | DALYs (Disability-Adjusted Life Years) | Age-standardized | 73.5 (56.1 to 89.8) | 100.8 (71.5 to 118.3) | 60.1 (44.6 to 77.5) | 72.7 (54.6 to 88.7) | 98.1 (70.8 to 117.2) | 58.6 (43.2 to 76.3) | -1.1 (-8.9 to 7.4) | -2.7 (-13 to 7.9) | -2.5 (-11.5 to 7.1) |
|  |  | All ages | 618.8 (471.9 to 761.9) | 338.1 (254.7 to 407.7) | 280.7 (209.6 to 360) | 651.9 (495.9 to 797.7) | 362.1 (268.4 to 436.7) | 289.9 (212 to 374.7) | 5.3 (-3.4 to 14.5) | 7.1 (-5.3 to 20.7) | 3.3 (-6.6 to 13.5) |
|  | Deaths | Age-standardized | 3.6 (2.4 to 4.2) | 6.7 (4.3 to 7.9) | 2.1 (1.3 to 2.6) | 3.5 (2.4 to 4.2) | 6.4 (4 to 7.7) | 2 (1.2 to 2.5) | -1.8 (-13.2 to 11.9) | -4.2 (-15.4 to 7.8) | -6.8 (-23.1 to 12.8) |
|  |  | All ages | 26.2 (17.5 to 31.4) | 17.2 (11.1 to 20.9) | 9 (5.5 to 11.3) | 27.2 (18.9 to 32.4) | 18.5 (12.1 to 22.6) | 8.8 (5.6 to 11) | 3.8 (-8.7 to 19.3) | 7.5 (-8.2 to 25.1) | -3.2 (-20.4 to 17.5) |
| Semnan | Incidence | Age-standardized | 39.8 (29.4 to 53.3) | 36.1 (26.5 to 48.9) | 43.8 (32.3 to 58.6) | 39.8 (29.4 to 53.1) | 35.9 (26.1 to 48.2) | 43.9 (32.4 to 58.9) | -0.1 (-4.9 to 5.3) | -0.4 (-5.9 to 6.2) | 0.3 (-6.8 to 7.2) |
|  |  | All ages | 252.6 (191.3 to 334.8) | 118.1 (88.3 to 157.6) | 134.5 (101.6 to 180.2) | 267.2 (203.4 to 355) | 124.6 (92.2 to 165.4) | 142.5 (107.9 to 188.2) | 5.8 (0.7 to 11.4) | 5.5 (-0.1 to 12.1) | 6 (-1.4 to 13.8) |
|  | Prevalence | Age-standardized | 415.1 (317.5 to 548.2) | 372.9 (283.5 to 492) | 463 (354.8 to 607.6) | 414.7 (318.4 to 545.3) | 372.2 (283.5 to 491) | 463.5 (352.8 to 605.7) | -0.1 (-4.8 to 5.5) | -0.2 (-5.7 to 6) | 0.1 (-6.6 to 7.3) |
|  |  | All ages | 2544 (1948.1 to 3317.6) | 1189.6 (904.5 to 1565.8) | 1354.4 (1036.2 to 1775.7) | 2667.5 (2068.7 to 3458.5) | 1248.3 (954.6 to 1638) | 1419.2 (1098.2 to 1844.1) | 4.9 (0.5 to 10.5) | 4.9 (-0.7 to 11.2) | 4.8 (-1.9 to 12.7) |
|  | DALYs (Disability-Adjusted Life Years) | Age-standardized | 74.4 (58.6 to 90.6) | 78.5 (61.4 to 94.8) | 68.5 (51.2 to 86.3) | 68.7 (53.2 to 85.7) | 72.5 (56.2 to 90.1) | 63.1 (46.5 to 80.2) | -7.6 (-16.1 to 1.6) | -7.7 (-19.6 to 5.2) | -7.9 (-18 to 2.2) |
|  |  | All ages | 434.8 (340.3 to 534.4) | 244.8 (191.5 to 295) | 190 (143.6 to 241.6) | 415.9 (322.3 to 520.4) | 233.4 (181.5 to 290.3) | 182.5 (134 to 235) | -4.3 (-13.4 to 5.2) | -4.7 (-17.1 to 9) | -3.9 (-13.9 to 7.3) |
|  | Deaths | Age-standardized | 3.6 (2.7 to 4.2) | 4.2 (3.1 to 5.1) | 2.7 (1.8 to 3.3) | 3.1 (2.3 to 3.8) | 3.8 (2.7 to 4.7) | 2.3 (1.5 to 2.9) | -12.6 (-24.3 to 1.4) | -11 (-25.8 to 6.8) | -16.6 (-32.4 to -0.1) |
|  |  | All ages | 19.2 (14.6 to 22.5) | 12.8 (9.4 to 15.3) | 6.4 (4.3 to 7.8) | 16.8 (12.5 to 20.2) | 11.4 (8.1 to 14.3) | 5.4 (3.6 to 6.8) | -12.4 (-24.5 to 1.7) | -10.6 (-26 to 7.6) | -16.1 (-31.6 to 0.7) |
| Sistan and Baluchistan | Incidence | Age-standardized | 38.8 (28.6 to 52.1) | 35 (25.8 to 46.5) | 42.2 (31 to 57.2) | 38.7 (28.2 to 51.9) | 34.9 (25.8 to 46.5) | 42.3 (31 to 56.7) | -0.1 (-5.5 to 4.3) | -0.2 (-6.6 to 7) | 0.2 (-7.4 to 7.3) |
|  |  | All ages | 519.1 (395.7 to 681.2) | 226.9 (173.6 to 295.7) | 292.1 (220.1 to 383.6) | 541.1 (412.1 to 715.5) | 239.8 (183.6 to 310.7) | 301.4 (227.4 to 393.4) | 4.2 (-0.7 to 8.9) | 5.7 (-0.8 to 12.3) | 3.2 (-3.6 to 9.6) |
|  | Prevalence | Age-standardized | 399.3 (306.3 to 525) | 354.2 (267.7 to 464.8) | 440.1 (338.9 to 582) | 398.7 (306.5 to 522.2) | 353.9 (270 to 462.6) | 440.7 (337.3 to 577.7) | -0.1 (-5 to 4.6) | -0.1 (-6.3 to 6.2) | 0.1 (-6.6 to 6.4) |
|  |  | All ages | 5165.5 (4008.8 to 6717.1) | 2186.6 (1692 to 2866) | 2979 (2307.6 to 3857.8) | 5330.7 (4143.3 to 6913.7) | 2299.2 (1771.1 to 2970.6) | 3031.6 (2360 to 3949.4) | 3.2 (-1.6 to 8) | 5.1 (-1.1 to 11.3) | 1.8 (-4.8 to 7.9) |
|  | DALYs (Disability-Adjusted Life Years) | Age-standardized | 68.8 (51.2 to 84.9) | 77.7 (56.7 to 95.7) | 61 (44.6 to 77.4) | 68.7 (50.9 to 85.5) | 77.7 (53.5 to 97.6) | 60.5 (43 to 76.9) | -0.1 (-9.8 to 11.9) | 0 (-13.7 to 17.7) | -1 (-12.5 to 11.6) |
|  |  | All ages | 890.9 (661.9 to 1092.5) | 475.8 (348.5 to 588.5) | 415.1 (302.8 to 523.7) | 912.4 (678.6 to 1130.6) | 496.3 (347.2 to 623.9) | 416.1 (299.4 to 525.9) | 2.4 (-7.7 to 14.7) | 4.3 (-9.8 to 22.5) | 0.2 (-11.4 to 12.9) |
|  | Deaths | Age-standardized | 2.8 (1.7 to 3.4) | 3.7 (2.4 to 4.7) | 2 (1.2 to 2.5) | 2.8 (1.7 to 3.4) | 3.7 (2.3 to 4.8) | 2 (1.1 to 2.6) | 1.2 (-14.1 to 19.3) | 1.5 (-17.6 to 26.9) | -1.7 (-21.9 to 22.1) |
|  |  | All ages | 35.4 (22.4 to 43.2) | 21.7 (14.5 to 28) | 13.6 (7.8 to 17.4) | 35.9 (22.6 to 44.4) | 22.6 (14.3 to 29.5) | 13.2 (7.4 to 17.5) | 1.4 (-13.9 to 19.5) | 4.2 (-15.6 to 30.5) | -3 (-23 to 20.4) |
| South Khorasan | Incidence | Age-standardized | 38.2 (28.1 to 51.2) | 34.5 (25.3 to 46.2) | 42 (31 to 56.2) | 38.4 (28.2 to 51) | 34.6 (25.7 to 46.3) | 42.3 (30.8 to 56) | 0.5 (-3.9 to 4.5) | 0.3 (-5.7 to 7.3) | 0.8 (-4.7 to 6.8) |
|  |  | All ages | 270.3 (202 to 359.4) | 125.2 (93.4 to 164.4) | 145.1 (106.7 to 193.1) | 285.1 (212.6 to 374.4) | 132.6 (100 to 175.6) | 152.5 (112.4 to 200.1) | 5.5 (0.5 to 10.2) | 5.9 (-0.6 to 13.3) | 5.1 (-0.6 to 11.5) |
|  | Prevalence | Age-standardized | 396.6 (302.2 to 520.2) | 354.7 (268.9 to 464.5) | 441.7 (332.9 to 584) | 398.4 (303.8 to 520.4) | 356.4 (272.3 to 464.2) | 444.1 (338.2 to 584.9) | 0.4 (-3.8 to 5.2) | 0.5 (-5.3 to 8.5) | 0.5 (-5 to 5.9) |
|  |  | All ages | 2817.8 (2140.4 to 3711.2) | 1288.3 (980.1 to 1686.1) | 1529.5 (1151.4 to 2020.1) | 2938.5 (2241.8 to 3824.5) | 1354 (1044.8 to 1759.6) | 1584.6 (1206.2 to 2078.2) | 4.3 (-0.6 to 9.3) | 5.1 (-1 to 13) | 3.6 (-2.2 to 9.4) |
|  | DALYs (Disability-Adjusted Life Years) | Age-standardized | 65.2 (51.4 to 81) | 70.8 (55.8 to 86.4) | 59.7 (44.6 to 76.7) | 64.4 (49.9 to 80.4) | 70.2 (54.2 to 86.3) | 58.2 (43.7 to 75.3) | -1.2 (-8.9 to 6.7) | -0.8 (-12.7 to 11.2) | -2.4 (-12.1 to 7.1) |
|  |  | All ages | 470.6 (371.4 to 583.1) | 264.9 (209.3 to 322.4) | 205.7 (154.6 to 262.4) | 477.9 (369.7 to 596) | 272.5 (210.6 to 335.5) | 205.4 (154.1 to 267.9) | 1.6 (-6.4 to 9.8) | 2.9 (-9.6 to 15.3) | -0.1 (-9.3 to 9.1) |
|  | Deaths | Age-standardized | 2.9 (2.1 to 3.4) | 3.5 (2.6 to 4.3) | 2.2 (1.5 to 2.7) | 2.8 (2 to 3.4) | 3.5 (2.4 to 4.4) | 2.1 (1.4 to 2.6) | -1.8 (-13.5 to 10.4) | -0.3 (-16.3 to 16.9) | -6.6 (-21 to 10) |
|  |  | All ages | 21.5 (15.5 to 25.3) | 14 (10.1 to 17.1) | 7.5 (5.1 to 9.2) | 21.3 (14.8 to 26) | 14.3 (9.8 to 17.9) | 7 (4.7 to 9) | -0.9 (-12.7 to 11.6) | 2.1 (-14.3 to 19.4) | -6.5 (-20.9 to 10.4) |
| Tehran | Incidence | Age-standardized | 39.1 (29.2 to 52.4) | 35.1 (26 to 46.8) | 42.7 (31.6 to 57.3) | 39.2 (29 to 52.7) | 35.2 (26 to 47.9) | 42.9 (31.4 to 57.4) | 0.3 (-4.6 to 5.4) | 0.3 (-4.8 to 6.9) | 0.5 (-6.7 to 7.6) |
|  |  | All ages | 5279.7 (4040.4 to 6916.6) | 2262.4 (1714 to 2977.9) | 3017.3 (2275 to 3970.2) | 5680.5 (4266.8 to 7525.2) | 2454.7 (1858.9 to 3267.8) | 3225.9 (2401 to 4279.2) | 7.6 (2.6 to 12.7) | 8.5 (3 to 15.7) | 6.9 (-0.5 to 13.9) |
|  | Prevalence | Age-standardized | 412.2 (316.1 to 542.6) | 364.4 (278.6 to 473.7) | 452.4 (343.9 to 597.5) | 413.3 (317.9 to 543.5) | 365.6 (278 to 480.2) | 453.9 (349.3 to 588) | 0.3 (-4.8 to 5.3) | 0.3 (-4.9 to 6.6) | 0.3 (-6.9 to 6.9) |
|  |  | All ages | 54157.8 (42113 to 70008.6) | 22418 (17302.6 to 29165) | 31739.8 (24256.4 to 41618.8) | 58038.5 (45232.7 to 76182.8) | 24288.1 (18550.5 to 31855.8) | 33750.3 (26028.7 to 43851.8) | 7.2 (2 to 11.9) | 8.3 (2 to 15.2) | 6.3 (-1.1 to 13.2) |
|  | DALYs (Disability-Adjusted Life Years) | Age-standardized | 66.5 (51.4 to 82.6) | 73.8 (56.1 to 89.7) | 61.4 (44.6 to 80.6) | 63.9 (49.3 to 79.3) | 70.9 (53 to 86.3) | 59.3 (44 to 78.2) | -3.9 (-12.8 to 7.3) | -3.9 (-16.8 to 13.4) | -3.4 (-13.9 to 8.2) |
|  |  | All ages | 8626.2 (6685.1 to 10681.4) | 4236.6 (3240.2 to 5150.4) | 4389.7 (3195.2 to 5728.6) | 8800 (6771.1 to 10928.6) | 4338 (3230.1 to 5300.8) | 4461.9 (3324.6 to 5867.8) | 2 (-7.7 to 13.4) | 2.4 (-11.4 to 20.6) | 1.6 (-9.8 to 13.5) |
|  | Deaths | Age-standardized | 3 (2.2 to 3.6) | 3.9 (2.9 to 5) | 2.3 (1.5 to 3.1) | 2.8 (2 to 3.4) | 3.8 (2.7 to 4.7) | 2.1 (1.4 to 2.8) | -7 (-21.8 to 11) | -4.7 (-24.3 to 21.1) | -8.7 (-24.4 to 12.1) |
|  |  | All ages | 378.3 (280.1 to 461) | 204.9 (148.6 to 260.1) | 173.5 (114.1 to 232.7) | 366.8 (270.6 to 443.2) | 203.3 (145.4 to 256.3) | 163.6 (109.4 to 215.2) | -3 (-18.5 to 15.6) | -0.8 (-21.1 to 27) | -5.7 (-21.8 to 15.3) |
| West Azarbayejan | Incidence | Age-standardized | 38.5 (28.6 to 51.5) | 34.9 (25.7 to 47) | 42.3 (31.5 to 56.5) | 38.5 (28.6 to 51.4) | 34.9 (25.8 to 46.7) | 42.5 (31.4 to 56.7) | 0.2 (-4.3 to 5.4) | 0.1 (-5.6 to 6.9) | 0.5 (-5.5 to 9) |
|  |  | All ages | 936.3 (717.9 to 1231.7) | 437.3 (332.9 to 581.6) | 499 (381.5 to 657.8) | 992.9 (760.6 to 1303.7) | 464.6 (352.9 to 622.2) | 528.2 (407.4 to 691.2) | 6 (1.5 to 11.9) | 6.3 (0.1 to 13.9) | 5.9 (-0.3 to 14) |
|  | Prevalence | Age-standardized | 400.8 (307.8 to 522) | 358.4 (274.4 to 469.9) | 445.1 (340.5 to 578.8) | 401.8 (309 to 526) | 359 (275.1 to 475.1) | 447.3 (344.7 to 584.6) | 0.2 (-4.1 to 5.1) | 0.2 (-5.7 to 6.8) | 0.5 (-4.8 to 7.7) |
|  |  | All ages | 9140.9 (7068.4 to 11768.4) | 4234.7 (3223.7 to 5522.7) | 4906.2 (3786.6 to 6314.4) | 9613.2 (7515 to 12648.7) | 4469.8 (3476.9 to 5961.1) | 5143.4 (3966.3 to 6701.9) | 5.2 (0.6 to 10.3) | 5.6 (-0.4 to 12.5) | 4.8 (-1 to 11.5) |
|  | DALYs (Disability-Adjusted Life Years) | Age-standardized | 90.3 (67.2 to 105.7) | 108.7 (78.2 to 128.5) | 72.8 (52 to 89.5) | 86.4 (64.7 to 103.9) | 102.5 (73.3 to 123.5) | 70.3 (51.5 to 87.7) | -4.3 (-12.2 to 3.4) | -5.7 (-15.7 to 6.3) | -3.4 (-12.6 to 6.6) |
|  |  | All ages | 1711.1 (1320.1 to 2024.6) | 996.5 (755.8 to 1180.4) | 714.6 (529 to 883.8) | 1725.1 (1331.3 to 2093) | 1010.2 (753.7 to 1234.4) | 715 (522.2 to 899.4) | 0.8 (-7.5 to 10) | 1.4 (-10.1 to 15.2) | 0 (-9.9 to 10.5) |
|  | Deaths | Age-standardized | 5.2 (3.5 to 6.2) | 7.3 (4.9 to 8.9) | 3.3 (1.9 to 4.1) | 4.9 (3.3 to 5.9) | 6.7 (4.4 to 8.3) | 3.1 (1.8 to 4) | -6.8 (-16.6 to 3) | -8.4 (-20.5 to 6.7) | -6.1 (-21.3 to 9.4) |
|  |  | All ages | 75.4 (51.1 to 88.4) | 51.3 (34.4 to 61.8) | 24.1 (14.8 to 29.7) | 73.7 (51 to 89.5) | 51.1 (34 to 63.8) | 22.7 (14.1 to 29.4) | -2.1 (-14.4 to 9.8) | -0.4 (-15.7 to 18.7) | -5.8 (-22.3 to 11.4) |
| Yazd | Incidence | Age-standardized | 39.6 (29 to 53) | 35.8 (26.4 to 47.9) | 43.4 (31.7 to 57.6) | 39.7 (29.3 to 53.7) | 35.8 (26.5 to 47.9) | 43.7 (32.3 to 59.6) | 0.5 (-3.2 to 4.2) | 0 (-6.6 to 6.7) | 0.9 (-4.5 to 6.9) |
|  |  | All ages | 353 (266.1 to 464.5) | 157.9 (118 to 209.3) | 195.1 (148.1 to 253) | 364.5 (276.8 to 486.8) | 162.1 (122.9 to 214.7) | 202.4 (154 to 269.8) | 3.3 (-0.6 to 7.2) | 2.7 (-4.3 to 9.3) | 3.8 (-1.9 to 9.6) |
|  | Prevalence | Age-standardized | 412.7 (313.4 to 541.3) | 369.8 (279.9 to 484.4) | 458.4 (351 to 602.3) | 414.6 (317 to 547.8) | 370.1 (281.1 to 488.7) | 461.5 (351 to 608.2) | 0.5 (-3.5 to 4.5) | 0.1 (-6.6 to 6.5) | 0.7 (-5.2 to 6.5) |
|  |  | All ages | 3575.2 (2748.3 to 4632) | 1594.4 (1211.2 to 2096.1) | 1980.8 (1515.7 to 2568.8) | 3652.8 (2819.8 to 4760.5) | 1622.2 (1241 to 2141.8) | 2030.6 (1566.5 to 2622.3) | 2.2 (-1.8 to 6.7) | 1.7 (-5.4 to 8.5) | 2.5 (-3.5 to 8.8) |
|  | DALYs (Disability-Adjusted Life Years) | Age-standardized | 77.9 (60.9 to 94.8) | 86.9 (67 to 104) | 67.1 (50.8 to 84.6) | 73.2 (56.7 to 88.5) | 82.3 (63.8 to 102.1) | 62.6 (46.9 to 79.9) | -6 (-15.6 to 4) | -5.3 (-18.7 to 9) | -6.7 (-17.4 to 4.8) |
|  |  | All ages | 636.9 (501.1 to 774.2) | 364.1 (281.4 to 438.7) | 272.8 (205.7 to 345.5) | 604.8 (470.4 to 736.7) | 345.1 (266.5 to 429.4) | 259.7 (194 to 330.7) | -5 (-14.4 to 4.9) | -5.2 (-18.5 to 9) | -4.8 (-16.8 to 6.9) |
|  | Deaths | Age-standardized | 4 (2.9 to 4.7) | 5 (3.6 to 6.1) | 2.7 (1.8 to 3.4) | 3.6 (2.6 to 4.3) | 4.6 (3.3 to 5.8) | 2.3 (1.5 to 3) | -10.8 (-24.7 to 6.1) | -7.8 (-25.2 to 12.1) | -15.7 (-32.2 to 3.2) |
|  |  | All ages | 29.9 (21.7 to 35) | 20.4 (14.4 to 24.8) | 9.5 (6.1 to 11.8) | 26.5 (19.3 to 32.3) | 18.5 (13.3 to 23.3) | 8 (5.1 to 10.3) | -11.2 (-24.9 to 5.7) | -9.1 (-26.4 to 10.9) | -15.5 (-34.3 to 6) |
| Zanjan | Incidence | Age-standardized | 40.9 (30.3 to 55.1) | 37.1 (27.6 to 49.4) | 45.1 (33.2 to 61.3) | 41 (30.3 to 54.4) | 37.1 (27.6 to 50) | 45.2 (33.4 to 60.4) | 0.2 (-4 to 4) | 0.1 (-4.5 to 6.5) | 0.4 (-5.8 to 5.8) |
|  |  | All ages | 386.3 (289.4 to 515.5) | 181.4 (136.3 to 238.2) | 204.9 (152.8 to 275.1) | 404.6 (307.1 to 532.4) | 191.3 (144.5 to 253.3) | 213.3 (161 to 280.7) | 4.7 (0.4 to 8.6) | 5.4 (0.3 to 11.3) | 4.1 (-2.5 to 9.9) |
|  | Prevalence | Age-standardized | 428.1 (326.9 to 566.5) | 383.7 (292 to 510.4) | 477.2 (358.9 to 629.1) | 428.7 (329.8 to 564.2) | 384.6 (291.5 to 505.4) | 478.4 (368.4 to 629.4) | 0.1 (-4.2 to 3.8) | 0.2 (-5.3 to 5.7) | 0.2 (-6.5 to 6.2) |
|  |  | All ages | 3948.7 (3014.6 to 5194.4) | 1839.5 (1406.9 to 2432.8) | 2109.2 (1586.6 to 2762.5) | 4103.5 (3184.1 to 5355.7) | 1931 (1482.2 to 2516.2) | 2172.5 (1688.6 to 2840.8) | 3.9 (-0.4 to 7.8) | 5 (-0.9 to 10.7) | 3 (-3.7 to 8.3) |
|  | DALYs (Disability-Adjusted Life Years) | Age-standardized | 72.1 (56.1 to 89.7) | 74.2 (57.7 to 91.7) | 69.1 (51.6 to 87.5) | 70 (53.8 to 86.7) | 72.3 (55.1 to 89.5) | 66.5 (50.2 to 85.5) | -2.9 (-9.6 to 4.7) | -2.5 (-13 to 9.1) | -3.7 (-11.4 to 5.3) |
|  |  | All ages | 648.8 (505.1 to 808.8) | 351.6 (274.4 to 436.4) | 297.2 (224.2 to 379.4) | 647.5 (498.8 to 808.3) | 355 (270.4 to 439.4) | 292.5 (218.6 to 375.9) | -0.2 (-7.2 to 7.2) | 1 (-10 to 12.5) | -1.6 (-9.6 to 8) |
|  | Deaths | Age-standardized | 3.3 (2.3 to 3.9) | 3.7 (2.7 to 4.6) | 2.7 (1.8 to 3.2) | 3.1 (2.2 to 3.8) | 3.6 (2.5 to 4.6) | 2.4 (1.6 to 3) | -5 (-15.5 to 7.2) | -3.3 (-17.9 to 13.9) | -8.7 (-20.4 to 5.1) |
|  |  | All ages | 27.9 (19.9 to 33.3) | 17.5 (12.5 to 21.4) | 10.5 (6.8 to 12.7) | 26.8 (18.9 to 32.5) | 17.2 (11.9 to 21.8) | 9.6 (6.3 to 12) | -4.2 (-14.6 to 8.1) | -1.5 (-16.4 to 16.1) | -8.6 (-20.4 to 5.1) |

**Table S3.** All ages number and age‑standardized rate of disability-adjusted life years (DALYs) and deaths of atrial fibrillation and flutter attributable to risk factors by sex in 1990 and 2021 and overall percent change over 1990–2021 in Iran

| Location | Risk factor | Measure | Age, Metric | Year | | | | | | % Change (1990 to 2021) | | |
| --- | --- | --- | --- | --- | --- | --- | --- | --- | --- | --- | --- | --- |
|  |  |  |  | 1990 | | | 2021 | | |  |  |  |
|  |  |  |  | Both | Women | Men | Both | Women | Men | Both | Women | Men |
| Iran (Islamic Republic of) | All risk factors | DALYs (Disability-Adjusted Life Years) | Age-standardized | 22.6 (10.1 to 35.6) | 25.9 (11.5 to 41.7) | 18.9 (8.1 to 29.8) | 27.4 (14.7 to 40.8) | 32.2 (16.8 to 49.5) | 23 (12.1 to 35.3) | 21 (-2.1 to 61.4) | 24.1 (-6.6 to 66.3) | 21.9 (2.9 to 69.3) |
|  |  |  | All ages | 4046.2 (1834.1 to 6377.4) | 2210.8 (993.9 to 3499.2) | 1835.4 (827.8 to 2919.4) | 18252.5 (9774.3 to 27579.4) | 10305.9 (5381.3 to 15540.2) | 7946.6 (4210.4 to 12110.5) | 351.1 (274.3 to 481.3) | 366.2 (259.2 to 510.8) | 333 (268.3 to 470.6) |
|  |  | Deaths | Age-standardized | 1 (0.4 to 1.7) | 1.3 (0.6 to 2.2) | 0.7 (0.2 to 1.1) | 1.2 (0.6 to 1.8) | 1.7 (0.8 to 2.6) | 0.7 (0.4 to 1.1) | 17 (-13.8 to 71.4) | 27.6 (-12.7 to 85.1) | 13.1 (-12.7 to 97.3) |
|  |  |  | All ages | 129 (56.3 to 209.6) | 86.1 (38.5 to 144.7) | 42.9 (16.5 to 70.5) | 704.2 (368 to 1079) | 468.9 (228.4 to 722) | 235.3 (118.8 to 358.2) | 445.9 (303.9 to 686) | 444.8 (277.8 to 675.6) | 448.1 (316.8 to 818.7) |
|  | Diet high in sodium | DALYs (Disability-Adjusted Life Years) | Age-standardized | 0.4 (0 to 2.3) | 0.3 (0 to 2) | 0.5 (0 to 2.6) | 0.4 (0 to 2.4) | 0.3 (0 to 2.1) | 0.5 (0 to 2.7) | 0.7 (-86.9 to 356) | 0.2 (-99.5 to 2615.1) | 1 (-86.2 to 374.9) |
|  |  |  | All ages | 78 (0 to 443.5) | 25.8 (0 to 190) | 52.2 (0 to 263.7) | 264.2 (0 to 1594.9) | 91.1 (0 to 690.3) | 173.1 (0 to 940.5) | 238.6 (-55 to 1036.4) | 252.9 (-99.8 to 4560.2) | 231.5 (-57.4 to 1037.7) |
|  |  | Deaths | Age-standardized | 0 (0 to 0.1) | 0 (0 to 0.1) | 0 (0 to 0.1) | 0 (0 to 0.1) | 0 (0 to 0.1) | 0 (0 to 0.1) | -1.6 (-88.4 to 381.9) | 3.6 (-100 to 3113) | -6.9 (-88.7 to 425.1) |
|  |  |  | All ages | 2 (0 to 12.3) | 0.9 (0 to 6.9) | 1.1 (0 to 5.8) | 8.1 (0 to 56.5) | 3.6 (0 to 29.6) | 4.5 (0 to 27.8) | 308.9 (-60.8 to 1189.9) | 311.7 (-99.3 to 7053.5) | 306.8 (-61.7 to 1249.8) |
|  | High alcohol use | DALYs (Disability-Adjusted Life Years) | Age-standardized | 0 (0 to 0) | 0 (0 to 0) | 0 (0 to 0) | 0.2 (0.1 to 0.3) | 0 (0 to 0.1) | 0.3 (0.2 to 0.5) | NA | NA | NA |
|  |  |  | All ages | 0 (0 to 0) | 0 (0 to 0) | 0 (0 to 0) | 141 (87.4 to 211.6) | 11.5 (5.7 to 20) | 129.5 (80.8 to 195.2) | NA | NA | NA |
|  |  | Deaths | Age-standardized | 0 (0 to 0) | 0 (0 to 0) | 0 (0 to 0) | 0 (0 to 0) | 0 (0 to 0) | 0 (0 to 0) | NA | NA | NA |
|  |  |  | All ages | 0 (0 to 0) | 0 (0 to 0) | 0 (0 to 0) | 3.2 (1.8 to 4.9) | 0.4 (0.2 to 0.8) | 2.8 (1.5 to 4.2) | NA | NA | NA |
|  | High body-mass index | DALYs (Disability-Adjusted Life Years) | Age-standardized | 3.5 (1.4 to 6) | 5.4 (2.1 to 9.3) | 1.5 (0.6 to 2.9) | 10.5 (4.4 to 18.1) | 13.9 (5.9 to 23.9) | 7.4 (2.9 to 12.8) | 200.7 (80.4 to 354.7) | 157.8 (37.1 to 299.2) | 375.2 (198.8 to 904.3) |
|  |  |  | All ages | 706.5 (276.7 to 1173.3) | 523.9 (210.9 to 881.9) | 182.7 (69.5 to 329.9) | 7061.4 (2941.6 to 12080.8) | 4520.5 (1937.9 to 7735.9) | 2540.9 (1001.6 to 4465.5) | 899.4 (537 to 1280.2) | 762.9 (410.3 to 1114.4) | 1291 (800.1 to 2445.4) |
|  |  | Deaths | Age-standardized | 0.1 (0.1 to 0.3) | 0.2 (0.1 to 0.4) | 0 (0 to 0.1) | 0.5 (0.2 to 0.8) | 0.7 (0.3 to 1.2) | 0.3 (0.1 to 0.4) | 219.6 (66.3 to 494.4) | 198 (39.3 to 468.7) | 476.7 (198.5 to 1714) |
|  |  |  | All ages | 20.4 (8.1 to 35.2) | 17 (6.7 to 31.1) | 3.4 (1.1 to 6.5) | 273.6 (115.9 to 484.6) | 195.3 (81.9 to 340.9) | 78.3 (30.9 to 134.5) | 1241.6 (654.1 to 2101.8) | 1048.8 (479.9 to 1853.4) | 2208.2 (1226.6 to 5379.2) |
|  | High systolic blood pressure | DALYs (Disability-Adjusted Life Years) | Age-standardized | 19.6 (6.5 to 32.9) | 22.8 (7.7 to 38.5) | 15.9 (5 to 27.4) | 20.5 (6.8 to 34.5) | 24.5 (8.2 to 41.7) | 16.8 (5.6 to 29.4) | 4.7 (-12.1 to 23.1) | 7.7 (-14.5 to 29.8) | 5.2 (-8.9 to 28.8) |
|  |  |  | All ages | 3370.5 (1113.1 to 5682) | 1896.4 (641.9 to 3199.7) | 1474.1 (458.5 to 2552.3) | 13514.7 (4484.7 to 22734.4) | 7813.4 (2582.7 to 13294.2) | 5701.2 (1888.2 to 9843.1) | 301 (241.1 to 367.9) | 312 (229.8 to 391) | 286.8 (237.6 to 372.6) |
|  |  | Deaths | Age-standardized | 0.9 (0.3 to 1.6) | 1.2 (0.4 to 2.1) | 0.6 (0.2 to 1.1) | 0.9 (0.3 to 1.5) | 1.3 (0.5 to 2.2) | 0.6 (0.2 to 1) | -0.1 (-24.1 to 31.3) | 9.6 (-21.5 to 44.1) | -4.7 (-24.6 to 45.7) |
|  |  |  | All ages | 113.1 (38.1 to 193.5) | 76.3 (26.6 to 132.5) | 36.8 (10.9 to 64.8) | 537.5 (189.4 to 916.7) | 362.9 (126.4 to 617.3) | 174.6 (59.6 to 306.2) | 375.3 (260.9 to 520.2) | 375.5 (240.1 to 524.6) | 375.1 (268.3 to 616.1) |
|  | Lead exposure | DALYs (Disability-Adjusted Life Years) | Age-standardized | 3.8 (-0.5 to 9.8) | 4 (-0.6 to 10.4) | 3.6 (-0.5 to 8.9) | 4 (-0.6 to 10.1) | 4.3 (-0.6 to 10.8) | 3.8 (-0.5 to 9.4) | 5.2 (-11.5 to 26.2) | 7.1 (-16.6 to 32.9) | 6.1 (-8.6 to 32.9) |
|  |  |  | All ages | 674.1 (-90.6 to 1695.5) | 340.4 (-47.8 to 873.6) | 333.7 (-44.5 to 834.4) | 2604.4 (-350.4 to 6540) | 1330.8 (-180.3 to 3297.7) | 1273.6 (-170.1 to 3156.2) | 286.4 (226.7 to 362.4) | 291 (209.4 to 380) | 281.7 (229 to 374) |
|  |  | Deaths | Age-standardized | 0.2 (0 to 0.4) | 0.2 (0 to 0.5) | 0.1 (0 to 0.3) | 0.2 (0 to 0.5) | 0.3 (0 to 0.6) | 0.1 (0 to 0.3) | 8.8 (-16.3 to 43.7) | 18.8 (-15.7 to 58.9) | 3.8 (-17 to 63.7) |
|  |  |  | All ages | 22.1 (-3.4 to 55.4) | 13.6 (-2.2 to 34.9) | 8.4 (-1.3 to 21.5) | 111.9 (-16.7 to 273.9) | 68.2 (-10 to 166.1) | 43.7 (-7 to 107.2) | 406.6 (290.4 to 572.4) | 400 (254 to 561.7) | 417.4 (305.5 to 708.9) |
|  | Smoking | DALYs (Disability-Adjusted Life Years) | Age-standardized | 1.8 (1 to 2.8) | 0.6 (0.3 to 1) | 3 (1.7 to 4.6) | 1.9 (1.1 to 3) | 0.6 (0.3 to 1) | 3.2 (1.8 to 5.1) | 8.3 (-10.9 to 30.9) | 3.9 (-30.7 to 52.8) | 9.5 (-10.1 to 36.3) |
|  |  |  | All ages | 444 (253.9 to 692.2) | 72.6 (39.8 to 113.1) | 371.4 (210.7 to 580.6) | 1487.3 (822.5 to 2315.4) | 241.9 (126.5 to 399.2) | 1245.3 (688.9 to 1930.4) | 234.9 (178 to 298.5) | 233.3 (131.3 to 373.7) | 235.3 (176 to 312.8) |
|  |  | Deaths | Age-standardized | 0 (0 to 0.1) | 0 (0 to 0) | 0.1 (0 to 0.1) | 0 (0 to 0.1) | 0 (0 to 0) | 0.1 (0 to 0.1) | 7.1 (-23.7 to 63.5) | 6 (-44 to 97.8) | 1.5 (-29.4 to 64.6) |
|  |  |  | All ages | 7.9 (4.3 to 11.7) | 1.8 (0.9 to 2.8) | 6.1 (3.2 to 9.5) | 31.7 (17 to 49.7) | 6.8 (3.4 to 12.1) | 24.9 (13 to 39.6) | 301 (186.6 to 497.3) | 285 (121.4 to 564.9) | 305.5 (174.2 to 542.3) |
| Alborz | All risk factors | DALYs (Disability-Adjusted Life Years) | Age-standardized | 25.8 (11.6 to 41.6) | 30.5 (12.8 to 53.1) | 20.4 (8.9 to 32.8) | 30.5 (16.2 to 45.9) | 40.7 (20.5 to 63.5) | 25.3 (12.6 to 38.7) | 18.3 (-11.9 to 59.7) | 33.3 (-14.5 to 102.5) | 23.7 (-4.8 to 75.9) |
|  |  |  | All ages | 100.7 (47.8 to 156.7) | 55.1 (24.4 to 93.9) | 45.6 (21 to 72.4) | 595.4 (323.6 to 887.1) | 315 (160.6 to 487.8) | 280.4 (143.5 to 431.4) | 491.1 (359.2 to 671.5) | 471.5 (282.6 to 729.6) | 514.6 (389.5 to 740.1) |
|  |  | Deaths | Age-standardized | 1.2 (0.5 to 2.1) | 1.7 (0.7 to 3.3) | 0.6 (0.2 to 1.2) | 1.3 (0.6 to 2.1) | 2.5 (1.2 to 4.1) | 0.7 (0.3 to 1.2) | 10.8 (-29.8 to 76) | 46.1 (-19.9 to 152.4) | 17.5 (-26.3 to 105.7) |
|  |  |  | All ages | 3.1 (1.4 to 5.3) | 2.2 (0.9 to 4.2) | 0.9 (0.4 to 1.6) | 19.6 (9.8 to 30) | 13 (6.1 to 20.9) | 6.6 (3.1 to 10.6) | 530.8 (320.5 to 866.7) | 493.4 (242.2 to 890.8) | 621.2 (350.2 to 1147.5) |
|  | Diet high in sodium | DALYs (Disability-Adjusted Life Years) | Age-standardized | 0.4 (0 to 2.6) | 0.3 (0 to 2.4) | 0.5 (0 to 2.6) | 0.4 (0 to 2.5) | 0.3 (0 to 2.5) | 0.5 (0 to 2.8) | 1.4 (-98.1 to 867.4) | 2.6 (-9717.4 to 26427.6) | 0.7 (-98 to 916) |
|  |  |  | All ages | 1.9 (0 to 10.6) | 0.6 (0 to 4.7) | 1.2 (0 to 6.1) | 8.6 (0 to 50.2) | 2.8 (0 to 21.3) | 5.8 (0 to 30.7) | 362.6 (-90.3 to 4041.9) | 333.7 (-37674.2 to 89052.5) | 378 (-90.5 to 4041.7) |
|  |  | Deaths | Age-standardized | 0 (0 to 0.1) | 0 (0 to 0.1) | 0 (0 to 0.1) | 0 (0 to 0.1) | 0 (0 to 0.1) | 0 (0 to 0.1) | -3 (-99.1 to 1104.5) | 11.9 (-16635 to 21374.9) | -6.5 (-99.1 to 1119.7) |
|  |  |  | All ages | 0 (0 to 0.3) | 0 (0 to 0.2) | 0 (0 to 0.1) | 0.2 (0 to 1.4) | 0.1 (0 to 0.8) | 0.1 (0 to 0.7) | 383.3 (-94.8 to 4046.4) | 339.3 (-33044.4 to 97355.7) | 426.5 (-94.9 to 4066.2) |
|  | High alcohol use | DALYs (Disability-Adjusted Life Years) | Age-standardized | 0 (0 to 0) | 0 (0 to 0) | 0 (0 to 0) | 0.2 (0.1 to 0.4) | 0 (0 to 0.1) | 0.4 (0.2 to 0.6) | NA | NA | NA |
|  |  |  | All ages | 0 (0 to 0) | 0 (0 to 0) | 0 (0 to 0) | 5 (2.8 to 8.1) | 0.4 (0.1 to 0.7) | 4.6 (2.6 to 7.3) | NA | NA | NA |
|  |  | Deaths | Age-standardized | 0 (0 to 0) | 0 (0 to 0) | 0 (0 to 0) | 0 (0 to 0) | 0 (0 to 0) | 0 (0 to 0) | NA | NA | NA |
|  |  |  | All ages | 0 (0 to 0) | 0 (0 to 0) | 0 (0 to 0) | 0.1 (0 to 0.2) | 0 (0 to 0) | 0.1 (0 to 0.1) | NA | NA | NA |
|  | High body-mass index | DALYs (Disability-Adjusted Life Years) | Age-standardized | 5 (2 to 9.1) | 7.5 (2.9 to 14.5) | 2.3 (0.9 to 4.6) | 12.1 (5.2 to 21.5) | 18.8 (8.2 to 34.5) | 8 (3.1 to 14.4) | 142 (30.8 to 288) | 149.6 (21.4 to 325) | 246.2 (77.5 to 653) |
|  |  |  | All ages | 21.1 (8.6 to 35.9) | 15.2 (6.3 to 27.5) | 5.9 (2.2 to 11.4) | 236.8 (99.6 to 419.3) | 148.9 (66.8 to 263.7) | 87.9 (34.5 to 158.7) | 1019.7 (564.4 to 1490.6) | 877.7 (448.4 to 1403.6) | 1384.9 (735.8 to 2576.6) |
|  |  | Deaths | Age-standardized | 0.2 (0.1 to 0.5) | 0.4 (0.1 to 0.9) | 0.1 (0 to 0.1) | 0.5 (0.2 to 1) | 1.1 (0.4 to 2.1) | 0.2 (0.1 to 0.5) | 139.6 (4.8 to 421.1) | 199.7 (20 to 629.6) | 296.3 (80.2 to 1127.1) |
|  |  |  | All ages | 0.6 (0.3 to 1.3) | 0.5 (0.2 to 1.1) | 0.1 (0 to 0.2) | 8 (3.4 to 14.4) | 5.9 (2.5 to 10.7) | 2.1 (0.8 to 3.9) | 1152.8 (527.9 to 2107.9) | 999.6 (382.9 to 2100.3) | 1944.8 (840.9 to 4506.9) |
|  | High systolic blood pressure | DALYs (Disability-Adjusted Life Years) | Age-standardized | 22 (7 to 38.1) | 26.2 (8.2 to 48.9) | 17.1 (5.5 to 29.6) | 22.9 (7.8 to 38.1) | 30.4 (10.5 to 54.1) | 18.9 (6.2 to 32.5) | 4.2 (-24.2 to 41.2) | 16.3 (-25.1 to 79) | 10.4 (-17 to 49.3) |
|  |  |  | All ages | 82.7 (25.7 to 139.7) | 46.1 (14.5 to 82.9) | 36.5 (11.4 to 64) | 439.1 (145.5 to 738.4) | 233.6 (78.4 to 406.7) | 205.5 (68.8 to 358.9) | 431.3 (307.4 to 597.1) | 406.5 (235.2 to 644.6) | 462.6 (347 to 659.4) |
|  |  | Deaths | Age-standardized | 1.1 (0.3 to 1.9) | 1.5 (0.4 to 3) | 0.6 (0.2 to 1.1) | 1 (0.3 to 1.8) | 1.9 (0.6 to 3.4) | 0.6 (0.2 to 1) | -4.1 (-40.8 to 44.5) | 25.4 (-31.6 to 113.4) | 2.4 (-37.8 to 75.9) |
|  |  |  | All ages | 2.7 (0.8 to 4.8) | 1.9 (0.6 to 3.7) | 0.8 (0.2 to 1.4) | 14.8 (5 to 25.6) | 9.9 (3.2 to 17.7) | 5 (1.6 to 8.9) | 457.4 (259.2 to 725.8) | 420.8 (189.8 to 767.9) | 547.7 (302.8 to 998.5) |
|  | Lead exposure | DALYs (Disability-Adjusted Life Years) | Age-standardized | 3.2 (-0.4 to 8.2) | 3.7 (-0.5 to 9.6) | 2.7 (-0.4 to 6.9) | 3.4 (-0.5 to 8.6) | 4.4 (-0.6 to 11) | 3 (-0.4 to 7.5) | 6.3 (-16.2 to 35.5) | 18.4 (-20.9 to 67.2) | 13.5 (-10.8 to 49.1) |
|  |  |  | All ages | 12.2 (-1.6 to 31) | 6.5 (-0.9 to 16.7) | 5.7 (-0.8 to 14.4) | 61.4 (-8.3 to 157.9) | 30 (-4 to 77.3) | 31.4 (-4.3 to 79.1) | 401.9 (311.2 to 527.4) | 359.7 (215.4 to 525.8) | 450.1 (332.8 to 602.1) |
|  |  | Deaths | Age-standardized | 0.2 (0 to 0.4) | 0.2 (0 to 0.6) | 0.1 (0 to 0.2) | 0.2 (0 to 0.4) | 0.3 (0 to 0.8) | 0.1 (0 to 0.3) | 6.9 (-27.4 to 53.6) | 40.2 (-20.5 to 115) | 15.9 (-28.3 to 95.1) |
|  |  |  | All ages | 0.4 (-0.1 to 1) | 0.3 (0 to 0.7) | 0.1 (0 to 0.3) | 2.3 (-0.4 to 6) | 1.4 (-0.2 to 3.7) | 0.9 (-0.1 to 2.3) | 494.5 (308.3 to 750.4) | 435.3 (215.2 to 739.8) | 626.7 (365.4 to 1161.8) |
|  | Smoking | DALYs (Disability-Adjusted Life Years) | Age-standardized | 1.9 (1.1 to 3) | 0.8 (0.4 to 1.4) | 3.1 (1.7 to 4.9) | 2.2 (1.2 to 3.4) | 0.8 (0.4 to 1.5) | 3.4 (1.9 to 5.3) | 11.9 (-14.1 to 43.6) | 3.7 (-44.5 to 88.4) | 10.4 (-19.6 to 43.9) |
|  |  |  | All ages | 10.9 (6 to 16.9) | 1.9 (0.9 to 3.2) | 9 (4.9 to 14.4) | 54.4 (29.6 to 85.8) | 8.8 (4 to 15.3) | 45.6 (24.4 to 72.4) | 400.6 (294.3 to 528.4) | 373.4 (173.2 to 690.2) | 406.2 (288.5 to 560.6) |
|  |  | Deaths | Age-standardized | 0 (0 to 0.1) | 0 (0 to 0.1) | 0.1 (0 to 0.1) | 0.1 (0 to 0.1) | 0 (0 to 0.1) | 0.1 (0 to 0.1) | 12.4 (-34.8 to 85.9) | 14.3 (-59.1 to 224.7) | 2.3 (-42.7 to 77.4) |
|  |  |  | All ages | 0.2 (0.1 to 0.3) | 0 (0 to 0.1) | 0.1 (0.1 to 0.2) | 0.9 (0.5 to 1.5) | 0.2 (0.1 to 0.4) | 0.7 (0.3 to 1.2) | 414.8 (225 to 720.2) | 348.7 (116.1 to 839.4) | 438.8 (208.7 to 857.5) |
| Ardebil | All risk factors | DALYs (Disability-Adjusted Life Years) | Age-standardized | 21.6 (9 to 35.1) | 24.4 (10.6 to 41) | 19 (7.3 to 31.8) | 32.5 (16.8 to 49.1) | 33.8 (17 to 52.5) | 30.6 (15.6 to 46.7) | 50.4 (18 to 110.1) | 38.7 (-2.4 to 99.8) | 61.1 (26.4 to 141.4) |
|  |  |  | All ages | 74.7 (32.9 to 119.9) | 38.2 (16.4 to 64.5) | 36.5 (15.9 to 60.6) | 341.2 (177 to 513.4) | 192.2 (97.4 to 298.4) | 149 (76 to 227.5) | 356.6 (259.6 to 525.3) | 403.1 (256.1 to 617) | 307.9 (221.4 to 488.3) |
|  |  | Deaths | Age-standardized | 0.9 (0.3 to 1.5) | 1.2 (0.5 to 2.2) | 0.6 (0.2 to 1.1) | 1.4 (0.7 to 2.3) | 1.7 (0.8 to 2.7) | 1.1 (0.5 to 1.7) | 62.4 (10.7 to 165.9) | 47.9 (-9.1 to 145.9) | 74.4 (13.5 to 265.4) |
|  |  |  | All ages | 2.3 (0.9 to 3.9) | 1.4 (0.6 to 2.7) | 0.8 (0.3 to 1.5) | 13.7 (6.9 to 21.5) | 9.1 (4.2 to 14.5) | 4.7 (2.1 to 7.2) | 504.4 (314.1 to 875.9) | 534.2 (294.8 to 917.4) | 453.7 (259.4 to 1019.4) |
|  | Diet high in sodium | DALYs (Disability-Adjusted Life Years) | Age-standardized | 0.4 (0 to 2.2) | 0.3 (0 to 1.8) | 0.5 (0 to 2.6) | 0.4 (0 to 2.5) | 0.3 (0 to 2.1) | 0.6 (0 to 3.2) | 9.3 (-95.4 to 2126.2) | 7.4 (-89916 to 24305.5) | 18.4 (-95.1 to 2493.7) |
|  |  |  | All ages | 1.4 (0 to 7.9) | 0.4 (0 to 3) | 1 (0 to 5.1) | 4.5 (0 to 26.1) | 1.6 (0 to 12.2) | 2.8 (0 to 15.5) | 207.9 (-88.2 to 4410.9) | 270.1 (-3676765.2 to 35393.7) | 181.2 (-88.2 to 4595.1) |
|  |  | Deaths | Age-standardized | 0 (0 to 0.1) | 0 (0 to 0.1) | 0 (0 to 0.1) | 0 (0 to 0.1) | 0 (0 to 0.1) | 0 (0 to 0.1) | 18.6 (-97.8 to 3709.5) | 14.2 (-493.4 to 57836.4) | 26.3 (-98 to 3852.5) |
|  |  |  | All ages | 0 (0 to 0.2) | 0 (0 to 0.1) | 0 (0 to 0.1) | 0.1 (0 to 1) | 0.1 (0 to 0.5) | 0.1 (0 to 0.5) | 309 (-91.9 to 7737.5) | 364 (-11115.6 to 51157.4) | 271.7 (-92.3 to 7784.5) |
|  | High alcohol use | DALYs (Disability-Adjusted Life Years) | Age-standardized | 0 (0 to 0) | 0 (0 to 0) | 0 (0 to 0) | 0.2 (0.1 to 0.3) | 0 (0 to 0.1) | 0.4 (0.2 to 0.6) | NA | NA | NA |
|  |  |  | All ages | 0 (0 to 0) | 0 (0 to 0) | 0 (0 to 0) | 2.2 (1.2 to 3.4) | 0.2 (0.1 to 0.4) | 2 (1.1 to 3) | NA | NA | NA |
|  |  | Deaths | Age-standardized | 0 (0 to 0) | 0 (0 to 0) | 0 (0 to 0) | 0 (0 to 0) | 0 (0 to 0) | 0 (0 to 0) | NA | NA | NA |
|  |  |  | All ages | 0 (0 to 0) | 0 (0 to 0) | 0 (0 to 0) | 0.1 (0 to 0.1) | 0 (0 to 0) | 0 (0 to 0.1) | NA | NA | NA |
|  | High body-mass index | DALYs (Disability-Adjusted Life Years) | Age-standardized | 2.5 (0.9 to 4.5) | 4.3 (1.5 to 8.1) | 0.9 (0.2 to 1.9) | 10.5 (4.3 to 18) | 12.8 (5.1 to 22.7) | 7.7 (3 to 13.9) | 323.6 (129.9 to 684) | 196 (47.1 to 442.1) | 779.4 (351.1 to 2860.6) |
|  |  |  | All ages | 10.2 (3.7 to 17.3) | 7.9 (3.1 to 14.1) | 2.3 (0.7 to 4.4) | 111.1 (45.6 to 190.6) | 73.2 (29.5 to 129.9) | 37.9 (14.7 to 68.5) | 988.5 (568 to 1645.5) | 823.2 (416.8 to 1368.9) | 1562.7 (850.8 to 4493.2) |
|  |  | Deaths | Age-standardized | 0.1 (0 to 0.2) | 0.2 (0.1 to 0.4) | 0 (0 to 0.1) | 0.5 (0.2 to 0.9) | 0.6 (0.2 to 1.2) | 0.3 (0.1 to 0.5) | 409.7 (110 to 1340) | 266.2 (37.4 to 946.2) | 1322.5 (365.3 to 9291) |
|  |  |  | All ages | 0.3 (0.1 to 0.5) | 0.2 (0.1 to 0.5) | 0 (0 to 0.1) | 4.5 (1.7 to 8) | 3.3 (1.3 to 6.2) | 1.2 (0.4 to 2.2) | 1530.3 (684.3 to 3520.2) | 1284.6 (472.2 to 3090.3) | 3163 (1227.2 to 12981.2) |
|  | High systolic blood pressure | DALYs (Disability-Adjusted Life Years) | Age-standardized | 19.2 (6.3 to 33.3) | 21.8 (7.1 to 38.7) | 16.7 (5.6 to 29.7) | 26 (9.1 to 43.4) | 27 (9.1 to 46.2) | 24.6 (8.5 to 41) | 35.2 (6.6 to 77.7) | 23.8 (-12.7 to 75) | 46.7 (13.5 to 105) |
|  |  |  | All ages | 64.1 (22.5 to 111.1) | 33.4 (11.2 to 59.6) | 30.7 (10.3 to 55.7) | 270.3 (94.8 to 450.2) | 153.4 (52.1 to 260.3) | 116.9 (40.2 to 198.1) | 321.6 (228.3 to 448.1) | 359.5 (221.2 to 545.6) | 280.4 (191.8 to 441.2) |
|  |  | Deaths | Age-standardized | 0.8 (0.3 to 1.5) | 1.1 (0.3 to 2.1) | 0.6 (0.1 to 1.1) | 1.2 (0.4 to 2) | 1.4 (0.5 to 2.4) | 0.9 (0.3 to 1.5) | 43.5 (-2.3 to 121.9) | 30 (-21.8 to 109.2) | 56.1 (1.9 to 202.7) |
|  |  |  | All ages | 2 (0.7 to 3.6) | 1.3 (0.4 to 2.5) | 0.8 (0.2 to 1.4) | 11.1 (4 to 18.9) | 7.3 (2.5 to 12.9) | 3.8 (1.3 to 6.3) | 445.1 (279.1 to 734.4) | 466.8 (247.5 to 800.8) | 407.7 (230 to 901.3) |
|  | Lead exposure | DALYs (Disability-Adjusted Life Years) | Age-standardized | 4.3 (-0.6 to 10.7) | 4.5 (-0.7 to 12.2) | 4 (-0.6 to 9.8) | 5 (-0.7 to 12.1) | 5 (-0.7 to 11.9) | 4.8 (-0.7 to 12.1) | 16.3 (-7.2 to 50) | 11.3 (-23.4 to 52.7) | 19.8 (-3.7 to 66.8) |
|  |  |  | All ages | 14.3 (-2 to 35) | 6.9 (-1 to 18.4) | 7.5 (-1 to 18) | 50.7 (-7 to 124.4) | 27.9 (-3.9 to 66.2) | 22.9 (-3.2 to 56.3) | 253.9 (183.5 to 350.6) | 305.4 (185.7 to 446.2) | 206.3 (144.5 to 317.2) |
|  |  | Deaths | Age-standardized | 0.2 (0 to 0.5) | 0.2 (0 to 0.6) | 0.1 (0 to 0.4) | 0.2 (0 to 0.6) | 0.3 (0 to 0.7) | 0.2 (0 to 0.5) | 32 (-7.3 to 102) | 26.2 (-22 to 89.3) | 30.9 (-11.6 to 145.1) |
|  |  |  | All ages | 0.5 (-0.1 to 1.2) | 0.3 (0 to 0.7) | 0.2 (0 to 0.5) | 2.3 (-0.3 to 5.7) | 1.5 (-0.2 to 3.6) | 0.8 (-0.1 to 2) | 392.3 (249.4 to 644.8) | 441.1 (233.7 to 716.4) | 321.4 (182.4 to 695.8) |
|  | Smoking | DALYs (Disability-Adjusted Life Years) | Age-standardized | 1.6 (0.9 to 2.6) | 0.6 (0.3 to 1) | 2.6 (1.4 to 4.2) | 2.1 (1.2 to 3.3) | 0.7 (0.4 to 1.2) | 3.7 (2 to 6) | 28.5 (-0.3 to 74.4) | 23.3 (-28.8 to 117.8) | 43.3 (9.5 to 100.6) |
|  |  |  | All ages | 7.8 (4.1 to 12.5) | 1.3 (0.6 to 2.4) | 6.5 (3.3 to 10.4) | 25.3 (13.9 to 41.1) | 4.5 (2.3 to 7.7) | 20.8 (11.3 to 33.9) | 225.5 (152.8 to 339.4) | 239.4 (92.1 to 483.6) | 222.6 (148.8 to 352.1) |
|  |  | Deaths | Age-standardized | 0 (0 to 0.1) | 0 (0 to 0) | 0.1 (0 to 0.1) | 0.1 (0 to 0.1) | 0 (0 to 0.1) | 0.1 (0 to 0.2) | 37.8 (-15.5 to 164.9) | 29.3 (-44.6 to 198.7) | 55.1 (-5.4 to 230.5) |
|  |  |  | All ages | 0.1 (0.1 to 0.2) | 0 (0 to 0.1) | 0.1 (0 to 0.2) | 0.6 (0.3 to 0.9) | 0.1 (0.1 to 0.3) | 0.4 (0.2 to 0.7) | 325.3 (174.3 to 692.7) | 336.4 (109.3 to 847.8) | 322 (158 to 737.9) |
| Bushehr | All risk factors | DALYs (Disability-Adjusted Life Years) | Age-standardized | 18.6 (8.3 to 29.7) | 20.4 (8.8 to 33.9) | 16.8 (7.2 to 27.5) | 24.8 (12.7 to 37.8) | 25.9 (12.5 to 40.8) | 23.6 (12.4 to 36.6) | 33 (-0.4 to 94.8) | 27 (-15.8 to 94.9) | 40.8 (7.7 to 111.8) |
|  |  |  | All ages | 34.4 (14.9 to 54.9) | 18.3 (8 to 29.5) | 16.2 (7 to 26.2) | 175.2 (93.4 to 264.7) | 90.6 (44.7 to 143.1) | 84.6 (44.7 to 129.5) | 408.8 (292.8 to 606.4) | 395.9 (241.7 to 615.5) | 423.3 (303.9 to 633.9) |
|  |  | Deaths | Age-standardized | 0.8 (0.3 to 1.4) | 1 (0.4 to 1.7) | 0.6 (0.2 to 1) | 1.1 (0.5 to 1.6) | 1.3 (0.6 to 2.1) | 0.8 (0.4 to 1.3) | 36 (-10.1 to 133.5) | 36.3 (-22.1 to 137.6) | 37.2 (-9.3 to 189.3) |
|  |  |  | All ages | 1 (0.4 to 1.7) | 0.7 (0.3 to 1.2) | 0.4 (0.1 to 0.7) | 5.8 (3 to 8.8) | 3.6 (1.7 to 5.7) | 2.2 (1.1 to 3.5) | 469.9 (287.3 to 838.7) | 457.3 (227.8 to 824.6) | 492.1 (293.2 to 1082.5) |
|  | Diet high in sodium | DALYs (Disability-Adjusted Life Years) | Age-standardized | 0.4 (0 to 2.2) | 0.2 (0 to 1.9) | 0.5 (0 to 2.5) | 0.4 (0 to 2.4) | 0.3 (0 to 2.1) | 0.5 (0 to 2.8) | 8.9 (-95.4 to 2467.2) | 8.7 (-491.3 to 925317.9) | 9.4 (-96.8 to 2445.7) |
|  |  |  | All ages | 0.7 (0 to 4.2) | 0.2 (0 to 1.7) | 0.5 (0 to 2.5) | 2.8 (0 to 17) | 1 (0 to 7.5) | 1.9 (0 to 9.9) | 299.4 (-81.1 to 6879.8) | 311.8 (-1002 to 1497192.9) | 293.5 (-83.8 to 6879.8) |
|  |  | Deaths | Age-standardized | 0 (0 to 0.1) | 0 (0 to 0.1) | 0 (0 to 0.1) | 0 (0 to 0.1) | 0 (0 to 0.1) | 0 (0 to 0.1) | 12.8 (-97.9 to 5341.3) | 20.1 (-412.5 to 539916.4) | 6.2 (-98.1 to 5894.6) |
|  |  |  | All ages | 0 (0 to 0.1) | 0 (0 to 0.1) | 0 (0 to 0.1) | 0.1 (0 to 0.6) | 0 (0 to 0.3) | 0 (0 to 0.3) | 357.9 (-87.2 to 11989.9) | 381.8 (-1668.8 to 1569302.1) | 340.8 (-87.2 to 11990.1) |
|  | High alcohol use | DALYs (Disability-Adjusted Life Years) | Age-standardized | 0 (0 to 0) | 0 (0 to 0) | 0 (0 to 0) | 0.2 (0.1 to 0.3) | 0 (0 to 0.1) | 0.3 (0.2 to 0.5) | NA | NA | NA |
|  |  |  | All ages | 0 (0 to 0) | 0 (0 to 0) | 0 (0 to 0) | 1.6 (0.9 to 2.5) | 0.1 (0 to 0.2) | 1.4 (0.8 to 2.3) | NA | NA | NA |
|  |  | Deaths | Age-standardized | 0 (0 to 0) | 0 (0 to 0) | 0 (0 to 0) | 0 (0 to 0) | 0 (0 to 0) | 0 (0 to 0) | NA | NA | NA |
|  |  |  | All ages | 0 (0 to 0) | 0 (0 to 0) | 0 (0 to 0) | 0 (0 to 0.1) | 0 (0 to 0) | 0 (0 to 0) | NA | NA | NA |
|  | High body-mass index | DALYs (Disability-Adjusted Life Years) | Age-standardized | 3 (1.1 to 5.4) | 4.4 (1.6 to 8) | 1.7 (0.5 to 3.3) | 10.6 (4.3 to 18) | 12.7 (5.1 to 22.5) | 8.2 (3 to 14.1) | 248.9 (92 to 499.9) | 192.3 (45.3 to 408.7) | 396.3 (162.9 to 1041.8) |
|  |  |  | All ages | 6.4 (2.4 to 11) | 4.5 (1.7 to 7.9) | 2 (0.7 to 3.6) | 74.6 (30.3 to 128) | 45.4 (18.4 to 79.2) | 29.2 (11.1 to 50.1) | 1065.8 (590.9 to 1650.2) | 919.8 (440.9 to 1530.6) | 1398.8 (718.7 to 2966.2) |
|  |  | Deaths | Age-standardized | 0.1 (0 to 0.2) | 0.2 (0.1 to 0.4) | 0 (0 to 0.1) | 0.5 (0.2 to 0.8) | 0.6 (0.2 to 1.1) | 0.3 (0.1 to 0.5) | 308.9 (85.5 to 898.5) | 256.5 (42.3 to 808.7) | 531.9 (147.4 to 2515.1) |
|  |  |  | All ages | 0.2 (0.1 to 0.3) | 0.1 (0 to 0.3) | 0 (0 to 0.1) | 2.5 (1 to 4.4) | 1.8 (0.7 to 3.1) | 0.8 (0.3 to 1.4) | 1416.1 (639.4 to 2839.5) | 1229.4 (484.6 to 2574) | 2116.7 (910.2 to 6052.2) |
|  | High systolic blood pressure | DALYs (Disability-Adjusted Life Years) | Age-standardized | 15.9 (5.2 to 27.2) | 17.7 (5.8 to 30.7) | 13.9 (4.3 to 25.3) | 17.6 (5.7 to 31) | 18.3 (6 to 33.3) | 16.9 (5.3 to 29) | 11 (-17.3 to 48.8) | 3.5 (-32.5 to 55.3) | 21.5 (-9 to 72.8) |
|  |  |  | All ages | 28.1 (9.4 to 48.4) | 15.5 (5.1 to 26.8) | 12.6 (3.9 to 22.5) | 122.1 (39.2 to 211.6) | 63.5 (20.5 to 113.9) | 58.6 (18.1 to 101.7) | 334.9 (238.9 to 464.3) | 310.4 (170.4 to 494.2) | 364.9 (254.2 to 558.4) |
|  |  | Deaths | Age-standardized | 0.7 (0.2 to 1.2) | 0.9 (0.3 to 1.6) | 0.5 (0.1 to 1) | 0.8 (0.3 to 1.4) | 1 (0.3 to 1.8) | 0.6 (0.2 to 1.1) | 11.3 (-26.6 to 77.5) | 10.3 (-38.5 to 93.2) | 14.7 (-26.8 to 120.7) |
|  |  |  | All ages | 0.9 (0.3 to 1.6) | 0.6 (0.2 to 1.1) | 0.3 (0.1 to 0.6) | 4.2 (1.5 to 7.5) | 2.6 (0.8 to 4.8) | 1.6 (0.5 to 2.9) | 374.2 (223.1 to 630.7) | 354.2 (154.4 to 659.4) | 410.7 (230.6 to 876.2) |
|  | Lead exposure | DALYs (Disability-Adjusted Life Years) | Age-standardized | 3.7 (-0.5 to 9.4) | 3.8 (-0.5 to 9.6) | 3.7 (-0.5 to 9.5) | 4.3 (-0.6 to 10.9) | 4.3 (-0.6 to 10.7) | 4.2 (-0.6 to 10.4) | 14 (-7.5 to 41.1) | 13.9 (-19.2 to 50.6) | 14.2 (-7.2 to 56.4) |
|  |  |  | All ages | 6.6 (-0.9 to 16.9) | 3.2 (-0.4 to 8.2) | 3.3 (-0.4 to 8.7) | 27.7 (-3.7 to 70.6) | 14 (-1.9 to 34.9) | 13.8 (-1.8 to 34.3) | 322 (244.7 to 423) | 331.2 (214.2 to 462) | 313.2 (234.7 to 443.5) |
|  |  | Deaths | Age-standardized | 0.2 (0 to 0.4) | 0.2 (0 to 0.5) | 0.1 (0 to 0.4) | 0.2 (0 to 0.5) | 0.2 (0 to 0.6) | 0.2 (0 to 0.4) | 26.4 (-8.8 to 80.2) | 32.2 (-19 to 89.8) | 18.5 (-17.7 to 116.4) |
|  |  |  | All ages | 0.2 (0 to 0.5) | 0.1 (0 to 0.3) | 0.1 (0 to 0.2) | 1.1 (-0.2 to 2.7) | 0.7 (-0.1 to 1.6) | 0.4 (-0.1 to 1) | 422.3 (272.5 to 644.2) | 433.6 (235 to 669.3) | 405.8 (247.5 to 816.1) |
|  | Smoking | DALYs (Disability-Adjusted Life Years) | Age-standardized | 1.6 (0.9 to 2.5) | 0.6 (0.3 to 0.9) | 2.7 (1.5 to 4.3) | 1.9 (1.1 to 3.1) | 0.7 (0.3 to 1.2) | 3.2 (1.8 to 5.2) | 19.8 (-7.7 to 58.3) | 17.8 (-34.6 to 99.1) | 21.3 (-7.1 to 67) |
|  |  |  | All ages | 4.1 (2.2 to 6.4) | 0.7 (0.3 to 1.2) | 3.4 (1.8 to 5.4) | 17.1 (9.4 to 27.8) | 2.9 (1.3 to 5.1) | 14.3 (7.9 to 22.9) | 317.4 (223.1 to 444.6) | 307.6 (124.4 to 582.5) | 319.4 (221 to 452.7) |
|  |  | Deaths | Age-standardized | 0 (0 to 0.1) | 0 (0 to 0) | 0.1 (0 to 0.1) | 0 (0 to 0.1) | 0 (0 to 0) | 0.1 (0 to 0.1) | 22.7 (-22.5 to 113.5) | 25.4 (-47.8 to 192.2) | 20.3 (-29.2 to 130.2) |
|  |  |  | All ages | 0.1 (0 to 0.1) | 0 (0 to 0) | 0.1 (0 to 0.1) | 0.3 (0.2 to 0.5) | 0.1 (0 to 0.1) | 0.2 (0.1 to 0.4) | 362.4 (197.6 to 682) | 357.3 (110.2 to 843.9) | 363.8 (187.1 to 738.1) |
| Chahar Mahaal and Bakhtiari | All risk factors | DALYs (Disability-Adjusted Life Years) | Age-standardized | 19.1 (8.1 to 31.4) | 20.5 (8.3 to 33.8) | 17.5 (7.3 to 29.6) | 25.3 (12.9 to 38.4) | 26.5 (12.9 to 41.1) | 23.5 (12.1 to 36.4) | 32.4 (1.6 to 82.4) | 29.5 (-10.6 to 85.3) | 34.3 (1.9 to 98.1) |
|  |  |  | All ages | 39.4 (17.4 to 63.2) | 19.8 (8.6 to 32.7) | 19.5 (8.5 to 32.3) | 190.7 (97.8 to 289.6) | 107.7 (52.8 to 166.3) | 83.1 (43.5 to 129.6) | 384.7 (283 to 557.8) | 442.7 (287.2 to 668) | 325.7 (230 to 497.2) |
|  |  | Deaths | Age-standardized | 0.8 (0.3 to 1.3) | 0.9 (0.3 to 1.6) | 0.6 (0.2 to 1.1) | 1 (0.5 to 1.6) | 1.2 (0.5 to 2) | 0.8 (0.4 to 1.2) | 32.4 (-14.8 to 108.4) | 32.1 (-22.9 to 126) | 28.7 (-19.2 to 165) |
|  |  |  | All ages | 1.1 (0.4 to 1.9) | 0.7 (0.3 to 1.2) | 0.4 (0.1 to 0.7) | 6.9 (3.4 to 10.8) | 4.6 (2.1 to 7.4) | 2.3 (1.2 to 3.6) | 512.7 (302.1 to 849.8) | 552.6 (284.1 to 1000.9) | 445.1 (251.5 to 949.6) |
|  | Diet high in sodium | DALYs (Disability-Adjusted Life Years) | Age-standardized | 0.3 (0 to 2) | 0.2 (0 to 1.6) | 0.5 (0 to 2.4) | 0.4 (0 to 2.1) | 0.2 (0 to 1.7) | 0.5 (0 to 2.7) | 0.8 (-95.8 to 1455.5) | -3.5 (-610766.9 to 108235.1) | 7.7 (-95.6 to 1511.9) |
|  |  |  | All ages | 0.8 (0 to 4.7) | 0.2 (0 to 1.6) | 0.6 (0 to 3) | 2.7 (0 to 16.5) | 0.9 (0 to 6.9) | 1.8 (0 to 9.9) | 233.1 (-85.2 to 4132.7) | 277.8 (-1210221.9 to 236626.6) | 214.8 (-85.2 to 3944.2) |
|  |  | Deaths | Age-standardized | 0 (0 to 0.1) | 0 (0 to 0.1) | 0 (0 to 0.1) | 0 (0 to 0.1) | 0 (0 to 0.1) | 0 (0 to 0.1) | 0.8 (-97.6 to 2501) | -2.2 (-25126.5 to 97946.9) | 3.6 (-97.5 to 2826.8) |
|  |  |  | All ages | 0 (0 to 0.1) | 0 (0 to 0.1) | 0 (0 to 0.1) | 0.1 (0 to 0.6) | 0 (0 to 0.3) | 0 (0 to 0.3) | 312.4 (-90.7 to 6306.8) | 346.9 (-446276.5 to 218727.8) | 290.1 (-90.7 to 6352.6) |
|  | High alcohol use | DALYs (Disability-Adjusted Life Years) | Age-standardized | 0 (0 to 0) | 0 (0 to 0) | 0 (0 to 0) | 0.2 (0.1 to 0.3) | 0 (0 to 0.1) | 0.3 (0.2 to 0.6) | NA | NA | NA |
|  |  |  | All ages | 0 (0 to 0) | 0 (0 to 0) | 0 (0 to 0) | 1.4 (0.7 to 2.3) | 0.1 (0 to 0.2) | 1.3 (0.7 to 2.2) | NA | NA | NA |
|  |  | Deaths | Age-standardized | 0 (0 to 0) | 0 (0 to 0) | 0 (0 to 0) | 0 (0 to 0) | 0 (0 to 0) | 0 (0 to 0) | NA | NA | NA |
|  |  |  | All ages | 0 (0 to 0) | 0 (0 to 0) | 0 (0 to 0) | 0 (0 to 0.1) | 0 (0 to 0) | 0 (0 to 0) | NA | NA | NA |
|  | High body-mass index | DALYs (Disability-Adjusted Life Years) | Age-standardized | 2.5 (0.9 to 4.8) | 3.7 (1.3 to 7.2) | 1.3 (0.4 to 2.6) | 9 (3.4 to 15.8) | 10.3 (4.1 to 18.6) | 7.4 (2.9 to 12.9) | 262.7 (100.7 to 575.4) | 178.5 (43.2 to 405) | 491.8 (204.1 to 1526) |
|  |  |  | All ages | 5.9 (2.3 to 10.9) | 4.1 (1.5 to 7.5) | 1.8 (0.6 to 3.4) | 68.5 (26.3 to 120.5) | 42.4 (16.9 to 76.1) | 26.1 (10.3 to 45.2) | 1054.7 (593.9 to 1748.3) | 924.6 (467.3 to 1541.9) | 1353.9 (737 to 3282.5) |
|  |  | Deaths | Age-standardized | 0.1 (0 to 0.2) | 0.1 (0 to 0.3) | 0 (0 to 0.1) | 0.4 (0.1 to 0.7) | 0.4 (0.2 to 0.8) | 0.3 (0.1 to 0.5) | 307.3 (78.1 to 1074.9) | 224.4 (25.4 to 867.8) | 692 (172.7 to 4058.1) |
|  |  |  | All ages | 0.1 (0 to 0.3) | 0.1 (0 to 0.3) | 0 (0 to 0.1) | 2.5 (1 to 4.4) | 1.7 (0.6 to 3.2) | 0.7 (0.3 to 1.3) | 1545.6 (702.8 to 3333.7) | 1332 (516.3 to 3147.2) | 2403.6 (1031.3 to 7353.3) |
|  | High systolic blood pressure | DALYs (Disability-Adjusted Life Years) | Age-standardized | 16.7 (5.5 to 29.5) | 18.2 (6 to 31.8) | 14.9 (4.6 to 26.9) | 19.5 (6.5 to 33.7) | 21.1 (6.8 to 36.8) | 17.4 (5.7 to 30.3) | 16.8 (-13 to 50.5) | 15.5 (-20.8 to 62.9) | 16.3 (-12.9 to 61.1) |
|  |  |  | All ages | 33.1 (10.6 to 57.6) | 17.3 (5.8 to 30.6) | 15.8 (4.8 to 28.7) | 145.4 (47.8 to 250.6) | 85.1 (27 to 148.1) | 60.3 (20.1 to 106.4) | 339.3 (237.7 to 455.2) | 393.1 (243.8 to 593.2) | 280.7 (187.4 to 413.4) |
|  |  | Deaths | Age-standardized | 0.7 (0.2 to 1.3) | 0.8 (0.3 to 1.5) | 0.5 (0.1 to 1) | 0.8 (0.3 to 1.4) | 1 (0.3 to 1.7) | 0.6 (0.2 to 1) | 15.2 (-26.4 to 78.7) | 16.7 (-30.7 to 92.2) | 8.2 (-34.1 to 103.3) |
|  |  |  | All ages | 1 (0.3 to 1.8) | 0.6 (0.2 to 1.2) | 0.4 (0.1 to 0.7) | 5.4 (1.9 to 9.5) | 3.7 (1.2 to 6.5) | 1.7 (0.6 to 3) | 444.6 (249.8 to 727.3) | 484.6 (247.2 to 842.8) | 373.7 (186.9 to 789.4) |
|  | Lead exposure | DALYs (Disability-Adjusted Life Years) | Age-standardized | 3.9 (-0.5 to 9.7) | 3.8 (-0.5 to 9.9) | 4 (-0.5 to 9.9) | 4 (-0.5 to 10) | 3.7 (-0.5 to 9.2) | 4.4 (-0.6 to 10.7) | 4 (-17.3 to 31.2) | -1.5 (-29.3 to 31.6) | 10 (-10.2 to 45.9) |
|  |  |  | All ages | 7.7 (-1 to 18.8) | 3.6 (-0.5 to 9.3) | 4.1 (-0.5 to 10.2) | 29.3 (-4 to 73) | 14.8 (-2 to 36.7) | 14.5 (-2 to 36) | 279.2 (206.6 to 368.6) | 310.4 (199.2 to 443.1) | 252 (182.5 to 356.3) |
|  |  | Deaths | Age-standardized | 0.2 (0 to 0.4) | 0.2 (0 to 0.5) | 0.1 (0 to 0.4) | 0.2 (0 to 0.4) | 0.2 (0 to 0.5) | 0.2 (0 to 0.4) | 10.7 (-22.2 to 62.9) | 8.8 (-32.6 to 67.1) | 10.8 (-21.6 to 106.7) |
|  |  |  | All ages | 0.2 (0 to 0.6) | 0.1 (0 to 0.4) | 0.1 (0 to 0.3) | 1.2 (-0.2 to 2.9) | 0.7 (-0.1 to 1.8) | 0.5 (-0.1 to 1.2) | 415.7 (260.9 to 653.1) | 437.6 (229.7 to 719.3) | 385.4 (236.7 to 765.4) |
|  | Smoking | DALYs (Disability-Adjusted Life Years) | Age-standardized | 1.6 (0.9 to 2.5) | 0.5 (0.3 to 0.9) | 2.6 (1.4 to 4.2) | 1.8 (1 to 2.8) | 0.6 (0.3 to 1) | 3.2 (1.7 to 5) | 12.9 (-14.7 to 45.7) | 7.5 (-41 to 88.9) | 21.3 (-9.9 to 63.8) |
|  |  |  | All ages | 4.5 (2.5 to 7) | 0.7 (0.3 to 1.2) | 3.8 (2 to 6.1) | 15.5 (8.5 to 24.7) | 2.5 (1.2 to 4.3) | 13 (6.9 to 20.9) | 246.8 (156.7 to 355.4) | 265.3 (107.7 to 527.4) | 243.5 (154.1 to 360) |
|  |  | Deaths | Age-standardized | 0 (0 to 0.1) | 0 (0 to 0) | 0.1 (0 to 0.1) | 0 (0 to 0.1) | 0 (0 to 0) | 0.1 (0 to 0.1) | 11.1 (-31.6 to 95.4) | 3.6 (-56.4 to 146.7) | 16.9 (-32.7 to 122.3) |
|  |  |  | All ages | 0.1 (0 to 0.1) | 0 (0 to 0) | 0.1 (0 to 0.1) | 0.3 (0.1 to 0.5) | 0.1 (0 to 0.1) | 0.2 (0.1 to 0.4) | 312.7 (152.5 to 601.5) | 322.5 (90.3 to 799) | 310.2 (144.8 to 658.3) |
| East Azarbayejan | All risk factors | DALYs (Disability-Adjusted Life Years) | Age-standardized | 25.6 (10.8 to 41.5) | 30.3 (11.8 to 52.1) | 21.1 (8.6 to 35.2) | 34.5 (17.1 to 52.6) | 40.3 (19 to 63.7) | 28.6 (14 to 44.5) | 34.6 (3.3 to 88.2) | 33.2 (-9.4 to 104.4) | 35.9 (7.2 to 99.9) |
|  |  |  | All ages | 263.3 (117.1 to 414.8) | 139.3 (57.1 to 231.4) | 124 (54.3 to 206.5) | 1075.1 (543.5 to 1626) | 608.5 (293.8 to 938.6) | 466.6 (230.3 to 716.2) | 308.3 (223.7 to 446) | 336.8 (199.6 to 553.6) | 276.4 (207.6 to 414) |
|  |  | Deaths | Age-standardized | 1.3 (0.5 to 2.2) | 1.7 (0.7 to 3.2) | 0.8 (0.3 to 1.4) | 1.8 (0.8 to 2.7) | 2.4 (1.1 to 4) | 1.1 (0.5 to 1.7) | 36.8 (-9.5 to 119.6) | 38.8 (-18.8 to 142.6) | 32.4 (-10.4 to 160.6) |
|  |  |  | All ages | 8.9 (3.7 to 15.1) | 5.8 (2.3 to 10.6) | 3.1 (1.1 to 5.2) | 42.8 (19.7 to 66) | 29.2 (12.8 to 46.9) | 13.6 (6.3 to 21.2) | 381.5 (227.8 to 641.9) | 400.8 (198.8 to 740.4) | 344.6 (205.8 to 727.5) |
|  | Diet high in sodium | DALYs (Disability-Adjusted Life Years) | Age-standardized | 0.4 (0 to 2.6) | 0.3 (0 to 2.3) | 0.5 (0 to 3) | 0.4 (0 to 2.8) | 0.3 (0 to 2.5) | 0.6 (0 to 3.2) | 3.5 (-94.2 to 1324) | 5.1 (-1410.6 to 28295.2) | 4.9 (-93.4 to 1348.7) |
|  |  |  | All ages | 5 (0 to 28.3) | 1.6 (0 to 11.4) | 3.4 (0 to 18) | 14.7 (0 to 90.1) | 5.2 (0 to 39.3) | 9.5 (0 to 51.4) | 195.1 (-82.8 to 3474.5) | 233.8 (-6935 to 39222.9) | 177.4 (-82.7 to 3555.7) |
|  |  | Deaths | Age-standardized | 0 (0 to 0.1) | 0 (0 to 0.1) | 0 (0 to 0.1) | 0 (0 to 0.1) | 0 (0 to 0.1) | 0 (0 to 0.1) | 4.9 (-97.1 to 1914.9) | 10 (-188.8 to 319795.9) | 0.5 (-96.7 to 2632) |
|  |  |  | All ages | 0.1 (0 to 0.8) | 0.1 (0 to 0.5) | 0.1 (0 to 0.4) | 0.5 (0 to 3.2) | 0.2 (0 to 1.7) | 0.2 (0 to 1.5) | 246.1 (-88.8 to 4712.4) | 282.9 (-26845.4 to 18492.8) | 218.5 (-89 to 5755.9) |
|  | High alcohol use | DALYs (Disability-Adjusted Life Years) | Age-standardized | 0 (0 to 0) | 0 (0 to 0) | 0 (0 to 0) | 0.2 (0.1 to 0.3) | 0 (0 to 0.1) | 0.4 (0.2 to 0.6) | NA | NA | NA |
|  |  |  | All ages | 0 (0 to 0) | 0 (0 to 0) | 0 (0 to 0) | 7.2 (4.1 to 11.4) | 0.6 (0.2 to 1.2) | 6.6 (3.8 to 10.2) | NA | NA | NA |
|  |  | Deaths | Age-standardized | 0 (0 to 0) | 0 (0 to 0) | 0 (0 to 0) | 0 (0 to 0) | 0 (0 to 0) | 0 (0 to 0) | NA | NA | NA |
|  |  |  | All ages | 0 (0 to 0) | 0 (0 to 0) | 0 (0 to 0) | 0.2 (0.1 to 0.3) | 0 (0 to 0.1) | 0.1 (0.1 to 0.2) | NA | NA | NA |
|  | High body-mass index | DALYs (Disability-Adjusted Life Years) | Age-standardized | 3.1 (1.1 to 5.7) | 5.1 (1.7 to 9.9) | 1.2 (0.3 to 2.6) | 11.3 (4.7 to 20) | 15 (6.1 to 27.6) | 7.5 (3 to 13.7) | 266.7 (79 to 615.3) | 195.9 (28.2 to 521) | 523.1 (217.9 to 1705.3) |
|  |  |  | All ages | 37.4 (13.9 to 65.5) | 27.9 (10.5 to 48.9) | 9.4 (2.9 to 18) | 351.8 (148.8 to 617.5) | 230.2 (93.6 to 418) | 121.7 (49.2 to 215.2) | 841.7 (441.5 to 1486.6) | 724.2 (302.8 to 1385) | 1189.2 (649.5 to 2963.9) |
|  |  | Deaths | Age-standardized | 0.1 (0 to 0.3) | 0.2 (0.1 to 0.6) | 0 (0 to 0.1) | 0.6 (0.2 to 1) | 0.9 (0.3 to 1.6) | 0.3 (0.1 to 0.6) | 315.3 (62.7 to 1071.7) | 251.7 (18.3 to 1005.5) | 760.3 (174.4 to 4770) |
|  |  |  | All ages | 1.1 (0.4 to 2.1) | 0.9 (0.3 to 1.9) | 0.2 (0 to 0.4) | 14 (5.7 to 24.8) | 10.4 (4.1 to 19.1) | 3.6 (1.2 to 6.7) | 1162.8 (462.6 to 2692) | 1011.2 (327.5 to 2617.3) | 2007.4 (815.8 to 7039.1) |
|  | High systolic blood pressure | DALYs (Disability-Adjusted Life Years) | Age-standardized | 22.7 (7.5 to 39) | 27.3 (9 to 49.1) | 18.3 (5.9 to 31.7) | 27.4 (9.4 to 46.8) | 32.3 (10.7 to 57.6) | 22.5 (7.3 to 38.3) | 20.7 (-7.4 to 56.9) | 18.6 (-20.7 to 74.9) | 23.4 (-3 to 71.2) |
|  |  |  | All ages | 224.1 (74.3 to 379.9) | 122.1 (40.5 to 218.2) | 102 (33 to 182.3) | 847.2 (285.9 to 1418.6) | 486.1 (160.4 to 846) | 361.1 (117.7 to 612.3) | 278 (200.7 to 378.5) | 298 (177.6 to 474) | 254 (183.8 to 370.8) |
|  |  | Deaths | Age-standardized | 1.2 (0.4 to 2.1) | 1.6 (0.5 to 3.1) | 0.7 (0.2 to 1.3) | 1.4 (0.4 to 2.4) | 2 (0.6 to 3.5) | 0.9 (0.3 to 1.5) | 20.7 (-19.1 to 79.9) | 22.2 (-27.3 to 107.1) | 17.5 (-23.5 to 118.3) |
|  |  |  | All ages | 8 (2.6 to 14.1) | 5.3 (1.8 to 9.9) | 2.7 (0.8 to 4.8) | 34.7 (11.3 to 58.5) | 23.7 (7.3 to 41.7) | 10.9 (3.5 to 18.8) | 336 (199.2 to 542.2) | 349.8 (165.8 to 643) | 308.9 (174.4 to 650) |
|  | Lead exposure | DALYs (Disability-Adjusted Life Years) | Age-standardized | 4.5 (-0.6 to 11.3) | 4.9 (-0.7 to 12.7) | 4.1 (-0.6 to 9.9) | 5.1 (-0.7 to 12.6) | 5.6 (-0.8 to 14.4) | 4.5 (-0.6 to 11.2) | 12.9 (-12 to 44.9) | 13.9 (-20.4 to 61.7) | 11.8 (-10 to 48.3) |
|  |  |  | All ages | 44.5 (-6 to 110.7) | 22 (-3.1 to 57.8) | 22.5 (-3.1 to 56.1) | 148.4 (-20.7 to 375.7) | 78.9 (-11.6 to 198.5) | 69.5 (-9.3 to 172.4) | 233.8 (170 to 328) | 259 (158.6 to 407.9) | 209.1 (151.8 to 293.9) |
|  |  | Deaths | Age-standardized | 0.2 (0 to 0.6) | 0.3 (0 to 0.7) | 0.2 (0 to 0.4) | 0.3 (0 to 0.7) | 0.4 (-0.1 to 1) | 0.2 (0 to 0.5) | 20.9 (-15.6 to 76.8) | 25.7 (-20.8 to 93.7) | 12.8 (-21.5 to 102) |
|  |  |  | All ages | 1.6 (-0.2 to 3.9) | 1 (-0.1 to 2.4) | 0.6 (-0.1 to 1.6) | 6.5 (-1 to 16.2) | 4.2 (-0.7 to 10.8) | 2.3 (-0.4 to 5.6) | 312 (190.7 to 498.6) | 335.5 (172.1 to 576.5) | 274.7 (157.4 to 546.2) |
|  | Smoking | DALYs (Disability-Adjusted Life Years) | Age-standardized | 1.9 (1.1 to 3.2) | 0.7 (0.4 to 1.2) | 3.1 (1.7 to 5.1) | 2.1 (1.2 to 3.4) | 0.8 (0.4 to 1.5) | 3.5 (1.9 to 5.7) | 9.7 (-14.8 to 43.9) | 8.1 (-40.9 to 104.6) | 14 (-12.1 to 51.1) |
|  |  |  | All ages | 29.4 (15.9 to 47.3) | 5.1 (2.6 to 8.6) | 24.3 (13.3 to 39) | 81.9 (44.8 to 130.5) | 14.3 (6.9 to 26) | 67.5 (36.1 to 108.9) | 178.5 (117.7 to 256.3) | 183.5 (57.5 to 408.9) | 177.5 (113.3 to 263.7) |
|  |  | Deaths | Age-standardized | 0.1 (0 to 0.1) | 0 (0 to 0.1) | 0.1 (0 to 0.1) | 0.1 (0 to 0.1) | 0 (0 to 0.1) | 0.1 (0 to 0.2) | 9.9 (-32.7 to 91.2) | 14.2 (-56.1 to 187.1) | 9.4 (-34.4 to 104.3) |
|  |  |  | All ages | 0.5 (0.3 to 0.9) | 0.1 (0.1 to 0.2) | 0.4 (0.2 to 0.7) | 1.7 (0.9 to 2.8) | 0.4 (0.2 to 0.9) | 1.3 (0.6 to 2.2) | 223.1 (107.5 to 437.5) | 240.5 (53.6 to 647.2) | 217.8 (95.4 to 485) |
| Fars | All risk factors | DALYs (Disability-Adjusted Life Years) | Age-standardized | 25.6 (11.1 to 40.7) | 32.1 (13.1 to 51.3) | 18 (8 to 30.7) | 33.3 (17.2 to 50.1) | 43.5 (21.5 to 66.1) | 23 (12 to 35.1) | 30.2 (1 to 83.7) | 35.2 (-0.5 to 101.9) | 27.5 (-7.5 to 96.3) |
|  |  |  | All ages | 265.7 (120.4 to 426) | 165.4 (69.1 to 273.4) | 100.3 (48 to 168.6) | 1294.8 (670.9 to 1931.6) | 839.9 (406.8 to 1270.9) | 454.9 (240 to 689.4) | 387.3 (288.5 to 563) | 407.9 (277.8 to 633.2) | 353.3 (239.5 to 562.6) |
|  |  | Deaths | Age-standardized | 1.1 (0.4 to 1.8) | 1.3 (0.5 to 2.3) | 0.7 (0.3 to 1.3) | 1.4 (0.7 to 2.2) | 2 (0.9 to 3.2) | 0.9 (0.4 to 1.4) | 37 (-7.4 to 113.2) | 52.4 (-7.3 to 160.6) | 27 (-18.2 to 154.7) |
|  |  |  | All ages | 7.3 (3.1 to 11.9) | 4.9 (2 to 8.5) | 2.4 (1 to 4.2) | 46.7 (23.3 to 72.4) | 32.1 (14.8 to 49.7) | 14.6 (7.1 to 23.6) | 536.8 (341 to 874.7) | 550.8 (302.4 to 993.8) | 508.1 (274.8 to 1032.3) |
|  | Diet high in sodium | DALYs (Disability-Adjusted Life Years) | Age-standardized | 0.4 (0 to 2.5) | 0.3 (0 to 2.5) | 0.5 (0 to 2.5) | 0.4 (0 to 2.6) | 0.4 (0 to 2.8) | 0.5 (0 to 2.7) | 3.7 (-97.1 to 956.3) | 5.1 (-643.8 to 31459.2) | 1.8 (-97.2 to 1058.8) |
|  |  |  | All ages | 4.9 (0 to 28.3) | 2 (0 to 13.9) | 2.9 (0 to 14) | 17.3 (0 to 103.9) | 7.4 (0 to 54.4) | 9.9 (0 to 54.3) | 254.6 (-90.3 to 2852.2) | 275.2 (-1425.5 to 3632212.5) | 240.8 (-90.6 to 2930.9) |
|  |  | Deaths | Age-standardized | 0 (0 to 0.1) | 0 (0 to 0.1) | 0 (0 to 0.1) | 0 (0 to 0.1) | 0 (0 to 0.1) | 0 (0 to 0.1) | 9.1 (-97.9 to 1794) | 18.7 (-461.9 to 39892.2) | -0.4 (-97.9 to 1875.2) |
|  |  |  | All ages | 0.1 (0 to 0.7) | 0 (0 to 0.4) | 0.1 (0 to 0.4) | 0.5 (0 to 3.7) | 0.2 (0 to 2) | 0.3 (0 to 1.8) | 352.2 (-93 to 4244.9) | 375.4 (-2797 to 62147.2) | 334.2 (-93.9 to 4321.9) |
|  | High alcohol use | DALYs (Disability-Adjusted Life Years) | Age-standardized | 0 (0 to 0) | 0 (0 to 0) | 0 (0 to 0) | 0.2 (0.1 to 0.3) | 0 (0 to 0.1) | 0.3 (0.2 to 0.5) | NA | NA | NA |
|  |  |  | All ages | 0 (0 to 0) | 0 (0 to 0) | 0 (0 to 0) | 8.6 (4.7 to 13) | 0.9 (0.3 to 1.8) | 7.6 (4.2 to 11.7) | NA | NA | NA |
|  |  | Deaths | Age-standardized | 0 (0 to 0) | 0 (0 to 0) | 0 (0 to 0) | 0 (0 to 0) | 0 (0 to 0) | 0 (0 to 0) | NA | NA | NA |
|  |  |  | All ages | 0 (0 to 0) | 0 (0 to 0) | 0 (0 to 0) | 0.2 (0.1 to 0.3) | 0 (0 to 0.1) | 0.2 (0.1 to 0.3) | NA | NA | NA |
|  | High body-mass index | DALYs (Disability-Adjusted Life Years) | Age-standardized | 4.4 (1.6 to 8) | 6.9 (2.4 to 12.7) | 1.7 (0.5 to 3.5) | 13.5 (5.6 to 23.4) | 19 (7.9 to 33.4) | 8 (3.1 to 14.1) | 206.6 (68.2 to 404.4) | 173.7 (39.1 to 373.1) | 377.1 (151.9 to 949.5) |
|  |  |  | All ages | 53 (19.8 to 90.5) | 41.2 (15.7 to 71.8) | 11.8 (3.9 to 21.5) | 531.8 (220.1 to 930.8) | 375.3 (159.2 to 653.7) | 156.5 (60.9 to 272.8) | 904 (495.8 to 1357.2) | 811 (409.9 to 1250.5) | 1229.6 (626.9 to 2548.8) |
|  |  | Deaths | Age-standardized | 0.2 (0.1 to 0.3) | 0.2 (0.1 to 0.5) | 0.1 (0 to 0.1) | 0.6 (0.2 to 1) | 0.9 (0.3 to 1.6) | 0.3 (0.1 to 0.6) | 265 (64.9 to 765.9) | 248.4 (46.6 to 779.5) | 524.6 (153.7 to 2504.2) |
|  |  |  | All ages | 1.2 (0.4 to 2.3) | 1 (0.3 to 2) | 0.2 (0.1 to 0.5) | 18.6 (7.8 to 32.9) | 13.4 (5.3 to 24.5) | 5.2 (2 to 9.1) | 1409.7 (684 to 2687.1) | 1234.5 (507.2 to 2698.6) | 2191.2 (953.1 to 5806.7) |
|  | High systolic blood pressure | DALYs (Disability-Adjusted Life Years) | Age-standardized | 22.1 (7.4 to 37.7) | 28.5 (9.2 to 48.1) | 14.7 (4.8 to 27.4) | 24.8 (8.1 to 42.1) | 33.6 (11.4 to 57.5) | 15.9 (5 to 28.1) | 12.4 (-12 to 48.6) | 17.9 (-16.8 to 66.8) | 8.2 (-22.3 to 48.6) |
|  |  |  | All ages | 220.9 (72.1 to 382.7) | 143.4 (45.6 to 248.6) | 77.5 (25.1 to 144.1) | 955.1 (307.7 to 1616.4) | 644.9 (216.7 to 1101.5) | 310.2 (95.4 to 548) | 332.4 (234.1 to 467.2) | 349.6 (224.7 to 520.7) | 300.5 (193.4 to 462.4) |
|  |  | Deaths | Age-standardized | 0.9 (0.3 to 1.6) | 1.2 (0.4 to 2.2) | 0.6 (0.2 to 1.1) | 1.1 (0.4 to 1.9) | 1.6 (0.5 to 2.7) | 0.6 (0.2 to 1.2) | 16.7 (-23.4 to 71.9) | 31.5 (-20.4 to 115.2) | 5 (-35.3 to 83.3) |
|  |  |  | All ages | 6.4 (2.2 to 11.1) | 4.4 (1.4 to 8.1) | 2 (0.6 to 3.7) | 35.5 (12 to 61.1) | 25.2 (8.3 to 43.9) | 10.3 (3.3 to 19.3) | 455.8 (272.1 to 702) | 471 (240.4 to 823) | 421.8 (211.1 to 788) |
|  | Lead exposure | DALYs (Disability-Adjusted Life Years) | Age-standardized | 4.3 (-0.6 to 11) | 4.9 (-0.7 to 12.8) | 3.5 (-0.6 to 9.2) | 4.6 (-0.6 to 11.5) | 5.4 (-0.7 to 13.7) | 3.9 (-0.5 to 9.9) | 8.9 (-13.5 to 34.6) | 10.1 (-18.4 to 41.3) | 11.1 (-13.4 to 46.9) |
|  |  |  | All ages | 43.4 (-5.9 to 113.4) | 24.8 (-3.5 to 64.4) | 18.6 (-2.7 to 47.9) | 171.9 (-22.2 to 424.5) | 98.9 (-12.9 to 253.3) | 73.1 (-9.5 to 184.9) | 296 (216.6 to 390.3) | 298 (201.9 to 414.8) | 293.5 (204.4 to 419.9) |
|  |  | Deaths | Age-standardized | 0.2 (0 to 0.5) | 0.2 (0 to 0.6) | 0.1 (0 to 0.4) | 0.2 (0 to 0.5) | 0.3 (0 to 0.7) | 0.2 (0 to 0.4) | 23.4 (-11.2 to 69) | 34.9 (-14.9 to 93.5) | 17.5 (-19.2 to 93.4) |
|  |  |  | All ages | 1.2 (-0.2 to 3.1) | 0.8 (-0.1 to 2) | 0.5 (-0.1 to 1.3) | 7 (-1 to 17.2) | 4.3 (-0.6 to 11.1) | 2.7 (-0.4 to 6.7) | 465.4 (300.7 to 678.7) | 461.5 (252 to 712.9) | 471.6 (284.9 to 839) |
|  | Smoking | DALYs (Disability-Adjusted Life Years) | Age-standardized | 1.7 (1 to 2.8) | 0.3 (0.1 to 0.5) | 3.3 (1.8 to 5.4) | 2.1 (1.1 to 3.3) | 0.3 (0.2 to 0.6) | 3.8 (2 to 6.1) | 18.2 (-14.4 to 59.1) | 22.6 (-28.3 to 111.9) | 15.8 (-18.7 to 58.3) |
|  |  |  | All ages | 24.8 (13.5 to 40.6) | 1.9 (0.9 to 3.2) | 22.9 (12.5 to 37.8) | 90.9 (48.5 to 143.5) | 7.3 (3.6 to 13.1) | 83.7 (44.9 to 131.6) | 266.9 (173.6 to 391.6) | 293.5 (123.3 to 569.6) | 264.8 (161.9 to 400.2) |
|  |  | Deaths | Age-standardized | 0 (0 to 0.1) | 0 (0 to 0) | 0.1 (0 to 0.2) | 0.1 (0 to 0.1) | 0 (0 to 0) | 0.1 (0.1 to 0.2) | 29.7 (-24.1 to 119.7) | 32.9 (-37 to 206.4) | 16.9 (-34.3 to 107.3) |
|  |  |  | All ages | 0.5 (0.2 to 0.7) | 0 (0 to 0.1) | 0.4 (0.2 to 0.7) | 2.2 (1.1 to 3.5) | 0.2 (0.1 to 0.3) | 2 (1 to 3.4) | 379.1 (185.6 to 701.5) | 380.3 (146.6 to 944.4) | 379.1 (173.2 to 735.8) |
| Gilan | All risk factors | DALYs (Disability-Adjusted Life Years) | Age-standardized | 24.3 (10.9 to 39.7) | 26.9 (10.8 to 44.6) | 19.9 (8.6 to 32.7) | 31.2 (16.8 to 48) | 37.5 (18.6 to 58.9) | 25.2 (13.4 to 39.9) | 28.3 (-1.7 to 74.6) | 39.4 (-1.9 to 106.1) | 26.8 (-1.5 to 87.8) |
|  |  |  | All ages | 207.3 (100 to 334.2) | 123.4 (54.3 to 202) | 83.9 (38.8 to 138.3) | 855.3 (461.7 to 1299.7) | 501.6 (253.1 to 765.2) | 353.7 (191.5 to 556.3) | 312.5 (223 to 442.7) | 306.5 (190.4 to 479.8) | 321.4 (235.9 to 483) |
|  |  | Deaths | Age-standardized | 1.2 (0.5 to 2) | 1.4 (0.5 to 2.4) | 0.7 (0.3 to 1.2) | 1.4 (0.7 to 2.3) | 2.1 (1 to 3.3) | 0.8 (0.4 to 1.4) | 23.6 (-21.5 to 97) | 47.4 (-13 to 152.9) | 16 (-21.9 to 109.9) |
|  |  |  | All ages | 7.3 (3.1 to 12.2) | 5.4 (2.1 to 9.5) | 1.9 (0.8 to 3.1) | 34.3 (17.8 to 53.7) | 23.9 (11.2 to 38.3) | 10.3 (5.3 to 16.6) | 371.7 (209.2 to 626.2) | 345.9 (166.8 to 655.1) | 444.6 (270.3 to 828.3) |
|  | Diet high in sodium | DALYs (Disability-Adjusted Life Years) | Age-standardized | 0.4 (0 to 2.3) | 0.3 (0 to 2.1) | 0.5 (0 to 2.7) | 0.4 (0 to 2.6) | 0.3 (0 to 2.3) | 0.5 (0 to 2.8) | 8.5 (-95.3 to 1065.5) | 7.3 (-438.3 to 94892.5) | 3.8 (-95.3 to 936.7) |
|  |  |  | All ages | 3.7 (0 to 21.6) | 1.4 (0 to 9.9) | 2.3 (0 to 11.8) | 11.6 (0 to 71.7) | 4.1 (0 to 31.1) | 7.5 (0 to 40) | 214.4 (-87.3 to 2631.3) | 202 (-1468.4 to 153464.2) | 221.6 (-87.8 to 2426.5) |
|  |  | Deaths | Age-standardized | 0 (0 to 0.1) | 0 (0 to 0.1) | 0 (0 to 0.1) | 0 (0 to 0.1) | 0 (0 to 0.1) | 0 (0 to 0.1) | 7.4 (-95.7 to 2757.6) | 15.5 (-608.9 to 82676.1) | -4.3 (-96.4 to 2283.1) |
|  |  |  | All ages | 0.1 (0 to 0.7) | 0.1 (0 to 0.4) | 0 (0 to 0.3) | 0.4 (0 to 2.7) | 0.2 (0 to 1.4) | 0.2 (0 to 1.1) | 273.8 (-89.7 to 5436.9) | 240.7 (-3253.1 to 167478.4) | 308.8 (-91.8 to 5416.6) |
|  | High alcohol use | DALYs (Disability-Adjusted Life Years) | Age-standardized | 0 (0 to 0) | 0 (0 to 0) | 0 (0 to 0) | 0.2 (0.1 to 0.3) | 0 (0 to 0.1) | 0.4 (0.2 to 0.6) | NA | NA | NA |
|  |  |  | All ages | 0 (0 to 0) | 0 (0 to 0) | 0 (0 to 0) | 6.2 (3.5 to 9.6) | 0.5 (0.2 to 1.1) | 5.7 (3.2 to 8.8) | NA | NA | NA |
|  |  | Deaths | Age-standardized | 0 (0 to 0) | 0 (0 to 0) | 0 (0 to 0) | 0 (0 to 0) | 0 (0 to 0) | 0 (0 to 0) | NA | NA | NA |
|  |  |  | All ages | 0 (0 to 0) | 0 (0 to 0) | 0 (0 to 0) | 0.1 (0.1 to 0.2) | 0 (0 to 0) | 0.1 (0.1 to 0.2) | NA | NA | NA |
|  | High body-mass index | DALYs (Disability-Adjusted Life Years) | Age-standardized | 4.7 (1.8 to 8.8) | 6.6 (2.6 to 12) | 2 (0.6 to 4) | 12.8 (5.2 to 22.1) | 17.2 (7.2 to 29.9) | 8.5 (3.2 to 15.3) | 170.2 (52.6 to 336.9) | 159.3 (35.7 to 343.6) | 317.8 (139.8 to 807.7) |
|  |  |  | All ages | 43.7 (18 to 76.4) | 33.2 (13.4 to 58.9) | 10.6 (3.7 to 20.1) | 349.9 (144.4 to 600.7) | 231.5 (98.7 to 394.6) | 118.5 (46.8 to 212.5) | 699.9 (386.8 to 1065.3) | 597.7 (299.4 to 1017.6) | 1020.9 (567.8 to 1918.5) |
|  |  | Deaths | Age-standardized | 0.2 (0.1 to 0.5) | 0.3 (0.1 to 0.7) | 0.1 (0 to 0.2) | 0.6 (0.3 to 1.1) | 0.9 (0.4 to 1.7) | 0.3 (0.1 to 0.6) | 177.7 (27.3 to 499.3) | 200.6 (21.9 to 580) | 386.4 (102.3 to 1777.6) |
|  |  |  | All ages | 1.4 (0.6 to 2.8) | 1.2 (0.5 to 2.5) | 0.2 (0.1 to 0.4) | 14.3 (6 to 25.5) | 10.7 (4.4 to 19.1) | 3.6 (1.4 to 6.8) | 887.4 (395 to 1748.3) | 753.8 (268.1 to 1647.6) | 1736.3 (809.6 to 4604.6) |
|  | High systolic blood pressure | DALYs (Disability-Adjusted Life Years) | Age-standardized | 20.7 (7 to 35.9) | 23.1 (7.6 to 40.8) | 16.7 (5.1 to 29.7) | 23.3 (8.3 to 41.5) | 28.4 (9.5 to 50.6) | 18.4 (6.2 to 33.3) | 12.4 (-14.3 to 47.1) | 22.7 (-14.5 to 77.9) | 10.2 (-16.3 to 52.8) |
|  |  |  | All ages | 170.3 (60.3 to 298.5) | 103.9 (35.7 to 185.8) | 66.4 (20.4 to 119) | 633.2 (222.4 to 1129.6) | 378.1 (124.1 to 655.1) | 255.1 (86.9 to 459.4) | 271.7 (188.5 to 375.2) | 263.9 (150.5 to 412.7) | 284 (195.5 to 427.9) |
|  |  | Deaths | Age-standardized | 1 (0.3 to 1.9) | 1.2 (0.4 to 2.3) | 0.6 (0.2 to 1.2) | 1.1 (0.4 to 1.9) | 1.6 (0.5 to 2.9) | 0.6 (0.2 to 1.1) | 6.5 (-31.7 to 63.9) | 27.9 (-26.5 to 119.6) | -1.9 (-35.6 to 73.4) |
|  |  |  | All ages | 6.3 (2 to 11.4) | 4.7 (1.4 to 8.8) | 1.6 (0.5 to 2.9) | 26 (9.6 to 46) | 18.3 (6.1 to 32.5) | 7.7 (2.6 to 14) | 314.5 (172.7 to 521.2) | 292.6 (127 to 560.4) | 378.4 (217.2 to 742.2) |
|  | Lead exposure | DALYs (Disability-Adjusted Life Years) | Age-standardized | 3.7 (-0.5 to 9.2) | 3.8 (-0.6 to 9.9) | 3.4 (-0.5 to 8.4) | 4 (-0.5 to 10.1) | 4.3 (-0.6 to 11) | 3.8 (-0.5 to 9.2) | 8.5 (-15.6 to 37.6) | 13 (-23.6 to 55.3) | 9.4 (-11.6 to 44.3) |
|  |  |  | All ages | 30.8 (-4.1 to 75.8) | 17.1 (-2.5 to 44.3) | 13.7 (-1.7 to 33.7) | 105.1 (-14.1 to 264.3) | 54.6 (-7.5 to 140.4) | 50.5 (-6.9 to 125.4) | 241.2 (168.8 to 325.2) | 218.9 (120.3 to 329.9) | 269.2 (203.7 to 387.5) |
|  |  | Deaths | Age-standardized | 0.2 (0 to 0.5) | 0.2 (0 to 0.6) | 0.1 (0 to 0.4) | 0.2 (0 to 0.5) | 0.3 (0 to 0.7) | 0.1 (0 to 0.4) | 12.1 (-25.8 to 61.9) | 29.4 (-24.1 to 102.4) | 7.2 (-27.2 to 86.4) |
|  |  |  | All ages | 1.1 (-0.2 to 3) | 0.8 (-0.1 to 2.1) | 0.3 (0 to 0.9) | 4.7 (-0.7 to 11.9) | 2.9 (-0.4 to 7.7) | 1.7 (-0.3 to 4.3) | 320.7 (181 to 510.5) | 280.2 (124.6 to 490.3) | 414.4 (256.5 to 779.8) |
|  | Smoking | DALYs (Disability-Adjusted Life Years) | Age-standardized | 1.7 (1 to 2.7) | 0.7 (0.3 to 1.1) | 3.1 (1.7 to 4.8) | 2.1 (1.1 to 3.3) | 0.7 (0.3 to 1.4) | 3.4 (1.9 to 5.5) | 18.7 (-6.4 to 53.9) | 11.8 (-37.2 to 102.7) | 12 (-14.6 to 47.9) |
|  |  |  | All ages | 21.2 (11.6 to 33.3) | 4 (2 to 6.7) | 17.2 (9.2 to 27.1) | 64.8 (35.5 to 103.8) | 11.7 (5.5 to 20.9) | 53.1 (29.5 to 85.8) | 205.4 (132.7 to 299) | 190.2 (68.6 to 417.6) | 208.9 (132.2 to 308.6) |
|  |  | Deaths | Age-standardized | 0 (0 to 0.1) | 0 (0 to 0) | 0.1 (0 to 0.1) | 0.1 (0 to 0.1) | 0 (0 to 0.1) | 0.1 (0 to 0.1) | 20.7 (-23.1 to 102.2) | 19.3 (-52.7 to 195.4) | 1.6 (-39.4 to 79.7) |
|  |  |  | All ages | 0.4 (0.2 to 0.6) | 0.1 (0 to 0.2) | 0.3 (0.1 to 0.5) | 1.4 (0.7 to 2.3) | 0.3 (0.1 to 0.7) | 1.1 (0.5 to 1.8) | 268.6 (142 to 490.1) | 227.5 (41.9 to 607.3) | 284.3 (125.5 to 526.1) |
| Golestan | All risk factors | DALYs (Disability-Adjusted Life Years) | Age-standardized | 22.7 (9.9 to 36.2) | 25.8 (11.3 to 41.7) | 18.8 (7.8 to 31.2) | 32.3 (17.3 to 48.8) | 37.9 (19.6 to 58) | 26.1 (13.7 to 39.8) | 42.1 (12.5 to 97.4) | 47.2 (6.8 to 114.8) | 38.5 (7.2 to 98.9) |
|  |  |  | All ages | 80.8 (37.4 to 127) | 44.8 (20.5 to 72.5) | 36 (16.2 to 59.9) | 415.1 (221.1 to 620.4) | 252.6 (129.8 to 388) | 162.5 (87.7 to 247.2) | 413.7 (315 to 589.5) | 463.5 (315.4 to 678.7) | 351.6 (249 to 515.2) |
|  |  | Deaths | Age-standardized | 1 (0.4 to 1.6) | 1.3 (0.5 to 2.1) | 0.7 (0.2 to 1.1) | 1.4 (0.7 to 2.2) | 2 (1 to 3) | 0.9 (0.5 to 1.4) | 44.1 (-0.6 to 124.7) | 56.1 (1.6 to 148.1) | 37.9 (-7.8 to 160.8) |
|  |  |  | All ages | 2.4 (1 to 3.8) | 1.7 (0.7 to 2.7) | 0.8 (0.3 to 1.3) | 15.7 (8.1 to 23.7) | 11 (5.3 to 16.9) | 4.7 (2.4 to 7.2) | 549.7 (359.7 to 876.4) | 565.8 (338 to 946.4) | 514.6 (311.5 to 982.8) |
|  | Diet high in sodium | DALYs (Disability-Adjusted Life Years) | Age-standardized | 0.4 (0 to 2.3) | 0.3 (0 to 2.1) | 0.5 (0 to 2.8) | 0.4 (0 to 2.8) | 0.3 (0 to 2.4) | 0.6 (0 to 3.2) | 9.7 (-95.6 to 1574.9) | 10.3 (-2871.5 to 2068909.4) | 11.2 (-96.1 to 1700.2) |
|  |  |  | All ages | 1.6 (0 to 9.2) | 0.5 (0 to 3.9) | 1.1 (0 to 5.2) | 5.7 (0 to 36.1) | 2.1 (0 to 16.2) | 3.6 (0 to 19.3) | 254.2 (-84.3 to 4510.1) | 297.4 (-185011.5 to 7660549.1) | 232.8 (-89.2 to 4669.8) |
|  |  | Deaths | Age-standardized | 0 (0 to 0.1) | 0 (0 to 0.1) | 0 (0 to 0.1) | 0 (0 to 0.1) | 0 (0 to 0.1) | 0 (0 to 0.1) | 15.2 (-97.2 to 2316.4) | 17.3 (-1812 to 640323.5) | 11.5 (-97.8 to 2461.1) |
|  |  |  | All ages | 0 (0 to 0.2) | 0 (0 to 0.1) | 0 (0 to 0.1) | 0.2 (0 to 1.2) | 0.1 (0 to 0.7) | 0.1 (0 to 0.5) | 349.1 (-89.9 to 4830.5) | 366 (-426196.7 to 2574811.5) | 335.1 (-93.8 to 5912.5) |
|  | High alcohol use | DALYs (Disability-Adjusted Life Years) | Age-standardized | 0 (0 to 0) | 0 (0 to 0) | 0 (0 to 0) | 0.2 (0.1 to 0.3) | 0 (0 to 0.1) | 0.4 (0.2 to 0.6) | NA | NA | NA |
|  |  |  | All ages | 0 (0 to 0) | 0 (0 to 0) | 0 (0 to 0) | 2.9 (1.6 to 4.7) | 0.3 (0.1 to 0.5) | 2.7 (1.5 to 4.2) | NA | NA | NA |
|  |  | Deaths | Age-standardized | 0 (0 to 0) | 0 (0 to 0) | 0 (0 to 0) | 0 (0 to 0) | 0 (0 to 0) | 0 (0 to 0) | NA | NA | NA |
|  |  |  | All ages | 0 (0 to 0) | 0 (0 to 0) | 0 (0 to 0) | 0.1 (0 to 0.1) | 0 (0 to 0) | 0.1 (0 to 0.1) | NA | NA | NA |
|  | High body-mass index | DALYs (Disability-Adjusted Life Years) | Age-standardized | 3.4 (1.3 to 6.2) | 5.2 (1.9 to 9.3) | 1.4 (0.4 to 2.8) | 11.9 (5 to 20.6) | 14.9 (6.1 to 26.5) | 8.6 (3.4 to 15.1) | 250.5 (88.9 to 498) | 188.9 (46.1 to 409.3) | 500 (227.7 to 1367.1) |
|  |  |  | All ages | 14.3 (5.5 to 24.5) | 10.6 (4.2 to 17.9) | 3.7 (1.2 to 7.1) | 155.6 (67.3 to 271.6) | 102 (42.9 to 178.5) | 53.6 (21.4 to 94.5) | 986.1 (549.2 to 1524.1) | 858.4 (430 to 1341.5) | 1355.1 (734.9 to 3061) |
|  |  | Deaths | Age-standardized | 0.1 (0 to 0.3) | 0.2 (0.1 to 0.5) | 0 (0 to 0.1) | 0.5 (0.2 to 0.9) | 0.7 (0.3 to 1.3) | 0.3 (0.1 to 0.5) | 288.1 (75.2 to 843) | 247.8 (51.9 to 745.2) | 734.6 (214.8 to 3356.5) |
|  |  |  | All ages | 0.4 (0.1 to 0.7) | 0.3 (0.1 to 0.6) | 0.1 (0 to 0.1) | 5.7 (2.3 to 10.2) | 4.2 (1.7 to 7.5) | 1.6 (0.6 to 2.8) | 1386.7 (663.2 to 2574.8) | 1184.8 (517 to 2271.5) | 2462.1 (1121.8 to 6404.3) |
|  | High systolic blood pressure | DALYs (Disability-Adjusted Life Years) | Age-standardized | 19.8 (6.7 to 33.6) | 22.7 (8.3 to 38.6) | 16 (4.8 to 28.3) | 24.9 (8.6 to 41.1) | 30.2 (10.3 to 50.6) | 19.1 (6.3 to 33.1) | 26 (-2.4 to 59.5) | 32.8 (-3.2 to 84) | 18.9 (-8.1 to 58.9) |
|  |  |  | All ages | 66.9 (22.2 to 114.4) | 38.3 (14 to 65.7) | 28.6 (8.5 to 52.3) | 314.3 (110 to 517.8) | 199 (69.7 to 335) | 115.3 (39.4 to 200.3) | 369.4 (272.1 to 499.9) | 419.3 (275.3 to 615.8) | 302.7 (206.6 to 443.3) |
|  |  | Deaths | Age-standardized | 0.9 (0.3 to 1.5) | 1.1 (0.4 to 2) | 0.6 (0.2 to 1.1) | 1.1 (0.4 to 1.9) | 1.6 (0.5 to 2.7) | 0.7 (0.2 to 1.2) | 26.3 (-11.6 to 83.1) | 39.1 (-8.7 to 117.4) | 15.8 (-24.8 to 94.3) |
|  |  |  | All ages | 2.1 (0.7 to 3.5) | 1.5 (0.5 to 2.5) | 0.6 (0.2 to 1.2) | 12.3 (4.4 to 20.6) | 8.9 (3 to 14.9) | 3.5 (1.1 to 6.1) | 484.7 (307.1 to 719.6) | 505.2 (290 to 815.1) | 437.9 (244.6 to 791.1) |
|  | Lead exposure | DALYs (Disability-Adjusted Life Years) | Age-standardized | 4.2 (-0.6 to 10.7) | 4.3 (-0.6 to 11.3) | 4 (-0.6 to 10.1) | 4.7 (-0.7 to 11.6) | 4.7 (-0.7 to 12.1) | 4.7 (-0.7 to 11.8) | 12.1 (-10.8 to 38.6) | 10.1 (-22.8 to 47) | 17.1 (-2.9 to 53.9) |
|  |  |  | All ages | 14.7 (-2.1 to 37.3) | 7.4 (-1.1 to 19.4) | 7.3 (-1 to 18.5) | 57 (-8.3 to 142) | 29.8 (-4.3 to 77.4) | 27.3 (-3.9 to 67.8) | 288.9 (214.8 to 379.6) | 301.9 (192 to 435.3) | 275.6 (208.4 to 386.3) |
|  |  | Deaths | Age-standardized | 0.2 (0 to 0.5) | 0.2 (0 to 0.5) | 0.1 (0 to 0.4) | 0.2 (0 to 0.6) | 0.3 (0 to 0.7) | 0.2 (0 to 0.5) | 20.8 (-15.5 to 66.5) | 26.1 (-22.1 to 82.1) | 22.4 (-12.4 to 106) |
|  |  |  | All ages | 0.4 (-0.1 to 1.1) | 0.3 (0 to 0.7) | 0.2 (0 to 0.4) | 2.3 (-0.4 to 5.8) | 1.4 (-0.2 to 3.7) | 0.9 (-0.2 to 2.3) | 434.2 (279.5 to 643.3) | 419.3 (224.1 to 655.5) | 460.1 (297.7 to 836.2) |
|  | Smoking | DALYs (Disability-Adjusted Life Years) | Age-standardized | 1.7 (0.9 to 2.8) | 0.6 (0.3 to 1.1) | 2.8 (1.6 to 4.6) | 2 (1.2 to 3.2) | 0.8 (0.4 to 1.4) | 3.5 (1.9 to 5.6) | 19.8 (-4.3 to 57) | 19.9 (-27.5 to 113.7) | 23.7 (-3.6 to 63.6) |
|  |  |  | All ages | 9 (4.9 to 14.3) | 1.6 (0.8 to 2.6) | 7.4 (4.1 to 12.1) | 32.7 (18.3 to 51.8) | 6.3 (3.2 to 11.2) | 26.4 (14.8 to 42.1) | 263.9 (188.2 to 369.5) | 299.6 (131.7 to 606.3) | 256.3 (176 to 365.6) |
|  |  | Deaths | Age-standardized | 0 (0 to 0.1) | 0 (0 to 0) | 0.1 (0 to 0.1) | 0.1 (0 to 0.1) | 0 (0 to 0.1) | 0.1 (0 to 0.1) | 28.3 (-16.8 to 115.2) | 25.9 (-44.6 to 188.2) | 24.2 (-21.3 to 116.8) |
|  |  |  | All ages | 0.2 (0.1 to 0.2) | 0 (0 to 0.1) | 0.1 (0.1 to 0.2) | 0.7 (0.4 to 1.1) | 0.2 (0.1 to 0.3) | 0.5 (0.3 to 0.8) | 347.2 (203.6 to 626.5) | 363.1 (141.7 to 831.3) | 342 (181.7 to 647.8) |
| Hamadan | All risk factors | DALYs (Disability-Adjusted Life Years) | Age-standardized | 18.5 (8.3 to 31.1) | 21.1 (8.9 to 36.5) | 16.1 (6.7 to 27.2) | 24.8 (12.4 to 38.8) | 29.5 (14.2 to 47.4) | 21.2 (11 to 32.6) | 33.8 (1.6 to 92.3) | 39.5 (-3.3 to 116.1) | 31.4 (-1.4 to 99.4) |
|  |  |  | All ages | 108.1 (50.5 to 176) | 55.6 (24.5 to 93.6) | 52.6 (23.7 to 87.1) | 440.4 (219.8 to 679.3) | 244.7 (118.7 to 395.2) | 195.6 (101.9 to 298.9) | 307.2 (220.2 to 463.8) | 340.4 (205.7 to 559.1) | 272.1 (178.2 to 436.4) |
|  |  | Deaths | Age-standardized | 0.8 (0.3 to 1.4) | 1.1 (0.4 to 1.9) | 0.5 (0.2 to 0.9) | 1 (0.5 to 1.6) | 1.6 (0.7 to 2.5) | 0.6 (0.3 to 1) | 29.1 (-14.5 to 114.6) | 45.4 (-13.3 to 152.4) | 16.8 (-23.4 to 137.4) |
|  |  |  | All ages | 3.4 (1.3 to 5.7) | 2.1 (0.9 to 3.8) | 1.2 (0.4 to 2.2) | 18.2 (8.4 to 29.1) | 12 (5.3 to 19.7) | 6.1 (2.7 to 9.9) | 436.7 (263.4 to 769.4) | 462.5 (243.6 to 853.9) | 392.3 (210.1 to 858.8) |
|  | Diet high in sodium | DALYs (Disability-Adjusted Life Years) | Age-standardized | 0.4 (0 to 2.1) | 0.3 (0 to 1.8) | 0.5 (0 to 2.4) | 0.4 (0 to 2.2) | 0.3 (0 to 2.1) | 0.5 (0 to 2.6) | 4.4 (-97.2 to 1023.8) | 7.3 (-861.1 to 29017.5) | 5 (-97 to 1087.5) |
|  |  |  | All ages | 2.4 (0 to 13.1) | 0.7 (0 to 5.1) | 1.6 (0 to 8.4) | 6.6 (0 to 39.2) | 2.3 (0 to 17.3) | 4.3 (0 to 23.5) | 180.2 (-94.3 to 2460.7) | 208.1 (-18443.8 to 36641) | 167.4 (-93.6 to 2497.5) |
|  |  | Deaths | Age-standardized | 0 (0 to 0.1) | 0 (0 to 0.1) | 0 (0 to 0.1) | 0 (0 to 0.1) | 0 (0 to 0.1) | 0 (0 to 0.1) | 2.1 (-98 to 846.4) | 13.6 (-548.3 to 35247.2) | -5.4 (-97.8 to 910.7) |
|  |  |  | All ages | 0.1 (0 to 0.3) | 0 (0 to 0.2) | 0 (0 to 0.2) | 0.2 (0 to 1.5) | 0.1 (0 to 0.8) | 0.1 (0 to 0.7) | 268 (-95.3 to 2884.8) | 295.1 (-98079.2 to 41210.8) | 248.7 (-95.1 to 2915.3) |
|  | High alcohol use | DALYs (Disability-Adjusted Life Years) | Age-standardized | 0 (0 to 0) | 0 (0 to 0) | 0 (0 to 0) | 0.2 (0.1 to 0.3) | 0 (0 to 0.1) | 0.3 (0.2 to 0.5) | NA | NA | NA |
|  |  |  | All ages | 0 (0 to 0) | 0 (0 to 0) | 0 (0 to 0) | 3.3 (1.7 to 5.3) | 0.3 (0.1 to 0.6) | 3 (1.6 to 4.9) | NA | NA | NA |
|  |  | Deaths | Age-standardized | 0 (0 to 0) | 0 (0 to 0) | 0 (0 to 0) | 0 (0 to 0) | 0 (0 to 0) | 0 (0 to 0) | NA | NA | NA |
|  |  |  | All ages | 0 (0 to 0) | 0 (0 to 0) | 0 (0 to 0) | 0.1 (0 to 0.1) | 0 (0 to 0) | 0.1 (0 to 0.1) | NA | NA | NA |
|  | High body-mass index | DALYs (Disability-Adjusted Life Years) | Age-standardized | 2.7 (1 to 5.1) | 4.5 (1.7 to 8.6) | 1.1 (0.3 to 2.2) | 9.7 (3.8 to 16.9) | 12.9 (4.9 to 22.6) | 6.8 (2.6 to 12.1) | 253.7 (100.3 to 501.3) | 185.7 (41.8 to 418) | 530.4 (218.7 to 1730.9) |
|  |  |  | All ages | 18.4 (7.4 to 32.2) | 14 (5.7 to 24.8) | 4.4 (1.3 to 8.5) | 172.1 (67.6 to 300.2) | 107.9 (41.2 to 188.9) | 64.1 (24.2 to 113.4) | 836.9 (475.9 to 1328) | 672.8 (322 to 1143.1) | 1357.8 (678.6 to 3674.7) |
|  |  | Deaths | Age-standardized | 0.1 (0 to 0.2) | 0.2 (0.1 to 0.4) | 0 (0 to 0.1) | 0.4 (0.2 to 0.7) | 0.6 (0.2 to 1.2) | 0.2 (0.1 to 0.4) | 283.2 (74.8 to 789.6) | 245.4 (35.5 to 808.5) | 697 (187.7 to 4114.7) |
|  |  |  | All ages | 0.5 (0.2 to 1) | 0.4 (0.2 to 0.9) | 0.1 (0 to 0.2) | 7.2 (2.7 to 12.7) | 5 (1.9 to 9.4) | 2.1 (0.7 to 3.9) | 1297.3 (641.1 to 2526.7) | 1064 (440.4 to 2347.8) | 2584.5 (1004.7 to 9414.2) |
|  | High systolic blood pressure | DALYs (Disability-Adjusted Life Years) | Age-standardized | 15.8 (5.3 to 28.2) | 18.3 (6 to 33.4) | 13.5 (4 to 24.5) | 18.2 (6 to 31.9) | 22.1 (7.5 to 39.6) | 15.1 (4.7 to 27) | 15.3 (-13.1 to 60.1) | 21 (-18.6 to 89) | 12.4 (-17.1 to 60.3) |
|  |  |  | All ages | 88.5 (29.1 to 155.6) | 46.5 (15.1 to 83.5) | 42 (11.8 to 77.3) | 321.5 (107.4 to 562.2) | 183.1 (61.3 to 329.8) | 138.4 (43 to 245.4) | 263.4 (172.5 to 392.7) | 293.6 (159 to 518.6) | 229.9 (140.3 to 366.8) |
|  |  | Deaths | Age-standardized | 0.7 (0.2 to 1.3) | 1 (0.3 to 1.8) | 0.5 (0.1 to 0.9) | 0.8 (0.3 to 1.4) | 1.2 (0.4 to 2.2) | 0.4 (0.1 to 0.8) | 9.6 (-27.7 to 72.2) | 24.5 (-27.9 to 109.6) | -2.6 (-39.8 to 87.7) |
|  |  |  | All ages | 2.9 (0.9 to 5.4) | 1.9 (0.6 to 3.5) | 1.1 (0.3 to 2) | 13.7 (4.6 to 24.4) | 9.2 (3 to 16.8) | 4.5 (1.4 to 8.2) | 366.3 (209.4 to 630.9) | 391.9 (186.1 to 751.5) | 321.3 (154.7 to 724.9) |
|  | Lead exposure | DALYs (Disability-Adjusted Life Years) | Age-standardized | 4.1 (-0.6 to 10.1) | 4.3 (-0.6 to 11.2) | 3.8 (-0.5 to 9.5) | 4.4 (-0.6 to 10.8) | 4.9 (-0.7 to 11.9) | 4.1 (-0.6 to 10.4) | 8.3 (-14.1 to 36.4) | 12.3 (-18.9 to 50.4) | 7.9 (-13 to 44.5) |
|  |  |  | All ages | 23 (-3 to 58.4) | 11.2 (-1.5 to 28.7) | 11.8 (-1.5 to 30.5) | 77.6 (-10.6 to 191.5) | 39.5 (-5.7 to 97.4) | 38.1 (-5.3 to 95.8) | 237 (170.4 to 319.6) | 252.7 (158.5 to 369.1) | 222 (156 to 321) |
|  |  | Deaths | Age-standardized | 0.2 (0 to 0.4) | 0.2 (0 to 0.6) | 0.1 (0 to 0.3) | 0.2 (0 to 0.5) | 0.3 (0 to 0.7) | 0.1 (0 to 0.3) | 11 (-22.6 to 63.5) | 26.2 (-20.1 to 86.4) | 1 (-31.7 to 83.4) |
|  |  |  | All ages | 0.7 (-0.1 to 1.8) | 0.4 (-0.1 to 1.1) | 0.3 (0 to 0.8) | 3.5 (-0.6 to 8.5) | 2.1 (-0.3 to 5.2) | 1.4 (-0.2 to 3.4) | 373.4 (233.7 to 583.8) | 387.5 (206.2 to 625.3) | 353.1 (204.8 to 710.5) |
|  | Smoking | DALYs (Disability-Adjusted Life Years) | Age-standardized | 1.7 (1 to 2.7) | 0.6 (0.3 to 1) | 2.8 (1.5 to 4.3) | 1.9 (1.1 to 3) | 0.7 (0.3 to 1.2) | 3.2 (1.8 to 5) | 13.6 (-12.7 to 51.5) | 13.7 (-38.2 to 92.9) | 16.3 (-10.5 to 59.4) |
|  |  |  | All ages | 13.2 (7.3 to 20.7) | 2.2 (1.1 to 3.8) | 11 (6.1 to 17.4) | 36.4 (20.6 to 56.9) | 6.1 (3 to 10.6) | 30.3 (16.8 to 47.4) | 175.9 (110.3 to 263.7) | 181.1 (62.9 to 375.6) | 174.8 (105.6 to 283.8) |
|  |  | Deaths | Age-standardized | 0 (0 to 0.1) | 0 (0 to 0) | 0.1 (0 to 0.1) | 0 (0 to 0.1) | 0 (0 to 0) | 0.1 (0 to 0.1) | 8.9 (-33.3 to 99.1) | 19.5 (-52 to 175.4) | 4.5 (-40.8 to 104.6) |
|  |  |  | All ages | 0.2 (0.1 to 0.4) | 0.1 (0 to 0.1) | 0.2 (0.1 to 0.3) | 0.8 (0.4 to 1.3) | 0.2 (0.1 to 0.4) | 0.7 (0.3 to 1.1) | 250.7 (118.4 to 522.2) | 267 (49.9 to 644.4) | 246.2 (94.3 to 571.3) |
| Hormozgan | All risk factors | DALYs (Disability-Adjusted Life Years) | Age-standardized | 20 (8.3 to 33) | 24.3 (9.8 to 40.7) | 14.8 (6.4 to 25.9) | 26.1 (13.6 to 40.5) | 32.5 (16 to 51.2) | 19.7 (10.4 to 30.9) | 30.9 (-1.5 to 87.9) | 34 (-6.7 to 99.4) | 32.6 (-3.5 to 98.2) |
|  |  |  | All ages | 58.8 (25.1 to 94.6) | 33.1 (13.7 to 55.1) | 25.7 (11.4 to 43.1) | 264.8 (138.8 to 411.8) | 158.9 (79.1 to 249.7) | 105.9 (56.4 to 164.1) | 350.3 (249.8 to 513.7) | 380.1 (238.4 to 595.6) | 311.9 (207.1 to 493.2) |
|  |  | Deaths | Age-standardized | 0.8 (0.3 to 1.5) | 1.1 (0.4 to 2.1) | 0.5 (0.2 to 0.9) | 1.1 (0.6 to 1.7) | 1.6 (0.8 to 2.7) | 0.6 (0.3 to 1) | 32.1 (-12.5 to 123.4) | 43.2 (-9.9 to 148.5) | 27 (-28.8 to 179.4) |
|  |  |  | All ages | 1.8 (0.7 to 3.2) | 1.3 (0.5 to 2.4) | 0.5 (0.2 to 1) | 9.6 (4.8 to 14.9) | 7 (3.2 to 11.1) | 2.6 (1.2 to 4.3) | 420 (250.1 to 763.2) | 436.3 (235.9 to 796.5) | 380.6 (191 to 892.4) |
|  | Diet high in sodium | DALYs (Disability-Adjusted Life Years) | Age-standardized | 0.4 (0 to 2.2) | 0.3 (0 to 1.9) | 0.5 (0 to 2.6) | 0.4 (0 to 2.3) | 0.3 (0 to 2) | 0.5 (0 to 2.7) | 3.2 (-96.3 to 1481.5) | 2.9 (-212331.9 to 74705.9) | 4.6 (-96.5 to 1532) |
|  |  |  | All ages | 1.2 (0 to 6.6) | 0.4 (0 to 2.7) | 0.8 (0 to 4.2) | 4.1 (0 to 22.9) | 1.4 (0 to 10.3) | 2.7 (0 to 13.6) | 235.8 (-80.5 to 4948.5) | 258 (-383867.6 to 431217.4) | 225.4 (-82.6 to 4973.8) |
|  |  | Deaths | Age-standardized | 0 (0 to 0.1) | 0 (0 to 0.1) | 0 (0 to 0.1) | 0 (0 to 0.1) | 0 (0 to 0.1) | 0 (0 to 0.1) | 4.2 (-97.9 to 2018.8) | 9.2 (-1130.1 to 108420.4) | -2.1 (-98.3 to 2084.9) |
|  |  |  | All ages | 0 (0 to 0.2) | 0 (0 to 0.1) | 0 (0 to 0.1) | 0.1 (0 to 0.8) | 0.1 (0 to 0.4) | 0.1 (0 to 0.4) | 280.1 (-89.2 to 6445.4) | 290.8 (-157205.7 to 485401.6) | 272.1 (-91 to 6461.3) |
|  | High alcohol use | DALYs (Disability-Adjusted Life Years) | Age-standardized | 0 (0 to 0) | 0 (0 to 0) | 0 (0 to 0) | 0.2 (0.1 to 0.3) | 0 (0 to 0.1) | 0.3 (0.2 to 0.5) | NA | NA | NA |
|  |  |  | All ages | 0 (0 to 0) | 0 (0 to 0) | 0 (0 to 0) | 2.1 (1.2 to 3.3) | 0.2 (0 to 0.3) | 2 (1.1 to 3.1) | NA | NA | NA |
|  |  | Deaths | Age-standardized | 0 (0 to 0) | 0 (0 to 0) | 0 (0 to 0) | 0 (0 to 0) | 0 (0 to 0) | 0 (0 to 0) | NA | NA | NA |
|  |  |  | All ages | 0 (0 to 0) | 0 (0 to 0) | 0 (0 to 0) | 0 (0 to 0.1) | 0 (0 to 0) | 0 (0 to 0.1) | NA | NA | NA |
|  | High body-mass index | DALYs (Disability-Adjusted Life Years) | Age-standardized | 2.8 (1 to 5.2) | 4.6 (1.7 to 8.6) | 1 (0.3 to 2.3) | 9.5 (3.8 to 17.1) | 12.2 (4.9 to 21.9) | 6.7 (2.7 to 12.1) | 241.4 (96.3 to 523.3) | 167 (33.8 to 369.8) | 571.8 (235.5 to 2032.8) |
|  |  |  | All ages | 9 (3.3 to 15.7) | 7 (2.6 to 12.6) | 2 (0.6 to 4) | 96.3 (38.9 to 170.7) | 61.6 (24.8 to 110) | 34.7 (14.5 to 64) | 970.5 (551.6 to 1604.4) | 780.4 (389.6 to 1341.1) | 1634.5 (903.6 to 4758.9) |
|  |  | Deaths | Age-standardized | 0.1 (0 to 0.2) | 0.2 (0.1 to 0.4) | 0 (0 to 0.1) | 0.4 (0.2 to 0.7) | 0.6 (0.2 to 1.1) | 0.2 (0.1 to 0.5) | 277.6 (74.4 to 887.9) | 223 (37.4 to 739.3) | 840.7 (188.5 to 5582.2) |
|  |  |  | All ages | 0.3 (0.1 to 0.5) | 0.2 (0.1 to 0.5) | 0 (0 to 0.1) | 3.6 (1.4 to 6.4) | 2.5 (1 to 4.7) | 1 (0.4 to 1.9) | 1247.3 (607.7 to 2632.4) | 1007.7 (413.3 to 2284.6) | 2777.6 (1078.6 to 10270.2) |
|  | High systolic blood pressure | DALYs (Disability-Adjusted Life Years) | Age-standardized | 17.2 (5.4 to 30.6) | 21.6 (6.8 to 38) | 12 (3.4 to 23.5) | 19.8 (6.8 to 34.8) | 26.1 (8.8 to 46.1) | 13.4 (3.9 to 25) | 15.2 (-11.7 to 54.7) | 21.1 (-15.7 to 77.4) | 12 (-21.2 to 65) |
|  |  |  | All ages | 49.2 (15.6 to 86.7) | 28.9 (9 to 51) | 20.4 (6.2 to 37.9) | 198.1 (66.9 to 345.6) | 126.1 (42.7 to 221) | 72.1 (22.6 to 134) | 302.5 (216 to 428) | 336.6 (200 to 532.9) | 254.1 (157.7 to 421.6) |
|  |  | Deaths | Age-standardized | 0.7 (0.2 to 1.4) | 1 (0.3 to 2) | 0.4 (0.1 to 0.8) | 0.9 (0.3 to 1.5) | 1.3 (0.4 to 2.4) | 0.4 (0.1 to 0.8) | 13.8 (-25.2 to 90.3) | 28.1 (-20 to 118.2) | -2 (-46.3 to 104.3) |
|  |  |  | All ages | 1.6 (0.5 to 3) | 1.2 (0.4 to 2.2) | 0.4 (0.1 to 0.9) | 7.3 (2.4 to 12.7) | 5.7 (1.9 to 10) | 1.7 (0.5 to 3.5) | 355.1 (202 to 617.9) | 384 (195.3 to 725.1) | 278.4 (118.5 to 671.4) |
|  | Lead exposure | DALYs (Disability-Adjusted Life Years) | Age-standardized | 4.4 (-0.6 to 10.7) | 4.4 (-0.6 to 10.6) | 4.3 (-0.6 to 10.6) | 4.7 (-0.6 to 11.7) | 4.7 (-0.7 to 12) | 4.7 (-0.6 to 11.5) | 7.2 (-16.1 to 35.8) | 6.4 (-27.8 to 41.7) | 8.7 (-12.4 to 50.5) |
|  |  |  | All ages | 12.1 (-1.7 to 29.4) | 5.9 (-0.8 to 14.3) | 6.2 (-0.9 to 15.1) | 44.5 (-5.9 to 112.4) | 21.8 (-3.1 to 56.2) | 22.7 (-3 to 56.6) | 268.6 (190.3 to 365.6) | 272.2 (148.9 to 391) | 265.2 (192.4 to 392.1) |
|  |  | Deaths | Age-standardized | 0.2 (0 to 0.5) | 0.2 (0 to 0.5) | 0.2 (0 to 0.4) | 0.2 (0 to 0.6) | 0.3 (0 to 0.7) | 0.2 (0 to 0.5) | 14.5 (-21.6 to 77) | 21 (-27.3 to 83.9) | 9.3 (-26.7 to 117.5) |
|  |  |  | All ages | 0.4 (-0.1 to 1) | 0.2 (0 to 0.6) | 0.2 (0 to 0.4) | 1.9 (-0.3 to 4.7) | 1.1 (-0.2 to 2.7) | 0.8 (-0.1 to 1.9) | 360.5 (216 to 613.7) | 348.9 (170.7 to 577.5) | 377.6 (234.1 to 821.3) |
|  | Smoking | DALYs (Disability-Adjusted Life Years) | Age-standardized | 1.9 (1 to 2.9) | 0.6 (0.3 to 1.1) | 3 (1.7 to 4.7) | 1.9 (1.1 to 3) | 0.6 (0.3 to 1.1) | 3.2 (1.8 to 5.1) | 3.6 (-19.6 to 39.1) | 2 (-40.4 to 78.2) | 6.9 (-18.7 to 49.1) |
|  |  |  | All ages | 6.9 (3.9 to 10.8) | 1.1 (0.5 to 1.9) | 5.8 (3.2 to 9.1) | 23.6 (13.1 to 36.8) | 3.9 (1.9 to 6.9) | 19.7 (10.9 to 30.7) | 240.7 (160.8 to 343.7) | 261.4 (110.4 to 504.9) | 236.8 (149.9 to 348.9) |
|  |  | Deaths | Age-standardized | 0 (0 to 0.1) | 0 (0 to 0) | 0.1 (0 to 0.1) | 0 (0 to 0.1) | 0 (0 to 0) | 0.1 (0 to 0.1) | 4.6 (-33.2 to 94.2) | 5.1 (-53.8 to 141.2) | 0.3 (-39.6 to 114.3) |
|  |  |  | All ages | 0.1 (0.1 to 0.2) | 0 (0 to 0.1) | 0.1 (0 to 0.2) | 0.5 (0.3 to 0.8) | 0.1 (0 to 0.2) | 0.4 (0.2 to 0.6) | 263.9 (142.8 to 561.9) | 269.8 (79.7 to 648.5) | 262.3 (124.3 to 630.2) |
| Ilam | All risk factors | DALYs (Disability-Adjusted Life Years) | Age-standardized | 17.5 (7.4 to 28.6) | 19.2 (8 to 31.9) | 16 (6.5 to 27.5) | 27.6 (15.2 to 41.5) | 30.4 (16.1 to 46.3) | 24.2 (12.6 to 36.5) | 57.7 (20.7 to 137) | 58.4 (7.8 to 150.3) | 51.1 (10.8 to 139) |
|  |  |  | All ages | 21.8 (9.5 to 35.1) | 10.3 (4.4 to 17.4) | 11.5 (4.6 to 19.7) | 112 (61.6 to 167.3) | 64 (35.1 to 96.8) | 48 (25.1 to 73.7) | 414.8 (301.7 to 638.8) | 521.7 (336.6 to 844.5) | 318.8 (214 to 541.1) |
|  |  | Deaths | Age-standardized | 0.7 (0.2 to 1.2) | 0.8 (0.3 to 1.5) | 0.5 (0.2 to 1) | 1.2 (0.7 to 1.9) | 1.5 (0.8 to 2.4) | 0.9 (0.5 to 1.5) | 86.4 (23.4 to 241) | 85.2 (9.3 to 230.5) | 73.5 (2 to 307.7) |
|  |  |  | All ages | 0.7 (0.2 to 1.1) | 0.4 (0.1 to 0.7) | 0.3 (0.1 to 0.5) | 4.1 (2.2 to 6.1) | 2.6 (1.3 to 4.1) | 1.5 (0.7 to 2.3) | 511.5 (309.4 to 984.2) | 607.1 (318.9 to 1154.5) | 391.5 (190.3 to 1021.8) |
|  | Diet high in sodium | DALYs (Disability-Adjusted Life Years) | Age-standardized | 0.4 (0 to 2.1) | 0.2 (0 to 1.6) | 0.5 (0 to 2.4) | 0.4 (0 to 2.3) | 0.3 (0 to 2.1) | 0.5 (0 to 2.9) | 9.5 (-95.5 to 3719.7) | 19 (-1826.5 to 27625.4) | 13.8 (-95 to 3955.6) |
|  |  |  | All ages | 0.5 (0 to 2.6) | 0.1 (0 to 0.9) | 0.3 (0 to 1.7) | 1.7 (0 to 9.6) | 0.6 (0 to 4.5) | 1.1 (0 to 5.9) | 252.9 (-81 to 9017.8) | 370.5 (-37265.3 to 61441.6) | 211.4 (-81.5 to 9185.9) |
|  |  | Deaths | Age-standardized | 0 (0 to 0.1) | 0 (0 to 0.1) | 0 (0 to 0.1) | 0 (0 to 0.1) | 0 (0 to 0.1) | 0 (0 to 0.1) | 27.2 (-97.2 to 8210.9) | 38.7 (-2240.2 to 55955.4) | 26.6 (-96.9 to 8527.3) |
|  |  |  | All ages | 0 (0 to 0.1) | 0 (0 to 0) | 0 (0 to 0) | 0 (0 to 0.3) | 0 (0 to 0.2) | 0 (0 to 0.2) | 313.1 (-89.8 to 15245.2) | 435.1 (-6312.6 to 215113.5) | 254.1 (-91.5 to 16603.2) |
|  | High alcohol use | DALYs (Disability-Adjusted Life Years) | Age-standardized | 0 (0 to 0) | 0 (0 to 0) | 0 (0 to 0) | 0.2 (0.1 to 0.3) | 0 (0 to 0.1) | 0.4 (0.2 to 0.6) | NA | NA | NA |
|  |  |  | All ages | 0 (0 to 0) | 0 (0 to 0) | 0 (0 to 0) | 0.9 (0.5 to 1.4) | 0.1 (0 to 0.1) | 0.8 (0.5 to 1.3) | NA | NA | NA |
|  |  | Deaths | Age-standardized | 0 (0 to 0) | 0 (0 to 0) | 0 (0 to 0) | 0 (0 to 0) | 0 (0 to 0) | 0 (0 to 0) | NA | NA | NA |
|  |  |  | All ages | 0 (0 to 0) | 0 (0 to 0) | 0 (0 to 0) | 0 (0 to 0) | 0 (0 to 0) | 0 (0 to 0) | NA | NA | NA |
|  | High body-mass index | DALYs (Disability-Adjusted Life Years) | Age-standardized | 2.3 (0.8 to 4) | 3.8 (1.4 to 6.7) | 1.1 (0.3 to 2.3) | 11.4 (4.7 to 20) | 13.3 (5.7 to 24) | 9.1 (3.6 to 15.7) | 397 (160.2 to 772.5) | 254.8 (78.8 to 559.1) | 731.3 (297.4 to 2379.7) |
|  |  |  | All ages | 3.2 (1.2 to 5.4) | 2.2 (0.9 to 3.9) | 1 (0.3 to 1.9) | 46.7 (19.4 to 82.2) | 28.7 (12.1 to 51.1) | 18 (7.2 to 31.7) | 1355.2 (717.1 to 2271.1) | 1178.4 (562.4 to 2045.7) | 1766.9 (837.1 to 5017.6) |
|  |  | Deaths | Age-standardized | 0.1 (0 to 0.2) | 0.1 (0 to 0.3) | 0 (0 to 0.1) | 0.5 (0.2 to 0.9) | 0.6 (0.3 to 1.1) | 0.4 (0.1 to 0.7) | 574 (194.6 to 1553.4) | 380 (91.1 to 1211.6) | 1263.1 (311.4 to 8725) |
|  |  |  | All ages | 0.1 (0 to 0.2) | 0.1 (0 to 0.1) | 0 (0 to 0) | 1.7 (0.7 to 3) | 1.1 (0.5 to 2) | 0.6 (0.2 to 1.1) | 1905.9 (866.2 to 3946.9) | 1600.1 (640.3 to 3833) | 3048.3 (1030.6 to 13950.4) |
|  | High systolic blood pressure | DALYs (Disability-Adjusted Life Years) | Age-standardized | 15.1 (4.8 to 26.3) | 16.9 (5.5 to 30) | 13.5 (4.1 to 25) | 20.2 (6.8 to 34.8) | 23.1 (7.9 to 40.6) | 16.7 (5.3 to 29.1) | 33.4 (3.3 to 79.1) | 36.5 (-9.5 to 94.7) | 23.2 (-11 to 87.9) |
|  |  |  | All ages | 18.2 (5.8 to 31.8) | 8.9 (2.9 to 15.9) | 9.3 (2.8 to 17.2) | 80.2 (27.3 to 139.2) | 48.1 (16.7 to 84.6) | 32.1 (10.1 to 57.6) | 341.1 (246.9 to 496.2) | 440.8 (265.5 to 674.3) | 245.6 (154.7 to 406.5) |
|  |  | Deaths | Age-standardized | 0.6 (0.2 to 1.1) | 0.7 (0.2 to 1.4) | 0.5 (0.1 to 0.9) | 0.9 (0.3 to 1.6) | 1.2 (0.4 to 2.1) | 0.7 (0.2 to 1.2) | 55.7 (0.7 to 150.6) | 57.9 (-10.1 to 159.8) | 39.1 (-20.9 to 221.9) |
|  |  |  | All ages | 0.6 (0.2 to 1.1) | 0.3 (0.1 to 0.6) | 0.3 (0.1 to 0.5) | 3.1 (1.1 to 5.2) | 2 (0.7 to 3.5) | 1 (0.3 to 1.9) | 418.5 (244.7 to 729.6) | 508.9 (250.1 to 886.6) | 301.6 (136.6 to 819.8) |
|  | Lead exposure | DALYs (Disability-Adjusted Life Years) | Age-standardized | 4 (-0.6 to 9.9) | 3.8 (-0.6 to 9.6) | 4.1 (-0.6 to 10.7) | 4.8 (-0.7 to 12.3) | 4.7 (-0.7 to 11.5) | 5 (-0.7 to 12.6) | 21.6 (-2.2 to 57.1) | 22.2 (-13.7 to 63.1) | 21.8 (-3.3 to 74.9) |
|  |  |  | All ages | 4.8 (-0.7 to 12.3) | 2 (-0.3 to 5.1) | 2.8 (-0.4 to 7.2) | 18.6 (-2.6 to 48.2) | 9.3 (-1.3 to 23.4) | 9.3 (-1.3 to 23.7) | 284.8 (212.3 to 386.8) | 359.7 (221.6 to 509.5) | 231.1 (167.3 to 349.5) |
|  |  | Deaths | Age-standardized | 0.2 (0 to 0.4) | 0.2 (0 to 0.4) | 0.1 (0 to 0.4) | 0.2 (0 to 0.6) | 0.3 (0 to 0.6) | 0.2 (0 to 0.6) | 50.4 (4.8 to 135.8) | 51.9 (-9.8 to 123.4) | 44.7 (-10.4 to 207.1) |
|  |  |  | All ages | 0.2 (0 to 0.4) | 0.1 (0 to 0.2) | 0.1 (0 to 0.2) | 0.7 (-0.1 to 1.9) | 0.4 (-0.1 to 1) | 0.3 (-0.1 to 0.8) | 380.9 (235.8 to 649.9) | 461 (231.6 to 729.8) | 305 (161.7 to 743.3) |
|  | Smoking | DALYs (Disability-Adjusted Life Years) | Age-standardized | 1.6 (0.9 to 2.5) | 0.5 (0.2 to 0.8) | 2.5 (1.3 to 4) | 2 (1.1 to 3.2) | 0.7 (0.3 to 1.2) | 3.4 (1.9 to 5.6) | 23.3 (-3.8 to 65.4) | 38.4 (-21.7 to 134.3) | 35.8 (3.1 to 89.6) |
|  |  |  | All ages | 2.6 (1.3 to 4.1) | 0.3 (0.2 to 0.6) | 2.2 (1.2 to 3.5) | 9.9 (5.5 to 15.6) | 1.7 (0.8 to 2.8) | 8.2 (4.5 to 12.9) | 285.5 (201.9 to 414.7) | 407.2 (188.5 to 740.9) | 267 (180.4 to 403) |
|  |  | Deaths | Age-standardized | 0 (0 to 0.1) | 0 (0 to 0) | 0.1 (0 to 0.1) | 0.1 (0 to 0.1) | 0 (0 to 0) | 0.1 (0 to 0.2) | 41.2 (-11.7 to 183.2) | 59.8 (-30.7 to 288.2) | 52.2 (-7.4 to 229.2) |
|  |  |  | All ages | 0 (0 to 0.1) | 0 (0 to 0) | 0 (0 to 0.1) | 0.2 (0.1 to 0.3) | 0 (0 to 0.1) | 0.2 (0.1 to 0.3) | 338.6 (190 to 739.9) | 481.5 (162.4 to 1128.1) | 311 (154.4 to 760.5) |
| Isfahan | All risk factors | DALYs (Disability-Adjusted Life Years) | Age-standardized | 23.7 (10.3 to 38.1) | 27.3 (11.9 to 43.9) | 19.3 (8 to 31.4) | 23.8 (13.1 to 35.7) | 29.6 (15.7 to 46) | 19 (9.9 to 29.7) | 0.6 (-22.1 to 38.7) | 8.5 (-23.6 to 58) | -1.4 (-25.1 to 44.3) |
|  |  |  | All ages | 289.8 (130.9 to 461.3) | 163.3 (71.8 to 262.3) | 126.5 (55.8 to 206.5) | 1140.1 (630.3 to 1723.5) | 651.8 (347.6 to 1009.8) | 488.3 (256.9 to 758.1) | 293.4 (210.7 to 428.7) | 299.2 (189.5 to 467.3) | 285.9 (191.8 to 452.1) |
|  |  | Deaths | Age-standardized | 1 (0.4 to 1.8) | 1.4 (0.6 to 2.4) | 0.6 (0.2 to 1.1) | 1 (0.5 to 1.6) | 1.6 (0.7 to 2.6) | 0.5 (0.3 to 0.9) | -3.7 (-35.7 to 50.4) | 12.4 (-31.5 to 83.6) | -10.3 (-44 to 72.3) |
|  |  |  | All ages | 8.9 (3.8 to 14.8) | 6.3 (2.8 to 10.8) | 2.6 (1 to 4.6) | 43.2 (21.7 to 66.5) | 30.4 (14.2 to 49) | 12.9 (6.1 to 21.4) | 385.3 (223.8 to 646.7) | 381.1 (196.7 to 660.6) | 395.5 (210.7 to 834.2) |
|  | Diet high in sodium | DALYs (Disability-Adjusted Life Years) | Age-standardized | 0.4 (0 to 2.1) | 0.3 (0 to 1.9) | 0.5 (0 to 2.6) | 0.4 (0 to 2.2) | 0.3 (0 to 2) | 0.5 (0 to 2.5) | 0.3 (-94.8 to 2721.4) | 2.6 (-272179.9 to 17386.3) | -3.3 (-94.6 to 3413.2) |
|  |  |  | All ages | 5.2 (0 to 28.7) | 1.7 (0 to 12.3) | 3.4 (0 to 17.5) | 17.6 (0 to 107.1) | 5.9 (0 to 44.8) | 11.7 (0 to 62.4) | 241.1 (-84.2 to 7590.6) | 243.6 (-3478715.2 to 31229.4) | 239.8 (-84.4 to 7692.8) |
|  |  | Deaths | Age-standardized | 0 (0 to 0.1) | 0 (0 to 0.1) | 0 (0 to 0.1) | 0 (0 to 0.1) | 0 (0 to 0.1) | 0 (0 to 0.1) | 0.4 (-96.3 to 3565.5) | 11.5 (-63008.1 to 21620.1) | -8.5 (-96.5 to 3859.6) |
|  |  |  | All ages | 0.1 (0 to 0.7) | 0.1 (0 to 0.4) | 0.1 (0 to 0.3) | 0.5 (0 to 3.6) | 0.2 (0 to 1.9) | 0.3 (0 to 1.6) | 339.6 (-87.5 to 9404.6) | 334.7 (-406344.3 to 46051.4) | 344 (-87.5 to 11137.3) |
|  | High alcohol use | DALYs (Disability-Adjusted Life Years) | Age-standardized | 0 (0 to 0) | 0 (0 to 0) | 0 (0 to 0) | 0.2 (0.1 to 0.3) | 0 (0 to 0.1) | 0.3 (0.2 to 0.5) | NA | NA | NA |
|  |  |  | All ages | 0 (0 to 0) | 0 (0 to 0) | 0 (0 to 0) | 9.8 (5.6 to 15.3) | 0.8 (0.3 to 1.5) | 9 (5.2 to 13.9) | NA | NA | NA |
|  |  | Deaths | Age-standardized | 0 (0 to 0) | 0 (0 to 0) | 0 (0 to 0) | 0 (0 to 0) | 0 (0 to 0) | 0 (0 to 0) | NA | NA | NA |
|  |  |  | All ages | 0 (0 to 0) | 0 (0 to 0) | 0 (0 to 0) | 0.2 (0.1 to 0.4) | 0 (0 to 0.1) | 0.2 (0.1 to 0.3) | NA | NA | NA |
|  | High body-mass index | DALYs (Disability-Adjusted Life Years) | Age-standardized | 3.5 (1.3 to 6.3) | 4.9 (1.8 to 9.2) | 1.8 (0.7 to 3.4) | 10.1 (4.1 to 17.7) | 13.9 (5.8 to 24.4) | 6.9 (2.6 to 12.1) | 193.3 (62.4 to 402.5) | 181.1 (42.2 to 394.5) | 285.6 (113.2 to 769.9) |
|  |  |  | All ages | 48.2 (18.2 to 84.8) | 34 (13 to 60.9) | 14.3 (5.3 to 27.6) | 484.2 (194.6 to 846.7) | 307.9 (129 to 541.4) | 176.3 (66.5 to 306.7) | 904.1 (492.6 to 1415.3) | 806.4 (402.3 to 1324.2) | 1136.9 (585.5 to 2227.6) |
|  |  | Deaths | Age-standardized | 0.1 (0 to 0.3) | 0.2 (0.1 to 0.5) | 0 (0 to 0.1) | 0.4 (0.2 to 0.8) | 0.7 (0.3 to 1.3) | 0.2 (0.1 to 0.4) | 223.2 (44.2 to 698) | 240.4 (33.3 to 769) | 368.1 (92.6 to 1767.2) |
|  |  |  | All ages | 1.3 (0.5 to 2.5) | 1.1 (0.4 to 2.1) | 0.2 (0.1 to 0.5) | 18.9 (7.6 to 34) | 13.9 (5.5 to 25.2) | 5 (1.8 to 9.1) | 1357 (604 to 2688.1) | 1209 (469.8 to 2590.4) | 2036.1 (878.4 to 5548.4) |
|  | High systolic blood pressure | DALYs (Disability-Adjusted Life Years) | Age-standardized | 20.9 (6.6 to 35.1) | 24.5 (7.8 to 42.1) | 16.4 (5 to 28.8) | 16.9 (5.6 to 29.9) | 21.7 (7.4 to 38.2) | 12.8 (3.9 to 23.9) | -18.9 (-39 to 1.7) | -11.5 (-39.2 to 20.7) | -21.9 (-42.4 to 5.4) |
|  |  |  | All ages | 246 (75.1 to 418.2) | 143.8 (45.7 to 243.8) | 102.2 (31.3 to 180.8) | 801.5 (263.2 to 1406.9) | 476.2 (161.1 to 833.3) | 325.3 (98.2 to 600.6) | 225.8 (149.8 to 305) | 231.1 (133.1 to 345.8) | 218.3 (130.9 to 324.7) |
|  |  | Deaths | Age-standardized | 1 (0.3 to 1.7) | 1.3 (0.5 to 2.3) | 0.5 (0.2 to 1) | 0.7 (0.3 to 1.3) | 1.2 (0.4 to 2.1) | 0.4 (0.1 to 0.7) | -23.6 (-50.1 to 9) | -9.8 (-47.8 to 36.2) | -31.1 (-57.8 to 25.5) |
|  |  |  | All ages | 8 (2.8 to 13.8) | 5.7 (2 to 10.5) | 2.2 (0.7 to 4.2) | 31.3 (11.5 to 54.3) | 22.5 (7.6 to 41) | 8.8 (2.9 to 17.1) | 292 (155.6 to 461.3) | 291.9 (129 to 493.2) | 292.3 (136.4 to 609.8) |
|  | Lead exposure | DALYs (Disability-Adjusted Life Years) | Age-standardized | 3.3 (-0.5 to 8.1) | 3.4 (-0.5 to 8.5) | 3.1 (-0.5 to 7.9) | 3.7 (-0.5 to 9.3) | 4 (-0.6 to 10.1) | 3.4 (-0.5 to 8.7) | 11.8 (-9.5 to 37) | 18.7 (-13.3 to 53.2) | 9.9 (-13.1 to 42.3) |
|  |  |  | All ages | 40.2 (-5.6 to 101) | 20.4 (-2.8 to 50.8) | 19.8 (-2.8 to 51.5) | 167.8 (-23.5 to 428.5) | 84.4 (-12.2 to 212.3) | 83.4 (-11 to 213.8) | 317 (242.8 to 407.6) | 314 (203.3 to 445.9) | 320.1 (234.9 to 439.4) |
|  |  | Deaths | Age-standardized | 0.1 (0 to 0.4) | 0.2 (0 to 0.5) | 0.1 (0 to 0.3) | 0.2 (0 to 0.4) | 0.2 (0 to 0.6) | 0.1 (0 to 0.3) | 19.8 (-15 to 64.5) | 38.3 (-10.9 to 98.9) | 12.6 (-24.2 to 93.6) |
|  |  |  | All ages | 1.2 (-0.2 to 3.1) | 0.8 (-0.1 to 2) | 0.4 (-0.1 to 1.2) | 7.2 (-1.1 to 18) | 4.6 (-0.7 to 11.7) | 2.6 (-0.4 to 6.7) | 492.9 (313.7 to 708.8) | 476.2 (265.3 to 733.6) | 523.9 (319 to 957.3) |
|  | Smoking | DALYs (Disability-Adjusted Life Years) | Age-standardized | 1.7 (0.9 to 2.7) | 0.6 (0.3 to 1) | 2.9 (1.6 to 4.5) | 1.9 (1 to 2.9) | 0.6 (0.3 to 1.1) | 3.1 (1.7 to 4.8) | 11 (-12.8 to 43.1) | 8.9 (-36.5 to 78.7) | 7.5 (-17.4 to 43.4) |
|  |  |  | All ages | 29.5 (16.2 to 47.3) | 4.9 (2.5 to 8.1) | 24.6 (13.3 to 39.5) | 102.6 (56.2 to 158.4) | 16.9 (8 to 28.4) | 85.7 (46.9 to 133.7) | 247.7 (169.8 to 337) | 245.8 (103.9 to 445.9) | 248.1 (162.2 to 366) |
|  |  | Deaths | Age-standardized | 0 (0 to 0.1) | 0 (0 to 0) | 0.1 (0 to 0.1) | 0 (0 to 0.1) | 0 (0 to 0) | 0.1 (0 to 0.1) | 14.1 (-26.5 to 99.7) | 17.6 (-51.6 to 187.8) | 3.4 (-39.3 to 91.8) |
|  |  |  | All ages | 0.5 (0.2 to 0.7) | 0.1 (0 to 0.2) | 0.3 (0.2 to 0.6) | 2 (1 to 3.3) | 0.5 (0.2 to 1) | 1.6 (0.8 to 2.5) | 351.5 (194 to 640.1) | 333.8 (106.8 to 789.2) | 357.2 (174.4 to 694.5) |
| Kerman | All risk factors | DALYs (Disability-Adjusted Life Years) | Age-standardized | 25.6 (11.3 to 39.9) | 28.6 (12.4 to 46.8) | 21.5 (9.3 to 35.7) | 33.4 (17.6 to 50.9) | 40.3 (20.8 to 61.4) | 26.5 (13.8 to 40.6) | 30.6 (1.1 to 81.3) | 40.6 (-3.3 to 111.1) | 23.1 (-5.5 to 78.3) |
|  |  |  | All ages | 147 (66 to 227.8) | 79.2 (34.5 to 128.6) | 67.9 (31.1 to 108.9) | 678 (359.9 to 1024.2) | 395.2 (204.7 to 604.7) | 282.8 (145.2 to 426.5) | 361.1 (267 to 517) | 399.3 (254.3 to 629.7) | 316.7 (224.2 to 480.4) |
|  |  | Deaths | Age-standardized | 1.2 (0.5 to 2) | 1.5 (0.6 to 2.6) | 0.8 (0.3 to 1.4) | 1.6 (0.8 to 2.6) | 2.3 (1.1 to 3.7) | 1 (0.4 to 1.6) | 38.7 (-6.2 to 114.3) | 56.7 (-4 to 164) | 24.9 (-15.6 to 139.5) |
|  |  |  | All ages | 4.5 (2 to 7.3) | 3 (1.2 to 5.1) | 1.5 (0.5 to 2.6) | 27.1 (13.1 to 42.5) | 18.6 (8.6 to 30) | 8.4 (3.9 to 13.4) | 501.2 (307.9 to 817.8) | 524.2 (278 to 930.3) | 455.9 (257.8 to 930.3) |
|  | Diet high in sodium | DALYs (Disability-Adjusted Life Years) | Age-standardized | 0.4 (0 to 2.5) | 0.3 (0 to 2) | 0.6 (0 to 2.9) | 0.4 (0 to 2.8) | 0.3 (0 to 2.3) | 0.6 (0 to 3.2) | 2.7 (-95.8 to 3789.1) | 3.2 (-445.1 to 438644.6) | 1.3 (-95.7 to 4036.8) |
|  |  |  | All ages | 2.8 (0 to 15.5) | 0.9 (0 to 6.1) | 1.9 (0 to 9.5) | 9.2 (0 to 57.9) | 3.1 (0 to 23.4) | 6.1 (0 to 33.5) | 229.8 (-87.8 to 12421.4) | 245.8 (-933.6 to 994512.4) | 222.3 (-87.8 to 12661.5) |
|  |  | Deaths | Age-standardized | 0 (0 to 0.1) | 0 (0 to 0.1) | 0 (0 to 0.1) | 0 (0 to 0.1) | 0 (0 to 0.1) | 0 (0 to 0.1) | 8.6 (-97.7 to 3490) | 14.4 (-793 to 155418.2) | 0 (-97.6 to 4322.5) |
|  |  |  | All ages | 0.1 (0 to 0.4) | 0 (0 to 0.2) | 0 (0 to 0.2) | 0.3 (0 to 2) | 0.1 (0 to 1) | 0.2 (0 to 1.1) | 308.7 (-91.4 to 10855.2) | 317.7 (-2006.2 to 689677.4) | 301.9 (-91.4 to 12198.7) |
|  | High alcohol use | DALYs (Disability-Adjusted Life Years) | Age-standardized | 0 (0 to 0) | 0 (0 to 0) | 0 (0 to 0) | 0.2 (0.1 to 0.3) | 0 (0 to 0.1) | 0.4 (0.2 to 0.6) | NA | NA | NA |
|  |  |  | All ages | 0 (0 to 0) | 0 (0 to 0) | 0 (0 to 0) | 4.6 (2.4 to 7.3) | 0.4 (0.1 to 0.7) | 4.3 (2.3 to 6.7) | NA | NA | NA |
|  |  | Deaths | Age-standardized | 0 (0 to 0) | 0 (0 to 0) | 0 (0 to 0) | 0 (0 to 0) | 0 (0 to 0) | 0 (0 to 0) | NA | NA | NA |
|  |  |  | All ages | 0 (0 to 0) | 0 (0 to 0) | 0 (0 to 0) | 0.1 (0 to 0.2) | 0 (0 to 0) | 0.1 (0 to 0.2) | NA | NA | NA |
|  | High body-mass index | DALYs (Disability-Adjusted Life Years) | Age-standardized | 3.7 (1.5 to 6.9) | 5.8 (2.3 to 10.5) | 1.4 (0.4 to 2.9) | 12.3 (5 to 21.5) | 16.2 (6.5 to 27.9) | 8.3 (3.3 to 15.2) | 227.7 (83.2 to 439.7) | 180.3 (45.3 to 389.7) | 479.2 (184.2 to 1352.5) |
|  |  |  | All ages | 23.9 (9.5 to 40.1) | 18.1 (7.3 to 31.8) | 5.8 (2 to 11.4) | 248.6 (102 to 435.2) | 161.5 (66.4 to 284.1) | 87.1 (34.9 to 160.8) | 940.3 (533.5 to 1387.4) | 790.1 (418.3 to 1253.5) | 1413.5 (742.3 to 3258.6) |
|  |  | Deaths | Age-standardized | 0.2 (0.1 to 0.3) | 0.3 (0.1 to 0.6) | 0 (0 to 0.1) | 0.6 (0.3 to 1.1) | 0.9 (0.4 to 1.7) | 0.3 (0.1 to 0.6) | 270.7 (72.6 to 792.3) | 246.8 (48.7 to 784.6) | 715.9 (180.7 to 3749.6) |
|  |  |  | All ages | 0.7 (0.3 to 1.3) | 0.6 (0.2 to 1.1) | 0.1 (0 to 0.2) | 9.9 (4.1 to 17.8) | 7.2 (3 to 13.2) | 2.7 (1 to 5.1) | 1327.7 (649.3 to 2427.9) | 1125 (481 to 2339.4) | 2474 (1035.2 to 6859.2) |
|  | High systolic blood pressure | DALYs (Disability-Adjusted Life Years) | Age-standardized | 22.4 (7.7 to 37.4) | 25.4 (8.4 to 44.3) | 18.6 (5.7 to 32.8) | 25.8 (9.2 to 44.6) | 31.9 (11.8 to 54.8) | 19.7 (6.6 to 34.4) | 15.1 (-9.6 to 50.9) | 25.9 (-11.3 to 85.4) | 5.8 (-20.3 to 42.7) |
|  |  |  | All ages | 125.2 (41.7 to 206.3) | 68.7 (22 to 119.6) | 56.5 (18 to 98.8) | 518.7 (184.2 to 891) | 312 (113.5 to 535.2) | 206.7 (68.2 to 359.5) | 314.4 (232 to 432.6) | 354.2 (224.9 to 566.1) | 266 (177 to 382.6) |
|  |  | Deaths | Age-standardized | 1.1 (0.3 to 1.9) | 1.3 (0.4 to 2.4) | 0.7 (0.2 to 1.3) | 1.3 (0.5 to 2.2) | 1.8 (0.7 to 3.3) | 0.7 (0.2 to 1.3) | 20.6 (-17.8 to 83.9) | 38.3 (-15.5 to 131.2) | 5 (-30.6 to 80.5) |
|  |  |  | All ages | 4 (1.2 to 6.8) | 2.7 (0.8 to 4.9) | 1.3 (0.4 to 2.4) | 21.3 (8 to 37.1) | 15 (5.6 to 26.4) | 6.4 (2.1 to 11.5) | 436.9 (265.8 to 698.8) | 462.9 (237.8 to 836.1) | 384.3 (203.4 to 734.1) |
|  | Lead exposure | DALYs (Disability-Adjusted Life Years) | Age-standardized | 4.4 (-0.6 to 11.1) | 4.5 (-0.6 to 11.4) | 4.2 (-0.6 to 10.7) | 4.7 (-0.7 to 11.6) | 4.9 (-0.7 to 12) | 4.6 (-0.7 to 11.2) | 8.7 (-13.7 to 38.9) | 10.3 (-20.1 to 53.2) | 9.2 (-15.9 to 44.2) |
|  |  |  | All ages | 24 (-3.3 to 61) | 11.9 (-1.6 to 30.8) | 12.1 (-1.7 to 30.3) | 90.5 (-13 to 224.8) | 45.1 (-6.4 to 112) | 45.5 (-6.7 to 109.8) | 277.7 (200.1 to 373.3) | 279.5 (174.9 to 414.3) | 276 (195.9 to 386.1) |
|  |  | Deaths | Age-standardized | 0.2 (0 to 0.5) | 0.2 (0 to 0.6) | 0.2 (0 to 0.4) | 0.3 (0 to 0.6) | 0.3 (-0.1 to 0.8) | 0.2 (0 to 0.5) | 21.3 (-12.5 to 77.8) | 31.4 (-15.6 to 101.3) | 15.9 (-18.3 to 96.4) |
|  |  |  | All ages | 0.8 (-0.1 to 2) | 0.5 (-0.1 to 1.2) | 0.3 (0 to 0.8) | 4 (-0.7 to 10) | 2.4 (-0.4 to 6.2) | 1.6 (-0.3 to 4) | 424.3 (274.4 to 662.5) | 412.4 (234.7 to 683.6) | 443.3 (263.7 to 811.8) |
|  | Smoking | DALYs (Disability-Adjusted Life Years) | Age-standardized | 1.8 (1 to 2.9) | 0.7 (0.3 to 1.1) | 3.1 (1.6 to 4.9) | 2.1 (1.2 to 3.3) | 0.7 (0.4 to 1.3) | 3.5 (1.9 to 5.6) | 13.4 (-13.5 to 49.1) | 10.4 (-37.9 to 91.8) | 13 (-16.1 to 53.6) |
|  |  |  | All ages | 14.9 (8.4 to 23.7) | 2.5 (1.3 to 4.2) | 12.4 (6.5 to 19.6) | 52.9 (29.9 to 83.9) | 8.9 (4.4 to 15.3) | 44 (24.9 to 69.5) | 255.7 (170.6 to 360.9) | 259.8 (114.1 to 515) | 254.9 (160.6 to 375) |
|  |  | Deaths | Age-standardized | 0 (0 to 0.1) | 0 (0 to 0) | 0.1 (0 to 0.1) | 0.1 (0 to 0.1) | 0 (0 to 0.1) | 0.1 (0 to 0.1) | 20.3 (-22.9 to 106.7) | 22.4 (-50.3 to 203.7) | 10.7 (-32.5 to 103.2) |
|  |  |  | All ages | 0.3 (0.1 to 0.4) | 0.1 (0 to 0.1) | 0.2 (0.1 to 0.4) | 1.1 (0.6 to 1.9) | 0.3 (0.1 to 0.5) | 0.9 (0.4 to 1.4) | 312.1 (155.6 to 570.4) | 319.8 (99.9 to 777) | 309.8 (138.1 to 634.5) |
| Kermanshah | All risk factors | DALYs (Disability-Adjusted Life Years) | Age-standardized | 21.9 (9.4 to 36.3) | 25.5 (10.9 to 43.1) | 18.6 (7.4 to 31.4) | 29.9 (15.1 to 44.9) | 36.3 (17.5 to 56.9) | 24.5 (12.3 to 38.3) | 36.5 (4.3 to 86.2) | 42.3 (-5.4 to 112) | 32 (-0.7 to 94) |
|  |  |  | All ages | 109.8 (48.6 to 175.7) | 54.8 (23.9 to 91.2) | 55.1 (23.5 to 92.9) | 521.9 (267.9 to 776.6) | 301.7 (145.5 to 468) | 220.2 (110.4 to 344.7) | 375.2 (278.3 to 517.9) | 450.9 (282.8 to 689.4) | 300 (200.8 to 469.7) |
|  |  | Deaths | Age-standardized | 1 (0.4 to 1.7) | 1.3 (0.5 to 2.5) | 0.7 (0.2 to 1.2) | 1.3 (0.7 to 2) | 2 (0.9 to 3.1) | 0.8 (0.4 to 1.2) | 31.1 (-13 to 100.2) | 46.6 (-14.7 to 146.2) | 16.5 (-26.5 to 112.7) |
|  |  |  | All ages | 3.6 (1.5 to 6) | 2.2 (0.9 to 4) | 1.4 (0.5 to 2.4) | 20.8 (10.5 to 31.7) | 14.2 (6.7 to 21.9) | 6.6 (3.1 to 10.6) | 478.4 (297.3 to 744.1) | 533.5 (273 to 950) | 387.7 (207.5 to 742.3) |
|  | Diet high in sodium | DALYs (Disability-Adjusted Life Years) | Age-standardized | 0.4 (0 to 2.5) | 0.3 (0 to 2.3) | 0.5 (0 to 2.8) | 0.4 (0 to 2.6) | 0.3 (0 to 2.2) | 0.5 (0 to 2.9) | -2.4 (-92.6 to 2594.9) | 1.2 (-564.6 to 66841.3) | 0.3 (-92.3 to 2821.9) |
|  |  |  | All ages | 2.4 (0 to 13.7) | 0.7 (0 to 5.5) | 1.7 (0 to 8.5) | 7.3 (0 to 45.1) | 2.6 (0 to 19.1) | 4.7 (0 to 25.6) | 205.2 (-79.3 to 5140.9) | 270 (-1207.4 to 841669.9) | 178.4 (-80.8 to 5140.9) |
|  |  | Deaths | Age-standardized | 0 (0 to 0.1) | 0 (0 to 0.1) | 0 (0 to 0.1) | 0 (0 to 0.1) | 0 (0 to 0.1) | 0 (0 to 0.1) | -5 (-95.9 to 3041.3) | 5.4 (-782.5 to 42420.7) | -11.4 (-95.3 to 3343.5) |
|  |  |  | All ages | 0.1 (0 to 0.4) | 0 (0 to 0.2) | 0 (0 to 0.2) | 0.2 (0 to 1.6) | 0.1 (0 to 0.8) | 0.1 (0 to 0.8) | 264.9 (-84.6 to 6510) | 325.3 (-1044.1 to 104759.4) | 226.1 (-86.7 to 6510) |
|  | High alcohol use | DALYs (Disability-Adjusted Life Years) | Age-standardized | 0 (0 to 0) | 0 (0 to 0) | 0 (0 to 0) | 0.2 (0.1 to 0.3) | 0 (0 to 0.1) | 0.3 (0.2 to 0.5) | NA | NA | NA |
|  |  |  | All ages | 0 (0 to 0) | 0 (0 to 0) | 0 (0 to 0) | 3.6 (1.8 to 5.5) | 0.3 (0.1 to 0.6) | 3.3 (1.7 to 5.1) | NA | NA | NA |
|  |  | Deaths | Age-standardized | 0 (0 to 0) | 0 (0 to 0) | 0 (0 to 0) | 0 (0 to 0) | 0 (0 to 0) | 0 (0 to 0) | NA | NA | NA |
|  |  |  | All ages | 0 (0 to 0) | 0 (0 to 0) | 0 (0 to 0) | 0.1 (0 to 0.1) | 0 (0 to 0) | 0.1 (0 to 0.1) | NA | NA | NA |
|  | High body-mass index | DALYs (Disability-Adjusted Life Years) | Age-standardized | 3.1 (1.1 to 5.5) | 5.2 (1.9 to 9.9) | 1.2 (0.4 to 2.5) | 10.4 (4.3 to 18.1) | 13.5 (5.7 to 24) | 7.6 (3 to 12.9) | 236.1 (84.3 to 502.3) | 158 (33.1 to 383.9) | 510.2 (221 to 1580.8) |
|  |  |  | All ages | 18.2 (6.6 to 31.1) | 13.5 (4.8 to 24) | 4.7 (1.6 to 8.9) | 184.5 (77.1 to 320.9) | 115.7 (49.3 to 205.1) | 68.7 (26.8 to 117.8) | 912 (533.7 to 1434.1) | 757.2 (383.3 to 1242.7) | 1353.9 (714.1 to 3389.4) |
|  |  | Deaths | Age-standardized | 0.1 (0 to 0.3) | 0.2 (0.1 to 0.5) | 0 (0 to 0.1) | 0.5 (0.2 to 0.8) | 0.7 (0.3 to 1.3) | 0.3 (0.1 to 0.4) | 250.8 (52.5 to 830.8) | 203.3 (22.4 to 751.6) | 649.3 (168.6 to 3281.2) |
|  |  |  | All ages | 0.5 (0.2 to 1) | 0.4 (0.2 to 0.9) | 0.1 (0 to 0.2) | 7.2 (2.9 to 12.9) | 5.1 (2 to 9.4) | 2.1 (0.8 to 3.9) | 1247.6 (589.5 to 2576) | 1041 (418.7 to 2279.3) | 2257.2 (958.4 to 7346.2) |
|  | High systolic blood pressure | DALYs (Disability-Adjusted Life Years) | Age-standardized | 18.9 (6.5 to 33.3) | 22.3 (7.7 to 39.8) | 15.7 (4.7 to 28.8) | 23.3 (7.6 to 38.5) | 29.2 (9.2 to 49.6) | 18.2 (6.1 to 32.4) | 23.4 (-5.6 to 58.9) | 30.9 (-15 to 90.7) | 16.1 (-15.6 to 67.5) |
|  |  |  | All ages | 90.2 (29.3 to 158.2) | 46.1 (15.1 to 83.4) | 44.1 (13.2 to 81.4) | 401.6 (130.3 to 668.5) | 240.2 (74.7 to 409) | 161.4 (54.9 to 291.3) | 345.2 (245.5 to 476.9) | 421 (247.8 to 649.9) | 265.8 (169.2 to 418.4) |
|  |  | Deaths | Age-standardized | 0.9 (0.3 to 1.6) | 1.2 (0.4 to 2.3) | 0.6 (0.2 to 1.1) | 1.1 (0.3 to 1.7) | 1.6 (0.5 to 2.8) | 0.6 (0.2 to 1.1) | 17 (-21.8 to 69.7) | 33.1 (-23.5 to 127) | -0.1 (-37.1 to 76.2) |
|  |  |  | All ages | 3.1 (1.1 to 5.6) | 2 (0.7 to 3.8) | 1.2 (0.3 to 2.2) | 16.6 (5.5 to 27.4) | 11.6 (3.7 to 19.6) | 5 (1.7 to 9.4) | 430 (257.6 to 656.4) | 487.3 (243.2 to 862.1) | 332.6 (165.3 to 661) |
|  | Lead exposure | DALYs (Disability-Adjusted Life Years) | Age-standardized | 4.4 (-0.6 to 10.9) | 4.8 (-0.6 to 12.4) | 4.1 (-0.6 to 10.2) | 4.6 (-0.6 to 11.4) | 5.1 (-0.7 to 12.7) | 4.3 (-0.6 to 10.6) | 4.3 (-16.9 to 27.5) | 6.7 (-24.3 to 42.9) | 5.2 (-16.9 to 36.5) |
|  |  |  | All ages | 21.7 (-2.9 to 53.6) | 10.2 (-1.3 to 26.6) | 11.5 (-1.6 to 28.5) | 78.1 (-10.8 to 195.5) | 40.4 (-5.8 to 100.8) | 37.7 (-5 to 93.2) | 259.2 (188.8 to 337.5) | 296.1 (179.3 to 426.3) | 226.6 (156.5 to 323.4) |
|  |  | Deaths | Age-standardized | 0.2 (0 to 0.5) | 0.3 (0 to 0.7) | 0.2 (0 to 0.4) | 0.2 (0 to 0.5) | 0.3 (0 to 0.8) | 0.2 (0 to 0.4) | 6.5 (-23.1 to 43.6) | 18.7 (-25.7 to 75.3) | -1.4 (-33.7 to 58) |
|  |  |  | All ages | 0.7 (-0.1 to 1.8) | 0.4 (-0.1 to 1.1) | 0.3 (0 to 0.8) | 3.4 (-0.5 to 8.4) | 2.1 (-0.3 to 5.3) | 1.3 (-0.2 to 3.3) | 370 (237.6 to 529.2) | 397.9 (213.6 to 629) | 331.4 (183.7 to 594) |
|  | Smoking | DALYs (Disability-Adjusted Life Years) | Age-standardized | 1.9 (1.1 to 3) | 0.7 (0.3 to 1.1) | 3 (1.6 to 4.8) | 2 (1.1 to 3.1) | 0.7 (0.4 to 1.3) | 3.3 (1.8 to 5.2) | 4.2 (-22.3 to 33.5) | 6.2 (-40.8 to 88.6) | 11 (-18.7 to 48.9) |
|  |  |  | All ages | 13.6 (7.5 to 21.4) | 2.1 (1.1 to 3.5) | 11.4 (6 to 18.3) | 40 (21.4 to 61.8) | 7.1 (3.6 to 12.5) | 32.9 (17.8 to 50.7) | 194.8 (118.5 to 279.2) | 236.1 (82.3 to 480.8) | 187.1 (110.2 to 280.5) |
|  |  | Deaths | Age-standardized | 0.1 (0 to 0.1) | 0 (0 to 0) | 0.1 (0 to 0.1) | 0.1 (0 to 0.1) | 0 (0 to 0.1) | 0.1 (0 to 0.1) | -0.5 (-40.5 to 54.4) | 8.6 (-56.3 to 155.6) | -2.7 (-43.9 to 62.4) |
|  |  |  | All ages | 0.3 (0.1 to 0.4) | 0.1 (0 to 0.1) | 0.2 (0.1 to 0.4) | 0.9 (0.4 to 1.4) | 0.2 (0.1 to 0.4) | 0.7 (0.3 to 1.1) | 236.2 (99.1 to 407.3) | 286.2 (64.3 to 743.8) | 223.4 (78.8 to 433.5) |
| Khorasan-e-Razavi | All risk factors | DALYs (Disability-Adjusted Life Years) | Age-standardized | 24.5 (10.3 to 40.6) | 27.4 (11.6 to 47.4) | 21.4 (8.7 to 35.9) | 29.2 (14.9 to 44.7) | 33 (16.7 to 52.1) | 25.2 (12.6 to 39.5) | 19 (-6.9 to 67) | 20.7 (-16.6 to 81.2) | 17.9 (-7.6 to 65.5) |
|  |  |  | All ages | 351.5 (155.3 to 565.3) | 179.3 (77.5 to 298.4) | 172.2 (73.9 to 284.7) | 1385.7 (722.3 to 2122.8) | 778.1 (401 to 1219.9) | 607.6 (312.8 to 942.1) | 294.2 (211.5 to 431.8) | 333.9 (206.5 to 545.8) | 252.8 (173.5 to 385.9) |
|  |  | Deaths | Age-standardized | 1.2 (0.5 to 2) | 1.5 (0.6 to 2.6) | 0.8 (0.3 to 1.4) | 1.3 (0.6 to 2.2) | 1.8 (0.8 to 3) | 0.9 (0.4 to 1.4) | 15.6 (-21.3 to 85.7) | 21.3 (-28 to 105) | 8.2 (-26.2 to 95.4) |
|  |  |  | All ages | 10.6 (4.4 to 17.5) | 6.7 (2.8 to 11.5) | 3.9 (1.5 to 6.6) | 55.3 (26.2 to 89.6) | 37.2 (17.4 to 61) | 18 (8.3 to 29.1) | 420.2 (262.4 to 696.3) | 457.7 (238.3 to 829.7) | 356.8 (208 to 703.6) |
|  | Diet high in sodium | DALYs (Disability-Adjusted Life Years) | Age-standardized | 0.4 (0 to 2.5) | 0.3 (0 to 2.2) | 0.5 (0 to 2.8) | 0.4 (0 to 2.5) | 0.3 (0 to 2.2) | 0.5 (0 to 3) | -2.3 (-95.7 to 2314.7) | -1.3 (-558.1 to 63000.5) | -1.3 (-95.7 to 2491) |
|  |  |  | All ages | 6.9 (0 to 39.6) | 2.2 (0 to 15.8) | 4.7 (0 to 23.8) | 20 (0 to 119.6) | 7.1 (0 to 53) | 12.9 (0 to 70.8) | 188.3 (-87.3 to 6183.6) | 220.6 (-2098.7 to 482759.8) | 173.1 (-87.3 to 6197) |
|  |  | Deaths | Age-standardized | 0 (0 to 0.1) | 0 (0 to 0.1) | 0 (0 to 0.1) | 0 (0 to 0.1) | 0 (0 to 0.1) | 0 (0 to 0.1) | -5.5 (-98 to 4233.9) | -0.5 (-535.6 to 97114.8) | -10.2 (-97.8 to 4323.1) |
|  |  |  | All ages | 0.2 (0 to 1.1) | 0.1 (0 to 0.6) | 0.1 (0 to 0.5) | 0.6 (0 to 4.3) | 0.3 (0 to 2.2) | 0.3 (0 to 2.1) | 263.6 (-92.9 to 8346) | 296.8 (-1378.6 to 475214.8) | 239.4 (-92.9 to 8938.6) |
|  | High alcohol use | DALYs (Disability-Adjusted Life Years) | Age-standardized | 0 (0 to 0) | 0 (0 to 0) | 0 (0 to 0) | 0.2 (0.1 to 0.3) | 0 (0 to 0.1) | 0.3 (0.2 to 0.5) | NA | NA | NA |
|  |  |  | All ages | 0 (0 to 0) | 0 (0 to 0) | 0 (0 to 0) | 9.8 (5.3 to 15.4) | 0.8 (0.3 to 1.6) | 9 (4.9 to 14.1) | NA | NA | NA |
|  |  | Deaths | Age-standardized | 0 (0 to 0) | 0 (0 to 0) | 0 (0 to 0) | 0 (0 to 0) | 0 (0 to 0) | 0 (0 to 0) | NA | NA | NA |
|  |  |  | All ages | 0 (0 to 0) | 0 (0 to 0) | 0 (0 to 0) | 0.2 (0.1 to 0.4) | 0 (0 to 0.1) | 0.2 (0.1 to 0.3) | NA | NA | NA |
|  | High body-mass index | DALYs (Disability-Adjusted Life Years) | Age-standardized | 3.6 (1.4 to 6.7) | 5.8 (2.2 to 11) | 1.4 (0.4 to 2.9) | 10.4 (4.3 to 18.4) | 14 (5.7 to 25.1) | 6.7 (2.6 to 12.1) | 188.1 (60.2 to 374.3) | 140.1 (19.4 to 303.1) | 376.7 (158 to 1203.4) |
|  |  |  | All ages | 59.2 (23.5 to 99) | 44.8 (17.7 to 75.3) | 14.4 (4.8 to 28) | 494.6 (205.8 to 869.3) | 334.5 (138.3 to 594.4) | 160.1 (63.2 to 287.9) | 735 (428.4 to 1117.9) | 646.2 (329.1 to 1044.2) | 1011.1 (538.6 to 2488.3) |
|  |  | Deaths | Age-standardized | 0.2 (0.1 to 0.3) | 0.3 (0.1 to 0.6) | 0 (0 to 0.1) | 0.5 (0.2 to 0.9) | 0.7 (0.3 to 1.3) | 0.2 (0.1 to 0.5) | 204.7 (38.7 to 577.2) | 168.8 (10.3 to 512.8) | 494.2 (120.4 to 2818.4) |
|  |  |  | All ages | 1.7 (0.7 to 3.2) | 1.4 (0.6 to 2.7) | 0.3 (0.1 to 0.6) | 20 (8.1 to 35.8) | 15.2 (6 to 27.3) | 4.9 (1.7 to 9.4) | 1083.3 (535.5 to 2025.3) | 961.6 (386.8 to 1818.1) | 1741.6 (752.5 to 4971.4) |
|  | High systolic blood pressure | DALYs (Disability-Adjusted Life Years) | Age-standardized | 21.4 (6.7 to 37.6) | 23.9 (7.1 to 44.7) | 18.6 (5.6 to 33) | 22.5 (7.3 to 39.1) | 25.3 (7.3 to 46.6) | 19.6 (6.4 to 34.1) | 5.2 (-18 to 36.2) | 5.6 (-28.8 to 59.4) | 5.7 (-21 to 44.9) |
|  |  |  | All ages | 295.1 (96 to 509.4) | 152.1 (48.3 to 273.3) | 143 (44.2 to 252.5) | 1056.5 (346.4 to 1825.2) | 591.7 (179.7 to 1074.4) | 464.8 (154.7 to 807.9) | 258 (181.8 to 366.7) | 289.1 (167.2 to 481.5) | 225 (143.1 to 340.5) |
|  |  | Deaths | Age-standardized | 1 (0.3 to 1.9) | 1.3 (0.4 to 2.5) | 0.7 (0.2 to 1.3) | 1 (0.3 to 1.9) | 1.4 (0.4 to 2.6) | 0.7 (0.2 to 1.2) | 0.7 (-31.7 to 54.2) | 5.3 (-37.5 to 79.8) | -4.9 (-35.5 to 71.7) |
|  |  |  | All ages | 9.3 (2.8 to 16.3) | 5.8 (1.8 to 10.6) | 3.4 (1 to 6) | 43.2 (14.3 to 79.1) | 28.9 (8.5 to 53.5) | 14.3 (4.5 to 25.4) | 365.7 (218 to 585) | 395.4 (188.1 to 741.3) | 315.2 (172.4 to 615.6) |
|  | Lead exposure | DALYs (Disability-Adjusted Life Years) | Age-standardized | 4.7 (-0.7 to 11.9) | 5 (-0.8 to 12.9) | 4.3 (-0.6 to 10.8) | 4.8 (-0.6 to 12) | 5.2 (-0.7 to 13.7) | 4.4 (-0.7 to 10.7) | 3.3 (-19.2 to 31.9) | 3.8 (-27.7 to 43.3) | 3.3 (-17.5 to 42.3) |
|  |  |  | All ages | 62.9 (-8.7 to 158) | 31.6 (-4.9 to 80.6) | 31.3 (-4.2 to 79.7) | 219.2 (-29.1 to 545) | 118.1 (-15.1 to 306.5) | 101.1 (-14.9 to 249.7) | 248.5 (178.5 to 343.6) | 273.4 (158.8 to 406) | 223.3 (155.6 to 334.5) |
|  |  | Deaths | Age-standardized | 0.2 (0 to 0.6) | 0.3 (-0.1 to 0.7) | 0.2 (0 to 0.5) | 0.2 (0 to 0.6) | 0.3 (0 to 0.8) | 0.2 (0 to 0.4) | 5.2 (-26.4 to 51.7) | 11.2 (-30.7 to 69.9) | -2.6 (-30.1 to 80.8) |
|  |  |  | All ages | 2 (-0.3 to 5.1) | 1.2 (-0.2 to 3.2) | 0.8 (-0.1 to 2) | 9.8 (-1.5 to 24.1) | 6.3 (-0.8 to 16.6) | 3.5 (-0.6 to 8.3) | 385.6 (244.1 to 591.1) | 413.1 (217.6 to 671.2) | 342.3 (211.8 to 693.8) |
|  | Smoking | DALYs (Disability-Adjusted Life Years) | Age-standardized | 1.9 (1.1 to 2.9) | 0.7 (0.3 to 1.1) | 3 (1.7 to 4.8) | 1.9 (1.1 to 3.1) | 0.7 (0.4 to 1.2) | 3.2 (1.7 to 5.1) | 2.9 (-20.8 to 37.1) | 2.3 (-43.9 to 80.3) | 6 (-21.3 to 45.5) |
|  |  |  | All ages | 38.3 (21.7 to 61) | 6.5 (3.2 to 10.9) | 31.8 (17.6 to 51.2) | 107.8 (59.6 to 170) | 19.2 (9.5 to 33.6) | 88.6 (47.8 to 142.4) | 181.2 (113.1 to 268.2) | 194.8 (64.4 to 408.6) | 178.4 (102.9 to 271.4) |
|  |  | Deaths | Age-standardized | 0.1 (0 to 0.1) | 0 (0 to 0) | 0.1 (0 to 0.1) | 0.1 (0 to 0.1) | 0 (0 to 0.1) | 0.1 (0 to 0.1) | -2.9 (-37.5 to 77.1) | 2.3 (-58.7 to 141.1) | -5.2 (-42.4 to 74.8) |
|  |  |  | All ages | 0.7 (0.4 to 1.1) | 0.2 (0.1 to 0.3) | 0.5 (0.3 to 0.9) | 2.3 (1.2 to 3.8) | 0.6 (0.3 to 1.1) | 1.8 (0.9 to 2.9) | 232.2 (112.7 to 458.4) | 261.7 (63.1 to 690.9) | 223.8 (92.4 to 475) |
| Khuzestan | All risk factors | DALYs (Disability-Adjusted Life Years) | Age-standardized | 22.8 (10.2 to 37.5) | 26.2 (11 to 44.7) | 18.9 (8 to 32.9) | 29.5 (14.7 to 45.4) | 34.3 (16.6 to 54.2) | 25 (12.3 to 39.4) | 29.3 (-1 to 77.7) | 30.8 (-10.6 to 88.8) | 31.8 (2.3 to 90.2) |
|  |  |  | All ages | 224 (99.2 to 366.1) | 128.4 (54.8 to 213.5) | 95.6 (41.9 to 164) | 920.8 (467.9 to 1410.2) | 520.5 (260.9 to 822.8) | 400.3 (201.1 to 625.9) | 311 (218.4 to 455.1) | 305.4 (182.9 to 486.1) | 318.5 (229.6 to 490.3) |
|  |  | Deaths | Age-standardized | 1 (0.4 to 1.7) | 1.3 (0.5 to 2.4) | 0.7 (0.2 to 1.2) | 1.2 (0.6 to 1.9) | 1.8 (0.8 to 2.9) | 0.8 (0.3 to 1.2) | 22.5 (-18.4 to 85) | 32.4 (-21.4 to 118.4) | 17.6 (-23.4 to 133.4) |
|  |  |  | All ages | 8.3 (3.3 to 13.9) | 5.7 (2.2 to 10.1) | 2.6 (0.9 to 4.7) | 34.1 (16.3 to 53.3) | 23.2 (10.7 to 37.6) | 10.9 (4.9 to 17.5) | 313.1 (176.1 to 514.7) | 308.2 (147.5 to 555.6) | 323.8 (174.2 to 751.1) |
|  | Diet high in sodium | DALYs (Disability-Adjusted Life Years) | Age-standardized | 0.4 (0 to 2.4) | 0.3 (0 to 2.1) | 0.5 (0 to 2.8) | 0.4 (0 to 2.5) | 0.3 (0 to 2.3) | 0.5 (0 to 2.9) | 2.3 (-97.1 to 1477.9) | 0.3 (-32741.1 to 28322.9) | 2.9 (-96.9 to 1854.5) |
|  |  |  | All ages | 4.2 (0 to 24.7) | 1.5 (0 to 10.9) | 2.7 (0 to 13.9) | 13.1 (0 to 77.6) | 4.6 (0 to 34.8) | 8.5 (0 to 45.6) | 211 (-86.4 to 4739.1) | 205.1 (-1070275.4 to 85487.5) | 214.3 (-86.3 to 4860.7) |
|  |  | Deaths | Age-standardized | 0 (0 to 0.1) | 0 (0 to 0.1) | 0 (0 to 0.1) | 0 (0 to 0.1) | 0 (0 to 0.1) | 0 (0 to 0.1) | -2.5 (-97.1 to 1467.1) | 2.3 (-31969.5 to 24698.2) | -7.5 (-97.4 to 1883.3) |
|  |  |  | All ages | 0.1 (0 to 0.8) | 0.1 (0 to 0.4) | 0.1 (0 to 0.4) | 0.4 (0 to 2.6) | 0.2 (0 to 1.5) | 0.2 (0 to 1.2) | 212.4 (-91.4 to 4483.1) | 208.1 (-329491.9 to 67384.1) | 216.2 (-91.4 to 5065) |
|  | High alcohol use | DALYs (Disability-Adjusted Life Years) | Age-standardized | 0 (0 to 0) | 0 (0 to 0) | 0 (0 to 0) | 0.2 (0.1 to 0.3) | 0 (0 to 0.1) | 0.3 (0.2 to 0.5) | NA | NA | NA |
|  |  |  | All ages | 0 (0 to 0) | 0 (0 to 0) | 0 (0 to 0) | 6.6 (3.4 to 10.7) | 0.5 (0.2 to 1) | 6.1 (3.2 to 9.7) | NA | NA | NA |
|  |  | Deaths | Age-standardized | 0 (0 to 0) | 0 (0 to 0) | 0 (0 to 0) | 0 (0 to 0) | 0 (0 to 0) | 0 (0 to 0) | NA | NA | NA |
|  |  |  | All ages | 0 (0 to 0) | 0 (0 to 0) | 0 (0 to 0) | 0.1 (0.1 to 0.2) | 0 (0 to 0) | 0.1 (0.1 to 0.2) | NA | NA | NA |
|  | High body-mass index | DALYs (Disability-Adjusted Life Years) | Age-standardized | 3.2 (1.2 to 5.9) | 5.1 (1.9 to 9.6) | 1.2 (0.3 to 2.4) | 9.5 (3.8 to 16.3) | 12.7 (5.4 to 22.4) | 6.4 (2.4 to 11.7) | 199.7 (66.8 to 439.4) | 150.7 (30 to 347.7) | 443.7 (186.4 to 1434.6) |
|  |  |  | All ages | 33.9 (13.3 to 58.8) | 26.9 (10.3 to 48.1) | 7.1 (2.2 to 13.8) | 301.3 (119.1 to 517.1) | 197.4 (83.9 to 347.9) | 103.8 (39.1 to 185.4) | 787.5 (436.7 to 1362.3) | 634.7 (300.5 to 1082.4) | 1368.3 (750.3 to 3596.7) |
|  |  | Deaths | Age-standardized | 0.1 (0 to 0.3) | 0.2 (0.1 to 0.5) | 0 (0 to 0.1) | 0.4 (0.2 to 0.7) | 0.6 (0.3 to 1.2) | 0.2 (0.1 to 0.4) | 211.6 (39.5 to 676.5) | 185.1 (16.9 to 644.3) | 566.4 (132.7 to 3205.8) |
|  |  |  | All ages | 1.1 (0.4 to 2.3) | 1 (0.3 to 2.1) | 0.1 (0 to 0.4) | 11.1 (4.4 to 19.1) | 8.3 (3.3 to 14.8) | 2.9 (1 to 5.6) | 878.2 (383 to 1900.3) | 729.8 (270.6 to 1694) | 1926 (715.2 to 7044.7) |
|  | High systolic blood pressure | DALYs (Disability-Adjusted Life Years) | Age-standardized | 20 (6.5 to 35.1) | 23.2 (7.3 to 42.5) | 16.3 (5.3 to 30.3) | 23.4 (8 to 39.8) | 27.5 (8.9 to 48.5) | 19.6 (6.5 to 34.5) | 17.2 (-9.3 to 53.2) | 18.5 (-17.8 to 68.9) | 19.8 (-8.6 to 68.1) |
|  |  |  | All ages | 191.8 (60.9 to 338) | 112.1 (34.7 to 203.1) | 79.7 (26 to 146.7) | 720.6 (245 to 1226.5) | 413.7 (136.5 to 729.6) | 306.9 (102.2 to 547.3) | 275.7 (194.6 to 391.6) | 269.2 (160.4 to 425.4) | 285 (196.8 to 441) |
|  |  | Deaths | Age-standardized | 0.9 (0.3 to 1.6) | 1.2 (0.4 to 2.3) | 0.6 (0.2 to 1.1) | 1 (0.3 to 1.7) | 1.4 (0.5 to 2.6) | 0.6 (0.2 to 1.1) | 9.5 (-25.8 to 62.6) | 18.6 (-29.2 to 91.2) | 4.8 (-31.2 to 99.9) |
|  |  |  | All ages | 7.4 (2.3 to 13.1) | 5.1 (1.6 to 9.5) | 2.3 (0.7 to 4.4) | 27.5 (9.3 to 47.4) | 18.8 (6.3 to 33.6) | 8.7 (2.9 to 15.4) | 272.9 (154.2 to 453.7) | 268.5 (124 to 498) | 282.7 (146.7 to 627.4) |
|  | Lead exposure | DALYs (Disability-Adjusted Life Years) | Age-standardized | 4.3 (-0.6 to 10.9) | 4.7 (-0.7 to 12) | 3.9 (-0.6 to 9.5) | 4.5 (-0.6 to 11.2) | 4.9 (-0.7 to 11.9) | 4.2 (-0.6 to 10.6) | 3.7 (-17.7 to 30) | 4.3 (-25.2 to 39.8) | 5.9 (-15 to 42.3) |
|  |  |  | All ages | 41.6 (-5.8 to 104) | 22.5 (-3.3 to 57.7) | 19.1 (-2.6 to 47) | 134.4 (-18.8 to 334.5) | 70.2 (-9.7 to 174) | 64.1 (-9.2 to 165.6) | 223 (158.1 to 302.6) | 212.1 (126.3 to 321) | 235.8 (171.2 to 343.7) |
|  |  | Deaths | Age-standardized | 0.2 (0 to 0.5) | 0.2 (0 to 0.6) | 0.1 (0 to 0.4) | 0.2 (0 to 0.5) | 0.3 (0 to 0.7) | 0.1 (0 to 0.4) | 4.4 (-25.3 to 46.9) | 13.8 (-29.9 to 65.7) | -1.3 (-31.5 to 75.5) |
|  |  |  | All ages | 1.6 (-0.3 to 4) | 1 (-0.2 to 2.7) | 0.6 (-0.1 to 1.4) | 5.5 (-0.9 to 14.3) | 3.5 (-0.5 to 8.9) | 2 (-0.4 to 5.2) | 247.8 (147.1 to 390.6) | 242.2 (111.6 to 395.5) | 257.9 (147.1 to 526.3) |
|  | Smoking | DALYs (Disability-Adjusted Life Years) | Age-standardized | 1.7 (0.9 to 2.7) | 0.7 (0.3 to 1.1) | 2.8 (1.5 to 4.5) | 2 (1.1 to 3.1) | 0.7 (0.3 to 1.2) | 3.2 (1.7 to 5.1) | 13.3 (-12.3 to 46.5) | 9.2 (-34.9 to 82.3) | 14.2 (-13.2 to 51.5) |
|  |  |  | All ages | 21.1 (11.7 to 32.8) | 3.9 (1.9 to 6.4) | 17.2 (9.3 to 27.1) | 72.7 (40.1 to 115.1) | 13.1 (6.3 to 22.3) | 59.6 (31.9 to 95.2) | 245.2 (164.4 to 341.2) | 239.8 (103.2 to 446.1) | 246.4 (167 to 356) |
|  |  | Deaths | Age-standardized | 0 (0 to 0.1) | 0 (0 to 0) | 0.1 (0 to 0.1) | 0 (0 to 0.1) | 0 (0 to 0) | 0.1 (0 to 0.1) | 7.6 (-30.8 to 80.8) | 10.4 (-46.9 to 140.8) | 2 (-36.8 to 85.9) |
|  |  |  | All ages | 0.4 (0.2 to 0.7) | 0.1 (0 to 0.2) | 0.3 (0.2 to 0.5) | 1.5 (0.7 to 2.3) | 0.4 (0.2 to 0.7) | 1.1 (0.5 to 1.8) | 238.2 (124.3 to 449.3) | 235.5 (68.5 to 571.5) | 239.1 (105.6 to 501) |
| Kohgiluyeh and Boyer-Ahmad | All risk factors | DALYs (Disability-Adjusted Life Years) | Age-standardized | 17.8 (7.4 to 30) | 20.9 (8.1 to 36.1) | 14.6 (6 to 25.1) | 25.2 (12.9 to 38.9) | 31.2 (15.2 to 48.9) | 20.6 (10.8 to 32.6) | 41.3 (7.7 to 104.8) | 49.5 (0.5 to 122.8) | 40.9 (7 to 111.7) |
|  |  |  | All ages | 20 (9.1 to 33.4) | 10.7 (4.3 to 18.1) | 9.4 (4.1 to 15.4) | 126.1 (65.3 to 195.9) | 69.5 (34 to 109) | 56.5 (29.5 to 88.8) | 528.9 (389.4 to 747.5) | 551.2 (351.5 to 833.4) | 503.5 (359.9 to 772.4) |
|  |  | Deaths | Age-standardized | 0.7 (0.3 to 1.3) | 1 (0.3 to 1.9) | 0.4 (0.1 to 0.8) | 1 (0.5 to 1.6) | 1.6 (0.7 to 2.6) | 0.6 (0.3 to 0.9) | 39.1 (-11.7 to 141) | 59.8 (-10.3 to 183.7) | 34.7 (-17.5 to 199.9) |
|  |  |  | All ages | 0.5 (0.2 to 1) | 0.4 (0.1 to 0.7) | 0.2 (0.1 to 0.3) | 4.7 (2.3 to 7.5) | 3.2 (1.5 to 5.2) | 1.5 (0.7 to 2.6) | 763.2 (454.4 to 1379.8) | 744.1 (382.1 to 1330) | 805.3 (451 to 1859.4) |
|  | Diet high in sodium | DALYs (Disability-Adjusted Life Years) | Age-standardized | 0.3 (0 to 1.9) | 0.2 (0 to 1.7) | 0.4 (0 to 2.3) | 0.4 (0 to 2.3) | 0.3 (0 to 1.9) | 0.5 (0 to 2.4) | 9.6 (-95.6 to 913) | 10.6 (-43453.1 to 153285.6) | 8.9 (-95.7 to 973.1) |
|  |  |  | All ages | 0.4 (0 to 2.4) | 0.1 (0 to 0.9) | 0.3 (0 to 1.5) | 1.8 (0 to 11.4) | 0.6 (0 to 4.3) | 1.2 (0 to 6.5) | 333.6 (-76.1 to 3075.9) | 342.9 (-1126388.7 to 1945390.3) | 329.4 (-76.1 to 3164.7) |
|  |  | Deaths | Age-standardized | 0 (0 to 0.1) | 0 (0 to 0.1) | 0 (0 to 0.1) | 0 (0 to 0.1) | 0 (0 to 0.1) | 0 (0 to 0.1) | 10.5 (-98.1 to 1315.1) | 19.3 (-17662.9 to 57174.2) | 3.8 (-98.3 to 1272.3) |
|  |  |  | All ages | 0 (0 to 0) | 0 (0 to 0) | 0 (0 to 0) | 0.1 (0 to 0.4) | 0 (0 to 0.2) | 0 (0 to 0.2) | 500.8 (-87.3 to 4773.2) | 474.7 (-100906.4 to 209058.9) | 522 (-87.2 to 5269.1) |
|  | High alcohol use | DALYs (Disability-Adjusted Life Years) | Age-standardized | 0 (0 to 0) | 0 (0 to 0) | 0 (0 to 0) | 0.2 (0.1 to 0.3) | 0 (0 to 0.1) | 0.3 (0.2 to 0.5) | NA | NA | NA |
|  |  |  | All ages | 0 (0 to 0) | 0 (0 to 0) | 0 (0 to 0) | 1 (0.5 to 1.5) | 0.1 (0 to 0.1) | 0.9 (0.5 to 1.4) | NA | NA | NA |
|  |  | Deaths | Age-standardized | 0 (0 to 0) | 0 (0 to 0) | 0 (0 to 0) | 0 (0 to 0) | 0 (0 to 0) | 0 (0 to 0) | NA | NA | NA |
|  |  |  | All ages | 0 (0 to 0) | 0 (0 to 0) | 0 (0 to 0) | 0 (0 to 0) | 0 (0 to 0) | 0 (0 to 0) | NA | NA | NA |
|  | High body-mass index | DALYs (Disability-Adjusted Life Years) | Age-standardized | 2.3 (0.8 to 4.3) | 3.6 (1.3 to 7.1) | 1 (0.3 to 2) | 9 (3.9 to 15.6) | 11.2 (4.6 to 20.2) | 7.2 (2.8 to 12.9) | 294.8 (110 to 612.3) | 208.6 (44.1 to 517.6) | 634.1 (268 to 1827.6) |
|  |  |  | All ages | 3.1 (1.1 to 5.3) | 2.2 (0.9 to 4) | 0.8 (0.3 to 1.6) | 45.5 (19.6 to 79.6) | 25.6 (10.7 to 45.1) | 19.9 (7.8 to 35.8) | 1386.5 (756.4 to 2324) | 1042.5 (485.6 to 1891.1) | 2329 (1264.1 to 5743.7) |
|  |  | Deaths | Age-standardized | 0.1 (0 to 0.2) | 0.1 (0 to 0.3) | 0 (0 to 0.1) | 0.3 (0.1 to 0.6) | 0.5 (0.2 to 1.1) | 0.2 (0.1 to 0.4) | 325.2 (86.6 to 1141.8) | 283.9 (48 to 1072.4) | 915.2 (215.3 to 5290.6) |
|  |  |  | All ages | 0.1 (0 to 0.1) | 0.1 (0 to 0.1) | 0 (0 to 0) | 1.6 (0.7 to 3) | 1.1 (0.4 to 2.1) | 0.6 (0.2 to 1.1) | 2150.2 (1022.7 to 4710.6) | 1648.8 (635.4 to 3865.1) | 4992.5 (1935.5 to 17077.6) |
|  | High systolic blood pressure | DALYs (Disability-Adjusted Life Years) | Age-standardized | 15.6 (5.1 to 28.1) | 18.7 (5.9 to 34.2) | 12.4 (3.9 to 22.6) | 19.2 (6.2 to 33.4) | 25.3 (8 to 43) | 14.6 (4.6 to 25.9) | 23.6 (-7.8 to 65.6) | 35.5 (-10.7 to 99.7) | 17.6 (-14.1 to 65.7) |
|  |  |  | All ages | 16.7 (5.7 to 29.9) | 9.3 (2.9 to 16.6) | 7.4 (2.2 to 13.4) | 95.1 (30.4 to 166.7) | 55.8 (17.8 to 95.1) | 39.2 (12.2 to 70.8) | 469.8 (328.3 to 662.8) | 502.5 (303.3 to 764.9) | 428.9 (265.3 to 667.9) |
|  |  | Deaths | Age-standardized | 0.7 (0.2 to 1.2) | 0.9 (0.3 to 1.8) | 0.4 (0.1 to 0.7) | 0.8 (0.3 to 1.4) | 1.3 (0.4 to 2.4) | 0.4 (0.1 to 0.8) | 21.2 (-23.9 to 100.4) | 43.4 (-21.4 to 150.9) | 10.2 (-35.1 to 129.9) |
|  |  |  | All ages | 0.5 (0.1 to 0.9) | 0.3 (0.1 to 0.7) | 0.1 (0 to 0.3) | 3.7 (1.2 to 6.5) | 2.6 (0.8 to 4.6) | 1.1 (0.4 to 2.2) | 671.2 (377.7 to 1164.6) | 671.4 (333 to 1200.9) | 670.9 (336.4 to 1541.8) |
|  | Lead exposure | DALYs (Disability-Adjusted Life Years) | Age-standardized | 4.1 (-0.6 to 10) | 4.3 (-0.6 to 10.9) | 3.8 (-0.6 to 9.5) | 4.4 (-0.6 to 10.9) | 4.8 (-0.7 to 12) | 4.3 (-0.6 to 10.8) | 9.3 (-13.9 to 38) | 11.4 (-25.2 to 53.5) | 12 (-10.3 to 50.9) |
|  |  |  | All ages | 4.4 (-0.6 to 11) | 2.1 (-0.3 to 5.5) | 2.3 (-0.3 to 5.7) | 21.8 (-3 to 54.2) | 10.3 (-1.5 to 25.9) | 11.5 (-1.5 to 28.9) | 394.3 (291.9 to 524.5) | 379.3 (229.7 to 566.4) | 408.6 (304.7 to 568.8) |
|  |  | Deaths | Age-standardized | 0.2 (0 to 0.4) | 0.2 (0 to 0.6) | 0.1 (0 to 0.3) | 0.2 (0 to 0.5) | 0.3 (0 to 0.7) | 0.1 (0 to 0.3) | 11.8 (-28.1 to 71.2) | 26.1 (-28.6 to 96.6) | 10.2 (-27.9 to 123.5) |
|  |  |  | All ages | 0.1 (0 to 0.3) | 0.1 (0 to 0.2) | 0 (0 to 0.1) | 0.9 (-0.1 to 2.3) | 0.5 (-0.1 to 1.3) | 0.4 (-0.1 to 0.9) | 608.1 (355 to 1003.4) | 562.2 (270.5 to 943.8) | 686.6 (405.3 to 1496.8) |
|  | Smoking | DALYs (Disability-Adjusted Life Years) | Age-standardized | 1.5 (0.8 to 2.4) | 0.5 (0.3 to 0.9) | 2.3 (1.2 to 3.9) | 1.9 (1.1 to 3) | 0.6 (0.3 to 1.2) | 3 (1.7 to 4.8) | 26.2 (-1.5 to 67.9) | 25.3 (-28.8 to 117.3) | 27.4 (-1.5 to 72.5) |
|  |  |  | All ages | 2.4 (1.3 to 3.9) | 0.4 (0.2 to 0.7) | 2 (1.1 to 3.4) | 10.5 (5.9 to 17.2) | 1.7 (0.8 to 3) | 8.8 (5 to 14.3) | 338.2 (243.7 to 482.2) | 349.8 (164.9 to 689.8) | 336 (230.4 to 490.5) |
|  |  | Deaths | Age-standardized | 0 (0 to 0.1) | 0 (0 to 0) | 0 (0 to 0.1) | 0 (0 to 0.1) | 0 (0 to 0) | 0.1 (0 to 0.1) | 30.8 (-20.9 to 153.7) | 32.5 (-47 to 202.2) | 22 (-31.5 to 161.1) |
|  |  |  | All ages | 0 (0 to 0.1) | 0 (0 to 0) | 0 (0 to 0) | 0.2 (0.1 to 0.3) | 0 (0 to 0.1) | 0.2 (0.1 to 0.3) | 509.6 (266.6 to 1050) | 468.1 (144.1 to 1148.8) | 522.9 (260.6 to 1222) |
| Kurdistan | All risk factors | DALYs (Disability-Adjusted Life Years) | Age-standardized | 20 (8.3 to 32.6) | 21.3 (8.8 to 35.6) | 18.7 (7.5 to 30.9) | 26.6 (13.5 to 41.3) | 29.3 (14.1 to 44.5) | 24.2 (12.1 to 38.9) | 33.2 (2 to 81.4) | 37.6 (-3.8 to 97) | 29.7 (-3.4 to 95.1) |
|  |  |  | All ages | 79.5 (35.1 to 128.5) | 37.5 (15.4 to 61.4) | 42 (18 to 69.6) | 372.1 (189.2 to 577.4) | 200.3 (97.9 to 300.8) | 171.8 (86.8 to 274.6) | 368.1 (259.4 to 533.3) | 434 (282.2 to 647) | 309.2 (213 to 490.4) |
|  |  | Deaths | Age-standardized | 0.8 (0.3 to 1.4) | 1 (0.4 to 1.8) | 0.7 (0.2 to 1.2) | 1.1 (0.5 to 1.7) | 1.5 (0.7 to 2.4) | 0.8 (0.3 to 1.3) | 32.1 (-12.7 to 108.9) | 47.1 (-9.4 to 137.4) | 15.3 (-30.6 to 121.7) |
|  |  |  | All ages | 2.5 (0.9 to 4.2) | 1.4 (0.6 to 2.5) | 1 (0.3 to 1.9) | 14.3 (7 to 22.7) | 9.2 (4.2 to 14.7) | 5.2 (2.3 to 8.5) | 477.7 (286.2 to 802) | 538.3 (295.6 to 924.5) | 394.9 (196.6 to 898) |
|  | Diet high in sodium | DALYs (Disability-Adjusted Life Years) | Age-standardized | 0.4 (0 to 2.3) | 0.2 (0 to 1.8) | 0.5 (0 to 2.7) | 0.4 (0 to 2.5) | 0.3 (0 to 2.1) | 0.5 (0 to 2.8) | 1.4 (-94.6 to 1140.8) | 6.4 (-574.6 to 43270.8) | 3.3 (-93.5 to 1125.1) |
|  |  |  | All ages | 1.7 (0 to 9.7) | 0.5 (0 to 3.3) | 1.2 (0 to 6.3) | 5.6 (0 to 35.6) | 1.9 (0 to 14.6) | 3.7 (0 to 20.2) | 229.2 (-82.2 to 3366.1) | 292.4 (-1800 to 196563.3) | 204.8 (-82.1 to 3679.8) |
|  |  | Deaths | Age-standardized | 0 (0 to 0.1) | 0 (0 to 0.1) | 0 (0 to 0.1) | 0 (0 to 0.1) | 0 (0 to 0.1) | 0 (0 to 0.1) | 1 (-97.3 to 1260.6) | 15.4 (-690.4 to 91932.9) | -8.5 (-97 to 1298.5) |
|  |  |  | All ages | 0 (0 to 0.3) | 0 (0 to 0.1) | 0 (0 to 0.1) | 0.2 (0 to 1.3) | 0.1 (0 to 0.6) | 0.1 (0 to 0.7) | 298.5 (-91.4 to 3338.2) | 373.8 (-3697.2 to 80950.6) | 256.9 (-91.4 to 3516.5) |
|  | High alcohol use | DALYs (Disability-Adjusted Life Years) | Age-standardized | 0 (0 to 0) | 0 (0 to 0) | 0 (0 to 0) | 0.2 (0.1 to 0.3) | 0 (0 to 0.1) | 0.3 (0.2 to 0.5) | NA | NA | NA |
|  |  |  | All ages | 0 (0 to 0) | 0 (0 to 0) | 0 (0 to 0) | 2.6 (1.3 to 4.2) | 0.2 (0.1 to 0.4) | 2.4 (1.2 to 3.8) | NA | NA | NA |
|  |  | Deaths | Age-standardized | 0 (0 to 0) | 0 (0 to 0) | 0 (0 to 0) | 0 (0 to 0) | 0 (0 to 0) | 0 (0 to 0) | NA | NA | NA |
|  |  |  | All ages | 0 (0 to 0) | 0 (0 to 0) | 0 (0 to 0) | 0.1 (0 to 0.1) | 0 (0 to 0) | 0.1 (0 to 0.1) | NA | NA | NA |
|  | High body-mass index | DALYs (Disability-Adjusted Life Years) | Age-standardized | 2.1 (0.7 to 4) | 3.8 (1.4 to 7.3) | 0.7 (0.1 to 1.6) | 8.1 (3.4 to 13.7) | 10.4 (4.1 to 18.2) | 5.8 (2.1 to 10.6) | 281.3 (115.7 to 580.4) | 176 (38.9 to 393.8) | 728.9 (270.1 to 3474.9) |
|  |  |  | All ages | 9.9 (3.8 to 17.5) | 7.8 (3.1 to 14.2) | 2.1 (0.6 to 4.6) | 113.6 (47.5 to 194.3) | 72.1 (28.1 to 127.4) | 41.6 (15.5 to 74.9) | 1049.5 (620.2 to 1714.2) | 826.1 (414.6 to 1362.4) | 1876.3 (909.5 to 7110.3) |
|  |  | Deaths | Age-standardized | 0.1 (0 to 0.2) | 0.1 (0 to 0.4) | 0 (0 to 0.1) | 0.3 (0.1 to 0.6) | 0.5 (0.2 to 0.9) | 0.2 (0.1 to 0.4) | 327.3 (96.2 to 1006.9) | 247.7 (40.8 to 857.7) | 1051.8 (215.3 to 9742.6) |
|  |  |  | All ages | 0.3 (0.1 to 0.6) | 0.2 (0.1 to 0.5) | 0 (0 to 0.1) | 4.4 (1.7 to 7.7) | 3.1 (1.2 to 5.7) | 1.3 (0.4 to 2.6) | 1521.4 (713.4 to 3248.4) | 1229.8 (485.2 to 2734.5) | 3406.1 (1113.4 to 19215.6) |
|  | High systolic blood pressure | DALYs (Disability-Adjusted Life Years) | Age-standardized | 17.6 (6 to 30.4) | 19 (6.3 to 33.3) | 16.2 (4.9 to 28.9) | 21.2 (7.1 to 35.8) | 23.6 (7.6 to 39.8) | 19 (6.2 to 33.9) | 20.3 (-8.8 to 57.5) | 24.4 (-13 to 81.1) | 16.8 (-13.7 to 62.6) |
|  |  |  | All ages | 67.6 (21.5 to 116.4) | 32.5 (10.5 to 56.8) | 35.1 (10.3 to 63.2) | 293 (98.4 to 498.2) | 160.3 (52.9 to 273.2) | 132.6 (42.9 to 239) | 333.7 (226.9 to 467) | 393.8 (252.3 to 628.4) | 278.1 (180.1 to 433.4) |
|  |  | Deaths | Age-standardized | 0.8 (0.2 to 1.3) | 0.9 (0.3 to 1.7) | 0.6 (0.2 to 1.2) | 0.9 (0.3 to 1.6) | 1.2 (0.4 to 2.1) | 0.6 (0.2 to 1.1) | 17.4 (-22.9 to 74.7) | 31 (-21.5 to 121) | 2.1 (-38.3 to 77.4) |
|  |  |  | All ages | 2.2 (0.7 to 3.9) | 1.3 (0.4 to 2.4) | 0.9 (0.2 to 1.7) | 11.6 (3.9 to 20.3) | 7.4 (2.3 to 13) | 4.1 (1.5 to 7.4) | 423.3 (245.3 to 676.4) | 477.4 (245.7 to 848.4) | 348 (169.1 to 707.3) |
|  | Lead exposure | DALYs (Disability-Adjusted Life Years) | Age-standardized | 4.5 (-0.7 to 11.2) | 4.6 (-0.7 to 11.6) | 4.5 (-0.7 to 11.3) | 4.9 (-0.7 to 11.9) | 5.2 (-0.8 to 12.6) | 4.6 (-0.7 to 11.4) | 7.3 (-14.7 to 35.2) | 12.4 (-18.8 to 49.9) | 3.3 (-21.2 to 39.3) |
|  |  |  | All ages | 17.4 (-2.5 to 43.3) | 7.9 (-1.1 to 20) | 9.5 (-1.4 to 23.9) | 66.4 (-9.6 to 161.8) | 34.2 (-5.1 to 83.5) | 32.2 (-4.5 to 80) | 281.8 (204.6 to 376.5) | 330.8 (216.4 to 481.9) | 240.6 (164.3 to 353.1) |
|  |  | Deaths | Age-standardized | 0.2 (0 to 0.5) | 0.2 (0 to 0.6) | 0.2 (0 to 0.5) | 0.2 (0 to 0.5) | 0.3 (0 to 0.7) | 0.2 (0 to 0.4) | 12.2 (-20.5 to 63.3) | 28.9 (-17.2 to 96.4) | -5.3 (-40.5 to 67.3) |
|  |  |  | All ages | 0.6 (-0.1 to 1.5) | 0.3 (-0.1 to 0.8) | 0.3 (0 to 0.7) | 2.9 (-0.5 to 6.9) | 1.8 (-0.3 to 4.2) | 1.1 (-0.2 to 2.8) | 399.6 (245.7 to 626.6) | 454.5 (253.1 to 746.4) | 332.7 (171.2 to 657.2) |
|  | Smoking | DALYs (Disability-Adjusted Life Years) | Age-standardized | 1.8 (1 to 2.8) | 0.6 (0.3 to 1) | 2.8 (1.6 to 4.5) | 2 (1.1 to 3.1) | 0.7 (0.3 to 1.2) | 3.3 (1.9 to 5.3) | 10.8 (-16.7 to 49.9) | 16.2 (-31.9 to 91.2) | 17.2 (-14 to 66) |
|  |  |  | All ages | 9.4 (5.2 to 14.8) | 1.4 (0.7 to 2.4) | 8 (4.4 to 12.5) | 30.9 (17.2 to 48.1) | 5.2 (2.4 to 8.9) | 25.7 (14.1 to 40.5) | 228.6 (150.6 to 337.8) | 267.1 (120.4 to 509.7) | 221.7 (140 to 345.1) |
|  |  | Deaths | Age-standardized | 0 (0 to 0.1) | 0 (0 to 0) | 0.1 (0 to 0.1) | 0 (0 to 0.1) | 0 (0 to 0) | 0.1 (0 to 0.1) | 7.8 (-38.9 to 88.6) | 23.5 (-45.7 to 164.9) | 4.5 (-44.8 to 96.5) |
|  |  |  | All ages | 0.2 (0.1 to 0.3) | 0 (0 to 0.1) | 0.1 (0.1 to 0.2) | 0.7 (0.3 to 1.1) | 0.1 (0.1 to 0.3) | 0.5 (0.3 to 0.8) | 284.3 (122.4 to 561.6) | 344 (109.5 to 831.2) | 270.4 (98.4 to 579.8) |
| Lorestan | All risk factors | DALYs (Disability-Adjusted Life Years) | Age-standardized | 19.8 (8.2 to 32) | 22.5 (9.3 to 37.8) | 17.3 (7.1 to 29.7) | 26.5 (13.5 to 40.2) | 27.7 (14.2 to 43.5) | 24.6 (12.1 to 38.1) | 33.4 (0.9 to 96.4) | 23 (-14.9 to 91.4) | 42.2 (9.7 to 116.5) |
|  |  |  | All ages | 89.6 (39.9 to 141.7) | 45.5 (19.5 to 74.9) | 44.1 (19.9 to 71.6) | 337.3 (174.6 to 515.3) | 192.2 (100.4 to 300.9) | 145.1 (72.6 to 224) | 276.7 (192.7 to 441) | 322.6 (189.7 to 536.8) | 229.3 (157 to 372.1) |
|  |  | Deaths | Age-standardized | 0.8 (0.3 to 1.5) | 1.1 (0.4 to 2) | 0.6 (0.2 to 1.1) | 1.1 (0.6 to 1.7) | 1.3 (0.6 to 2.1) | 0.9 (0.4 to 1.4) | 36 (-8.9 to 132.8) | 25.9 (-26.2 to 124.7) | 39.4 (-10.4 to 209.8) |
|  |  |  | All ages | 2.8 (1.1 to 4.9) | 1.7 (0.6 to 3.1) | 1.1 (0.4 to 1.9) | 12.2 (6.1 to 18.6) | 8.1 (3.8 to 12.8) | 4.1 (1.8 to 6.6) | 334.9 (192.1 to 626.8) | 372.1 (183.3 to 712.5) | 276.5 (149 to 740.7) |
|  | Diet high in sodium | DALYs (Disability-Adjusted Life Years) | Age-standardized | 0.4 (0 to 2.1) | 0.3 (0 to 1.8) | 0.5 (0 to 2.6) | 0.4 (0 to 2.3) | 0.2 (0 to 2) | 0.5 (0 to 3.1) | 0.8 (-96.7 to 1557.9) | -1.1 (-361467.2 to 37975.3) | 11.6 (-96.3 to 1821.7) |
|  |  |  | All ages | 1.9 (0 to 10.2) | 0.6 (0 to 3.8) | 1.4 (0 to 6.9) | 5.1 (0 to 29.8) | 1.8 (0 to 14.2) | 3.3 (0 to 17.9) | 166.7 (-90.5 to 2798.6) | 224.6 (-3357855.1 to 46097.2) | 143 (-90.5 to 2801.6) |
|  |  | Deaths | Age-standardized | 0 (0 to 0.1) | 0 (0 to 0.1) | 0 (0 to 0.1) | 0 (0 to 0.1) | 0 (0 to 0.1) | 0 (0 to 0.1) | 2.7 (-98.1 to 2365.5) | -0.2 (-4552.4 to 174312.7) | 10.5 (-98.2 to 2817.1) |
|  |  |  | All ages | 0 (0 to 0.3) | 0 (0 to 0.1) | 0 (0 to 0.2) | 0.1 (0 to 1) | 0.1 (0 to 0.5) | 0.1 (0 to 0.5) | 206.1 (-94.5 to 4480.8) | 253.5 (-26930.4 to 522298.4) | 177.4 (-94.6 to 4480) |
|  | High alcohol use | DALYs (Disability-Adjusted Life Years) | Age-standardized | 0 (0 to 0) | 0 (0 to 0) | 0 (0 to 0) | 0.2 (0.1 to 0.3) | 0 (0 to 0.1) | 0.4 (0.2 to 0.6) | NA | NA | NA |
|  |  |  | All ages | 0 (0 to 0) | 0 (0 to 0) | 0 (0 to 0) | 2.6 (1.5 to 4.1) | 0.2 (0.1 to 0.4) | 2.4 (1.4 to 3.7) | NA | NA | NA |
|  |  | Deaths | Age-standardized | 0 (0 to 0) | 0 (0 to 0) | 0 (0 to 0) | 0 (0 to 0) | 0 (0 to 0) | 0 (0 to 0) | NA | NA | NA |
|  |  |  | All ages | 0 (0 to 0) | 0 (0 to 0) | 0 (0 to 0) | 0.1 (0 to 0.1) | 0 (0 to 0) | 0 (0 to 0.1) | NA | NA | NA |
|  | High body-mass index | DALYs (Disability-Adjusted Life Years) | Age-standardized | 2.5 (0.9 to 4.7) | 4.4 (1.6 to 8.5) | 0.9 (0.2 to 2) | 10 (4.2 to 17.5) | 11.9 (4.8 to 21) | 7.6 (3.1 to 13.9) | 294.2 (105.6 to 647.8) | 170.4 (27.5 to 381.1) | 732.4 (312 to 2622.9) |
|  |  |  | All ages | 13.2 (5 to 23.4) | 10.2 (3.7 to 18.5) | 3 (0.9 to 5.9) | 128.4 (55.2 to 225.8) | 84 (33.9 to 145.8) | 44.4 (18.7 to 81.8) | 871.4 (481.3 to 1492.4) | 723.1 (328.7 to 1235.1) | 1373.4 (714.9 to 3994.6) |
|  |  | Deaths | Age-standardized | 0.1 (0 to 0.2) | 0.2 (0.1 to 0.4) | 0 (0 to 0.1) | 0.4 (0.2 to 0.8) | 0.6 (0.2 to 1) | 0.3 (0.1 to 0.5) | 346.6 (89 to 1039.8) | 213.3 (21 to 702.6) | 1123.5 (283.8 to 7246) |
|  |  |  | All ages | 0.4 (0.1 to 0.7) | 0.3 (0.1 to 0.7) | 0.1 (0 to 0.1) | 4.6 (1.9 to 8.2) | 3.3 (1.3 to 6) | 1.3 (0.5 to 2.4) | 1147.1 (503.8 to 2401.9) | 949.6 (348.7 to 2051.5) | 2313 (954.6 to 9539) |
|  | High systolic blood pressure | DALYs (Disability-Adjusted Life Years) | Age-standardized | 17.2 (5.6 to 29.4) | 19.9 (6.2 to 35.5) | 14.8 (4.8 to 27.4) | 19.9 (6.2 to 34.1) | 21.1 (6.8 to 36.4) | 18.1 (5.5 to 32.2) | 15.2 (-15.6 to 53.6) | 6 (-29.5 to 54.7) | 22.7 (-10.1 to 76.2) |
|  |  |  | All ages | 75 (25.9 to 128.8) | 39.2 (12.7 to 67.8) | 35.9 (11.4 to 64.3) | 249.3 (79.5 to 427.7) | 145 (45.9 to 249.9) | 104.2 (32.6 to 184.5) | 232.2 (150.1 to 336.2) | 270.4 (156 to 432.2) | 190.5 (114.8 to 316) |
|  |  | Deaths | Age-standardized | 0.8 (0.2 to 1.4) | 1 (0.3 to 1.8) | 0.5 (0.1 to 1) | 0.9 (0.3 to 1.5) | 1 (0.3 to 1.9) | 0.6 (0.2 to 1.2) | 16 (-25.1 to 84.5) | 8 (-37.3 to 81.1) | 17.4 (-25.7 to 151.2) |
|  |  |  | All ages | 2.5 (0.8 to 4.5) | 1.5 (0.5 to 2.9) | 0.9 (0.2 to 1.8) | 9.4 (2.9 to 16.3) | 6.3 (1.8 to 11.2) | 3.1 (1 to 5.6) | 277.9 (146.1 to 492.5) | 310.5 (140.1 to 580) | 225.4 (108.1 to 597.2) |
|  | Lead exposure | DALYs (Disability-Adjusted Life Years) | Age-standardized | 4.5 (-0.6 to 11.7) | 4.6 (-0.7 to 12) | 4.5 (-0.7 to 11.4) | 4.8 (-0.7 to 12) | 4.7 (-0.7 to 11.5) | 5 (-0.7 to 12.3) | 6.9 (-15.4 to 37.8) | 1.6 (-28.2 to 36.6) | 12.7 (-8.2 to 61.9) |
|  |  |  | All ages | 19.8 (-2.7 to 49.9) | 9.1 (-1.4 to 23.8) | 10.7 (-1.5 to 27.5) | 59 (-8.1 to 146.4) | 31.1 (-4.4 to 78.1) | 27.8 (-3.8 to 68.5) | 198.3 (138.8 to 280.3) | 243.8 (145.7 to 359.9) | 159.8 (110.4 to 260.2) |
|  |  | Deaths | Age-standardized | 0.2 (0 to 0.5) | 0.2 (0 to 0.6) | 0.2 (0 to 0.4) | 0.2 (0 to 0.6) | 0.3 (0 to 0.6) | 0.2 (0 to 0.5) | 15.2 (-19.9 to 76.9) | 10.5 (-31.3 to 66.1) | 15.2 (-22.6 to 134.3) |
|  |  |  | All ages | 0.6 (-0.1 to 1.7) | 0.4 (-0.1 to 0.9) | 0.3 (0 to 0.8) | 2.4 (-0.4 to 5.8) | 1.5 (-0.2 to 3.7) | 0.9 (-0.1 to 2.3) | 266 (153.7 to 456.9) | 309.5 (155.3 to 519.6) | 212.6 (112.8 to 523.1) |
|  | Smoking | DALYs (Disability-Adjusted Life Years) | Age-standardized | 1.7 (0.9 to 2.7) | 0.6 (0.3 to 1) | 2.7 (1.5 to 4.3) | 1.9 (1 to 2.9) | 0.6 (0.3 to 1) | 3.4 (1.9 to 5.3) | 9.1 (-15.8 to 51.9) | 6.9 (-41.4 to 86) | 24.7 (-6.2 to 78.5) |
|  |  |  | All ages | 10.4 (5.6 to 16.3) | 1.6 (0.8 to 2.8) | 8.8 (4.7 to 14) | 28.9 (15.7 to 44.6) | 5 (2.5 to 8.5) | 23.9 (12.9 to 37.3) | 178.3 (115.2 to 283.2) | 213.2 (76.1 to 422.4) | 172 (110.3 to 281.9) |
|  |  | Deaths | Age-standardized | 0 (0 to 0.1) | 0 (0 to 0) | 0.1 (0 to 0.1) | 0 (0 to 0.1) | 0 (0 to 0) | 0.1 (0 to 0.1) | 7.8 (-30.6 to 106.2) | 3.9 (-55.1 to 148.5) | 23.3 (-26.5 to 153.1) |
|  |  |  | All ages | 0.2 (0.1 to 0.3) | 0 (0 to 0.1) | 0.2 (0.1 to 0.3) | 0.6 (0.3 to 0.9) | 0.1 (0.1 to 0.2) | 0.5 (0.2 to 0.7) | 205.8 (97 to 473) | 237 (50.4 to 644.3) | 198.2 (84.3 to 492.6) |
| Markazi | All risk factors | DALYs (Disability-Adjusted Life Years) | Age-standardized | 21.4 (9.4 to 34.5) | 25.1 (11 to 42.2) | 17.8 (7.4 to 29) | 25.6 (12.8 to 39.2) | 29.8 (14.6 to 46.4) | 21.6 (11 to 33.7) | 19.7 (-7.5 to 69.8) | 18.8 (-16.6 to 81.5) | 21.3 (-10.1 to 74.8) |
|  |  |  | All ages | 92 (41.1 to 147.4) | 49 (21.8 to 80.3) | 42.9 (19.3 to 68.4) | 399.4 (201.1 to 613.7) | 231.1 (112.9 to 363.3) | 168.3 (85.1 to 262.9) | 334.3 (243.1 to 490.8) | 371.2 (239.2 to 584.7) | 292.1 (191 to 447.8) |
|  |  | Deaths | Age-standardized | 1 (0.4 to 1.6) | 1.3 (0.5 to 2.4) | 0.6 (0.2 to 1) | 1 (0.5 to 1.6) | 1.5 (0.7 to 2.5) | 0.6 (0.3 to 1) | 9 (-26.3 to 75.4) | 15.5 (-29 to 98) | -0.9 (-37.9 to 79.9) |
|  |  |  | All ages | 3 (1.2 to 5.1) | 2 (0.8 to 3.5) | 1.1 (0.4 to 1.9) | 17 (8.4 to 26.5) | 11.9 (5.7 to 19.2) | 5.1 (2.4 to 8.3) | 456.3 (279.8 to 801.3) | 500.8 (273.6 to 926.4) | 373.7 (198.7 to 775.9) |
|  | Diet high in sodium | DALYs (Disability-Adjusted Life Years) | Age-standardized | 0.4 (0 to 2.5) | 0.3 (0 to 2) | 0.5 (0 to 2.6) | 0.4 (0 to 2.2) | 0.3 (0 to 2.1) | 0.5 (0 to 2.5) | -6.5 (-95.6 to 1058.9) | -6.9 (-77016.7 to 15989.5) | -3.9 (-95.6 to 1140.2) |
|  |  |  | All ages | 1.8 (0 to 10.8) | 0.6 (0 to 4.2) | 1.2 (0 to 6.6) | 5.5 (0 to 33.5) | 2 (0 to 15.8) | 3.5 (0 to 19.3) | 197.3 (-85.1 to 2856.5) | 230.8 (-271952.8 to 36887.7) | 181.3 (-85.1 to 3103.6) |
|  |  | Deaths | Age-standardized | 0 (0 to 0.1) | 0 (0 to 0.1) | 0 (0 to 0.1) | 0 (0 to 0.1) | 0 (0 to 0.1) | 0 (0 to 0.1) | -16.7 (-98.2 to 1312.3) | -10.1 (-5958.4 to 36245.4) | -21.7 (-98.8 to 1543.2) |
|  |  |  | All ages | 0 (0 to 0.3) | 0 (0 to 0.2) | 0 (0 to 0.2) | 0.2 (0 to 1.4) | 0.1 (0 to 0.7) | 0.1 (0 to 0.6) | 271.7 (-95.3 to 2884.4) | 321.1 (-49225.8 to 50304.4) | 234.9 (-95.4 to 3176) |
|  | High alcohol use | DALYs (Disability-Adjusted Life Years) | Age-standardized | 0 (0 to 0) | 0 (0 to 0) | 0 (0 to 0) | 0.2 (0.1 to 0.3) | 0 (0 to 0.1) | 0.3 (0.2 to 0.5) | NA | NA | NA |
|  |  |  | All ages | 0 (0 to 0) | 0 (0 to 0) | 0 (0 to 0) | 2.7 (1.4 to 4.3) | 0.2 (0.1 to 0.5) | 2.5 (1.4 to 4) | NA | NA | NA |
|  |  | Deaths | Age-standardized | 0 (0 to 0) | 0 (0 to 0) | 0 (0 to 0) | 0 (0 to 0) | 0 (0 to 0) | 0 (0 to 0) | NA | NA | NA |
|  |  |  | All ages | 0 (0 to 0) | 0 (0 to 0) | 0 (0 to 0) | 0.1 (0 to 0.1) | 0 (0 to 0) | 0.1 (0 to 0.1) | NA | NA | NA |
|  | High body-mass index | DALYs (Disability-Adjusted Life Years) | Age-standardized | 3 (1.1 to 5.5) | 4.8 (1.8 to 9.5) | 1.3 (0.4 to 2.6) | 9.6 (3.8 to 16.5) | 12.4 (5.2 to 22.4) | 6.8 (2.5 to 12.2) | 218.9 (68.2 to 453.8) | 159.5 (28.7 to 379.9) | 413.1 (182.6 to 1204.2) |
|  |  |  | All ages | 14.8 (5.4 to 25.4) | 10.9 (4.2 to 19.6) | 3.9 (1.3 to 7.6) | 150.2 (59.5 to 257.4) | 96.4 (40.3 to 172.9) | 53.8 (19.3 to 95.6) | 911.7 (502.2 to 1454) | 781.3 (368 to 1329.7) | 1277.1 (679.1 to 3127.8) |
|  |  | Deaths | Age-standardized | 0.1 (0 to 0.3) | 0.2 (0.1 to 0.5) | 0 (0 to 0.1) | 0.4 (0.2 to 0.7) | 0.6 (0.3 to 1.1) | 0.2 (0.1 to 0.4) | 224.6 (46.1 to 809.7) | 186 (10.7 to 782.1) | 476.2 (102.4 to 2622.9) |
|  |  |  | All ages | 0.4 (0.1 to 0.8) | 0.4 (0.1 to 0.7) | 0.1 (0 to 0.2) | 6.5 (2.7 to 11.4) | 4.8 (2 to 8.7) | 1.7 (0.6 to 3.1) | 1385.6 (636.3 to 2934) | 1218.4 (481.8 to 2916.8) | 2196.3 (850.1 to 6898) |
|  | High systolic blood pressure | DALYs (Disability-Adjusted Life Years) | Age-standardized | 18.6 (6.1 to 32.3) | 22.2 (7.1 to 39.8) | 15.1 (4.6 to 26) | 19.3 (6.3 to 34) | 22.9 (7.5 to 39.8) | 15.9 (5.3 to 28) | 3.8 (-21.7 to 31.6) | 3.3 (-31.2 to 49.6) | 4.9 (-23 to 47.1) |
|  |  |  | All ages | 77.3 (25.1 to 133.5) | 42.3 (13.3 to 76) | 35 (11.1 to 61.2) | 300.5 (99.3 to 535.6) | 178 (57.9 to 311.8) | 122.6 (41 to 216.1) | 288.8 (203.5 to 393.8) | 320.9 (179.6 to 508.1) | 250 (151.5 to 374.8) |
|  |  | Deaths | Age-standardized | 0.9 (0.3 to 1.5) | 1.2 (0.4 to 2.3) | 0.5 (0.1 to 1) | 0.8 (0.3 to 1.4) | 1.2 (0.4 to 2.2) | 0.4 (0.1 to 0.8) | -6.7 (-38.1 to 39.7) | -0.5 (-41.9 to 69.5) | -16.8 (-50.7 to 50.8) |
|  |  |  | All ages | 2.7 (0.8 to 4.7) | 1.8 (0.5 to 3.3) | 0.9 (0.3 to 1.7) | 13.1 (4.5 to 23.4) | 9.3 (3 to 16.9) | 3.8 (1.2 to 6.9) | 385.4 (228.3 to 629.9) | 426.3 (204.3 to 776.9) | 307 (138.5 to 637.3) |
|  | Lead exposure | DALYs (Disability-Adjusted Life Years) | Age-standardized | 4.1 (-0.6 to 10.1) | 4.5 (-0.6 to 11.5) | 3.8 (-0.6 to 9.4) | 4 (-0.5 to 10) | 4.4 (-0.6 to 10.6) | 3.8 (-0.5 to 9.4) | -1.9 (-20.4 to 18) | -2.5 (-26.7 to 29.1) | 0.3 (-22.1 to 26.8) |
|  |  |  | All ages | 17.1 (-2.4 to 42.7) | 8.5 (-1.2 to 22.1) | 8.6 (-1.3 to 22.1) | 63.4 (-8.6 to 156.1) | 33.6 (-4.5 to 81.8) | 29.8 (-4.2 to 73) | 270.7 (203 to 342.1) | 296.6 (203 to 419.3) | 245.1 (164.4 to 339.2) |
|  |  | Deaths | Age-standardized | 0.2 (0 to 0.5) | 0.2 (0 to 0.6) | 0.1 (0 to 0.4) | 0.2 (0 to 0.4) | 0.3 (0 to 0.6) | 0.1 (0 to 0.3) | -5.1 (-30.8 to 31.8) | 1.7 (-32.6 to 50.6) | -12.8 (-40 to 43.3) |
|  |  |  | All ages | 0.6 (-0.1 to 1.5) | 0.4 (-0.1 to 0.9) | 0.2 (0 to 0.6) | 3 (-0.4 to 7.1) | 1.9 (-0.3 to 4.6) | 1 (-0.2 to 2.6) | 399.4 (267.4 to 580.2) | 436.3 (257.5 to 677.4) | 343.2 (200 to 619.4) |
|  | Smoking | DALYs (Disability-Adjusted Life Years) | Age-standardized | 1.7 (0.9 to 2.7) | 0.6 (0.3 to 1.1) | 2.8 (1.5 to 4.4) | 1.8 (1 to 2.9) | 0.6 (0.3 to 1.1) | 3.1 (1.7 to 4.9) | 7.5 (-19 to 39.5) | 3.8 (-44 to 78.1) | 11.4 (-16.7 to 52.1) |
|  |  |  | All ages | 10 (5.4 to 15.4) | 1.7 (0.9 to 3.1) | 8.2 (4.3 to 12.6) | 29.7 (16.3 to 46.9) | 5.1 (2.5 to 8.9) | 24.5 (13.5 to 38.5) | 197.3 (124.1 to 279.9) | 194.1 (64 to 397.8) | 197.9 (121.9 to 303.8) |
|  |  | Deaths | Age-standardized | 0 (0 to 0.1) | 0 (0 to 0) | 0.1 (0 to 0.1) | 0 (0 to 0.1) | 0 (0 to 0) | 0.1 (0 to 0.1) | -7.9 (-45.1 to 52.6) | -0.8 (-58.9 to 138.4) | -9.3 (-47.1 to 72.8) |
|  |  |  | All ages | 0.2 (0.1 to 0.3) | 0 (0 to 0.1) | 0.1 (0.1 to 0.2) | 0.7 (0.3 to 1.1) | 0.2 (0.1 to 0.4) | 0.5 (0.2 to 0.8) | 256 (112 to 478.9) | 291.9 (64.5 to 780.1) | 245.3 (98 to 531.6) |
| Mazandaran | All risk factors | DALYs (Disability-Adjusted Life Years) | Age-standardized | 22.9 (10.1 to 36.7) | 26 (11 to 42.8) | 18.9 (8.3 to 31.5) | 30.3 (15.9 to 46.2) | 35.2 (17.8 to 54.6) | 25.5 (12.8 to 39.2) | 32.4 (0.3 to 86.7) | 35.2 (-8 to 103.2) | 34.6 (5.2 to 88.1) |
|  |  |  | All ages | 189.5 (84.5 to 304.4) | 107.5 (45.5 to 176) | 82 (36.1 to 133.3) | 1034.8 (545.5 to 1574) | 595.1 (305.2 to 924.3) | 439.7 (225.4 to 674.1) | 446.1 (320.4 to 640.8) | 453.5 (295.5 to 690.9) | 436.5 (322.4 to 637.2) |
|  |  | Deaths | Age-standardized | 1 (0.4 to 1.7) | 1.3 (0.5 to 2.2) | 0.6 (0.2 to 1.1) | 1.2 (0.6 to 1.9) | 1.8 (0.8 to 2.8) | 0.8 (0.4 to 1.2) | 25.6 (-17.4 to 98.5) | 36 (-21.9 to 124.5) | 23.8 (-19.5 to 133.6) |
|  |  |  | All ages | 6 (2.5 to 9.7) | 4.2 (1.8 to 7.3) | 1.7 (0.6 to 3) | 39 (20.1 to 60.6) | 27.2 (13.1 to 43.9) | 11.8 (5.8 to 18.8) | 554.1 (333.2 to 921.7) | 540.7 (268.5 to 944.4) | 587.3 (348.2 to 1149.3) |
|  | Diet high in sodium | DALYs (Disability-Adjusted Life Years) | Age-standardized | 0.4 (0 to 2.3) | 0.3 (0 to 2.1) | 0.5 (0 to 2.7) | 0.4 (0 to 2.4) | 0.3 (0 to 2.2) | 0.5 (0 to 2.9) | 5.4 (-94.7 to 1407.1) | 0.6 (-913711.9 to 20104.4) | 6.6 (-94.9 to 1519.1) |
|  |  |  | All ages | 3.5 (0 to 21.1) | 1.2 (0 to 8.9) | 2.3 (0 to 12) | 13.7 (0 to 83.3) | 4.7 (0 to 36.8) | 9 (0 to 50.9) | 295.1 (-83.5 to 3772.2) | 290.8 (-2000254.6 to 163614.5) | 297.3 (-83.6 to 4211) |
|  |  | Deaths | Age-standardized | 0 (0 to 0.1) | 0 (0 to 0.1) | 0 (0 to 0.1) | 0 (0 to 0.1) | 0 (0 to 0.1) | 0 (0 to 0.1) | -0.1 (-96.8 to 2919) | 0.2 (-199628.9 to 71072.9) | -2.1 (-96.5 to 3023.2) |
|  |  |  | All ages | 0.1 (0 to 0.5) | 0 (0 to 0.3) | 0 (0 to 0.3) | 0.4 (0 to 2.9) | 0.2 (0 to 1.6) | 0.2 (0 to 1.4) | 370.3 (-88.3 to 5846.6) | 344.4 (-438480.1 to 135549.6) | 395.1 (-88.9 to 5855.3) |
|  | High alcohol use | DALYs (Disability-Adjusted Life Years) | Age-standardized | 0 (0 to 0) | 0 (0 to 0) | 0 (0 to 0) | 0.2 (0.1 to 0.3) | 0 (0 to 0.1) | 0.4 (0.2 to 0.6) | NA | NA | NA |
|  |  |  | All ages | 0 (0 to 0) | 0 (0 to 0) | 0 (0 to 0) | 7.6 (4.3 to 11.8) | 0.7 (0.2 to 1.2) | 7 (4 to 10.6) | NA | NA | NA |
|  |  | Deaths | Age-standardized | 0 (0 to 0) | 0 (0 to 0) | 0 (0 to 0) | 0 (0 to 0) | 0 (0 to 0) | 0 (0 to 0) | NA | NA | NA |
|  |  |  | All ages | 0 (0 to 0) | 0 (0 to 0) | 0 (0 to 0) | 0.2 (0.1 to 0.3) | 0 (0 to 0.1) | 0.1 (0.1 to 0.2) | NA | NA | NA |
|  | High body-mass index | DALYs (Disability-Adjusted Life Years) | Age-standardized | 3.7 (1.4 to 6.7) | 5.4 (2.1 to 10.1) | 1.7 (0.6 to 3.5) | 11.9 (4.8 to 20.2) | 15.3 (6.1 to 26.5) | 8.4 (3.3 to 15) | 219.8 (74.5 to 448.1) | 181.7 (42.5 to 396.2) | 385 (167.9 to 1064.8) |
|  |  |  | All ages | 34.3 (13.2 to 60) | 25.2 (9.8 to 45) | 9.1 (3.3 to 17.3) | 408.7 (165.4 to 698.8) | 263.6 (106.2 to 455.2) | 145.1 (56.8 to 257.7) | 1091.2 (587.8 to 1677.6) | 944.4 (470.8 to 1555) | 1499.3 (795 to 3089.9) |
|  |  | Deaths | Age-standardized | 0.2 (0.1 to 0.3) | 0.2 (0.1 to 0.5) | 0 (0 to 0.1) | 0.5 (0.2 to 0.9) | 0.7 (0.3 to 1.4) | 0.3 (0.1 to 0.5) | 228.9 (46.4 to 711.6) | 214.6 (22.6 to 708.8) | 475.8 (140.3 to 2290.1) |
|  |  |  | All ages | 1 (0.4 to 1.8) | 0.8 (0.3 to 1.6) | 0.2 (0 to 0.3) | 15.6 (6.4 to 27.6) | 11.5 (4.5 to 20.9) | 4 (1.4 to 7.6) | 1462.4 (655.9 to 3006.5) | 1266.8 (476.7 to 2842.4) | 2538.7 (1163.1 to 7055.2) |
|  | High systolic blood pressure | DALYs (Disability-Adjusted Life Years) | Age-standardized | 19.9 (6.5 to 34.3) | 22.9 (7.4 to 39) | 16.1 (5.2 to 28.4) | 23.1 (7 to 38.6) | 27.2 (8.9 to 46.4) | 18.9 (6.1 to 33.5) | 16 (-11.3 to 51.9) | 18.7 (-17.6 to 71.6) | 17.9 (-11 to 56.5) |
|  |  |  | All ages | 159 (54.2 to 275.5) | 92.7 (30.5 to 160.9) | 66.3 (21.4 to 118.8) | 779.8 (236.9 to 1308.7) | 457.1 (146.3 to 781.7) | 322.7 (103.2 to 569.6) | 390.3 (282.4 to 533.9) | 393.1 (245 to 601.1) | 386.5 (269 to 553.6) |
|  |  | Deaths | Age-standardized | 0.9 (0.3 to 1.6) | 1.2 (0.4 to 2.1) | 0.5 (0.2 to 1) | 1 (0.3 to 1.7) | 1.4 (0.4 to 2.5) | 0.6 (0.2 to 1) | 8.5 (-29.3 to 62) | 18.2 (-30 to 98.7) | 5.7 (-31.2 to 93.3) |
|  |  |  | All ages | 5.2 (1.8 to 9.1) | 3.8 (1.2 to 6.8) | 1.5 (0.4 to 2.8) | 30.1 (10 to 52.8) | 21.2 (7 to 38.2) | 8.9 (2.9 to 15.7) | 474.3 (287.3 to 757.4) | 463.1 (238.7 to 845.9) | 502.8 (288.5 to 963.6) |
|  | Lead exposure | DALYs (Disability-Adjusted Life Years) | Age-standardized | 3.4 (-0.5 to 8.9) | 3.6 (-0.5 to 9.4) | 3.2 (-0.5 to 8.2) | 3.6 (-0.5 to 9.3) | 3.7 (-0.5 to 9.6) | 3.5 (-0.5 to 8.9) | 5.5 (-18.6 to 36.8) | 3.5 (-29.2 to 45.8) | 10.7 (-12.6 to 43.1) |
|  |  |  | All ages | 27.7 (-3.7 to 71.5) | 14.6 (-2 to 37.8) | 13.1 (-1.8 to 34.7) | 119.8 (-16.4 to 306) | 61.2 (-8.9 to 157.6) | 58.6 (-7.7 to 148.9) | 332.8 (245.7 to 453.3) | 319.3 (184.8 to 481.8) | 347.9 (259.2 to 471.6) |
|  |  | Deaths | Age-standardized | 0.2 (0 to 0.4) | 0.2 (0 to 0.5) | 0.1 (0 to 0.3) | 0.2 (0 to 0.4) | 0.2 (0 to 0.5) | 0.1 (0 to 0.3) | 6 (-27.8 to 54.2) | 11.4 (-32.3 to 72.2) | 8.1 (-25.2 to 81.6) |
|  |  |  | All ages | 0.9 (-0.1 to 2.3) | 0.6 (-0.1 to 1.5) | 0.3 (0 to 0.8) | 5 (-0.8 to 12.7) | 3.2 (-0.5 to 8) | 1.8 (-0.3 to 4.5) | 455.6 (278.6 to 708.9) | 424.8 (215.7 to 711.8) | 517.9 (320.1 to 907.5) |
|  | Smoking | DALYs (Disability-Adjusted Life Years) | Age-standardized | 1.7 (0.9 to 2.7) | 0.6 (0.3 to 1.1) | 2.8 (1.5 to 4.6) | 2 (1.1 to 3.1) | 0.7 (0.3 to 1.2) | 3.4 (1.8 to 5.3) | 19.4 (-7.3 to 56.3) | 10.5 (-36.7 to 92.6) | 19.3 (-8.5 to 59.5) |
|  |  |  | All ages | 19.3 (10.6 to 30.9) | 3.5 (1.8 to 6.3) | 15.8 (8.6 to 25.1) | 78 (43.2 to 120.9) | 13.7 (6.9 to 23.7) | 64.2 (35.7 to 101.4) | 303 (212.7 to 417.6) | 289.2 (127.6 to 552.4) | 306.1 (207.6 to 433.2) |
|  |  | Deaths | Age-standardized | 0 (0 to 0.1) | 0 (0 to 0) | 0.1 (0 to 0.1) | 0 (0 to 0.1) | 0 (0 to 0) | 0.1 (0 to 0.1) | 16 (-26 to 103.9) | 9.2 (-57.1 to 164.3) | 10.1 (-34.7 to 105.7) |
|  |  |  | All ages | 0.3 (0.2 to 0.5) | 0.1 (0 to 0.2) | 0.2 (0.1 to 0.4) | 1.5 (0.8 to 2.5) | 0.4 (0.2 to 0.8) | 1.2 (0.6 to 1.9) | 388 (207.4 to 734.5) | 347.2 (102.7 to 888.1) | 403.1 (194.1 to 835.5) |
| North Khorasan | All risk factors | DALYs (Disability-Adjusted Life Years) | Age-standardized | 21.8 (9.5 to 35.7) | 25.7 (10.8 to 43.4) | 17.8 (7.3 to 29.7) | 29.9 (15.5 to 45) | 36.4 (18.7 to 56.1) | 24.4 (12.2 to 37.7) | 37.3 (6 to 93.3) | 41.6 (-3.4 to 108.9) | 37.6 (5 to 104.3) |
|  |  |  | All ages | 38.6 (17.2 to 62.2) | 20.7 (8.7 to 34.5) | 17.9 (8 to 29.7) | 195 (103.5 to 293.3) | 111.3 (58.5 to 170.3) | 83.8 (42.8 to 128) | 405.7 (297.1 to 587.3) | 438.3 (271.9 to 696.7) | 368 (254.5 to 560.1) |
|  |  | Deaths | Age-standardized | 1 (0.4 to 1.7) | 1.3 (0.5 to 2.4) | 0.6 (0.2 to 1.1) | 1.2 (0.6 to 1.9) | 1.8 (0.9 to 2.9) | 0.7 (0.3 to 1.1) | 26.8 (-18.4 to 105.1) | 41.7 (-19.4 to 141) | 20.2 (-22.7 to 134.6) |
|  |  |  | All ages | 1.1 (0.5 to 1.9) | 0.7 (0.3 to 1.3) | 0.4 (0.1 to 0.7) | 7.4 (3.8 to 11.5) | 5 (2.4 to 7.9) | 2.4 (1.1 to 3.7) | 573 (350.3 to 964.8) | 585.2 (298.7 to 1036.3) | 549.3 (308.2 to 1116.9) |
|  | Diet high in sodium | DALYs (Disability-Adjusted Life Years) | Age-standardized | 0.4 (0 to 2.3) | 0.3 (0 to 2.1) | 0.5 (0 to 2.7) | 0.4 (0 to 2.4) | 0.3 (0 to 2.3) | 0.5 (0 to 2.7) | 3.6 (-95.7 to 1098.5) | 5 (-233.7 to 9553.5) | 3.6 (-95.6 to 1323.2) |
|  |  |  | All ages | 0.8 (0 to 4.6) | 0.3 (0 to 1.7) | 0.5 (0 to 2.8) | 2.7 (0 to 16.1) | 0.9 (0 to 7.2) | 1.7 (0 to 9.3) | 233.4 (-85.8 to 2632.9) | 267.7 (-563.7 to 10606.3) | 217.5 (-86.7 to 2866.7) |
|  |  | Deaths | Age-standardized | 0 (0 to 0.1) | 0 (0 to 0.1) | 0 (0 to 0.1) | 0 (0 to 0.1) | 0 (0 to 0.1) | 0 (0 to 0.1) | -2.4 (-97.8 to 1385.6) | 5.9 (-245.6 to 12772.6) | -9.9 (-98.1 to 1573.5) |
|  |  |  | All ages | 0 (0 to 0.1) | 0 (0 to 0.1) | 0 (0 to 0.1) | 0.1 (0 to 0.6) | 0 (0 to 0.3) | 0 (0 to 0.3) | 331.5 (-92.2 to 2880.7) | 354 (-928.5 to 22216.3) | 314.9 (-92.3 to 3520.6) |
|  | High alcohol use | DALYs (Disability-Adjusted Life Years) | Age-standardized | 0 (0 to 0) | 0 (0 to 0) | 0 (0 to 0) | 0.2 (0.1 to 0.3) | 0 (0 to 0.1) | 0.3 (0.2 to 0.5) | NA | NA | NA |
|  |  |  | All ages | 0 (0 to 0) | 0 (0 to 0) | 0 (0 to 0) | 1.3 (0.7 to 2) | 0.1 (0 to 0.2) | 1.2 (0.7 to 1.8) | NA | NA | NA |
|  |  | Deaths | Age-standardized | 0 (0 to 0) | 0 (0 to 0) | 0 (0 to 0) | 0 (0 to 0) | 0 (0 to 0) | 0 (0 to 0) | NA | NA | NA |
|  |  |  | All ages | 0 (0 to 0) | 0 (0 to 0) | 0 (0 to 0) | 0 (0 to 0) | 0 (0 to 0) | 0 (0 to 0) | NA | NA | NA |
|  | High body-mass index | DALYs (Disability-Adjusted Life Years) | Age-standardized | 2.9 (1.1 to 5.4) | 5.1 (1.9 to 9.6) | 0.8 (0.2 to 1.7) | 10.2 (4.2 to 17.7) | 14.1 (6.2 to 24.6) | 6.8 (2.6 to 11.9) | 250.4 (92.5 to 538.4) | 175.1 (37.1 to 377.1) | 735 (318.5 to 2940.1) |
|  |  |  | All ages | 6.2 (2.4 to 10.5) | 5 (2 to 8.8) | 1.2 (0.3 to 2.4) | 67.7 (28.2 to 116.5) | 44.2 (19.2 to 77.1) | 23.5 (8.9 to 41.8) | 993 (585.3 to 1618.5) | 780.1 (405.4 to 1320.5) | 1902.5 (972 to 6113.5) |
|  |  | Deaths | Age-standardized | 0.1 (0 to 0.3) | 0.2 (0.1 to 0.5) | 0 (0 to 0.1) | 0.4 (0.2 to 0.7) | 0.7 (0.3 to 1.2) | 0.2 (0.1 to 0.4) | 246 (46.1 to 769) | 210.7 (18 to 673) | 998.4 (271.7 to 6392.1) |
|  |  |  | All ages | 0.2 (0.1 to 0.3) | 0.1 (0.1 to 0.3) | 0 (0 to 0) | 2.6 (1.1 to 4.5) | 1.9 (0.8 to 3.3) | 0.7 (0.3 to 1.3) | 1437.4 (694.2 to 2680) | 1160.4 (464.2 to 2326.4) | 3622.2 (1521 to 13170.3) |
|  | High systolic blood pressure | DALYs (Disability-Adjusted Life Years) | Age-standardized | 19 (6.3 to 33.3) | 22.7 (7.7 to 41.1) | 15.2 (4.5 to 27.6) | 23.6 (8.1 to 40.1) | 29.1 (10.5 to 49.7) | 18.9 (6 to 31.8) | 24.5 (-5.3 to 65) | 28.6 (-14.1 to 91.7) | 24.5 (-7.7 to 74.1) |
|  |  |  | All ages | 31.8 (10.4 to 56.2) | 17.5 (5.7 to 31.9) | 14.3 (4.2 to 25.9) | 151.9 (52.2 to 257.3) | 88.2 (31.5 to 150.2) | 63.7 (20.3 to 107.4) | 377.4 (270.8 to 545.1) | 403.4 (251.1 to 669.5) | 345.6 (234.3 to 524.5) |
|  |  | Deaths | Age-standardized | 0.9 (0.3 to 1.6) | 1.2 (0.4 to 2.2) | 0.5 (0.1 to 1) | 1 (0.3 to 1.7) | 1.5 (0.5 to 2.5) | 0.6 (0.2 to 1) | 13.4 (-26.9 to 75) | 27.4 (-25.8 to 111.8) | 6.4 (-32.8 to 103.4) |
|  |  |  | All ages | 1 (0.3 to 1.8) | 0.6 (0.2 to 1.2) | 0.3 (0.1 to 0.6) | 6 (2.1 to 10.2) | 4 (1.4 to 7) | 1.9 (0.6 to 3.3) | 521.6 (306.9 to 846.6) | 531.3 (266.2 to 966.4) | 502.3 (275 to 1002.5) |
|  | Lead exposure | DALYs (Disability-Adjusted Life Years) | Age-standardized | 4.7 (-0.7 to 11.9) | 5.1 (-0.7 to 13) | 4.3 (-0.6 to 11.2) | 4.8 (-0.7 to 12) | 5.3 (-0.7 to 13.2) | 4.5 (-0.6 to 11.3) | 2.5 (-18.3 to 32.7) | 3.8 (-29.1 to 43) | 4.5 (-16.1 to 42.7) |
|  |  |  | All ages | 8.1 (-1.1 to 19.6) | 4 (-0.5 to 10.3) | 4.1 (-0.5 to 10.5) | 31 (-4.2 to 77.6) | 15.6 (-2.2 to 39.3) | 15.4 (-2.1 to 38.8) | 285.2 (210.2 to 395.1) | 292.2 (167.9 to 436.1) | 278.3 (201.4 to 406.2) |
|  |  | Deaths | Age-standardized | 0.2 (0 to 0.5) | 0.3 (0 to 0.7) | 0.2 (0 to 0.4) | 0.2 (0 to 0.5) | 0.3 (0 to 0.7) | 0.1 (0 to 0.4) | -1.6 (-32 to 47.3) | 10 (-34 to 68.7) | -6.8 (-34.4 to 74.4) |
|  |  |  | All ages | 0.2 (0 to 0.6) | 0.1 (0 to 0.4) | 0.1 (0 to 0.2) | 1.3 (-0.2 to 3.1) | 0.8 (-0.1 to 2) | 0.5 (-0.1 to 1.3) | 440.5 (280.7 to 705.2) | 434.1 (221.1 to 723.8) | 450.5 (276.2 to 916.3) |
|  | Smoking | DALYs (Disability-Adjusted Life Years) | Age-standardized | 1.8 (1 to 2.9) | 0.7 (0.3 to 1.2) | 2.9 (1.6 to 4.7) | 1.9 (1 to 3) | 0.7 (0.3 to 1.2) | 3.1 (1.7 to 5) | 2.9 (-22.7 to 38.8) | 2.2 (-45.1 to 82) | 4.8 (-22.4 to 45.1) |
|  |  |  | All ages | 4.9 (2.7 to 7.7) | 0.9 (0.4 to 1.4) | 4 (2.2 to 6.4) | 14 (7.6 to 22) | 2.5 (1.3 to 4.3) | 11.4 (6.3 to 18.2) | 186.4 (121.2 to 281.5) | 198.8 (60.3 to 445.2) | 183.7 (112.8 to 285.1) |
|  |  | Deaths | Age-standardized | 0 (0 to 0.1) | 0 (0 to 0) | 0.1 (0 to 0.1) | 0 (0 to 0.1) | 0 (0 to 0) | 0.1 (0 to 0.1) | -3.3 (-40.9 to 77.9) | 1.5 (-60.5 to 153.7) | -9.4 (-46.3 to 83) |
|  |  |  | All ages | 0.1 (0 to 0.1) | 0 (0 to 0) | 0.1 (0 to 0.1) | 0.3 (0.2 to 0.5) | 0.1 (0 to 0.1) | 0.2 (0.1 to 0.4) | 257.3 (114.9 to 533) | 265 (59.4 to 699.9) | 254.9 (97.2 to 597.9) |
| Qazvin | All risk factors | DALYs (Disability-Adjusted Life Years) | Age-standardized | 19.5 (8.4 to 32.5) | 21.2 (8.8 to 36.5) | 17 (6.9 to 28.2) | 25 (12.9 to 39.1) | 25.9 (12.7 to 42.5) | 23.4 (12.5 to 37.3) | 28.2 (-4.2 to 96.8) | 22.3 (-17.8 to 91.2) | 38.1 (3.8 to 118) |
|  |  |  | All ages | 58.3 (26 to 95.3) | 31.9 (13.1 to 53.3) | 26.4 (11.1 to 44) | 249.1 (130.5 to 387.3) | 135.1 (67 to 222.9) | 113.9 (62.3 to 179) | 327.1 (230.8 to 518.3) | 323 (199 to 533.3) | 332.1 (228.5 to 542.3) |
|  |  | Deaths | Age-standardized | 0.9 (0.3 to 1.6) | 1.1 (0.4 to 2) | 0.6 (0.2 to 1.2) | 1.1 (0.6 to 1.8) | 1.3 (0.6 to 2.2) | 0.9 (0.4 to 1.4) | 26.3 (-19.9 to 116.2) | 24.2 (-27.5 to 124.2) | 36.1 (-13.3 to 191.5) |
|  |  |  | All ages | 1.9 (0.7 to 3.3) | 1.3 (0.5 to 2.4) | 0.6 (0.2 to 1.1) | 9.6 (4.8 to 15.4) | 6.2 (2.7 to 10.4) | 3.4 (1.6 to 5.5) | 399.5 (231.3 to 770.5) | 372.5 (181.5 to 710.2) | 458.7 (260.3 to 1043.5) |
|  | Diet high in sodium | DALYs (Disability-Adjusted Life Years) | Age-standardized | 0.4 (0 to 2.1) | 0.2 (0 to 1.7) | 0.5 (0 to 2.8) | 0.4 (0 to 2.5) | 0.2 (0 to 1.9) | 0.5 (0 to 3.2) | 5 (-97.1 to 1429.8) | -1.8 (-997.4 to 72463.6) | 8.4 (-97.1 to 1507.1) |
|  |  |  | All ages | 1.3 (0 to 7.1) | 0.4 (0 to 2.8) | 0.8 (0 to 4.4) | 3.9 (0 to 24.6) | 1.3 (0 to 10.1) | 2.6 (0 to 14.8) | 213.6 (-90.6 to 4590.3) | 215.6 (-1856.9 to 317873.2) | 212.7 (-90.9 to 4613.2) |
|  |  | Deaths | Age-standardized | 0 (0 to 0.1) | 0 (0 to 0.1) | 0 (0 to 0.1) | 0 (0 to 0.1) | 0 (0 to 0.1) | 0 (0 to 0.1) | 7.8 (-98.9 to 1457.5) | 0.6 (-779.3 to 127372.4) | 8.7 (-98.8 to 1508.6) |
|  |  |  | All ages | 0 (0 to 0.2) | 0 (0 to 0.1) | 0 (0 to 0.1) | 0.1 (0 to 0.9) | 0.1 (0 to 0.4) | 0.1 (0 to 0.5) | 279.8 (-95.5 to 4269.2) | 257.4 (-3406.8 to 251850.4) | 297.8 (-95.5 to 4459.6) |
|  | High alcohol use | DALYs (Disability-Adjusted Life Years) | Age-standardized | 0 (0 to 0) | 0 (0 to 0) | 0 (0 to 0) | 0.2 (0.1 to 0.3) | 0 (0 to 0.1) | 0.4 (0.2 to 0.6) | NA | NA | NA |
|  |  |  | All ages | 0 (0 to 0) | 0 (0 to 0) | 0 (0 to 0) | 2.1 (1.2 to 3.3) | 0.2 (0 to 0.3) | 2 (1.1 to 3.1) | NA | NA | NA |
|  |  | Deaths | Age-standardized | 0 (0 to 0) | 0 (0 to 0) | 0 (0 to 0) | 0 (0 to 0) | 0 (0 to 0) | 0 (0 to 0) | NA | NA | NA |
|  |  |  | All ages | 0 (0 to 0) | 0 (0 to 0) | 0 (0 to 0) | 0 (0 to 0.1) | 0 (0 to 0) | 0 (0 to 0.1) | NA | NA | NA |
|  | High body-mass index | DALYs (Disability-Adjusted Life Years) | Age-standardized | 3 (1.1 to 5.5) | 4.6 (1.7 to 8.3) | 1.2 (0.4 to 2.6) | 9.9 (4 to 17.4) | 11.8 (4.7 to 21.4) | 7.6 (2.9 to 13.9) | 228.6 (76.7 to 481.7) | 158.8 (30.3 to 354.9) | 517.2 (208.4 to 1600.6) |
|  |  |  | All ages | 10.2 (3.8 to 17.9) | 7.7 (2.9 to 13.7) | 2.5 (0.9 to 4.8) | 98.7 (40.1 to 171) | 62.1 (25.1 to 111.5) | 36.6 (14.7 to 66.5) | 866.6 (479.6 to 1381.5) | 702.9 (344.2 to 1171.2) | 1377.1 (752.4 to 3342.3) |
|  |  | Deaths | Age-standardized | 0.1 (0 to 0.3) | 0.2 (0.1 to 0.4) | 0 (0 to 0.1) | 0.4 (0.2 to 0.8) | 0.6 (0.2 to 1.1) | 0.3 (0.1 to 0.6) | 259.2 (56.7 to 819.4) | 199.5 (21.9 to 638.7) | 760.8 (188.4 to 4086.9) |
|  |  |  | All ages | 0.3 (0.1 to 0.6) | 0.3 (0.1 to 0.5) | 0 (0 to 0.1) | 3.8 (1.6 to 7) | 2.7 (1.1 to 5.1) | 1.1 (0.4 to 2.1) | 1159.1 (518.2 to 2440.3) | 939.8 (358.6 to 2113.1) | 2447.7 (1013.1 to 7374.7) |
|  | High systolic blood pressure | DALYs (Disability-Adjusted Life Years) | Age-standardized | 16.7 (5.3 to 29.7) | 18.4 (5.9 to 34.3) | 14.3 (4.2 to 25.8) | 18.1 (5.8 to 32.5) | 19.2 (6 to 36) | 16.6 (5 to 30.1) | 8.6 (-21.2 to 50.2) | 4 (-30.8 to 55.2) | 16.7 (-16.1 to 72.2) |
|  |  |  | All ages | 48 (14.9 to 85.9) | 27.1 (8.2 to 47.9) | 21 (6.4 to 39.1) | 178.1 (57.9 to 319.3) | 99 (30.8 to 184.6) | 79.1 (23.8 to 142.5) | 270.7 (181.9 to 386.6) | 265.7 (144.1 to 447.9) | 277.1 (184.2 to 442.3) |
|  |  | Deaths | Age-standardized | 0.8 (0.2 to 1.5) | 0.9 (0.3 to 1.8) | 0.6 (0.1 to 1.1) | 0.8 (0.3 to 1.5) | 1 (0.3 to 1.9) | 0.6 (0.2 to 1.2) | 5.5 (-34.9 to 71.3) | 4.8 (-41.4 to 86.4) | 11.7 (-33.1 to 122.3) |
|  |  |  | All ages | 1.7 (0.5 to 3.2) | 1.2 (0.4 to 2.3) | 0.5 (0.1 to 1) | 7.1 (2.4 to 13.2) | 4.7 (1.5 to 8.8) | 2.4 (0.7 to 4.6) | 326 (168 to 565.4) | 303.9 (124.1 to 609.7) | 376.3 (197.1 to 845.4) |
|  | Lead exposure | DALYs (Disability-Adjusted Life Years) | Age-standardized | 4 (-0.5 to 10.1) | 4.1 (-0.6 to 10.3) | 3.9 (-0.5 to 10) | 4.4 (-0.6 to 11.1) | 4.2 (-0.6 to 10.6) | 4.5 (-0.6 to 11.1) | 9.3 (-12.9 to 39.1) | 3 (-24.8 to 35.3) | 16.5 (-7.5 to 61.9) |
|  |  |  | All ages | 11.6 (-1.5 to 29.8) | 6 (-0.8 to 15.2) | 5.6 (-0.7 to 14.3) | 41.3 (-5.6 to 104.9) | 21 (-2.9 to 53.8) | 20.3 (-2.8 to 49.7) | 255.4 (189.2 to 348.9) | 250.1 (157.7 to 359.7) | 261.1 (192.5 to 383.4) |
|  |  | Deaths | Age-standardized | 0.2 (0 to 0.5) | 0.2 (0 to 0.5) | 0.2 (0 to 0.4) | 0.2 (0 to 0.5) | 0.2 (0 to 0.6) | 0.2 (0 to 0.5) | 16.8 (-18.3 to 74) | 13.8 (-28.4 to 69.4) | 22.1 (-17.1 to 134.1) |
|  |  |  | All ages | 0.4 (-0.1 to 1) | 0.3 (0 to 0.6) | 0.1 (0 to 0.4) | 1.8 (-0.3 to 4.5) | 1.1 (-0.2 to 2.9) | 0.7 (-0.1 to 1.8) | 357.7 (219.2 to 586.8) | 330.2 (166.1 to 539.7) | 407.7 (247.4 to 860.3) |
|  | Smoking | DALYs (Disability-Adjusted Life Years) | Age-standardized | 1.6 (0.9 to 2.6) | 0.6 (0.3 to 1) | 2.8 (1.5 to 4.4) | 2.1 (1.1 to 3.3) | 0.6 (0.3 to 1.1) | 3.6 (1.9 to 5.8) | 25.9 (-2.5 to 69.9) | 11.5 (-37.4 to 88.8) | 30.1 (-2.6 to 81.3) |
|  |  |  | All ages | 6.6 (3.6 to 10.5) | 1.1 (0.5 to 1.9) | 5.5 (3 to 8.9) | 24.3 (13.4 to 38.9) | 3.9 (1.9 to 6.7) | 20.4 (10.9 to 32.5) | 267.2 (180.6 to 382) | 244.7 (96.5 to 470.9) | 271.8 (178.2 to 400) |
|  |  | Deaths | Age-standardized | 0 (0 to 0.1) | 0 (0 to 0) | 0.1 (0 to 0.1) | 0.1 (0 to 0.1) | 0 (0 to 0) | 0.1 (0 to 0.2) | 31.7 (-17.6 to 150) | 11.3 (-54.1 to 166.4) | 31.3 (-23.6 to 168.8) |
|  |  |  | All ages | 0.1 (0.1 to 0.2) | 0 (0 to 0.1) | 0.1 (0 to 0.1) | 0.5 (0.3 to 0.9) | 0.1 (0 to 0.2) | 0.4 (0.2 to 0.7) | 333.2 (177.6 to 665.8) | 274.9 (70.4 to 716.9) | 351.4 (180.7 to 769.4) |
| Qom | All risk factors | DALYs (Disability-Adjusted Life Years) | Age-standardized | 22.2 (9.6 to 35.1) | 27.7 (11.2 to 46.6) | 17.5 (7.3 to 29.5) | 25.7 (13.6 to 40) | 34.7 (17 to 55) | 20.5 (9.8 to 31.8) | 16 (-14.4 to 68.5) | 25.2 (-16.6 to 91.2) | 17.3 (-12.5 to 72.5) |
|  |  |  | All ages | 44.6 (19.8 to 71.3) | 24.4 (10.6 to 39.3) | 20.2 (8.6 to 33.3) | 236 (123.7 to 366.2) | 132.1 (67.7 to 204.7) | 103.9 (50.7 to 161.9) | 428.8 (300.2 to 644.2) | 441.5 (279.2 to 711.3) | 413.5 (291.9 to 626.6) |
|  |  | Deaths | Age-standardized | 1.1 (0.4 to 1.9) | 1.7 (0.6 to 3.1) | 0.6 (0.2 to 1.2) | 1.2 (0.6 to 1.9) | 2.2 (1 to 3.6) | 0.6 (0.3 to 1) | 4.1 (-31.7 to 65.6) | 27.1 (-24.9 to 120.6) | -3.5 (-36.7 to 70.8) |
|  |  |  | All ages | 1.6 (0.7 to 2.8) | 1.1 (0.4 to 1.9) | 0.5 (0.2 to 1) | 9.3 (4.6 to 14.7) | 6.4 (3 to 10.7) | 2.8 (1.3 to 4.6) | 462.2 (272.6 to 782.7) | 482.2 (253.1 to 864.5) | 421.5 (238.5 to 816.6) |
|  | Diet high in sodium | DALYs (Disability-Adjusted Life Years) | Age-standardized | 0.4 (0 to 2.5) | 0.3 (0 to 2.4) | 0.5 (0 to 2.6) | 0.4 (0 to 2.3) | 0.3 (0 to 2.3) | 0.5 (0 to 2.5) | -5.7 (-96.6 to 2938.4) | -2.1 (-376 to 248291) | -6.2 (-96.4 to 3051.9) |
|  |  |  | All ages | 0.9 (0 to 5.3) | 0.3 (0 to 2.4) | 0.6 (0 to 3.3) | 3.6 (0 to 21.7) | 1.2 (0 to 9.3) | 2.4 (0 to 12.7) | 286.5 (-83.2 to 8217.2) | 294.3 (-1567.1 to 1603313.5) | 282.5 (-82.6 to 8229.6) |
|  |  | Deaths | Age-standardized | 0 (0 to 0.1) | 0 (0 to 0.1) | 0 (0 to 0.1) | 0 (0 to 0.1) | 0 (0 to 0.1) | 0 (0 to 0.1) | -14.9 (-98.6 to 3500.5) | 1.8 (-276.6 to 170012.7) | -22.5 (-98.5 to 3702.2) |
|  |  |  | All ages | 0 (0 to 0.2) | 0 (0 to 0.1) | 0 (0 to 0.1) | 0.1 (0 to 0.8) | 0.1 (0 to 0.4) | 0.1 (0 to 0.3) | 308.4 (-92.3 to 9977.1) | 336.6 (-1069.8 to 969122.7) | 285.5 (-92.3 to 9979.3) |
|  | High alcohol use | DALYs (Disability-Adjusted Life Years) | Age-standardized | 0 (0 to 0) | 0 (0 to 0) | 0 (0 to 0) | 0.2 (0.1 to 0.3) | 0 (0 to 0.1) | 0.3 (0.2 to 0.5) | NA | NA | NA |
|  |  |  | All ages | 0 (0 to 0) | 0 (0 to 0) | 0 (0 to 0) | 1.9 (1.1 to 3.1) | 0.2 (0.1 to 0.3) | 1.8 (1 to 2.9) | NA | NA | NA |
|  |  | Deaths | Age-standardized | 0 (0 to 0) | 0 (0 to 0) | 0 (0 to 0) | 0 (0 to 0) | 0 (0 to 0) | 0 (0 to 0) | NA | NA | NA |
|  |  |  | All ages | 0 (0 to 0) | 0 (0 to 0) | 0 (0 to 0) | 0 (0 to 0.1) | 0 (0 to 0) | 0 (0 to 0.1) | NA | NA | NA |
|  | High body-mass index | DALYs (Disability-Adjusted Life Years) | Age-standardized | 3.1 (1.2 to 5.8) | 4.7 (1.8 to 9) | 1.6 (0.5 to 3.4) | 9.5 (4 to 16.7) | 13.9 (5.8 to 25.5) | 6.5 (2.7 to 11.9) | 207.5 (65.3 to 453.8) | 196.9 (39.6 to 488.7) | 299.1 (116.6 to 924.6) |
|  |  |  | All ages | 7.3 (2.8 to 12.6) | 5 (2 to 9) | 2.3 (0.8 to 4.4) | 87.6 (37.1 to 154.5) | 54.4 (23.2 to 97.8) | 33.2 (13.4 to 60.5) | 1105.9 (586.8 to 1781.4) | 986.2 (460.3 to 1740.1) | 1371.2 (754.3 to 3194.6) |
|  |  | Deaths | Age-standardized | 0.1 (0 to 0.3) | 0.2 (0.1 to 0.6) | 0 (0 to 0.1) | 0.4 (0.2 to 0.8) | 0.8 (0.3 to 1.6) | 0.2 (0.1 to 0.4) | 217.1 (34.5 to 718.5) | 248.5 (29.2 to 956.2) | 323.9 (56.9 to 1840.4) |
|  |  |  | All ages | 0.2 (0.1 to 0.5) | 0.2 (0.1 to 0.4) | 0 (0 to 0.1) | 3.4 (1.5 to 6.1) | 2.5 (1 to 4.7) | 0.9 (0.3 to 1.7) | 1404.3 (617 to 2969.8) | 1283.4 (485.2 to 3203.1) | 1858.6 (714.3 to 5958.5) |
|  | High systolic blood pressure | DALYs (Disability-Adjusted Life Years) | Age-standardized | 19.2 (6.3 to 33.4) | 24.7 (8.2 to 43.3) | 14.4 (4.4 to 26.4) | 19.3 (6.3 to 34.3) | 26.9 (9.3 to 48.2) | 14.8 (4.5 to 26.6) | 0.7 (-24.4 to 35.5) | 8.7 (-26.3 to 70.4) | 2.4 (-22.8 to 45.5) |
|  |  |  | All ages | 36.9 (12.3 to 64.5) | 21.1 (6.8 to 36.4) | 15.8 (4.7 to 28.9) | 174.5 (55.9 to 310.1) | 101.2 (34.9 to 178.6) | 73.2 (22.8 to 131.5) | 372.4 (256.4 to 518.7) | 379.6 (227.2 to 630.9) | 362.7 (248.4 to 556.1) |
|  |  | Deaths | Age-standardized | 1 (0.3 to 1.8) | 1.6 (0.5 to 3) | 0.6 (0.2 to 1.1) | 0.9 (0.3 to 1.7) | 1.7 (0.6 to 3.2) | 0.5 (0.1 to 0.9) | -10.7 (-41.8 to 34.3) | 9.2 (-36.8 to 94.4) | -18.1 (-47.7 to 40.1) |
|  |  |  | All ages | 1.5 (0.5 to 2.6) | 1 (0.3 to 1.8) | 0.5 (0.1 to 0.9) | 7.2 (2.5 to 13.3) | 5.1 (1.8 to 9.5) | 2.1 (0.6 to 3.9) | 392.2 (217.9 to 629.4) | 409.6 (206.3 to 785.9) | 354.1 (189.6 to 709.5) |
|  | Lead exposure | DALYs (Disability-Adjusted Life Years) | Age-standardized | 4.3 (-0.6 to 11) | 5 (-0.7 to 12.9) | 3.7 (-0.5 to 9.4) | 4.3 (-0.6 to 10.6) | 5.4 (-0.8 to 13.6) | 3.8 (-0.5 to 9.8) | -0.7 (-22.5 to 22.6) | 7 (-22.8 to 40.9) | 2 (-21.2 to 31.7) |
|  |  |  | All ages | 8.4 (-1.2 to 21.8) | 4.4 (-0.6 to 11.1) | 4.1 (-0.6 to 10.5) | 37.1 (-5 to 93.2) | 19 (-2.7 to 47.2) | 18.1 (-2.5 to 47.1) | 340.1 (246.5 to 436.8) | 336.2 (218 to 466.9) | 344.2 (246.6 to 465.5) |
|  |  | Deaths | Age-standardized | 0.2 (0 to 0.6) | 0.3 (-0.1 to 0.8) | 0.1 (0 to 0.4) | 0.2 (0 to 0.5) | 0.4 (-0.1 to 1) | 0.1 (0 to 0.3) | -4.2 (-31.2 to 30) | 17.7 (-21.2 to 68.8) | -9.5 (-38.5 to 50.2) |
|  |  |  | All ages | 0.3 (-0.1 to 0.8) | 0.2 (0 to 0.5) | 0.1 (0 to 0.3) | 1.6 (-0.3 to 3.9) | 1 (-0.2 to 2.7) | 0.6 (-0.1 to 1.4) | 409.7 (266.8 to 590.7) | 417.9 (243.7 to 641.8) | 395.8 (231.1 to 724.7) |
|  | Smoking | DALYs (Disability-Adjusted Life Years) | Age-standardized | 1.9 (1.1 to 2.9) | 0.7 (0.4 to 1.3) | 2.9 (1.6 to 4.7) | 1.9 (1 to 3.1) | 0.7 (0.3 to 1.3) | 3 (1.7 to 4.9) | 2.2 (-24.6 to 35.9) | -0.2 (-45.5 to 78.6) | 2.3 (-25.9 to 37.7) |
|  |  |  | All ages | 5.2 (2.9 to 8) | 0.9 (0.5 to 1.6) | 4.3 (2.3 to 6.8) | 20.8 (11.6 to 33.6) | 3.5 (1.7 to 6.2) | 17.3 (9.6 to 28.1) | 298 (204.6 to 415.2) | 282.9 (116.2 to 553.3) | 301.2 (195.4 to 440.2) |
|  |  | Deaths | Age-standardized | 0.1 (0 to 0.1) | 0 (0 to 0.1) | 0.1 (0 to 0.1) | 0 (0 to 0.1) | 0 (0 to 0.1) | 0.1 (0 to 0.1) | -10.1 (-48.4 to 54.6) | 1.6 (-61.6 to 158.9) | -16.2 (-54.2 to 55) |
|  |  |  | All ages | 0.1 (0.1 to 0.2) | 0 (0 to 0) | 0.1 (0 to 0.1) | 0.4 (0.2 to 0.7) | 0.1 (0 to 0.2) | 0.3 (0.2 to 0.5) | 294.1 (144.5 to 542.3) | 306 (88.6 to 738.8) | 290.3 (115.4 to 598.3) |
| Semnan | All risk factors | DALYs (Disability-Adjusted Life Years) | Age-standardized | 22 (10.4 to 35.1) | 23.5 (10.8 to 38.1) | 19.9 (8.8 to 32.4) | 27.1 (14.6 to 40.7) | 28.3 (14.7 to 43.3) | 25.3 (13.2 to 37.9) | 23.3 (-5.1 to 66.9) | 20.7 (-13.6 to 75) | 26.9 (-3.6 to 88.7) |
|  |  |  | All ages | 43.7 (20.7 to 69.8) | 24.3 (11.3 to 39.7) | 19.4 (9 to 31.4) | 166.6 (89.3 to 250.2) | 91.7 (47.5 to 140.2) | 74.8 (39.9 to 112.2) | 281.3 (199.8 to 406.4) | 277.9 (175.4 to 433.1) | 285.6 (201.4 to 448.5) |
|  |  | Deaths | Age-standardized | 1 (0.4 to 1.6) | 1.1 (0.5 to 1.9) | 0.7 (0.3 to 1.3) | 1.2 (0.6 to 1.8) | 1.4 (0.7 to 2.2) | 0.8 (0.4 to 1.3) | 19.1 (-20.8 to 91.8) | 23.6 (-24.1 to 108.4) | 12.9 (-26.5 to 126.9) |
|  |  |  | All ages | 1.4 (0.6 to 2.3) | 1 (0.4 to 1.7) | 0.5 (0.2 to 0.8) | 6.4 (3.4 to 9.6) | 4.3 (2.1 to 6.6) | 2.1 (1.1 to 3.2) | 340.9 (198.3 to 576.6) | 342 (175 to 636.4) | 338.6 (193.1 to 690.5) |
|  | Diet high in sodium | DALYs (Disability-Adjusted Life Years) | Age-standardized | 0.4 (0 to 2.3) | 0.3 (0 to 1.9) | 0.5 (0 to 2.7) | 0.4 (0 to 2.3) | 0.2 (0 to 1.9) | 0.5 (0 to 2.9) | -0.2 (-95.6 to 1317.4) | -5.1 (-149040.8 to 17851.1) | 0.3 (-95.3 to 1358.6) |
|  |  |  | All ages | 0.8 (0 to 5.1) | 0.3 (0 to 2) | 0.6 (0 to 3.1) | 2.4 (0 to 14.1) | 0.8 (0 to 6.2) | 1.6 (0 to 8.5) | 180 (-89 to 3481.6) | 177.4 (-1000556 to 46531.9) | 181.3 (-89 to 3511.8) |
|  |  | Deaths | Age-standardized | 0 (0 to 0.1) | 0 (0 to 0.1) | 0 (0 to 0.1) | 0 (0 to 0.1) | 0 (0 to 0.1) | 0 (0 to 0.1) | -5.2 (-98 to 1256.7) | -4.6 (-88825.3 to 18589.9) | -10.2 (-97.9 to 1282.9) |
|  |  |  | All ages | 0 (0 to 0.1) | 0 (0 to 0.1) | 0 (0 to 0.1) | 0.1 (0 to 0.5) | 0 (0 to 0.3) | 0 (0 to 0.2) | 215.6 (-93.9 to 3424.4) | 217.4 (-527606.9 to 52158.4) | 214.2 (-94.1 to 3498.3) |
|  | High alcohol use | DALYs (Disability-Adjusted Life Years) | Age-standardized | 0 (0 to 0) | 0 (0 to 0) | 0 (0 to 0) | 0.2 (0.1 to 0.3) | 0 (0 to 0.1) | 0.4 (0.2 to 0.6) | NA | NA | NA |
|  |  |  | All ages | 0 (0 to 0) | 0 (0 to 0) | 0 (0 to 0) | 1.3 (0.7 to 2) | 0.1 (0 to 0.2) | 1.2 (0.7 to 1.8) | NA | NA | NA |
|  |  | Deaths | Age-standardized | 0 (0 to 0) | 0 (0 to 0) | 0 (0 to 0) | 0 (0 to 0) | 0 (0 to 0) | 0 (0 to 0) | NA | NA | NA |
|  |  |  | All ages | 0 (0 to 0) | 0 (0 to 0) | 0 (0 to 0) | 0 (0 to 0) | 0 (0 to 0) | 0 (0 to 0) | NA | NA | NA |
|  | High body-mass index | DALYs (Disability-Adjusted Life Years) | Age-standardized | 4.3 (1.5 to 7.7) | 5.9 (2 to 10.7) | 2.3 (0.8 to 4.4) | 11.5 (4.7 to 19.9) | 13.7 (5.7 to 24.2) | 8.9 (3.4 to 15.3) | 169.2 (51.2 to 313.4) | 131.6 (19.3 to 272.1) | 294.1 (101.8 to 707.3) |
|  |  |  | All ages | 9.3 (3.4 to 15.8) | 6.6 (2.4 to 11.6) | 2.6 (0.9 to 5) | 71.1 (29.2 to 123) | 44.8 (18.7 to 79.1) | 26.3 (10.4 to 45.5) | 668.5 (352.2 to 978) | 573.5 (276.1 to 917.7) | 910.7 (430.1 to 1740.8) |
|  |  | Deaths | Age-standardized | 0.2 (0.1 to 0.4) | 0.3 (0.1 to 0.5) | 0.1 (0 to 0.2) | 0.5 (0.2 to 0.9) | 0.7 (0.3 to 1.2) | 0.3 (0.1 to 0.5) | 180.9 (32.8 to 501) | 157.4 (12.8 to 460.1) | 331.8 (76.4 to 1400.2) |
|  |  |  | All ages | 0.3 (0.1 to 0.5) | 0.2 (0.1 to 0.5) | 0.1 (0 to 0.1) | 2.7 (1.1 to 4.9) | 2 (0.8 to 3.6) | 0.7 (0.3 to 1.3) | 860.3 (386.8 to 1571.1) | 762.8 (301.2 to 1527.4) | 1287.1 (533.3 to 3315.1) |
|  | High systolic blood pressure | DALYs (Disability-Adjusted Life Years) | Age-standardized | 18.5 (6.5 to 32.4) | 20 (7.1 to 35.5) | 16.5 (5.3 to 29.3) | 19.8 (6.5 to 33.9) | 20.7 (6.8 to 36.2) | 18.5 (6 to 31.6) | 6.9 (-18.1 to 35.9) | 3.3 (-28.3 to 47.1) | 12 (-13.2 to 59.7) |
|  |  |  | All ages | 35.6 (12.3 to 61.5) | 20.3 (7.3 to 36.2) | 15.3 (4.6 to 27.8) | 120.2 (39.6 to 206) | 66.7 (22.2 to 117.4) | 53.6 (17.3 to 91.3) | 237.5 (164.5 to 335.2) | 227.8 (126.1 to 365.3) | 250.3 (173 to 377.2) |
|  |  | Deaths | Age-standardized | 0.9 (0.3 to 1.5) | 1 (0.4 to 1.8) | 0.7 (0.2 to 1.2) | 0.9 (0.3 to 1.5) | 1 (0.4 to 1.8) | 0.6 (0.2 to 1.1) | 1.8 (-33.8 to 48.5) | 5.2 (-39.3 to 65.3) | -2.7 (-37.7 to 87.6) |
|  |  |  | All ages | 1.2 (0.4 to 2.2) | 0.8 (0.3 to 1.5) | 0.4 (0.1 to 0.7) | 4.7 (1.6 to 8.2) | 3.2 (1.1 to 5.6) | 1.6 (0.5 to 2.7) | 283.9 (158.4 to 450.9) | 280.4 (120.6 to 510.4) | 291.3 (156.3 to 625.5) |
|  | Lead exposure | DALYs (Disability-Adjusted Life Years) | Age-standardized | 3.3 (-0.4 to 8.4) | 3.3 (-0.4 to 8.5) | 3.3 (-0.4 to 8.5) | 3.4 (-0.5 to 8.8) | 3.3 (-0.5 to 8.7) | 3.4 (-0.5 to 8.4) | 2.6 (-17.7 to 26.8) | 0.9 (-25.3 to 31.5) | 3.6 (-17.1 to 37.5) |
|  |  |  | All ages | 6.5 (-0.8 to 16.1) | 3.3 (-0.4 to 8.8) | 3.1 (-0.4 to 8.1) | 20.2 (-2.8 to 52.5) | 10.5 (-1.5 to 27.5) | 9.7 (-1.3 to 23.9) | 213.2 (153.8 to 287.8) | 215.5 (137 to 310) | 210.6 (147.8 to 299.2) |
|  |  | Deaths | Age-standardized | 0.2 (0 to 0.4) | 0.2 (0 to 0.4) | 0.1 (0 to 0.3) | 0.2 (0 to 0.4) | 0.2 (0 to 0.5) | 0.1 (0 to 0.3) | 6.9 (-24.1 to 48.5) | 11.5 (-28.3 to 60.5) | -1.2 (-32.5 to 73.5) |
|  |  |  | All ages | 0.2 (0 to 0.6) | 0.1 (0 to 0.4) | 0.1 (0 to 0.2) | 0.9 (-0.1 to 2.2) | 0.6 (-0.1 to 1.5) | 0.3 (-0.1 to 0.8) | 294.6 (179.9 to 449.4) | 300.1 (156.9 to 486.9) | 285 (162.8 to 568) |
|  | Smoking | DALYs (Disability-Adjusted Life Years) | Age-standardized | 1.8 (1 to 2.9) | 0.6 (0.3 to 1.1) | 3.1 (1.7 to 5) | 1.9 (1 to 2.9) | 0.6 (0.3 to 1) | 3.2 (1.7 to 5.1) | 3 (-19 to 35.4) | -3.8 (-44.7 to 66.5) | 2.2 (-24 to 35.7) |
|  |  |  | All ages | 4.6 (2.6 to 7.5) | 0.8 (0.4 to 1.4) | 3.8 (2.1 to 6.3) | 13.1 (7.1 to 20.7) | 2.2 (1 to 3.6) | 10.9 (6.1 to 17.5) | 181.2 (119.7 to 270.1) | 169.2 (59.6 to 361) | 183.7 (115.5 to 269.2) |
|  |  | Deaths | Age-standardized | 0 (0 to 0.1) | 0 (0 to 0) | 0.1 (0 to 0.1) | 0 (0 to 0.1) | 0 (0 to 0) | 0.1 (0 to 0.1) | -5.2 (-39.4 to 62) | -6.1 (-60 to 121.5) | -10.8 (-46.4 to 72.6) |
|  |  |  | All ages | 0.1 (0 to 0.1) | 0 (0 to 0) | 0.1 (0 to 0.1) | 0.3 (0.1 to 0.4) | 0.1 (0 to 0.1) | 0.2 (0.1 to 0.3) | 195.6 (89.7 to 390.1) | 195.2 (35.1 to 561.3) | 195.8 (79.8 to 408.9) |
| Sistan and Baluchistan | All risk factors | DALYs (Disability-Adjusted Life Years) | Age-standardized | 18.6 (7.7 to 31.6) | 21.8 (8.4 to 38.6) | 15.2 (6.2 to 26.3) | 21 (10.5 to 33.6) | 26.6 (12.5 to 43.5) | 15.8 (7.4 to 26.4) | 12.8 (-14.2 to 59.9) | 21.7 (-17.2 to 89) | 4.1 (-24.4 to 53.8) |
|  |  |  | All ages | 72.8 (32.5 to 119) | 37.8 (15.2 to 65.5) | 35 (15.6 to 60) | 281.6 (142.4 to 454) | 169.1 (81.1 to 274) | 112.5 (54.1 to 186) | 287.1 (200.7 to 439.1) | 347.9 (210.5 to 566.6) | 221.5 (132.9 to 356.1) |
|  |  | Deaths | Age-standardized | 0.8 (0.3 to 1.4) | 1 (0.4 to 1.9) | 0.5 (0.2 to 0.9) | 0.8 (0.4 to 1.3) | 1.2 (0.5 to 2.1) | 0.5 (0.2 to 0.8) | 5 (-30.5 to 68.2) | 19.3 (-29.6 to 103.8) | -8.4 (-44.1 to 74.2) |
|  |  |  | All ages | 2 (0.8 to 3.5) | 1.4 (0.5 to 2.5) | 0.7 (0.2 to 1.2) | 10.5 (5 to 17.2) | 7.4 (3.3 to 12.6) | 3.1 (1.3 to 5.4) | 412.9 (244.7 to 689.7) | 445.8 (227.6 to 821.7) | 348.8 (161.1 to 685.1) |
|  | Diet high in sodium | DALYs (Disability-Adjusted Life Years) | Age-standardized | 0.4 (0 to 2.3) | 0.3 (0 to 1.8) | 0.5 (0 to 2.5) | 0.4 (0 to 2.3) | 0.3 (0 to 2) | 0.5 (0 to 2.7) | 4.4 (-94.9 to 1974.1) | 5.2 (-451831.9 to 19098.4) | 5.7 (-95.2 to 2076.8) |
|  |  |  | All ages | 1.7 (0 to 9.2) | 0.5 (0 to 3.4) | 1.2 (0 to 6) | 5.1 (0 to 31.4) | 1.8 (0 to 12.5) | 3.4 (0 to 18.4) | 210.3 (-82.3 to 6036.8) | 256.7 (-26180080.6 to 37184) | 190.6 (-82.8 to 6577.1) |
|  |  | Deaths | Age-standardized | 0 (0 to 0.1) | 0 (0 to 0.1) | 0 (0 to 0.1) | 0 (0 to 0.1) | 0 (0 to 0.1) | 0 (0 to 0.1) | 3 (-97.1 to 1917.2) | 5.2 (-122601.5 to 20945.7) | -1.3 (-97.5 to 2208.7) |
|  |  |  | All ages | 0 (0 to 0.2) | 0 (0 to 0.1) | 0 (0 to 0.1) | 0.2 (0 to 1.1) | 0.1 (0 to 0.5) | 0.1 (0 to 0.5) | 310.6 (-88.1 to 6004.2) | 329.6 (-1888357.9 to 45654.6) | 297 (-88.8 to 6091.8) |
|  | High alcohol use | DALYs (Disability-Adjusted Life Years) | Age-standardized | 0 (0 to 0) | 0 (0 to 0) | 0 (0 to 0) | 0.1 (0.1 to 0.2) | 0 (0 to 0) | 0.3 (0.1 to 0.4) | NA | NA | NA |
|  |  |  | All ages | 0 (0 to 0) | 0 (0 to 0) | 0 (0 to 0) | 2.1 (1 to 3.5) | 0.2 (0 to 0.3) | 2 (1 to 3.2) | NA | NA | NA |
|  |  | Deaths | Age-standardized | 0 (0 to 0) | 0 (0 to 0) | 0 (0 to 0) | 0 (0 to 0) | 0 (0 to 0) | 0 (0 to 0) | NA | NA | NA |
|  |  |  | All ages | 0 (0 to 0) | 0 (0 to 0) | 0 (0 to 0) | 0 (0 to 0.1) | 0 (0 to 0) | 0 (0 to 0.1) | NA | NA | NA |
|  | High body-mass index | DALYs (Disability-Adjusted Life Years) | Age-standardized | 1.9 (0.7 to 3.5) | 3.2 (1.2 to 6.1) | 0.6 (0.1 to 1.4) | 6.2 (2.4 to 10.6) | 8.3 (3.2 to 14.6) | 4.2 (1.5 to 7.7) | 230.7 (91 to 577.2) | 155.5 (34.4 to 421.6) | 567.1 (208.9 to 2070.1) |
|  |  |  | All ages | 8.6 (3.2 to 15.6) | 6.6 (2.4 to 11.9) | 2 (0.5 to 4.1) | 83.8 (33 to 143.9) | 54.2 (21 to 95.3) | 29.6 (10.7 to 53.7) | 876.3 (524.3 to 1589.7) | 721.3 (364.5 to 1370.4) | 1391.5 (667.8 to 4411.1) |
|  |  | Deaths | Age-standardized | 0.1 (0 to 0.2) | 0.1 (0 to 0.3) | 0 (0 to 0) | 0.2 (0.1 to 0.4) | 0.4 (0.1 to 0.6) | 0.1 (0 to 0.2) | 230.4 (47.2 to 808.2) | 186 (20 to 689.9) | 812.3 (162.8 to 5554.7) |
|  |  |  | All ages | 0.2 (0.1 to 0.4) | 0.2 (0.1 to 0.4) | 0 (0 to 0.1) | 3 (1.1 to 5.3) | 2.2 (0.8 to 4) | 0.8 (0.3 to 1.6) | 1246.5 (589.3 to 2784) | 1029.8 (412.2 to 2488) | 2599 (949.1 to 8698.6) |
|  | High systolic blood pressure | DALYs (Disability-Adjusted Life Years) | Age-standardized | 16.3 (5.3 to 29.4) | 19.8 (6 to 36.8) | 12.8 (3.7 to 23.9) | 16.4 (5 to 28.9) | 21.9 (7.1 to 39.2) | 11.3 (3.3 to 21.8) | 0.5 (-26 to 34) | 10.9 (-26.5 to 63.5) | -11.5 (-40 to 28.5) |
|  |  |  | All ages | 61.4 (20 to 109) | 33.4 (10.6 to 61.7) | 28.1 (8.5 to 53.1) | 217.8 (66.7 to 383.3) | 138 (44 to 250.3) | 79.8 (23.7 to 153.9) | 254.4 (165.5 to 370.2) | 313.2 (175.8 to 513.1) | 184.4 (92.3 to 310.1) |
|  |  | Deaths | Age-standardized | 0.7 (0.2 to 1.3) | 0.9 (0.3 to 1.8) | 0.4 (0.1 to 0.8) | 0.7 (0.2 to 1.2) | 1 (0.3 to 1.9) | 0.3 (0.1 to 0.7) | -7.1 (-40.2 to 39.7) | 8.2 (-38.1 to 79.8) | -24 (-59.8 to 37.7) |
|  |  |  | All ages | 1.8 (0.5 to 3.3) | 1.2 (0.3 to 2.4) | 0.6 (0.2 to 1.1) | 8.4 (2.7 to 15.3) | 6.2 (1.9 to 11.3) | 2.3 (0.6 to 4.5) | 365.9 (202.7 to 600.3) | 400.7 (188.8 to 707.1) | 292.4 (111 to 591.6) |
|  | Lead exposure | DALYs (Disability-Adjusted Life Years) | Age-standardized | 4.6 (-0.6 to 11.6) | 4.9 (-0.7 to 12.4) | 4.4 (-0.6 to 10.7) | 5 (-0.7 to 12.4) | 5.3 (-0.8 to 13) | 4.8 (-0.7 to 11.8) | 8.5 (-12.9 to 35.3) | 9.1 (-22.7 to 49.1) | 9.2 (-13.2 to 42.6) |
|  |  |  | All ages | 17.2 (-2.3 to 42.9) | 8.1 (-1.2 to 21.1) | 9 (-1.2 to 22.2) | 65.5 (-9.5 to 161.1) | 33.2 (-4.8 to 81.6) | 32.3 (-4.7 to 80.3) | 281.8 (210.2 to 378.1) | 306.9 (191 to 453) | 259.1 (186.6 to 378.4) |
|  |  | Deaths | Age-standardized | 0.2 (0 to 0.5) | 0.2 (0 to 0.6) | 0.2 (0 to 0.4) | 0.2 (0 to 0.5) | 0.3 (0 to 0.6) | 0.2 (0 to 0.4) | 4.8 (-26.6 to 50.5) | 12.1 (-30.2 to 69.8) | 1.4 (-30.7 to 74.9) |
|  |  |  | All ages | 0.5 (-0.1 to 1.3) | 0.3 (0 to 0.8) | 0.2 (0 to 0.5) | 2.7 (-0.5 to 6.4) | 1.6 (-0.3 to 3.9) | 1.1 (-0.2 to 2.8) | 433.5 (277.3 to 673.9) | 428.1 (232.2 to 709.1) | 441.6 (257.4 to 836.9) |
|  | Smoking | DALYs (Disability-Adjusted Life Years) | Age-standardized | 1.7 (1 to 2.8) | 0.6 (0.3 to 1) | 2.7 (1.4 to 4.4) | 1.6 (0.9 to 2.5) | 0.6 (0.3 to 1) | 2.6 (1.4 to 4.1) | -8.1 (-29.8 to 24.6) | -2.8 (-45.8 to 73.2) | -5.4 (-28.3 to 33.1) |
|  |  |  | All ages | 9.3 (5.1 to 15.2) | 1.4 (0.7 to 2.4) | 7.9 (4.1 to 13) | 24 (13 to 37.5) | 4.4 (2.1 to 7.5) | 19.7 (10.8 to 31.4) | 158.1 (97.7 to 247) | 208.1 (70.7 to 420.9) | 149.1 (83.7 to 250.4) |
|  |  | Deaths | Age-standardized | 0 (0 to 0.1) | 0 (0 to 0) | 0.1 (0 to 0.1) | 0 (0 to 0.1) | 0 (0 to 0) | 0.1 (0 to 0.1) | -6 (-40.7 to 60.5) | -3.6 (-55.4 to 109.2) | -10.7 (-44.7 to 71.3) |
|  |  |  | All ages | 0.2 (0.1 to 0.3) | 0 (0 to 0.1) | 0.1 (0.1 to 0.2) | 0.5 (0.3 to 0.9) | 0.1 (0.1 to 0.2) | 0.4 (0.2 to 0.7) | 229.4 (108.2 to 454.1) | 261.7 (77.7 to 627.6) | 220.8 (90.9 to 507.6) |
| South Khorasan | All risk factors | DALYs (Disability-Adjusted Life Years) | Age-standardized | 17.1 (7.1 to 28.3) | 19.2 (7.8 to 32) | 15 (6.2 to 25.8) | 22.2 (11.4 to 35.3) | 24.4 (12.2 to 38.5) | 19.7 (10 to 31.7) | 30 (-2.3 to 89.8) | 26.9 (-13.1 to 97.4) | 31.9 (-2.6 to 101.4) |
|  |  |  | All ages | 43.3 (18.9 to 72.2) | 22.5 (9.5 to 37.2) | 20.8 (8.6 to 34.7) | 164.3 (84.1 to 261.6) | 94.3 (46.7 to 148.2) | 70 (35.8 to 111.4) | 279.2 (196.5 to 451.1) | 318.3 (191.3 to 529) | 236.8 (157 to 396.9) |
|  |  | Deaths | Age-standardized | 0.7 (0.3 to 1.3) | 0.9 (0.3 to 1.7) | 0.5 (0.2 to 1) | 0.9 (0.4 to 1.5) | 1.2 (0.5 to 1.9) | 0.6 (0.3 to 1) | 28.5 (-15.5 to 122.4) | 27.5 (-23.8 to 131.1) | 23.9 (-24 to 168.1) |
|  |  |  | All ages | 1.3 (0.5 to 2.3) | 0.8 (0.3 to 1.4) | 0.5 (0.2 to 0.9) | 7 (3.3 to 11.6) | 4.8 (2.2 to 8) | 2.2 (1 to 3.6) | 436.1 (252 to 806.4) | 483.4 (247.4 to 940.4) | 355.5 (186.5 to 851.3) |
|  | Diet high in sodium | DALYs (Disability-Adjusted Life Years) | Age-standardized | 0.3 (0 to 2) | 0.2 (0 to 1.7) | 0.4 (0 to 2.4) | 0.3 (0 to 2.1) | 0.2 (0 to 1.8) | 0.5 (0 to 2.6) | 2.7 (-94.5 to 2157.8) | 4.3 (-120706.3 to 26520.9) | 5.6 (-93.9 to 2352.4) |
|  |  |  | All ages | 1 (0 to 5.4) | 0.3 (0 to 2.2) | 0.7 (0 to 3.4) | 2.5 (0 to 15.3) | 0.9 (0 to 6.7) | 1.6 (0 to 9.3) | 162.9 (-89.9 to 3703.2) | 202 (-264943.1 to 70296.1) | 145 (-89.9 to 4375.5) |
|  |  | Deaths | Age-standardized | 0 (0 to 0.1) | 0 (0 to 0.1) | 0 (0 to 0.1) | 0 (0 to 0.1) | 0 (0 to 0.1) | 0 (0 to 0.1) | 0.4 (-97 to 2265.1) | 4.5 (-17270.3 to 41363.7) | 0.6 (-96.7 to 2653.3) |
|  |  |  | All ages | 0 (0 to 0.1) | 0 (0 to 0.1) | 0 (0 to 0.1) | 0.1 (0 to 0.6) | 0 (0 to 0.3) | 0 (0 to 0.3) | 264.6 (-93.8 to 5716.1) | 309.8 (-205854.5 to 57376) | 232.2 (-93.8 to 5719.6) |
|  | High alcohol use | DALYs (Disability-Adjusted Life Years) | Age-standardized | 0 (0 to 0) | 0 (0 to 0) | 0 (0 to 0) | 0.2 (0.1 to 0.3) | 0 (0 to 0.1) | 0.3 (0.2 to 0.5) | NA | NA | NA |
|  |  |  | All ages | 0 (0 to 0) | 0 (0 to 0) | 0 (0 to 0) | 1.2 (0.7 to 1.9) | 0.1 (0 to 0.2) | 1.1 (0.6 to 1.7) | NA | NA | NA |
|  |  | Deaths | Age-standardized | 0 (0 to 0) | 0 (0 to 0) | 0 (0 to 0) | 0 (0 to 0) | 0 (0 to 0) | 0 (0 to 0) | NA | NA | NA |
|  |  |  | All ages | 0 (0 to 0) | 0 (0 to 0) | 0 (0 to 0) | 0 (0 to 0.1) | 0 (0 to 0) | 0 (0 to 0) | NA | NA | NA |
|  | High body-mass index | DALYs (Disability-Adjusted Life Years) | Age-standardized | 2.4 (0.9 to 4.5) | 4.1 (1.6 to 7.7) | 0.8 (0.2 to 1.8) | 9.2 (3.7 to 16) | 11.4 (4.7 to 20.3) | 6.9 (2.6 to 12.1) | 286 (114.1 to 574.7) | 175.2 (34.5 to 400.3) | 807.1 (338.2 to 3099.7) |
|  |  |  | All ages | 7.1 (2.6 to 12.6) | 5.7 (2.2 to 10.2) | 1.4 (0.4 to 2.8) | 68.5 (27.3 to 119.2) | 44 (18 to 78.1) | 24.5 (9.1 to 43) | 867.5 (486.6 to 1448.5) | 675.4 (327.7 to 1166.8) | 1642.3 (833.5 to 5145.3) |
|  |  | Deaths | Age-standardized | 0.1 (0 to 0.2) | 0.2 (0.1 to 0.4) | 0 (0 to 0.1) | 0.4 (0.2 to 0.7) | 0.5 (0.2 to 1) | 0.2 (0.1 to 0.4) | 331.3 (88.3 to 1016.1) | 219.5 (24.8 to 762.1) | 1265.3 (287.2 to 12533.6) |
|  |  |  | All ages | 0.2 (0.1 to 0.4) | 0.2 (0.1 to 0.3) | 0 (0 to 0.1) | 2.9 (1.1 to 5.2) | 2.1 (0.8 to 4) | 0.8 (0.3 to 1.5) | 1438.4 (664.1 to 2967.6) | 1164 (447.2 to 2600.1) | 3524.2 (1300.4 to 17421.8) |
|  | High systolic blood pressure | DALYs (Disability-Adjusted Life Years) | Age-standardized | 14.6 (4.6 to 26.1) | 16.6 (5 to 30.1) | 12.6 (3.9 to 23.5) | 16 (5 to 28.6) | 17.8 (5.2 to 31.8) | 13.9 (4.5 to 25.9) | 9.5 (-19.6 to 47.5) | 7 (-31.6 to 66) | 10.8 (-19.3 to 56.9) |
|  |  |  | All ages | 35.5 (11.1 to 63.8) | 18.9 (5.9 to 34.3) | 16.6 (4.8 to 30.5) | 117.9 (37 to 210) | 68.7 (20.3 to 123.3) | 49.2 (15.9 to 91) | 232.3 (142.4 to 347.4) | 264.2 (132.2 to 443.9) | 196.1 (116.9 to 321.8) |
|  |  | Deaths | Age-standardized | 0.6 (0.2 to 1.2) | 0.8 (0.3 to 1.6) | 0.5 (0.1 to 0.9) | 0.7 (0.2 to 1.3) | 0.9 (0.3 to 1.7) | 0.5 (0.2 to 0.9) | 6.6 (-32.2 to 78.4) | 6.8 (-39.5 to 95.7) | 0.6 (-39.3 to 101.7) |
|  |  |  | All ages | 1.1 (0.4 to 2.1) | 0.7 (0.2 to 1.3) | 0.4 (0.1 to 0.8) | 5.2 (1.6 to 9.6) | 3.6 (1 to 6.8) | 1.6 (0.5 to 3) | 356.7 (189 to 651.2) | 399.7 (163.7 to 789.2) | 282.4 (130.2 to 647.2) |
|  | Lead exposure | DALYs (Disability-Adjusted Life Years) | Age-standardized | 4.3 (-0.6 to 11.1) | 4.5 (-0.7 to 11.2) | 4.1 (-0.6 to 10.3) | 4.6 (-0.6 to 11.5) | 4.7 (-0.6 to 12.2) | 4.5 (-0.6 to 10.7) | 7.7 (-15 to 35.5) | 5.6 (-24.1 to 47.9) | 9.7 (-14 to 48.3) |
|  |  |  | All ages | 10.4 (-1.4 to 26.7) | 5.1 (-0.7 to 13.1) | 5.3 (-0.7 to 13.4) | 34.1 (-4.7 to 85.2) | 18.4 (-2.5 to 47.2) | 15.7 (-2.2 to 37.7) | 226.9 (162.8 to 312.7) | 259.8 (160.6 to 395.7) | 195.3 (135.1 to 292.8) |
|  |  | Deaths | Age-standardized | 0.2 (0 to 0.5) | 0.2 (0 to 0.6) | 0.2 (0 to 0.4) | 0.2 (0 to 0.5) | 0.2 (0 to 0.6) | 0.2 (0 to 0.4) | 11.5 (-22.5 to 69.8) | 12.1 (-29.3 to 81.1) | 7.5 (-26 to 115.3) |
|  |  |  | All ages | 0.3 (-0.1 to 0.8) | 0.2 (0 to 0.5) | 0.1 (0 to 0.3) | 1.6 (-0.2 to 4) | 1 (-0.2 to 2.7) | 0.6 (-0.1 to 1.4) | 379.7 (231.9 to 634.7) | 427.3 (232.9 to 771) | 312 (179.8 to 701.6) |
|  | Smoking | DALYs (Disability-Adjusted Life Years) | Age-standardized | 1.6 (0.9 to 2.6) | 0.6 (0.3 to 1) | 2.6 (1.3 to 4.2) | 1.6 (0.8 to 2.4) | 0.5 (0.3 to 0.9) | 2.7 (1.4 to 4.2) | -3 (-25.4 to 31.2) | -2.4 (-44.4 to 67.3) | 2.9 (-22.3 to 43) |
|  |  |  | All ages | 5.4 (2.9 to 8.6) | 0.9 (0.5 to 1.5) | 4.5 (2.4 to 7.4) | 11.9 (6.4 to 18.6) | 2.1 (1.1 to 3.6) | 9.8 (5.1 to 15.3) | 120.7 (67.6 to 197) | 140.1 (37.3 to 320.7) | 116.8 (58.3 to 198.7) |
|  |  | Deaths | Age-standardized | 0 (0 to 0.1) | 0 (0 to 0) | 0.1 (0 to 0.1) | 0 (0 to 0.1) | 0 (0 to 0) | 0.1 (0 to 0.1) | -8 (-44.8 to 71.7) | -3.4 (-61.2 to 117) | -3.1 (-43.5 to 104.2) |
|  |  |  | All ages | 0.1 (0 to 0.1) | 0 (0 to 0) | 0.1 (0 to 0.1) | 0.3 (0.1 to 0.5) | 0.1 (0 to 0.1) | 0.2 (0.1 to 0.3) | 197.9 (75.5 to 451.3) | 231.5 (38.5 to 603.3) | 188.4 (65.2 to 497.1) |
| Tehran | All risk factors | DALYs (Disability-Adjusted Life Years) | Age-standardized | 22.2 (9.8 to 35.1) | 25.1 (11.1 to 41.8) | 19.1 (8.4 to 31.1) | 22.9 (11.8 to 35.3) | 26 (13.2 to 41.2) | 20.6 (10.6 to 32.5) | 3 (-21.7 to 44.1) | 3.7 (-30.9 to 58.9) | 8.3 (-21.1 to 59.8) |
|  |  |  | All ages | 638.3 (299.3 to 1027) | 349.9 (165.6 to 576.2) | 288.4 (131.1 to 468.5) | 3165.7 (1646.7 to 4860) | 1611.8 (829.1 to 2506.9) | 1553.9 (807 to 2443.3) | 395.9 (282.7 to 562.5) | 360.6 (217.3 to 567.8) | 438.8 (303.4 to 666.2) |
|  |  | Deaths | Age-standardized | 1 (0.4 to 1.6) | 1.2 (0.5 to 2.1) | 0.7 (0.2 to 1.2) | 0.9 (0.5 to 1.5) | 1.3 (0.6 to 2.2) | 0.7 (0.3 to 1.1) | -2 (-33.9 to 58.1) | 5.6 (-37.5 to 84.8) | 3.4 (-36.5 to 95.5) |
|  |  |  | All ages | 20.9 (8.9 to 34.2) | 13.7 (5.9 to 23.6) | 7.2 (2.5 to 13.3) | 123.8 (62 to 195.5) | 71.8 (34.6 to 119.5) | 52 (26 to 83.6) | 493.2 (297.8 to 842) | 423.2 (201.6 to 798.6) | 627.3 (350.4 to 1239.8) |
|  | Diet high in sodium | DALYs (Disability-Adjusted Life Years) | Age-standardized | 0.4 (0 to 2.1) | 0.2 (0 to 1.9) | 0.5 (0 to 2.5) | 0.3 (0 to 2.2) | 0.2 (0 to 1.8) | 0.5 (0 to 2.6) | -2.8 (-97.1 to 813.6) | -7.4 (-26381.9 to 6368.2) | -3.1 (-97 to 1000.3) |
|  |  |  | All ages | 11.6 (0 to 66.3) | 3.9 (0 to 27.9) | 7.7 (0 to 40.6) | 48.4 (0 to 304.9) | 14.8 (0 to 114.3) | 33.6 (0 to 192.4) | 318.7 (-89.9 to 2946) | 283.1 (-14578.6 to 35998.1) | 336.5 (-89.9 to 3641.7) |
|  |  | Deaths | Age-standardized | 0 (0 to 0.1) | 0 (0 to 0.1) | 0 (0 to 0.1) | 0 (0 to 0.1) | 0 (0 to 0.1) | 0 (0 to 0.1) | -5.8 (-98.4 to 807.7) | -5 (-615.5 to 12175) | -9.2 (-98.3 to 841.5) |
|  |  |  | All ages | 0.3 (0 to 1.9) | 0.1 (0 to 1) | 0.2 (0 to 1) | 1.5 (0 to 10.7) | 0.6 (0 to 4.5) | 1 (0 to 6.3) | 411.1 (-94 to 2776.2) | 336.6 (-32764.4 to 25507.5) | 468.5 (-94 to 3428.5) |
|  | High alcohol use | DALYs (Disability-Adjusted Life Years) | Age-standardized | 0 (0 to 0) | 0 (0 to 0) | 0 (0 to 0) | 0.2 (0.1 to 0.3) | 0 (0 to 0.1) | 0.4 (0.2 to 0.5) | NA | NA | NA |
|  |  |  | All ages | 0 (0 to 0) | 0 (0 to 0) | 0 (0 to 0) | 30.6 (17.5 to 46.1) | 2.3 (0.9 to 4) | 28.4 (16.2 to 42.5) | NA | NA | NA |
|  |  | Deaths | Age-standardized | 0 (0 to 0) | 0 (0 to 0) | 0 (0 to 0) | 0 (0 to 0) | 0 (0 to 0) | 0 (0 to 0) | NA | NA | NA |
|  |  |  | All ages | 0 (0 to 0) | 0 (0 to 0) | 0 (0 to 0) | 0.8 (0.4 to 1.3) | 0.1 (0 to 0.2) | 0.7 (0.4 to 1.2) | NA | NA | NA |
|  | High body-mass index | DALYs (Disability-Adjusted Life Years) | Age-standardized | 4.3 (1.8 to 7.5) | 6.1 (2.5 to 11) | 2.4 (0.9 to 4.9) | 10.4 (4.3 to 18.4) | 13.7 (5.6 to 24.1) | 7.7 (3 to 14.3) | 139.9 (43.1 to 247) | 123.3 (22.4 to 249.7) | 215.3 (64.3 to 511.5) |
|  |  |  | All ages | 137 (55.7 to 236) | 95.1 (37.6 to 168.1) | 42 (15.9 to 78.2) | 1442.8 (593.1 to 2562.4) | 860.8 (350.2 to 1509.7) | 582 (228.2 to 1084.9) | 952.9 (563.9 to 1336.7) | 805.6 (425.7 to 1224.6) | 1286.5 (674.1 to 2174.1) |
|  |  | Deaths | Age-standardized | 0.2 (0.1 to 0.3) | 0.3 (0.1 to 0.5) | 0.1 (0 to 0.2) | 0.4 (0.2 to 0.8) | 0.7 (0.3 to 1.2) | 0.3 (0.1 to 0.5) | 151.2 (32.7 to 345.5) | 151.2 (15.4 to 391.8) | 265.7 (54.7 to 922.4) |
|  |  |  | All ages | 4 (1.7 to 7.4) | 3.2 (1.3 to 6.2) | 0.9 (0.3 to 1.8) | 57.1 (23.2 to 103.2) | 36.8 (14.5 to 67.3) | 20.3 (7.8 to 39.1) | 1311.3 (671.7 to 2238.4) | 1058.5 (459.2 to 2011.1) | 2238.9 (997.7 to 5285.3) |
|  | High systolic blood pressure | DALYs (Disability-Adjusted Life Years) | Age-standardized | 18.6 (6.2 to 31.8) | 21.6 (7.1 to 37.9) | 15.5 (4.7 to 27.6) | 15.8 (5.2 to 28.6) | 18.1 (5.6 to 32.9) | 14.1 (4.5 to 25.5) | -15.5 (-37.3 to 10.1) | -16.3 (-46.5 to 21.8) | -9 (-35.9 to 25.1) |
|  |  |  | All ages | 513.7 (164.1 to 888.4) | 293.3 (98.3 to 511.8) | 220.4 (63.3 to 398.9) | 2158.4 (713.3 to 3908.4) | 1109.3 (343.2 to 2021.6) | 1049.1 (333.5 to 1913.5) | 320.2 (219.1 to 435.7) | 278.2 (145.8 to 447.2) | 376 (241 to 582.2) |
|  |  | Deaths | Age-standardized | 0.8 (0.3 to 1.5) | 1.1 (0.4 to 2) | 0.6 (0.1 to 1.1) | 0.7 (0.2 to 1.2) | 0.9 (0.3 to 1.8) | 0.5 (0.1 to 0.9) | -20.9 (-47.1 to 21.6) | -15.4 (-50.4 to 43.1) | -15.2 (-50.1 to 47.7) |
|  |  |  | All ages | 17.9 (5.7 to 31) | 12 (3.9 to 21.8) | 6 (1.6 to 12) | 87.3 (28.2 to 161.9) | 50.7 (15.2 to 98.1) | 36.6 (10.3 to 68.9) | 386.8 (225 to 642.8) | 324.2 (142.1 to 611.1) | 511.8 (261.9 to 971.8) |
|  | Lead exposure | DALYs (Disability-Adjusted Life Years) | Age-standardized | 2.5 (-0.3 to 6.5) | 2.5 (-0.4 to 6.8) | 2.5 (-0.3 to 6.4) | 2.8 (-0.4 to 7.4) | 2.8 (-0.4 to 7.4) | 2.9 (-0.4 to 7.4) | 13 (-8.5 to 38.3) | 12.5 (-21 to 52.5) | 16.2 (-11.6 to 51.6) |
|  |  |  | All ages | 72.1 (-9.4 to 187.5) | 35.2 (-4.7 to 95.6) | 36.9 (-4.8 to 96.7) | 380.7 (-49 to 1006.6) | 167.8 (-21.3 to 445.9) | 212.9 (-28 to 559.8) | 428.1 (336.7 to 546) | 377.4 (241 to 542.4) | 476.5 (352.6 to 651.2) |
|  |  | Deaths | Age-standardized | 0.1 (0 to 0.3) | 0.1 (0 to 0.3) | 0.1 (0 to 0.2) | 0.1 (0 to 0.3) | 0.2 (0 to 0.4) | 0.1 (0 to 0.3) | 18.8 (-15.4 to 65.9) | 27 (-23.5 to 92.6) | 21 (-20.8 to 99.8) |
|  |  |  | All ages | 2.3 (-0.3 to 6) | 1.4 (-0.2 to 3.7) | 0.9 (-0.1 to 2.6) | 16.6 (-2.3 to 43.1) | 8.4 (-1.2 to 21.8) | 8.1 (-1.2 to 21.1) | 616.6 (410.1 to 892.2) | 517.2 (272.4 to 832.2) | 760 (458.4 to 1337) |
|  | Smoking | DALYs (Disability-Adjusted Life Years) | Age-standardized | 1.9 (1 to 3.1) | 0.7 (0.3 to 1.1) | 3.2 (1.7 to 5) | 1.9 (1 to 3) | 0.6 (0.3 to 1) | 3.1 (1.7 to 4.9) | -3.6 (-26.6 to 21.3) | -13.1 (-50.4 to 43.6) | -5 (-28.3 to 22) |
|  |  |  | All ages | 75.3 (39.8 to 115.2) | 12.4 (6.3 to 21.1) | 63 (33.1 to 97.3) | 280.8 (151.3 to 442.9) | 41.3 (19.7 to 70) | 239.4 (132.2 to 384.5) | 272.7 (186.1 to 369.8) | 234.5 (102.3 to 436) | 280.1 (186.7 to 391.3) |
|  |  | Deaths | Age-standardized | 0 (0 to 0.1) | 0 (0 to 0) | 0.1 (0 to 0.1) | 0 (0 to 0.1) | 0 (0 to 0) | 0.1 (0 to 0.1) | -2.4 (-42.2 to 61.1) | -11.4 (-61.5 to 94.7) | -10.1 (-49.1 to 56.9) |
|  |  |  | All ages | 1.3 (0.7 to 2.2) | 0.3 (0.1 to 0.5) | 1 (0.5 to 1.8) | 6.3 (3.1 to 10.5) | 1.1 (0.5 to 2.1) | 5.2 (2.5 to 9.1) | 386 (190.1 to 690.8) | 279.1 (76.8 to 683.5) | 416.5 (192.4 to 797.2) |
| West Azarbayejan | All risk factors | DALYs (Disability-Adjusted Life Years) | Age-standardized | 23.9 (9.7 to 38.9) | 28.2 (11.6 to 47.3) | 19.4 (7.8 to 31.3) | 29.8 (14.5 to 46.7) | 35.7 (16.9 to 57.9) | 23.9 (11.6 to 37.1) | 24.6 (-4.7 to 77.2) | 26.7 (-15.7 to 91.5) | 22.8 (-8.2 to 89.7) |
|  |  |  | All ages | 142 (63.4 to 223) | 76.1 (32.6 to 124.3) | 65.9 (27.9 to 104) | 620.3 (318.4 to 946.2) | 364.4 (171.4 to 580.1) | 255.9 (128.9 to 396.3) | 337 (247.7 to 500.9) | 378.8 (232.8 to 604.6) | 288.6 (200.8 to 460) |
|  |  | Deaths | Age-standardized | 1.3 (0.5 to 2.2) | 1.7 (0.6 to 2.9) | 0.8 (0.3 to 1.5) | 1.6 (0.7 to 2.6) | 2.2 (1 to 3.7) | 1 (0.4 to 1.6) | 25.5 (-19.7 to 108.7) | 34 (-24.4 to 135.3) | 14.9 (-25.8 to 125.9) |
|  |  |  | All ages | 4.9 (1.9 to 8.1) | 3.2 (1.2 to 5.6) | 1.7 (0.6 to 2.9) | 25.1 (11.1 to 40.1) | 17.7 (7.5 to 29.4) | 7.4 (3.5 to 11.9) | 417.7 (248.8 to 721.7) | 454 (230.5 to 844.4) | 347.6 (196.5 to 732.9) |
|  | Diet high in sodium | DALYs (Disability-Adjusted Life Years) | Age-standardized | 0.4 (0 to 2.5) | 0.3 (0 to 2.3) | 0.5 (0 to 2.8) | 0.4 (0 to 2.6) | 0.3 (0 to 2.5) | 0.5 (0 to 3) | 1.2 (-95.9 to 2398.3) | 4.6 (-372.9 to 22500.1) | 1.9 (-96.4 to 2702.1) |
|  |  |  | All ages | 2.9 (0 to 16) | 0.9 (0 to 6.3) | 2 (0 to 10.2) | 9.5 (0 to 55) | 3.5 (0 to 25.7) | 6 (0 to 32.7) | 231.1 (-84.1 to 6779.5) | 284.4 (-1934.2 to 37345.4) | 206.7 (-87.5 to 7248.3) |
|  |  | Deaths | Age-standardized | 0 (0 to 0.1) | 0 (0 to 0.1) | 0 (0 to 0.1) | 0 (0 to 0.1) | 0 (0 to 0.2) | 0 (0 to 0.1) | 2.4 (-97 to 2691.6) | 10.1 (-5187.9 to 24193.6) | -4.5 (-97.6 to 2770.5) |
|  |  |  | All ages | 0.1 (0 to 0.5) | 0 (0 to 0.3) | 0 (0 to 0.2) | 0.3 (0 to 2) | 0.1 (0 to 1.2) | 0.2 (0 to 1) | 288.1 (-87.7 to 6361.9) | 341.5 (-654.6 to 61803) | 248.9 (-90.5 to 6515.7) |
|  | High alcohol use | DALYs (Disability-Adjusted Life Years) | Age-standardized | 0 (0 to 0) | 0 (0 to 0) | 0 (0 to 0) | 0.2 (0.1 to 0.3) | 0 (0 to 0.1) | 0.3 (0.2 to 0.5) | NA | NA | NA |
|  |  |  | All ages | 0 (0 to 0) | 0 (0 to 0) | 0 (0 to 0) | 4.5 (2.5 to 6.8) | 0.4 (0.1 to 0.8) | 4.1 (2.3 to 6.3) | NA | NA | NA |
|  |  | Deaths | Age-standardized | 0 (0 to 0) | 0 (0 to 0) | 0 (0 to 0) | 0 (0 to 0) | 0 (0 to 0) | 0 (0 to 0) | NA | NA | NA |
|  |  |  | All ages | 0 (0 to 0) | 0 (0 to 0) | 0 (0 to 0) | 0.1 (0 to 0.2) | 0 (0 to 0) | 0.1 (0 to 0.1) | NA | NA | NA |
|  | High body-mass index | DALYs (Disability-Adjusted Life Years) | Age-standardized | 2.9 (1 to 5.7) | 4.9 (1.8 to 10.2) | 0.9 (0.2 to 2) | 9.7 (4 to 16.3) | 12.9 (5.1 to 22.4) | 6.5 (2.3 to 12.1) | 235.3 (75.4 to 556.4) | 161 (27.9 to 419.5) | 621.7 (264.1 to 2515.5) |
|  |  |  | All ages | 20.6 (8.3 to 35.7) | 16.3 (6.6 to 29.4) | 4.3 (1.3 to 8.2) | 204.1 (84.3 to 351.5) | 135.3 (53.4 to 232.9) | 68.8 (26.1 to 123.5) | 892 (544.8 to 1485.7) | 731.6 (374.8 to 1218.5) | 1498.4 (830.1 to 4554.5) |
|  |  | Deaths | Age-standardized | 0.1 (0 to 0.3) | 0.2 (0.1 to 0.6) | 0 (0 to 0.1) | 0.5 (0.2 to 0.9) | 0.8 (0.3 to 1.4) | 0.3 (0.1 to 0.5) | 272.4 (44.4 to 1112.1) | 216.2 (15.1 to 1001.4) | 938.6 (240.2 to 8217) |
|  |  |  | All ages | 0.6 (0.2 to 1.3) | 0.5 (0.2 to 1.2) | 0.1 (0 to 0.2) | 8.1 (3.2 to 14.1) | 6.1 (2.4 to 10.9) | 2 (0.6 to 3.9) | 1221.1 (530.7 to 2711.2) | 1033.3 (387.3 to 2495.4) | 2575.9 (1126.9 to 9758.8) |
|  | High systolic blood pressure | DALYs (Disability-Adjusted Life Years) | Age-standardized | 21 (6.8 to 36) | 25.2 (8.4 to 44.5) | 16.7 (4.9 to 28.8) | 23.2 (7.6 to 40.5) | 28.5 (9.3 to 51.5) | 17.9 (5.9 to 31) | 10.5 (-20.2 to 48.4) | 13 (-28.9 to 70) | 7.6 (-22.2 to 53.7) |
|  |  |  | All ages | 118.8 (39.3 to 200) | 65.7 (22.1 to 113.8) | 53 (15.8 to 91.2) | 474.3 (155.5 to 806.6) | 287.7 (92.7 to 509.8) | 186.6 (59.4 to 322.5) | 299.4 (205.3 to 415.9) | 337.7 (187.6 to 545.5) | 251.9 (159.2 to 392.3) |
|  |  | Deaths | Age-standardized | 1.2 (0.3 to 2.1) | 1.5 (0.5 to 2.8) | 0.7 (0.2 to 1.4) | 1.3 (0.4 to 2.3) | 1.8 (0.5 to 3.4) | 0.7 (0.2 to 1.4) | 10.1 (-31.8 to 71.7) | 18.5 (-36.1 to 106.1) | -1.3 (-39 to 91.4) |
|  |  |  | All ages | 4.3 (1.3 to 7.5) | 2.9 (0.9 to 5.2) | 1.4 (0.4 to 2.6) | 20 (6.7 to 35.7) | 14.3 (4.4 to 25.8) | 5.7 (1.9 to 10.3) | 365.9 (197.4 to 609.7) | 399.6 (175.4 to 754.2) | 298.1 (156.9 to 615.7) |
|  | Lead exposure | DALYs (Disability-Adjusted Life Years) | Age-standardized | 5.1 (-0.7 to 12.8) | 5.5 (-0.8 to 14.4) | 4.6 (-0.7 to 11.4) | 5.7 (-0.8 to 13.9) | 6.3 (-0.8 to 15.7) | 5 (-0.7 to 12.3) | 11.8 (-11.8 to 43.4) | 15.5 (-19.4 to 56.5) | 9.2 (-11.5 to 51.8) |
|  |  |  | All ages | 28.5 (-3.9 to 72.4) | 14.2 (-2 to 37.6) | 14.3 (-2 to 36) | 109.2 (-14.1 to 266.7) | 59.8 (-7.7 to 145.8) | 49.4 (-6.5 to 121.6) | 283 (207 to 380.8) | 322.2 (199.6 to 459.8) | 244.2 (180.3 to 348.6) |
|  |  | Deaths | Age-standardized | 0.3 (0 to 0.7) | 0.3 (-0.1 to 0.9) | 0.2 (0 to 0.6) | 0.3 (0 to 0.8) | 0.4 (-0.1 to 1.1) | 0.2 (0 to 0.6) | 18.6 (-18.3 to 70.3) | 28.6 (-18.3 to 91.4) | 7.7 (-27 to 102.2) |
|  |  |  | All ages | 1 (-0.2 to 2.7) | 0.6 (-0.1 to 1.6) | 0.4 (-0.1 to 1.1) | 4.9 (-0.7 to 12) | 3.2 (-0.4 to 8.2) | 1.7 (-0.2 to 4.2) | 378.4 (237.1 to 590) | 415.1 (217.1 to 671.6) | 320.6 (194.7 to 660.6) |
|  | Smoking | DALYs (Disability-Adjusted Life Years) | Age-standardized | 1.9 (1 to 3) | 0.7 (0.3 to 1.2) | 3.1 (1.6 to 4.7) | 2 (1.1 to 3.2) | 0.7 (0.3 to 1.3) | 3.3 (1.8 to 5.5) | 4.6 (-20.4 to 36.9) | 7.3 (-41.5 to 94.5) | 8.4 (-18.8 to 49) |
|  |  |  | All ages | 17.2 (9.4 to 26.6) | 2.9 (1.4 to 5) | 14.3 (7.6 to 22.2) | 53.8 (29.7 to 86.2) | 9.8 (4.8 to 16.9) | 44 (24.1 to 69.6) | 212.3 (141.1 to 309) | 233 (93.9 to 463.8) | 208 (133.7 to 315.3) |
|  |  | Deaths | Age-standardized | 0.1 (0 to 0.1) | 0 (0 to 0.1) | 0.1 (0 to 0.2) | 0.1 (0 to 0.1) | 0 (0 to 0.1) | 0.1 (0 to 0.2) | 4.7 (-35.5 to 86.5) | 13.7 (-57.8 to 200.6) | 1.5 (-37.1 to 102.2) |
|  |  |  | All ages | 0.3 (0.2 to 0.5) | 0.1 (0 to 0.1) | 0.2 (0.1 to 0.4) | 1.1 (0.6 to 1.9) | 0.3 (0.1 to 0.6) | 0.8 (0.4 to 1.4) | 252.2 (138.9 to 477.2) | 292.7 (81.4 to 772.3) | 240.2 (113.9 to 528.7) |
| Yazd | All risk factors | DALYs (Disability-Adjusted Life Years) | Age-standardized | 23.1 (10.1 to 37.3) | 25.6 (10.7 to 42.8) | 19.1 (8.2 to 31.6) | 28.3 (14.2 to 43.5) | 33.2 (15.9 to 51.2) | 22.7 (11.6 to 35.1) | 22.8 (-5.3 to 74.6) | 29.9 (-7.9 to 98.6) | 19 (-7.5 to 76.1) |
|  |  |  | All ages | 56.3 (25.9 to 91.9) | 33.6 (14.8 to 54.8) | 22.7 (10.1 to 37.5) | 236.1 (122.7 to 363.2) | 139.7 (68.1 to 214.4) | 96.4 (49.5 to 149.1) | 319.5 (230 to 469.7) | 316.2 (199.4 to 531.9) | 324.4 (236.5 to 495.4) |
|  |  | Deaths | Age-standardized | 1.1 (0.4 to 1.9) | 1.3 (0.5 to 2.4) | 0.7 (0.2 to 1.3) | 1.3 (0.7 to 2) | 1.8 (0.8 to 2.8) | 0.8 (0.4 to 1.2) | 19.4 (-19.2 to 93.9) | 33 (-14.7 to 129.7) | 6.8 (-32.9 to 115.9) |
|  |  |  | All ages | 2 (0.8 to 3.3) | 1.4 (0.6 to 2.5) | 0.5 (0.2 to 0.9) | 10 (5.1 to 15.4) | 7.2 (3.4 to 11.5) | 2.7 (1.3 to 4.4) | 401 (242.5 to 692.6) | 400.1 (215.1 to 750.1) | 403.3 (231.2 to 864.6) |
|  | Diet high in sodium | DALYs (Disability-Adjusted Life Years) | Age-standardized | 0.4 (0 to 2.4) | 0.3 (0 to 2.1) | 0.5 (0 to 2.8) | 0.4 (0 to 2.4) | 0.3 (0 to 2.2) | 0.5 (0 to 2.6) | -1.1 (-95.6 to 1592.2) | -3.7 (-3879994.3 to 39489.2) | -4.5 (-95.8 to 1608.9) |
|  |  |  | All ages | 1.1 (0 to 6.1) | 0.4 (0 to 2.8) | 0.7 (0 to 3.4) | 3.2 (0 to 20.1) | 1.1 (0 to 9.2) | 2.1 (0 to 10.8) | 202.9 (-84.7 to 3893.1) | 182.3 (-13792928.1 to 74665.2) | 215.2 (-85.6 to 3893.1) |
|  |  | Deaths | Age-standardized | 0 (0 to 0.1) | 0 (0 to 0.1) | 0 (0 to 0.1) | 0 (0 to 0.1) | 0 (0 to 0.1) | 0 (0 to 0.1) | -7.1 (-98.3 to 2231.6) | -0.9 (-69134.6 to 69416.5) | -15.7 (-98.4 to 2468.2) |
|  |  |  | All ages | 0 (0 to 0.2) | 0 (0 to 0.1) | 0 (0 to 0.1) | 0.1 (0 to 0.7) | 0 (0 to 0.4) | 0.1 (0 to 0.3) | 249 (-93.9 to 5055.2) | 239.7 (-562774.1 to 189508.3) | 258.4 (-93.5 to 5347.9) |
|  | High alcohol use | DALYs (Disability-Adjusted Life Years) | Age-standardized | 0 (0 to 0) | 0 (0 to 0) | 0 (0 to 0) | 0.2 (0.1 to 0.3) | 0 (0 to 0.1) | 0.3 (0.2 to 0.5) | NA | NA | NA |
|  |  |  | All ages | 0 (0 to 0) | 0 (0 to 0) | 0 (0 to 0) | 1.8 (1 to 2.8) | 0.1 (0 to 0.3) | 1.6 (0.9 to 2.6) | NA | NA | NA |
|  |  | Deaths | Age-standardized | 0 (0 to 0) | 0 (0 to 0) | 0 (0 to 0) | 0 (0 to 0) | 0 (0 to 0) | 0 (0 to 0) | NA | NA | NA |
|  |  |  | All ages | 0 (0 to 0) | 0 (0 to 0) | 0 (0 to 0) | 0 (0 to 0.1) | 0 (0 to 0) | 0 (0 to 0.1) | NA | NA | NA |
|  | High body-mass index | DALYs (Disability-Adjusted Life Years) | Age-standardized | 3.9 (1.5 to 7) | 5.5 (2.1 to 9.8) | 1.9 (0.6 to 3.7) | 11.5 (4.8 to 20) | 14.5 (6.2 to 25.5) | 8.2 (3.2 to 14.4) | 190.4 (53.7 to 414) | 161.7 (24.2 to 373.9) | 341.4 (138 to 917) |
|  |  |  | All ages | 10.8 (4.3 to 18.4) | 8.1 (3.1 to 13.9) | 2.7 (1 to 5.2) | 96 (39.5 to 167.8) | 61.2 (26.2 to 107.6) | 34.7 (13.7 to 60.3) | 792.3 (399.2 to 1271) | 658.2 (291.1 to 1140.1) | 1197.4 (628.9 to 2611) |
|  |  | Deaths | Age-standardized | 0.2 (0.1 to 0.4) | 0.2 (0.1 to 0.5) | 0.1 (0 to 0.1) | 0.5 (0.2 to 1) | 0.8 (0.3 to 1.4) | 0.3 (0.1 to 0.5) | 210.2 (36 to 639.2) | 200.9 (11.3 to 628.9) | 405.4 (106.9 to 2066.8) |
|  |  |  | All ages | 0.3 (0.1 to 0.6) | 0.3 (0.1 to 0.6) | 0.1 (0 to 0.1) | 4 (1.7 to 7.2) | 3 (1.2 to 5.5) | 1 (0.4 to 1.9) | 1074.3 (459.2 to 2254) | 936.9 (321.7 to 2119) | 1839.4 (831.5 to 5005.9) |
|  | High systolic blood pressure | DALYs (Disability-Adjusted Life Years) | Age-standardized | 19.8 (6.3 to 34.2) | 22.3 (7 to 40) | 15.8 (4.8 to 28.4) | 21 (6.7 to 36.8) | 25.5 (7.8 to 44.4) | 15.9 (5.1 to 28.7) | 6.3 (-18 to 37.2) | 14.3 (-19.1 to 67) | 0.8 (-25.6 to 38.4) |
|  |  |  | All ages | 46.5 (15.2 to 80.6) | 28.7 (9.1 to 50.5) | 17.9 (5.3 to 31.8) | 173.1 (56.4 to 306.8) | 106.7 (33.8 to 187.1) | 66.5 (21.4 to 119.8) | 272.1 (189.7 to 378.1) | 272 (162.5 to 438.9) | 272.3 (177.9 to 400.2) |
|  |  | Deaths | Age-standardized | 1 (0.3 to 1.8) | 1.2 (0.4 to 2.3) | 0.6 (0.2 to 1.2) | 1 (0.3 to 1.7) | 1.4 (0.5 to 2.4) | 0.6 (0.2 to 1) | 2.3 (-31.4 to 54) | 15.7 (-28 to 91.9) | -11.7 (-46.5 to 68.1) |
|  |  |  | All ages | 1.7 (0.5 to 3.1) | 1.3 (0.4 to 2.4) | 0.5 (0.1 to 0.8) | 7.6 (2.5 to 13.2) | 5.6 (1.8 to 9.8) | 2 (0.7 to 3.7) | 338.4 (199.6 to 558.6) | 340.9 (176.5 to 633.4) | 331.3 (172 to 690.5) |
|  | Lead exposure | DALYs (Disability-Adjusted Life Years) | Age-standardized | 4 (-0.6 to 10) | 4.2 (-0.6 to 10.3) | 3.8 (-0.5 to 9.8) | 4.2 (-0.5 to 10.6) | 4.3 (-0.6 to 11) | 4 (-0.5 to 10) | 3.3 (-18.2 to 30.6) | 3 (-27.2 to 39.5) | 5 (-16.4 to 42.3) |
|  |  |  | All ages | 9.7 (-1.3 to 24.5) | 5.4 (-0.7 to 13.6) | 4.3 (-0.6 to 11) | 33.9 (-4.4 to 85.6) | 17.8 (-2.4 to 45.6) | 16.1 (-2.1 to 41.7) | 249.9 (182.3 to 326.8) | 229.1 (137.9 to 339.2) | 276.4 (205.1 to 391.6) |
|  |  | Deaths | Age-standardized | 0.2 (0 to 0.5) | 0.2 (0 to 0.6) | 0.2 (0 to 0.4) | 0.2 (0 to 0.5) | 0.3 (0 to 0.7) | 0.2 (0 to 0.4) | 5.9 (-25.3 to 54.2) | 13.5 (-27.4 to 69.8) | 0.4 (-35.4 to 89.1) |
|  |  |  | All ages | 0.3 (-0.1 to 0.9) | 0.2 (0 to 0.6) | 0.1 (0 to 0.3) | 1.5 (-0.2 to 4) | 1 (-0.1 to 2.6) | 0.5 (-0.1 to 1.4) | 345.1 (215 to 529.6) | 327.8 (171.9 to 538.6) | 382.8 (226.4 to 787.8) |
|  | Smoking | DALYs (Disability-Adjusted Life Years) | Age-standardized | 1.8 (1 to 2.8) | 0.7 (0.3 to 1.2) | 3.1 (1.7 to 5.1) | 1.9 (1 to 3) | 0.6 (0.3 to 1.1) | 3.1 (1.7 to 5) | 6.6 (-19.8 to 40.4) | -3.5 (-48.5 to 71.8) | 1.4 (-22 to 39.1) |
|  |  |  | All ages | 5.8 (3.3 to 9.1) | 1.1 (0.6 to 1.9) | 4.7 (2.6 to 7.5) | 18.1 (10.1 to 28.9) | 3 (1.4 to 5.3) | 15.2 (8.4 to 24.7) | 210.3 (134.9 to 307.5) | 168.9 (48.4 to 383) | 219.9 (142.8 to 327.9) |
|  |  | Deaths | Age-standardized | 0 (0 to 0.1) | 0 (0 to 0) | 0.1 (0 to 0.1) | 0 (0 to 0.1) | 0 (0 to 0) | 0.1 (0 to 0.1) | -2 (-41.1 to 74.2) | -2.5 (-62.1 to 146.6) | -11.3 (-47.2 to 70.7) |
|  |  |  | All ages | 0.1 (0.1 to 0.2) | 0 (0 to 0.1) | 0.1 (0 to 0.1) | 0.4 (0.2 to 0.6) | 0.1 (0 to 0.2) | 0.3 (0.1 to 0.5) | 240.6 (105.9 to 473.9) | 214.1 (32.7 to 628.9) | 250.3 (116.8 to 529.8) |
| Zanjan | All risk factors | DALYs (Disability-Adjusted Life Years) | Age-standardized | 21.2 (8.8 to 33.5) | 22.2 (9 to 37.6) | 19.6 (7.6 to 32.1) | 28 (14.4 to 42.6) | 29.5 (15 to 46.5) | 26 (13.5 to 39.4) | 32.3 (4.3 to 82.2) | 32.9 (-4.4 to 99) | 33.1 (4.2 to 97.4) |
|  |  |  | All ages | 60.5 (26.3 to 95.6) | 31.2 (13.3 to 51.7) | 29.4 (11.7 to 47.7) | 261.7 (136.6 to 395.4) | 145.3 (73.5 to 228.3) | 116.4 (61.2 to 177.6) | 332.3 (249.7 to 479.2) | 366 (237.5 to 568.4) | 296.5 (211 to 463.8) |
|  |  | Deaths | Age-standardized | 0.9 (0.3 to 1.5) | 1 (0.4 to 1.7) | 0.7 (0.2 to 1.2) | 1.2 (0.6 to 1.8) | 1.4 (0.6 to 2.3) | 0.9 (0.4 to 1.4) | 32.7 (-9.1 to 113.9) | 39.3 (-13.1 to 138.4) | 28.7 (-14.6 to 153.5) |
|  |  |  | All ages | 1.8 (0.7 to 3) | 1.2 (0.4 to 2) | 0.6 (0.2 to 1) | 10.1 (4.9 to 15.8) | 6.6 (3.1 to 10.9) | 3.5 (1.5 to 5.4) | 475.4 (293.5 to 794.1) | 467 (247.9 to 849.5) | 492.2 (304.4 to 1025.2) |
|  | Diet high in sodium | DALYs (Disability-Adjusted Life Years) | Age-standardized | 0.4 (0 to 2.2) | 0.2 (0 to 1.7) | 0.5 (0 to 2.8) | 0.4 (0 to 2.3) | 0.2 (0 to 1.8) | 0.5 (0 to 2.9) | 4.4 (-95.8 to 1524.5) | 1.2 (-2902.5 to 39509.3) | 5.5 (-96.1 to 1479.6) |
|  |  |  | All ages | 1.2 (0 to 6.6) | 0.4 (0 to 2.5) | 0.8 (0 to 4.2) | 3.6 (0 to 22) | 1.2 (0 to 9.1) | 2.4 (0 to 13.1) | 202.8 (-91 to 3687.2) | 236.7 (-3046.7 to 2601138.6) | 188.4 (-92 to 3703.6) |
|  |  | Deaths | Age-standardized | 0 (0 to 0.1) | 0 (0 to 0.1) | 0 (0 to 0.1) | 0 (0 to 0.1) | 0 (0 to 0.1) | 0 (0 to 0.1) | 8.5 (-96.8 to 3040.2) | 6.5 (-1469.3 to 29230.5) | 1.2 (-97 to 4463.2) |
|  |  |  | All ages | 0 (0 to 0.2) | 0 (0 to 0.1) | 0 (0 to 0.1) | 0.1 (0 to 0.8) | 0 (0 to 0.4) | 0.1 (0 to 0.4) | 312.3 (-92.6 to 7578.4) | 313.4 (-5125.5 to 358356) | 311.5 (-92.9 to 5879.1) |
|  | High alcohol use | DALYs (Disability-Adjusted Life Years) | Age-standardized | 0 (0 to 0) | 0 (0 to 0) | 0 (0 to 0) | 0.2 (0.1 to 0.3) | 0 (0 to 0.1) | 0.3 (0.2 to 0.5) | NA | NA | NA |
|  |  |  | All ages | 0 (0 to 0) | 0 (0 to 0) | 0 (0 to 0) | 1.8 (1 to 2.9) | 0.1 (0 to 0.3) | 1.7 (0.9 to 2.6) | NA | NA | NA |
|  |  | Deaths | Age-standardized | 0 (0 to 0) | 0 (0 to 0) | 0 (0 to 0) | 0 (0 to 0) | 0 (0 to 0) | 0 (0 to 0) | NA | NA | NA |
|  |  |  | All ages | 0 (0 to 0) | 0 (0 to 0) | 0 (0 to 0) | 0 (0 to 0.1) | 0 (0 to 0) | 0 (0 to 0.1) | NA | NA | NA |
|  | High body-mass index | DALYs (Disability-Adjusted Life Years) | Age-standardized | 2.5 (0.9 to 4.6) | 3.9 (1.4 to 7.2) | 0.9 (0.3 to 2) | 9.2 (3.6 to 16.2) | 10.8 (4.5 to 19.2) | 7.2 (2.9 to 13.1) | 271.3 (102.5 to 610.5) | 181.2 (41.5 to 415.2) | 699 (294.9 to 2113) |
|  |  |  | All ages | 8.1 (3 to 14.1) | 6.2 (2.4 to 10.7) | 1.9 (0.6 to 3.6) | 86.2 (34 to 152.5) | 53.8 (22.2 to 95.9) | 32.4 (12.7 to 58.1) | 968 (572.8 to 1719.7) | 769.9 (380.5 to 1360.5) | 1618.4 (887.7 to 4369.1) |
|  |  | Deaths | Age-standardized | 0.1 (0 to 0.2) | 0.1 (0 to 0.3) | 0 (0 to 0.1) | 0.4 (0.2 to 0.7) | 0.5 (0.2 to 0.9) | 0.3 (0.1 to 0.5) | 301.6 (67.2 to 1133.8) | 237.7 (31.8 to 891.2) | 1063.7 (271.3 to 6087.4) |
|  |  |  | All ages | 0.2 (0.1 to 0.4) | 0.2 (0.1 to 0.4) | 0 (0 to 0.1) | 3.3 (1.3 to 6) | 2.3 (0.9 to 4.2) | 1 (0.3 to 1.9) | 1452.3 (655.5 to 3436.5) | 1153.2 (434.3 to 2891.5) | 3397.5 (1428.1 to 11176.1) |
|  | High systolic blood pressure | DALYs (Disability-Adjusted Life Years) | Age-standardized | 18.8 (6 to 31.8) | 20 (6.3 to 35.7) | 17.2 (4.9 to 30) | 22.2 (7.6 to 36.9) | 23.9 (8.2 to 42.4) | 20 (6.9 to 34.4) | 17.7 (-8.2 to 52.1) | 19.5 (-16.1 to 70) | 16.8 (-9.2 to 62.1) |
|  |  |  | All ages | 52.1 (16.1 to 89) | 27.4 (9 to 49.5) | 24.6 (7.5 to 43.1) | 205.4 (70.7 to 343) | 117 (40.5 to 208) | 88.4 (30.7 to 151.5) | 294.4 (209.1 to 401.3) | 326.3 (198.7 to 491.7) | 258.9 (174.3 to 396) |
|  |  | Deaths | Age-standardized | 0.8 (0.3 to 1.4) | 0.9 (0.3 to 1.7) | 0.6 (0.1 to 1.1) | 0.9 (0.3 to 1.6) | 1.1 (0.4 to 2) | 0.7 (0.2 to 1.2) | 16.5 (-22.3 to 76.8) | 23.9 (-27.4 to 108.2) | 10.4 (-26 to 100.2) |
|  |  |  | All ages | 1.6 (0.5 to 2.8) | 1.1 (0.3 to 1.9) | 0.5 (0.1 to 0.9) | 8.1 (2.9 to 13.8) | 5.4 (1.8 to 9.7) | 2.7 (1 to 4.8) | 415.1 (245.1 to 669.7) | 409.9 (198.1 to 746.7) | 425.8 (245.1 to 873.7) |
|  | Lead exposure | DALYs (Disability-Adjusted Life Years) | Age-standardized | 3.9 (-0.5 to 9.5) | 3.8 (-0.5 to 9.7) | 4 (-0.5 to 9.8) | 4.2 (-0.6 to 10.6) | 4 (-0.6 to 10.6) | 4.4 (-0.6 to 10.9) | 9.5 (-12.2 to 36.2) | 6.7 (-22.1 to 46.4) | 11.4 (-9 to 45.7) |
|  |  |  | All ages | 10.7 (-1.4 to 26.4) | 5.2 (-0.7 to 13.5) | 5.5 (-0.7 to 14) | 38.7 (-5.2 to 97.1) | 19.5 (-2.7 to 51.3) | 19.2 (-2.6 to 47.6) | 262.1 (195.6 to 350.6) | 275.2 (179.9 to 405.2) | 249.7 (184.1 to 355.6) |
|  |  | Deaths | Age-standardized | 0.2 (0 to 0.4) | 0.2 (0 to 0.5) | 0.2 (0 to 0.4) | 0.2 (0 to 0.5) | 0.2 (0 to 0.6) | 0.2 (0 to 0.4) | 16.8 (-17.5 to 66.6) | 19.8 (-22.6 to 85.4) | 12 (-23.8 to 99.1) |
|  |  |  | All ages | 0.3 (0 to 0.8) | 0.2 (0 to 0.5) | 0.1 (0 to 0.3) | 1.7 (-0.3 to 4.1) | 1 (-0.2 to 2.7) | 0.7 (-0.1 to 1.6) | 411.8 (262.5 to 640.6) | 387.2 (210.3 to 649.7) | 453.9 (283.5 to 889.8) |
|  | Smoking | DALYs (Disability-Adjusted Life Years) | Age-standardized | 1.6 (0.9 to 2.5) | 0.5 (0.3 to 0.9) | 2.7 (1.5 to 4.4) | 2 (1.1 to 3.1) | 0.6 (0.3 to 1.1) | 3.4 (1.9 to 5.3) | 21.8 (-5 to 57.2) | 16.6 (-37.4 to 100.6) | 24.3 (-8.3 to 65.2) |
|  |  |  | All ages | 6.4 (3.6 to 10.2) | 1 (0.5 to 1.7) | 5.4 (3 to 8.7) | 20.2 (11.2 to 31.9) | 3.3 (1.7 to 5.7) | 16.9 (9.4 to 26.5) | 213.8 (144.3 to 301.2) | 225.6 (74.9 to 458.9) | 211.6 (131 to 311.6) |
|  |  | Deaths | Age-standardized | 0 (0 to 0.1) | 0 (0 to 0) | 0.1 (0 to 0.1) | 0 (0 to 0.1) | 0 (0 to 0) | 0.1 (0 to 0.1) | 29.3 (-19 to 120.3) | 19.6 (-48.1 to 171) | 20.2 (-30.2 to 128.4) |
|  |  |  | All ages | 0.1 (0.1 to 0.2) | 0 (0 to 0) | 0.1 (0 to 0.1) | 0.4 (0.2 to 0.7) | 0.1 (0 to 0.2) | 0.3 (0.2 to 0.6) | 321.7 (163.1 to 598.3) | 305.1 (78.4 to 787.5) | 326.4 (148.1 to 664.6) |
